# Germyliumylidene: A Versatile Low Valent Group 14 Catalyst

**DOI:** 10.1002/chem.202102233

**Published:** 2021-07-29

**Authors:** Debotra Sarkar, Sayan Dutta, Catherine Weetman, Emeric Schubert, Debasis Koley, Shigeyoshi Inoue

**Affiliations:** ^1^ Department of Chemistry WACKER-Institute of Silicon Chemistry and Catalysis Research Center Technische Universität München Lichtenbergstraße 4 85748 Garching Germany; ^2^ Department of Chemical Sciences Indian Institute of Science Education and Research (IISER) Kolkata Mohanpur 741 246 India; ^3^ Department of Pure and Applied Chemistry University of Strathclyde Glasgow G1 1XL UK

**Keywords:** catalysis, cations, CO_2_ conversion, DFT, germanium, N-heterocyclic carbenes

## Abstract

Bis‐NHC stabilized germyliumylidenes [RGe(NHC)_2_]^+^ are typically Lewis basic (LB) in nature, owing to their lone pair and coordination of two NHCs to the vacant p‐orbitals of the germanium center. However, they can also show Lewis acidity (LA) via Ge−C^NHC^ σ* orbital. Utilizing this unique electronic feature, we report the first example of bis‐NHC‐stabilized germyliumylidene [^Mes^TerGe(NHC)_2_]Cl (**1**), (^Mes^Ter=2,6‐(2,4,6‐Me_3_C_6_H_2_)_2_C_6_H_3_; NHC= IMe_4_=1,3,4,5‐tetramethylimidazol‐2‐ylidene) catalyzed reduction of CO_2_ with amines and arylsilane, which proceeds via its Lewis basic nature. In contrast, the Lewis acid nature of **1** is utilized in the catalyzed hydroboration and cyanosilylation of carbonyls, thus highlighting the versatile ambiphilic nature of bis‐NHC stabilized germyliumylidenes.

## Graphical abstract

**The versatile ambiphilic** catalytic application of a bis‐NHC‐stabilized germyliumylidene enables a range of reductive functionalization reactions, with a particular emphasis on C=O reduction that is, CO_2_ and carbonyls.

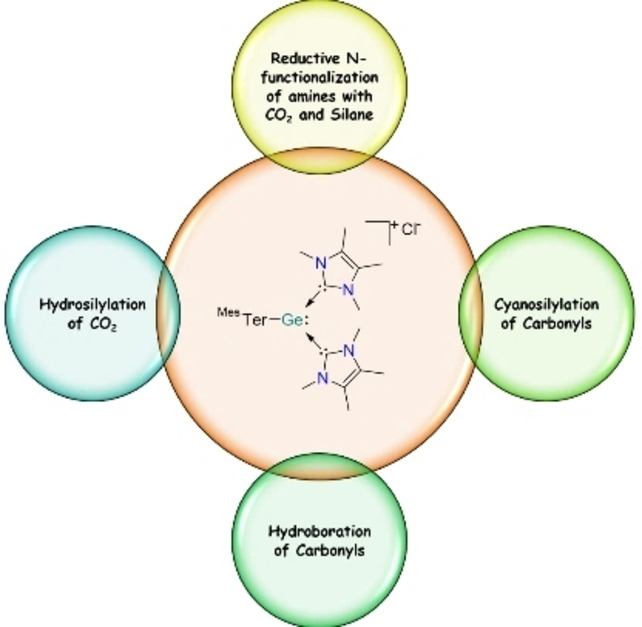

## Introduction

**Scheme 1:**
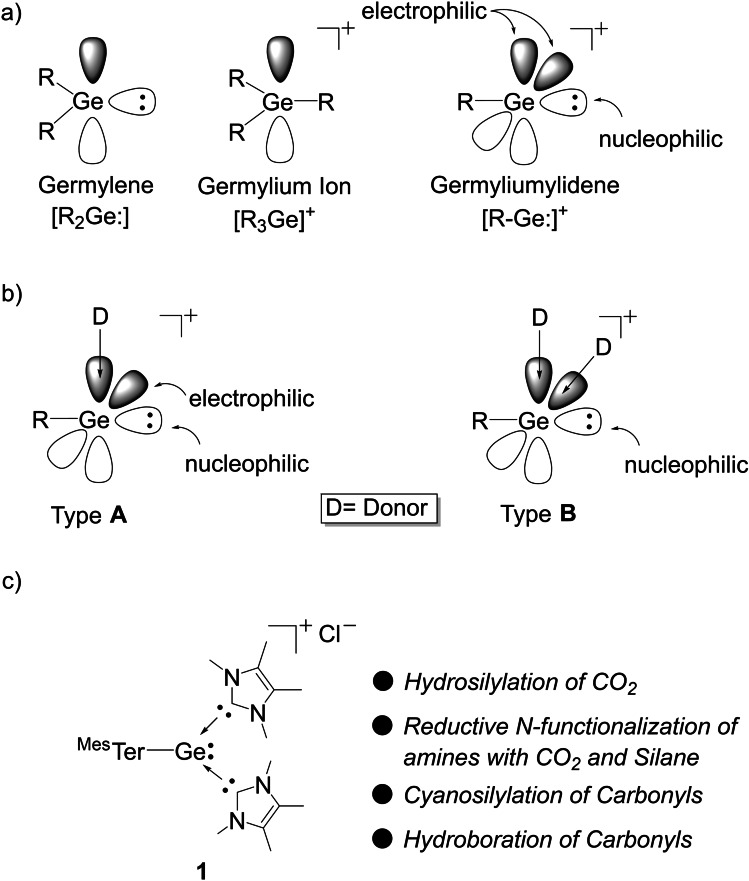
(a) Electronic features of germyliumylidene, (b) Lewis based stabilized germyliumylidenes, and (c) this work.

The ability of main group complexes to mimic transition metals has gained tremendous attention in recent years, driven by the desire for new sustainable processes.^[1]^ Activation of small molecules by low‐valent main group centers has been achieved and is the preliminary step towards transition metal‐free catalysis.[^1a^, ^1e^] However, their catalytic application is still limited due to challenges in reductive elimination from the resultant high‐oxidation state complex.[^1d^, ^1e^, ^2^] Recently, low valent germanium compounds have found themselves to be a diverse tool in enabling chemical transformations, attributed to the relative ease in which the +II and +IV oxidation states can be accessed.[^2b^, ^2c^, ^2d^, ^2e^, ^2f^, ^2g^, ^3^] This includes the first example of low‐valent main group dihydrogen activation and the use of multiple bonds (digermynes) in catalysis.[^2f^, ^3a^] Among the low valent germanium compounds, germyliumylidenes [R−Ge:]^+^ possess a unique electronic feature,^[4]^ due to the presence of a lone pair and two vacant p‐orbitals at the germanium center. It, therefore, combines the characteristics of germylium cations [R_3_Ge]^+^ and germylenes [R_2_Ge:] (Scheme 1a) and can simultaneously act as an electrophile and nucleophile,^[4]^ which has been further utilized in the activation of various small molecules,[^3e^, ^5^] including the thermodynamically robust H−H bond.^[3e]^

The reactivity of germyliumylidenes can be tuned depending on the number of donor ligands employed to stabilized the vacant p‐orbitals (Scheme 1b). Based on this, germyliumylidenes can be classified into two types, i) two‐coordinate germyliumylidenes, which have both electrophilic and nucleophilic centers (Scheme 1b, Type **A**), ii) three‐ coordinate germyliumylidenes where the nucleophilic character is more pronounced due to the occupancy of the two p‐orbitals (Scheme 1b, Type **B**).^[4]^

Among the type **B** classification, bis‐NHC stabilized germyliumylidenes are comparatively Lewis basic and relatively more stable than type **A** germyliumylidenes, due to the occupied p‐orbitals. Thus, on one hand they can be used as a robust Lewis base catalyst whilst on the other hand they can be used as a Lewis acid catalyst, due to the ability of the Ge−C^NHC^ σ* orbitals to accept electrons, which provides an additional cooperative site for potential catalytic application. Despite these unique electronic features, the catalytic application of germyliumylidenes is in its infancy. Recent examples from the groups of Rivard and Nagendran have shown the role of type **A** and **B** germyliumylidenes, respectively, in the hydroboration of carbonyls.^[6]^ However, the catalytic application of bis‐NHC‐stabilized germyliumylidenes is still unknown.^[5]^

Very recently, we have reported hydrosilylation of CO_2_ with a germa‐acylium ion (**I**, [MesTerGe(O)(NHC)_2_]Cl) (^Mes^Ter=2,6‐(2,4,6‐Me_3_C_6_H_2_)_2_C_6_H_3_; NHC=IMe_4_=1,3,4,5‐tetramethylimidazol‐2‐ylidene) which proceeds through the active germylene species (**II**, ^Mes^TerGe(OSiHPh_2_)(NHC)).^[7]^ Importantly, in our case the Lewis basicity of the Ge(II) center facilitates the hydride transfer from silane to CO_2_ via the formation of a hypercoordinate silane intermediate. Moreover, it has been shown that the Lewis acidity and Lewis basicity are both important for the catalytic transformation of CO_2_[^2d^, ^2e^, ^2h^, ^8^] This encouraged us to examine the recently reported bis NHC stabilized germyliumylidene [^Mes^TerGe(NHC)_2_]Cl (**1**) towards a range of catalytic reductive functionalization reactions, with a particular emphasis on C=O reduction i. e., CO_2_ and carbonyls.

## Results and Discussion

Following a similar protocol to the previous NHC‐stabilized germa‐acylium catalysis,^[7]^ compound **1** was found to transform CO_2_ into the corresponding hydrosilylated products in both a stoichiometric and catalytic manner in the presence of phenylsilane (PhSiH_3_). The ^1^H NMR spectrum revealed the complete consumption of PhSiH_3_ within 2.5 h at 60 °C, with the formation of silylformate, bis(silyl)acetal and silylated methanol observed (see Supporting Information, Figure S1). As expected, use of more sterically protected silanes required increased reaction times (PhSiH_3_ 2.5 h vs. Ph_2_SiH_2_ 3.5 h), and in the case of Ph_3_SiH, higher temperatures and prolonged reaction times are required (28 h at 80 °C). Furthermore, solvent optimization studies found increased rates of reaction in polar solvents (e. g., CD_3_CN) compared to non‐polar solvents (e. g., C_6_D_6_). This, however, can also be attributed to the low solubility of the catalyst in non‐polar solvents. To consider the influence of the counter ion in catalysis, the reaction was performed using the anion‐exchanged **1[BArF]**, [BArF={(3,5‐(CF_3_)_2_C_6_H_5_)_4_B], catalyst in CD_3_CN, no significant change in the rate of reaction was found. This points towards the dependence on the cationic germanium center during the catalysis. With the above points considered, use of 5 mol % of **1** with PhSiH_3_ in CD_3_CN at 60 °C provides the optimal reaction conditions for this study.

Whilst the catalytic activity of **1** is lower in comparison to our previously reported germa‐acylium ion catalyst (TOF: **I**=13.2 h^−1^ vs. **1**=7.9 h^−1^ for PhSiH_3_ at 60 °C),^[7]^ germyliumylidene **1** has the added advantage of being a more stable catalyst as well as requiring fewer synthetic steps. Using the optimized conditions mentioned above, the longevity of catalyst **1** was examined in which additional PhSiH_3_ and 1 bar of CO_2_ were added to the J‐Young NMR tube at the end of the cycle. This process could be repeated four times before a small drop in turnover was observed (TOF: Run 1=8 h^−1^ vs. Run 4=6 h^−1^). In contrast, the previously reported germa‐acylium ion catalyst decomposed after the third cycle.

**Figure 1:**
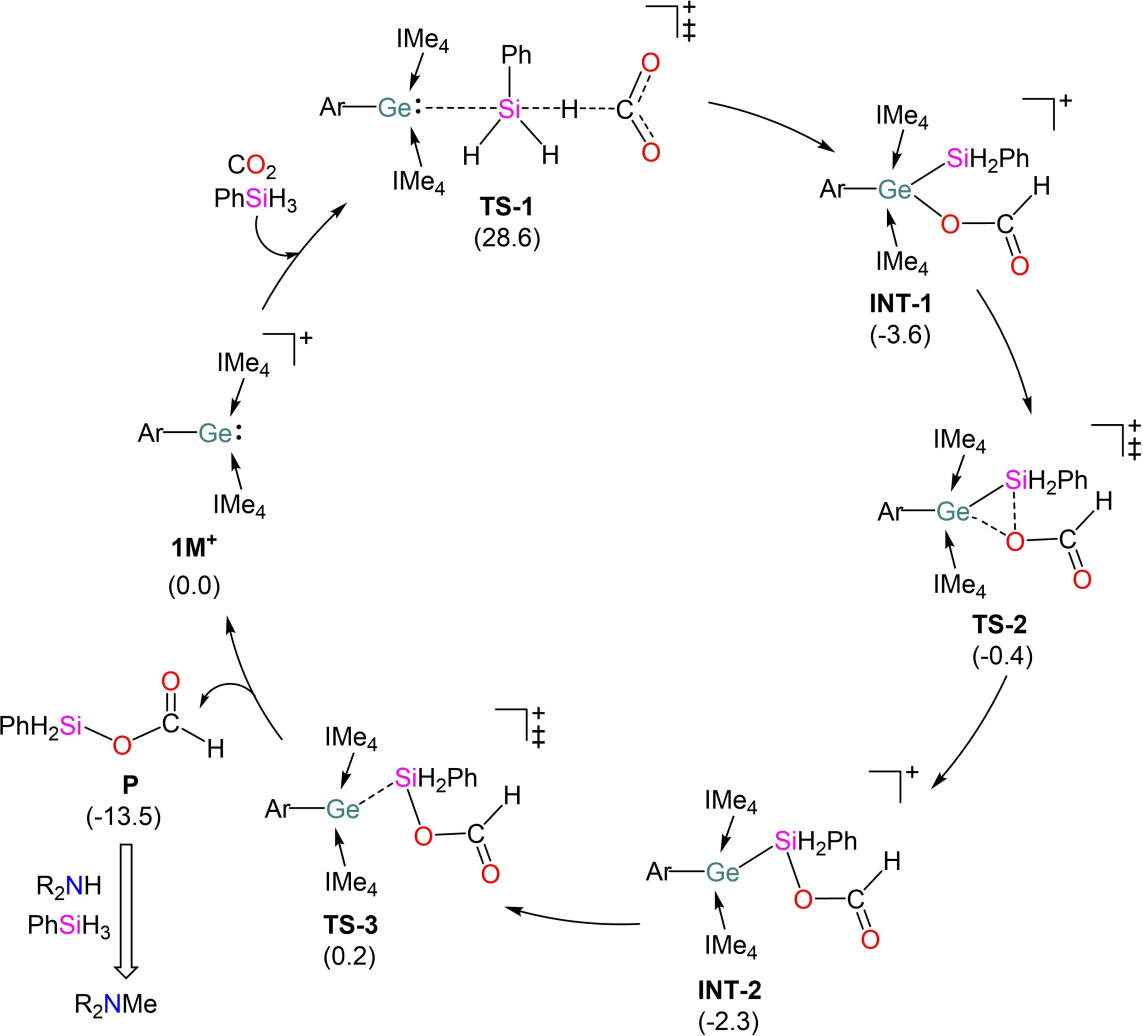
Proposed mechanism of **1M^+^**‐catalyzed N‐functionalization of amines with CO_2_ and PhSiH_3_. Δ*G*_L_^S^ (kcal mol^−1^) values are given in brackets. Ar=2,6‐dimethylphenyl; R=−Et.

A series of stoichiometric reactions were undertaken to probe the mechanism. No reaction was observed with **1** and CO_2_ in the absence of silane, even after prolonged heating at 60 °C. Additionally, no reaction was observed with varying equivalents of hydrosilane under the optimal catalytic conditions. This suggests a cooperative silane/CO_2_ mechanism, and therefore, the mechanism was investigated computationally (Figure S50). In a similar fashion to the previous case,^[7]^ the Si−H bond in PhSiH_3_ is activated by the germanium lone pair in **1M^+^** and the hydride transfer from the hypercoordinate silane to the free CO_2_ occurs in a concerted process via the transition state **TS‐1** (Figure 1). IRC calculations confirm the direct formation of the transition state from the separated species (**1M^+^** +PhSiH_3_+CO_2_) following a three‐component mechanism. Additionally, participation of the germanium lone pair in “concerted S_N_2@Si‐acceptor” mechanism^[9]^ rather than the classical activation of hydrosilanes is also clearly evident from the substantially longer Ge−Si distance (2.787 Å) in **TS‐1**. This step needs to surmount an energy barrier of 28.6 kcal mol^−1^ to provide the resulting intermediate (**INT‐1**). The Lewis basicity of NHC‐stabilized germyliumylidene towards PhSiH_3_ cannot be explained by drastically high energy separation (11.0 eV) between the germanium lone pair orbital in free **1M^+^** and Si−H σ* orbital in free PhSiH_3_ (Figure S51a). However, when the silane approaches the Ge center, significant stabilization of the Si−H σ* orbital in the PhSiH_3_ fragment of **TS‐1** results in favorable interaction (8.3 eV) with the germanium lone pair in the **1M^+^** fragment, thus enhancing the reactivity of germyliumylidene and highlighting the Lewis basic nature of **1M^+^** (Figure S51b). In the alternative pathway, oxidative addition of silane across the Ge center in **1M^+^** with concomitant liberation of IMe_4_ demands slightly lesser energy barrier of 28.2 kcal mol^−1^ (Figure S52), as suggested by favorable interaction (8.2 eV) between the Si−H σ and Ge−C^IMe4^ σ* orbitals in **TS‐7** (Figure S53b). However, CO_2_ insertion into the Ge(IV) hydride species (**INT‐5**) in the subsequent step demands drastically high intrinsic activation barrier of 44.9 kcal mol^−1^ and can be safely discarded. Moreover, **1M^+^** does not coordinate with either CO_2_ or silane. Thus **1[Cl]** assisted hydrosilylation of CO_2_ is proposed to occur through three‐component pathway rather than a two component mechanism. In agreement with experimental findings, the computed activation barriers for **TS‐1** increase with decreasing number of hydrides on the silane (PhSiH_3_: 28.6 kcal mol^−1^<Ph_2_SiH_2_: 31.4 kcal mol^−1^<Ph_3_SiH: 34.7 kcal mol^−1^, Figure S54). **INT‐1** finally delivers the formoxysilane (**P**) accompanying an energy barrier of 3.8 kcal mol^−1^.

Further comparisons to the previous germa‐acylium ion catalysis were sought through the extension to reductive N‐functionalization of amines.^[7]^ Accordingly, following an initial optimization study, we examined the scope of the reaction with various amines as listed in Table 1. The study revealed that aliphatic secondary amines proceed smoothly compared to aromatic secondary amines. For example, the aliphatic amines (Table 1, Entry 1–4) are converted to corresponding 2e^−^ (formamide), 4e^−^ (aminal) and 6e‐(methylamine) reduced products within 2 h, whereas N‐methylaniline (Table 1, Entry 5) requires 16 h. This is possibly attributed to the low nucleophilicity of the aromatic amine arising from the higher inductive effects of the phenyl ring compared to alkyl groups.^[10]^ In comparison to the previous germa‐acylium ion (**I**) lower activity was again observed for reductive N‐functionalization catalysis. This may, in part, be due to the role of the Ge center in the subsequent conversion of the formoxysilane product to the functionalized amine. In this system no metal center is implicated in the computed mechanism and formation of PhSiH_2_OH requires a slightly higher energy barrier for the reduction of formamide compared to the former germa‐acylium ion system.^[7]^

**Table 1:** N‐Methylation of amines using 1 bar CO_2_, 3 equiv. PhSiH_3_, in CD_3_CN and 5 mol% of **1** (All reactions carried out at 60 °C). TOF=(Conversion/catalyst loading)/time.

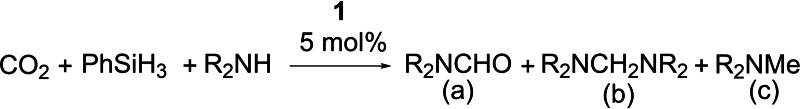					
Entry	Amine		NMR Yield [%]		TOF [h^−1^]
		a	b	c	
1	diethylamine	73	4	22	9.4
2	piperidine	82	0	16	9.4
3	morpholine	81	0	16	9.4
4	dicyclohexylamine	47	0	47	19.8
5	N‐methylaniline	70	25	4	1.25

Germyliumylidiene catalyst **1**, whilst not as active as the germa‐acylium system,^[7]^ its increased stability with regard to subsequent cycles prompted us expand the scope of the reactivity towards alternative reductive catalytic cycles. Cyanosilylation is one of the most fundamental carbon‐carbon bond forming reactions in organic chemistry, where the resulting cyanohydrin silylether [R_2_C(OTMS)CN] serves as a synthon for numerous biologically relevant molecules such as α‐hydroxyacids, α‐amino acids, and β‐amino alcohols.^[11]^ Catalysts for cyanosilylation of carbonyls typically utilize transition metals,^[11]^ whilst in contrast, only a handful of heavier p‐block compounds have been exploited as a single site cyanosilylation catalyst.[^2c^, ^2g^, ^12^] In this context, Khan and co‐workers recently demonstrated the role of a neutral NHC‐stabilized germylene in the catalyzed cyanosilylation of aldehydes. Here, the Lewis acidity of germanium facilitated the cyanide transfer to the carbonyl moiety via formation of a donor‐accepter complex [TMSCN→Ge(II)].^[2g]^ We, therefore, envisaged enhanced activity of germyliumylidenes over germylenes, due to the cationic germanium center. Accordingly, cyanosilylation was performed with various carbonyls, as listed in Table 2.

**Table 2:** Cyanosilylation: carbonyl (1.00 mmol), TMSCN (1.02 mmol), solvent (0.4 mL CD_3_CN), temperature (28±2 °C) and 0.1 mol % of **1**. TOF=(Conversion/catalyst loading)/time.^[a]^

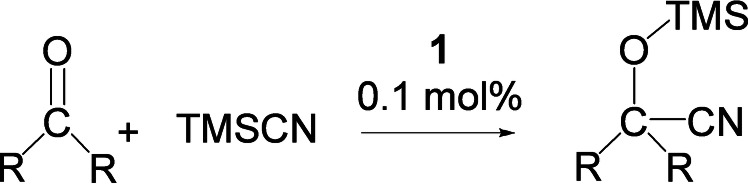				
Entry	Aldehyde	Conversion [%]	Time [h]	TOF [h^−1^]
1	propanal	>99	0.55	1800
2	pivaldehyde	>99	0.50	1900
3	cyclohexanecarboxaldehyde	>99	0.55	1800
4	4‐pyridinecarboxaldehyde	>99	0.50	1900
5	benzaldehyde	>99	1.1	900
6	4‐cyanobenzaldehyde	>99	1.5	660
7	2‐naphthalenecarboxaldehyde	>99	1.5	660
8	benzophenone	>99	4	25
9	acetophenone	>99	14	7
10	4‐fluroacetophenone	>99	16	6
11	4‐methoxyacetophenone	>99	15	6.6

[a] For ketones 1.00 mol % of **1** and 50 °C temperature was required.

Catalytic cyanosilylation of aldehydes and ketones, mediated by **1**, proceeds under mild conditions compared to the previously reported germylenes and transition metal catalysts.[^2c^, ^2g^, ^13^] Notably, in our case, low catalyst loadings (0.1 mol %) and reduced reaction times (≤90 min) are required for complete conversion of both aromatic and aliphatic aldehydes, yielding the corresponding cyanohydrin silylether (for aliphatic aldehydes TOF=≥1800 h^−1^ and aromatic aldehyde TOF=900 h^−1^). For benzophenone, increased catalyst loadings (1 mol %) and higher temperatures (50 °C) are required, likely due to the increased steric protection around the carbonyl moiety.

**Figure 2:**
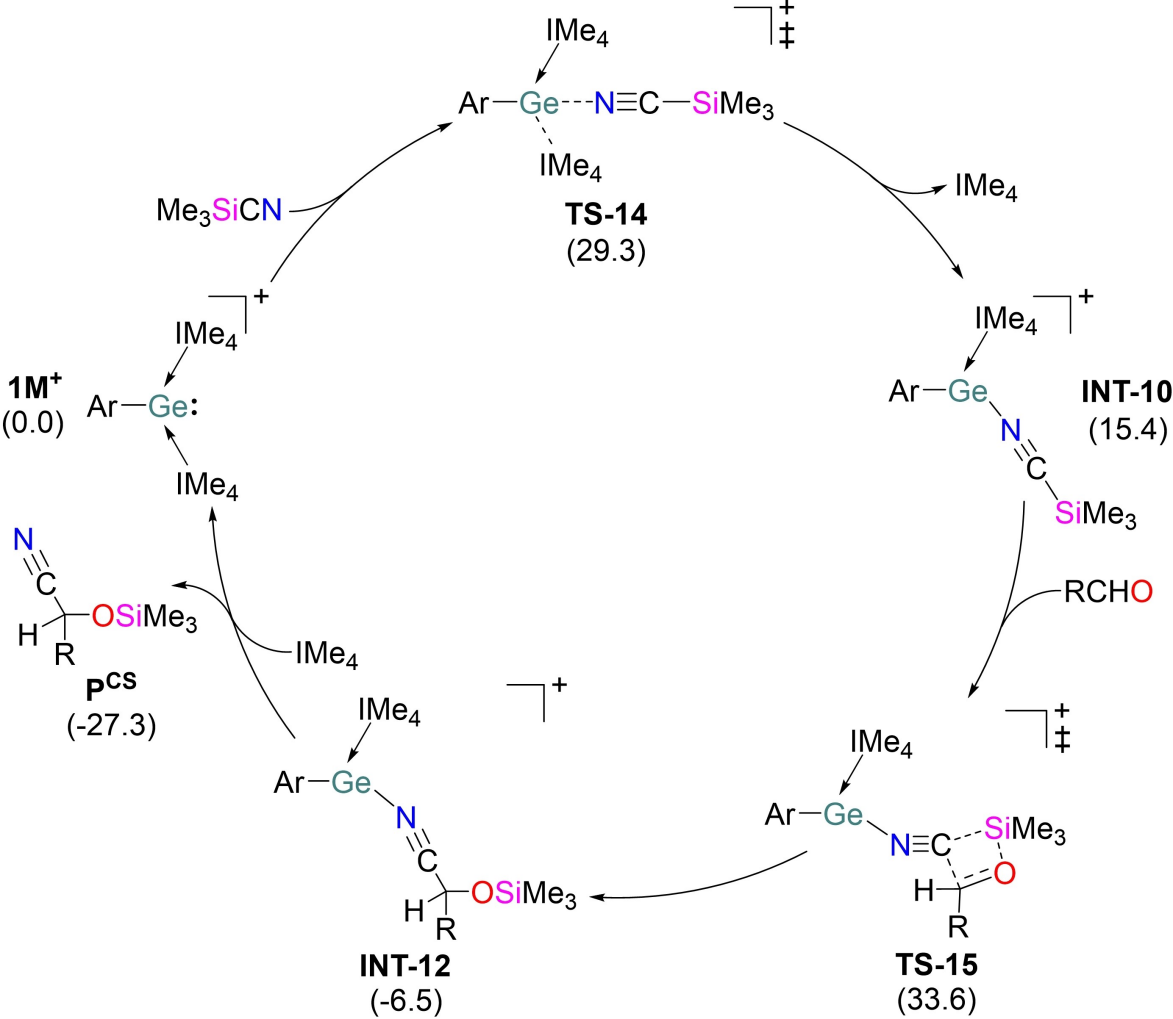
Proposed mechanism of **1M^+^**‐catalyzed cyanosilylation of aldehyde. Δ*G*_L_^S^ (kcal mol^−1^) values are given in brackets. R=−^*t*^Bu.

DFT studies indicate that initial coordination of Me_3_SiCN to the Ge center via **TS‐14** followed by de‐coordination of IMe_4_, leads to the formation of **INT‐10** (Figure 2, Figure S57). The intermediate **INT‐10** lies 15.4 kcal mol^−1^ higher in energy than the initial reactants and several attempts to isolate the **INT‐10** in presence of stoichiometric or excess Me_3_SiCN were unsuccessful. The transition vector in **TS‐14** animates the Ge−N bond formation (2.692 Å) with concomitant elongation of the Ge−C^IMe4^ bonds (2.372/2.313 Å) compared to those in **1M^+^** (2.076/2.066 Å). The significant weakening of the C^IMe4^→Ge interactions in **TS‐14** is also reflected in the Wiberg bond indices calculated for these bonds (**TS‐14**: 0.486/0.529; **1M^+^**: 0.715/0.720). NOCV calculations reveal that the strongest orbital interaction in **TS‐14** originates from the σ‐donation of nitrogen lone pair in Me_3_SiCN to the Ge−C^IMe4^ σ* orbitals in **1M^+^** (Figure S58). Analogous to the previous hydrosilylation mechanism, the electrophilic nature of **1M^+^** cannot be explained by the orbital interactions between free **1M^+^** and Me_3_SiCN species (Figure S60a). The promotion of the **1M^+^** fragment from its ground state equilibrium geometry to the perturbed form in **TS‐14**, as Me_3_SiCN approaches the Ge center, results in lowering of the Ge−C^IMe4^ σ* orbital from the LUMO+10 in the free **1M^+^** to LUMO in the **1M^+^** fragment of **TS‐14** (Figure S60b).^[14]^ The significant stabilization of the Ge−C^IMe4^ σ* orbital in **TS‐14** allows the germyliumylidene to exhibit its Lewis acidic nature towards Me_3_SiCN. The incoming carbonyl essentially inserts into the C−Si bond of NCSiMe_3_ via a 4‐membered cyclic transition state (**TS‐15**). This step needs to overcome overall energy barrier of 33.6 kcal mol^−1^ to furnish the substantially more stable intermediate (**INT‐12**). Similar moderately high activation barrier for cyanosilylation reactions at room temperature have been reported earlier.[^12a^, ^12b^] Finally, re‐coordination of IMe_4_ and a product de‐coordination step to deliver the desired cyanohydrin product accompanies an energy barrier of 7.5 kcal mol^−1^ In the alternative pathway, the substitution of IMe_4_ in **1M^+^** by RCHO demands slightly lower energy barrier of 28.6 kcal mol^−1^ compared to that by Me_3_SiCN (Figure S62). However, the Si−CN bond activation in the incoming Me_3_SiCN by free NHC in the subsequent step needs to surmount a significantly higher energy barrier of 37.1 kcal mol^−1^ and hence, the initial coordination of RCHO to the Ge center in **1M^+^** via ligand exchange can be safely discarded. Moreover, theoretical endeavor to optimize the transition state similar to **TS‐1** (Figure 1), depicting the activation of Si−CN bond in Me_3_SiCN by the germanium lone pair in **1M^+^** and concomitant −CN transfer to RCHO remained unsuccessful. Special attention is also directed towards the nucleophilic attack of germanium lone pair in **1M^+^** to the silicon center in Me_3_SiCN (Figure S73). However, geometry optimization of such hypercoordinate silane adduct (**INT‐37**) remained unsuccessful at the R‐BP86/def2‐SVP level. Importantly, we were able to optimize **INT‐37** at the R‐M06‐2X‐D3/def2‐TZVP level. Our calculations suggest that the formation of **INT‐37** is extremely endergonic by 29.5 kcal mol^−1^ and its generation also demands an activation barrier of 30.8 kcal mol^−1^ (Figure S74). The subsequent −CN transfer to the carbonyl carbon in the incoming substrate demands an energy barrier of 32.9 kcal mol^−1^. Importantly, the favorable pathway for the cyanosilylation reaction (Figure S57) shows the rate‐limiting energy barrier of 31.8 kcal mol^−1^ at the same level of theory. Moreover, structural optimization of the intermediate generated by the nucleophilic attack of **1M^+^** to the RCHO failed despite several attempts (Figure S73). Hence, the Lewis basicity of the germyliumylidene for the cyanosilylation reaction is less likely to operate on kinetic ground.

Given the current interest in hydroboration as an attractive method for mild and selective reduction of carbonyls, we were interested to see how our germyliumylidiene catalyst performed. Most relevant to this work is the previous reports of neutral tetrylene catalysts (R_2_E:, E=Si−Sn)[^2b^, ^15^] and the recent examples of cationic Ge(II) metal centers for hydroboration of carbonyls.^[6]^ Encouraged by these studies, we screened catalytic activity of **1** towards various aldehydes with pinacol borane (HBpin) as the hydroboration reagent. In line with previous studies, less bulky substituents (e. g., propanal, Table 3, entry 1) proceed rapidly with low catalyst loadings (0.1 mol %), whereas more sterically demanding substrates (e. g., ^*t*^BuCHO, Table 3, entry 2) require longer reaction times. Notably, this catalysis proceeds with much lower catalyst loadings than those previously reported for cationic germanium catalysts.^[6b]^

**Table 3:** Hydroboration: Aldehyde (1.00 mmol), HBpin (1.02 mmol), solvent (0.4 mL CD_3_CN), temperature (28±2 °C) and 0.1 mol  % of catalyst. TOF=(Conversion/catalyst loading)/time.

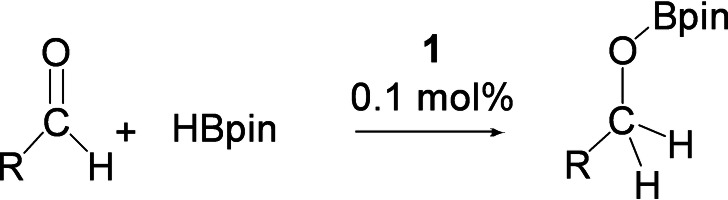				
Entry	Aldehyde	Conversion [%]	Time [h]	TOF [h^−1^]
1	propanal	>99	0.1	>5900
2	pivaldehyde	>92	24	38
3	cyclohexanecarboxaldehyde	>99	4	250
4	4‐pyridinecarboxaldehyde	>99	3.5	280
5	benzaldehyde	>85	0.5	1700
6	4‐cyanobenzaldehyde	>99	0.6	1600
7	2‐naphthalenecarboxaldehyde	>99	0.6	1600

**Figure 3:**
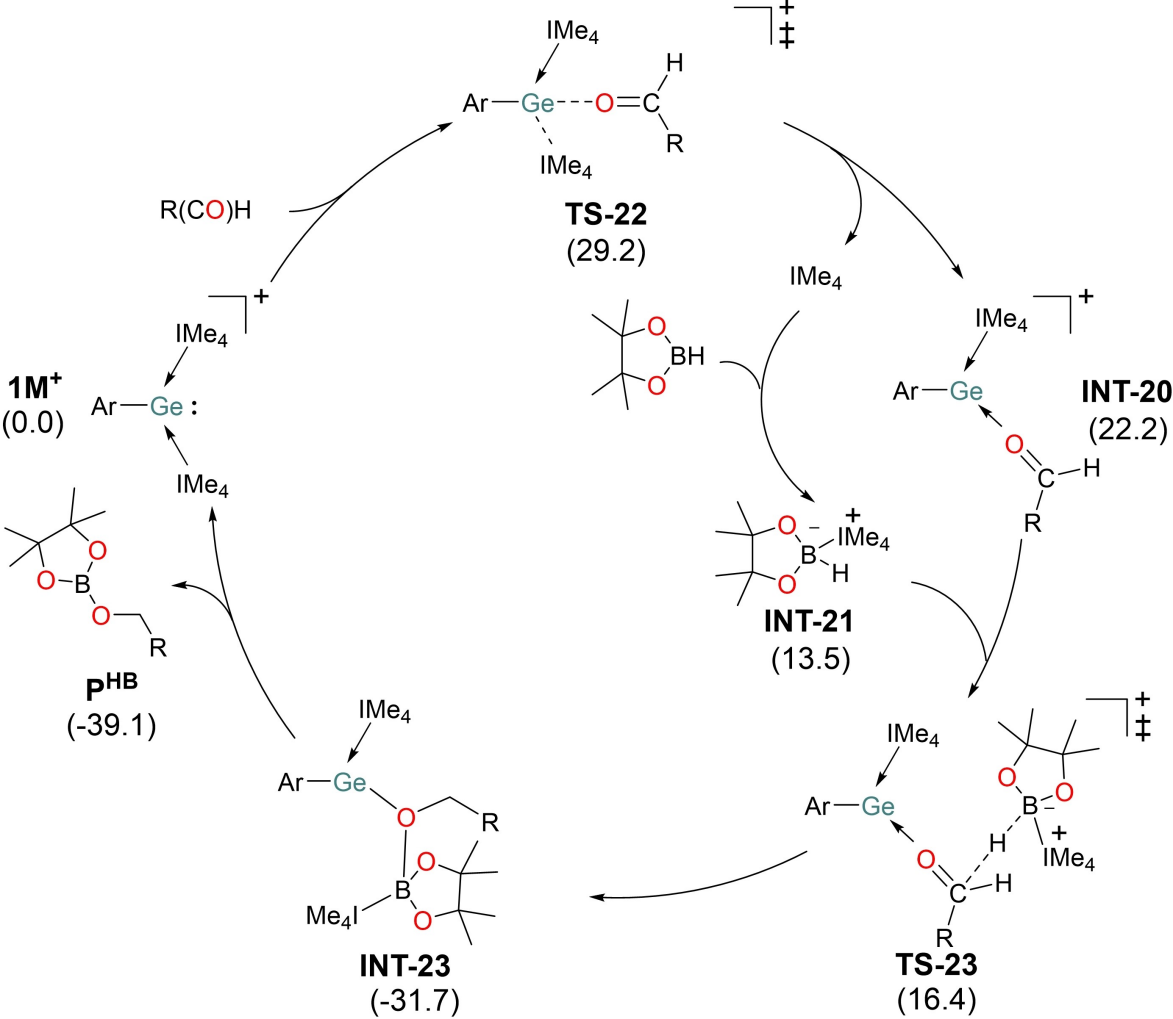
Proposed mechanism of **1M^+^**‐catalyzed hydroboration of aldehyde. Δ*G*_L_^S^ (kcal mol^−1^) values are given in brackets. R=−Et.

Similar to the cyanosilylation mechanism, the favorable pathway of hydroboration of aldehyde shows the initiation of the reaction by substitution of IMe_4_ by RCHO via **TS‐22** accompanying the rate‐limiting activation barrier of 29.2 kcal mol^−1^ (Figure 3 and S64). The strongest orbital interaction in **TS‐22** is associated with the charge flow from the oxygen lone pair in RCHO into the Ge−C^IMe4^ σ* orbitals in **1M^+^**, as suggested by NOCV calculations (Figure S65). Similar to **TS‐14**, the unique bonding features of the **1M^+^** and RCHO fragments in **TS‐22** can nicely explain electrophilic nature of germyliumylidene in hydroboration reactions. The significant stabilization of Ge−C^IMe4^ σ* orbital in the **1M^+^** fragment of **TS‐22** compared to that in the free **1M^+^** facilitates the nucleophilic attack of aldehyde (Figure S66). The subsequent B−H bond activation in HBpin is mediated by the free IMe_4_.[^14b^, ^16^] The adduct **INT‐21** transfers the hydride to the carbonyl carbon in **INT‐20**. This is followed by the coordination of boron to the oxygen center generating the substantially stable intermediate **INT‐23**. Finally, the IMe_4_ ligand transfer to the Ge center demands an energy barrier of 26.8 kcal mol^−1^ to afford the desired product and regenerate **1M^+^**. Notably, unlike transition metals,^[17]^ metal‐ligand cooperativity in low valent group 14 complexes is rare^[15a]^ and this provides a unique example of this cooperativity and its pivotal role in catalysis.Whilst the IMe_4_ ligand plays a key role in this transformation, subsequent control experiments highlight the requirement for the Ge center (0.1 mol % of IMe_4_ the TOF for hydroboration of benzaldehyde 50 h^−1^).

## Conclusion

In conclusion, we have demonstrated for the first time the utilization of a well‐defined low valent group 14 tetryliumylidene complex for diverse organic transformations. This includes the germyliumylidene (**1**)‐catalyzed hydrosilylation of CO_2_ and N‐methylation of amines using CO_2_ as a C1 source, where **1** acts as a Lewis base towards silane due to the presence of a lone pair on the germanium atom. Additionally, exploiting the electronic features of **1** other organic transformations, such as cyanosilylation and hydroboration of carbonyls, have been achieved under mild conditions. This is possible due to the accessible Ge−C^NHC^ σ* orbitals, which allow **1** to exhibit its electrophilic nature in cyanosilylation and hydroboration reactions. With the observed high catalytic activity and versatile transformations, this NHC‐stabilized germyliumylidene catalyst provides a viable alternative towards transition metal‐free catalysis.

## Conflict of interest

The authors declare no conflict of interest.

## Supporting information

As a service to our authors and readers, this journal provides supporting information supplied by the authors. Such materials are peer reviewed and may be re‐organized for online delivery, but are not copy‐edited or typeset. Technical support issues arising from supporting information (other than missing files) should be addressed to the authors.

Supporting Information
