# Supplementary material for: Germyliumylidene: A Versatile Low Valent Group 14 Catalyst
Source: Chemistry. 2021 Jul 29;27(51):13072–8. doi: 10.1002/chem.202102233 (PMC8518661; doi:10.1002/chem.202102233)
Supplement: Supplementary file 1 — Supporting Information [file CHEM-27-13072-s001.pdf]

Chemistry–A European Journal

Supporting Information

Germyliumylidene: A Versatile Low Valent Group 14 Catalyst

Debotra Sarkar, Sayan Dutta, Catherine Weetman, Emeric Schubert, Debasis Koley,* and Shigeyoshi Inoue*

Table of Contents

1. Experimental details S2
1.1. General information S2
1.1.1 General procedure for catalytic hydrosilylation of CO_2 .... S2
1.1.2. General procedure for catalytic N-functionalization amines with CO_2 .... S3
1.1.3. General procedure for catalytic cyanosilylation of carbonyls S8
1.1.4. General procedure for catalytic hydroboration of carbonyls S19
2. DFT Calculations S27
3. References S119

1. Experimental details

1.1. General information

All experiments and manipulations were carried out under dry oxygen-free argon atmosphere using standard Schlenk techniques or in a glovebox. The ^{1}H, ^{13}C{^{1}H} NMR spectra of the compounds were measured on Bruker 400 MHz and 500 MHz spectrometer. Chemical shifts are referenced to (residual) solvent signals (^{1}H and ^{13}C{^{1}H} NMR). Deuterated solvent CD_3CN was obtained from Deutero Deutschland GmbH and dried over 4 Å molecular sieves. Carbon dioxide (5.0) was purchased from Westfalen AG and used as received. Unless otherwise stated, all reagents were purchased from commercial sources and used as received. Germyliumylidene [{^{m}TerGe(IMe$_4$)$_2$Cl] **1** was synthesized according to the literature procedure.[S1]

1.1.1 General procedure for catalytic hydrosilylation of CO_2

Compound **1** (5.0 mol %) in 0.5 mL CD_3CN, silane (10 mg, .03 mmol) were added to a J-Young NMR tube. The solution was freeze-pump thaw and degassed two times before being refilled with 1 bar of CO_2. The ^{1}H NMR spectrum was recorded. Then the tube was placed in a preheated oil bath (60 °C). Notably, for Ph_3SiH hydrosilylation of CO_2 was performed at 80 °C temperature, so the heating bath was adjusted accordingly. The reactions were monitored by ^{1}H NMR spectroscopy at regular intervals until the silane was consumed.

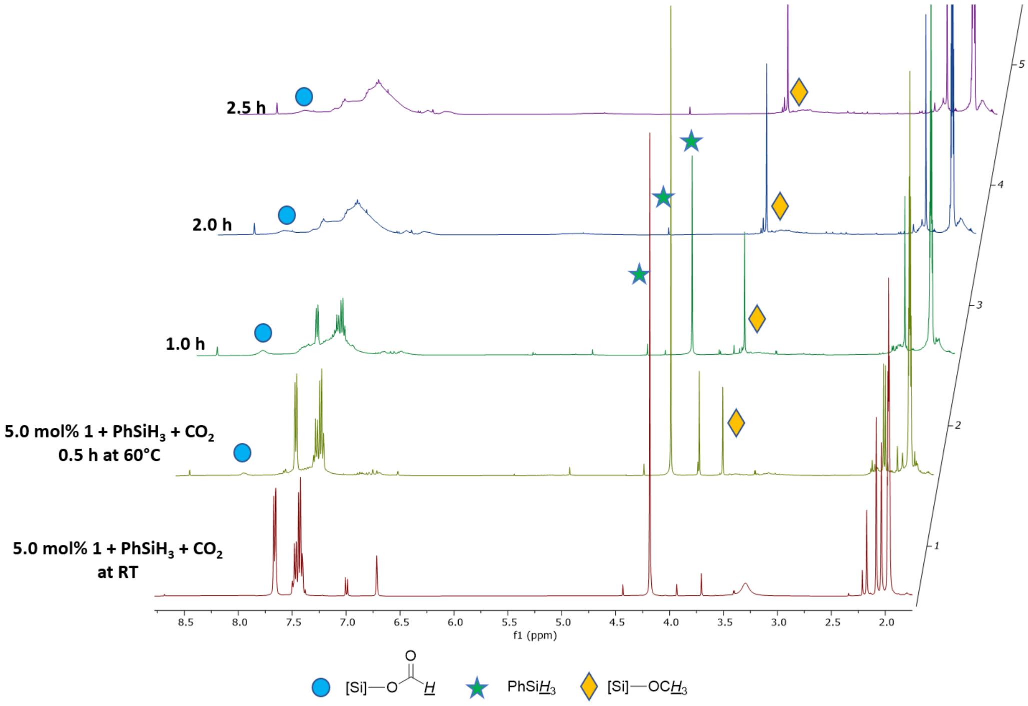

Figure S1. Stacked ^{1}H NMR spectra, compound **1** (5.0 mol%) in CD_3CN, with $PhSiH_3$ (10.0 mg) and CO_2 (1 bar).

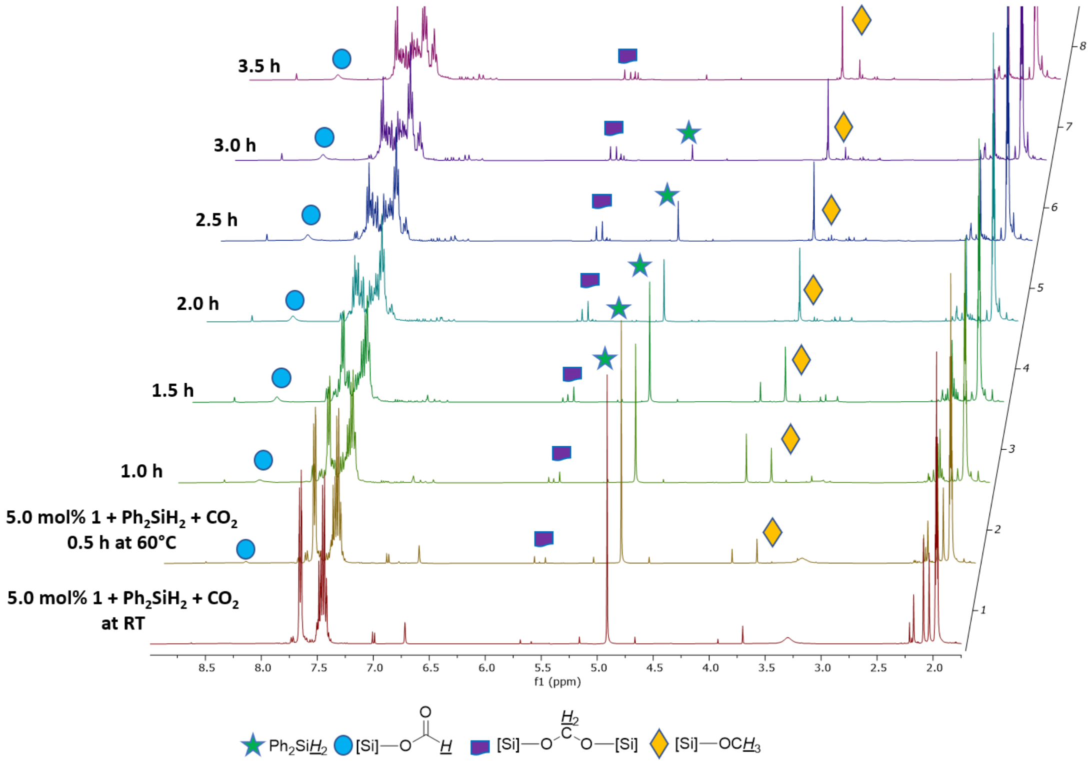

Figure S2. Stacked ^{1}H NMR spectra, compound **1** (5.0 mol%) in CD_3CN, with Ph_2SiH_2 (10.0 mg) and CO_2 (1 bar).

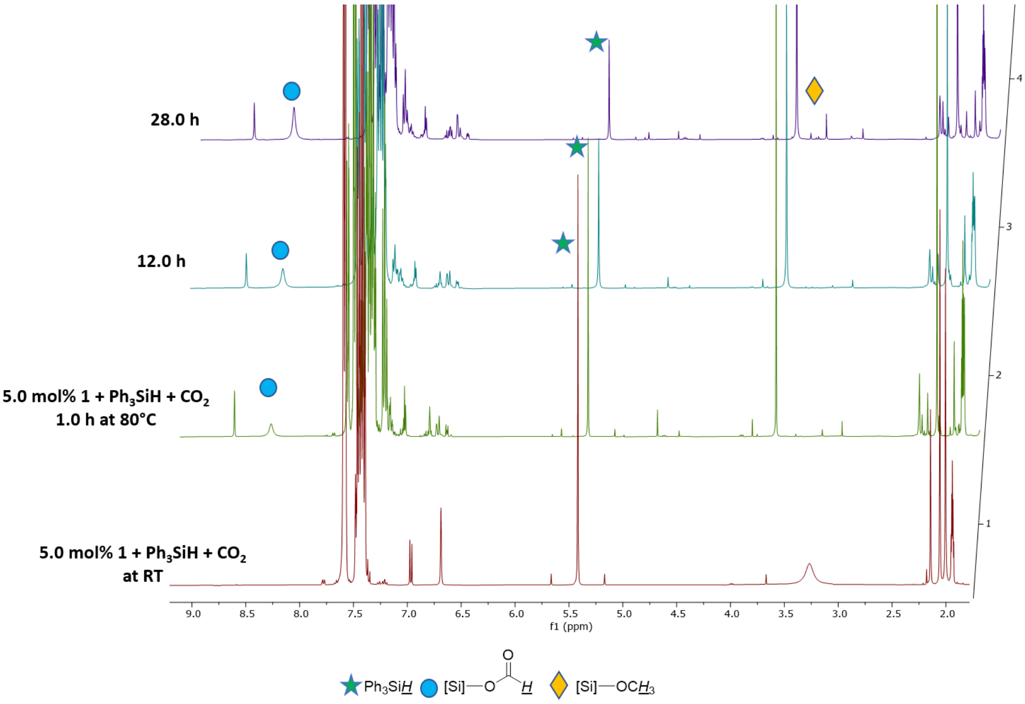

Figure S3. Stacked ^{1}H NMR spectra, compound **1** (5.0 mol%) in CD_3CN, with Ph_3SiH (10.0 mg) and CO_2 (1 bar), full conversion reaction time was not measured.

1.1.2. General procedure for catalytic N-functionalization amines with CO_2

We have performed the N-functionalization of amine with CO_2 at 60 °C. Therefore, all samples were prepared the following way. Compound **1** (5 mol %) in 0.5 mL CD_3CN, amine (10 mg, 1 eq), silane (3 eq) were added to the J-Young NMR tube. The solution was freeze-pump-thaw degassed two times before being refilled with 1 bar of CO_2.

The reactions was monitored by ^{1}H NMR spectroscopy. For the elevated temperature measurement, the tube was placed in a preheated oil bath (60 °C) and monitored regularly.

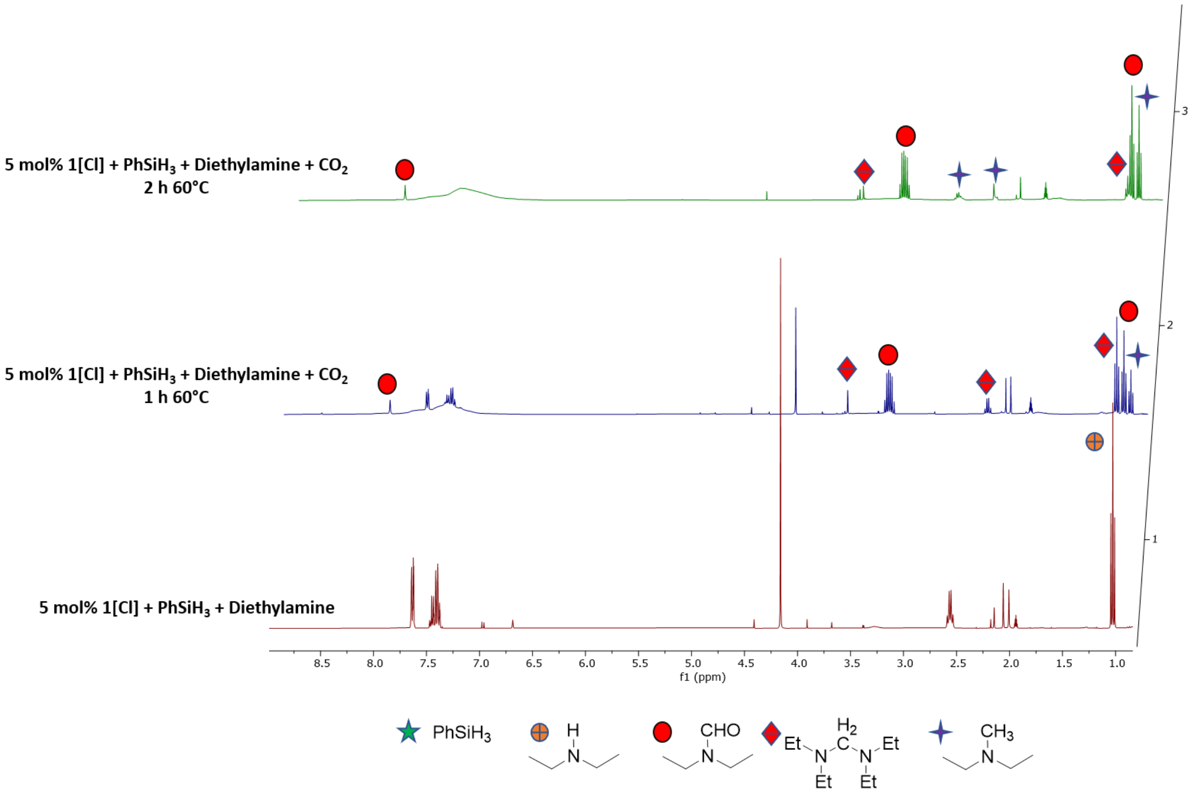

Figure S4. Stacked ^{1}H NMR spectra of reductive functionalization of CO_2 with diethylamine and $PhSiH_3$ at 60 °C in CD_3CN.

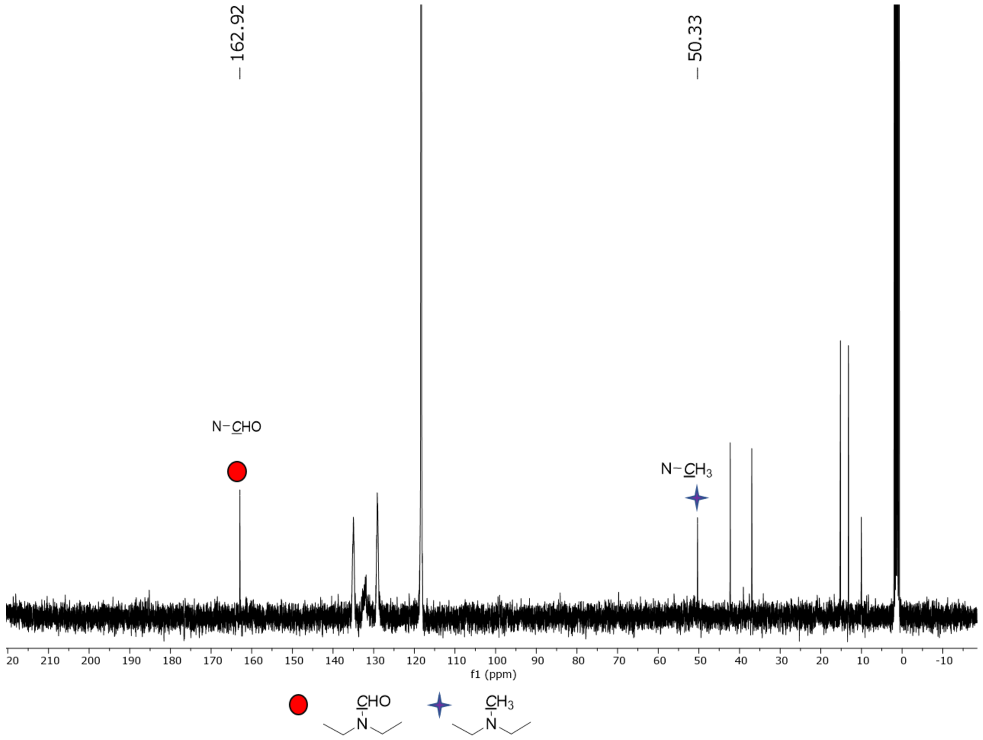

Figure S5. $^{13}C\{^1H\}$ NMR spectra of reductive functionalization of CO_2 with diethylamine and $PhSiH_3$ at 60 °C in CD_3CN.

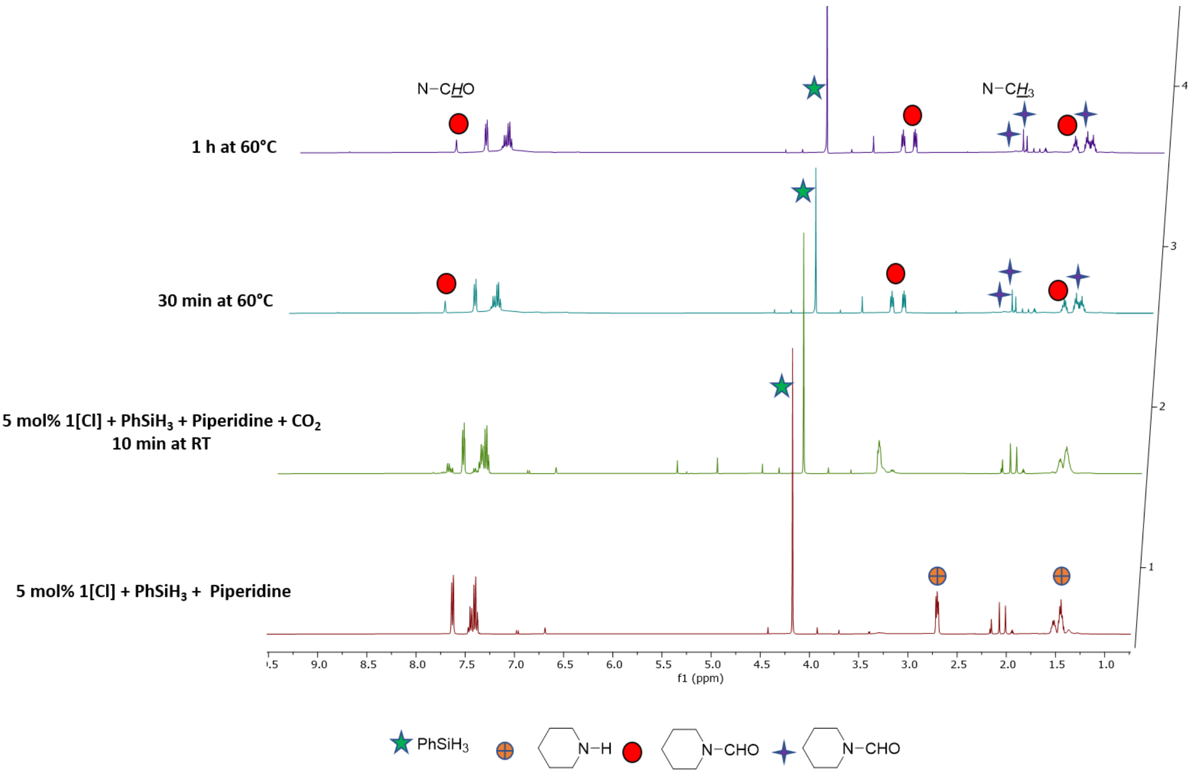

Figure S6. Stacked ^{1}H NMR spectra of reductive functionalization of CO_2 with piperidine and $PhSiH_3$ at 60 °C in CD_3CN.

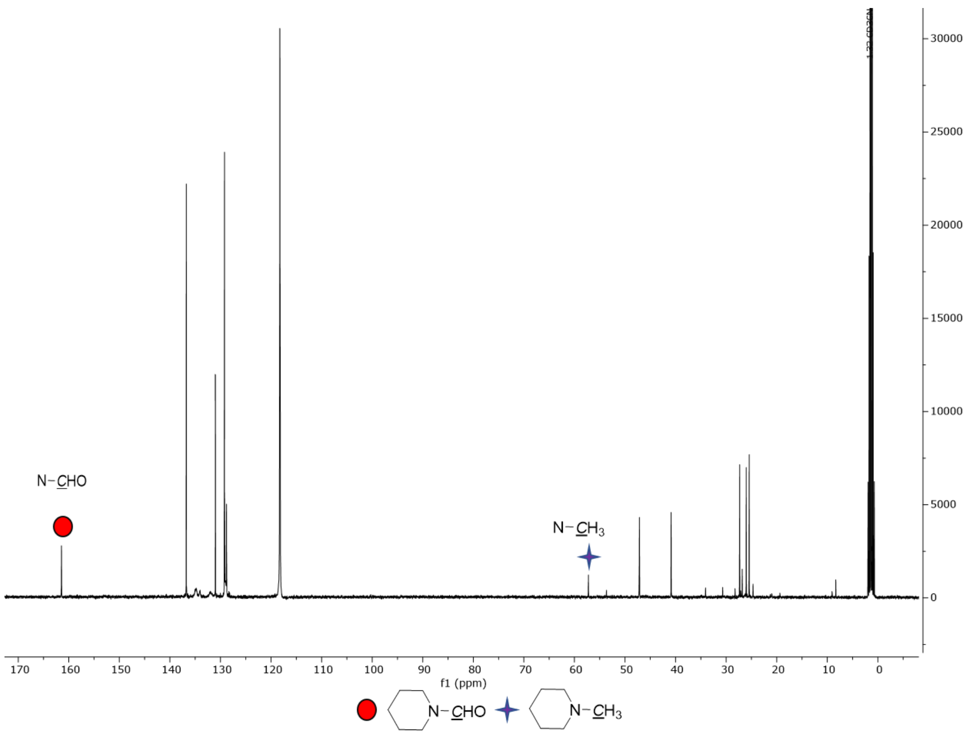

Figure S7. $^{13}C\{^1H\}$ NMR spectra of reductive functionalization of CO_2 with piperidine and $PhSiH_3$ at 60 °C in CD_3CN.

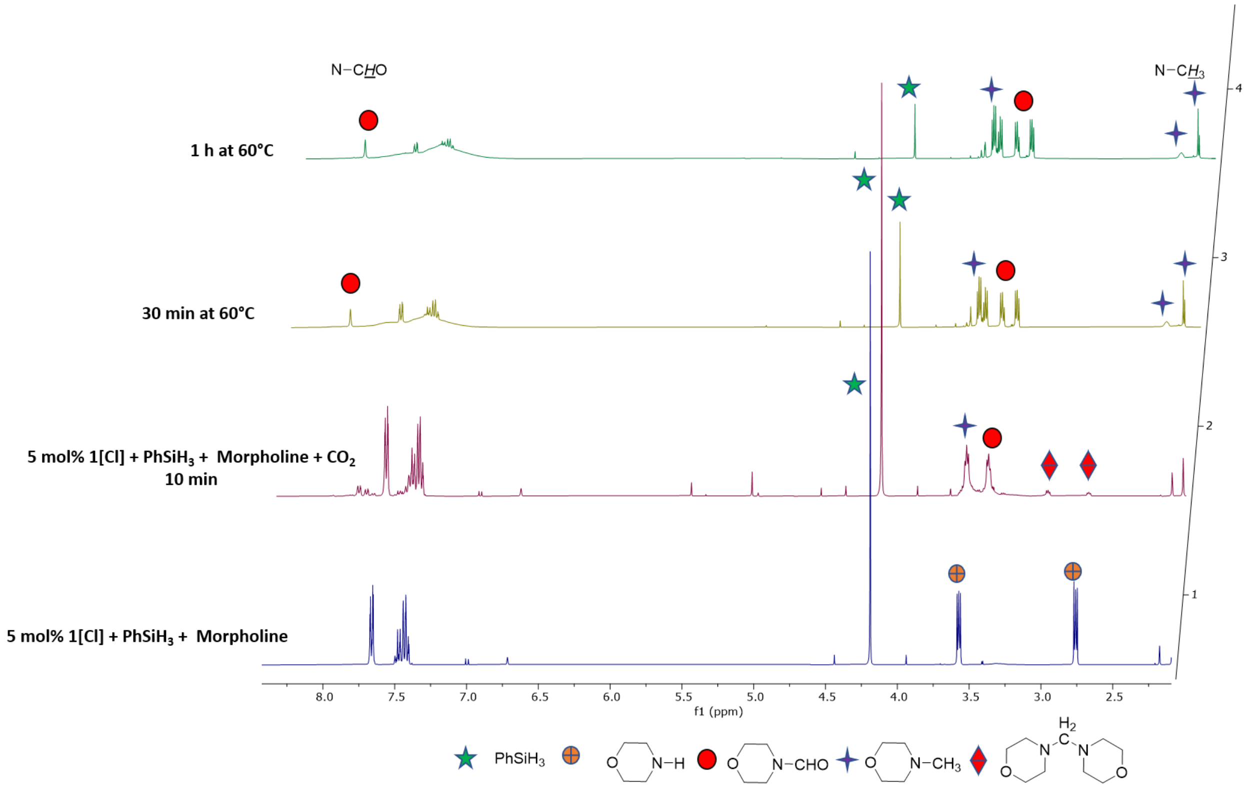

Figure S8. Stacked ^{1}H NMR spectra of reductive functionalization of CO_2 with morpholine and $PhSiH_3$ at 60 °C in CD_3CN.

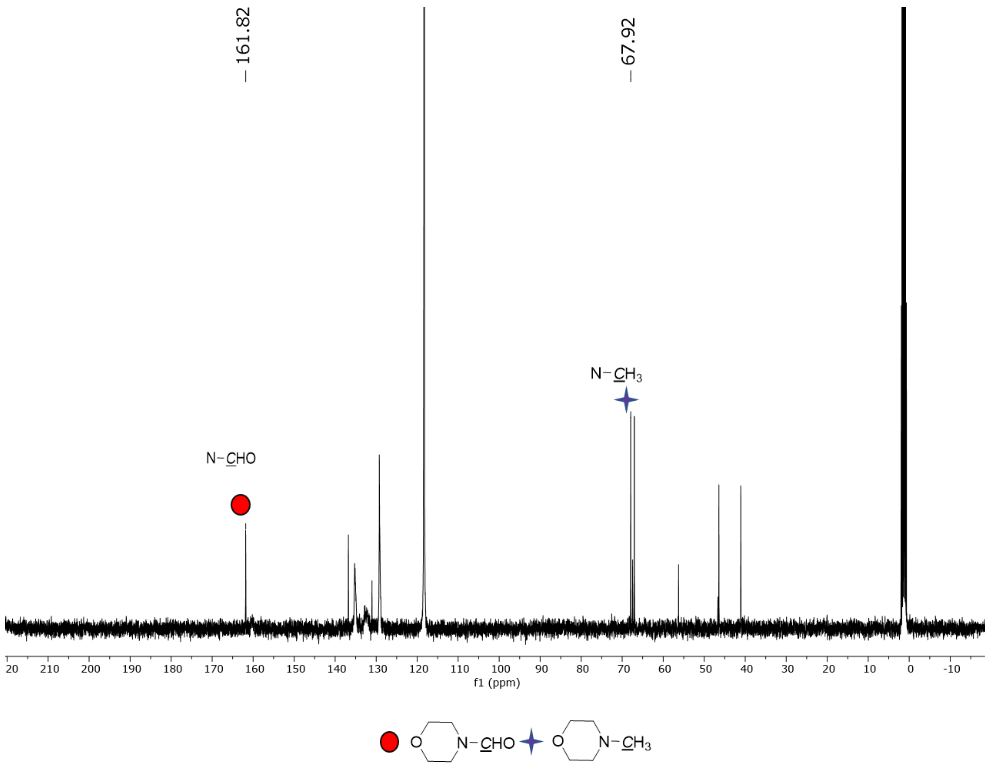

Figure S9. $^{13}C\{^{1}H\}$ NMR spectra of reductive functionalization of CO_2 with morpholine and $PhSiH_3$ at 60 °C in CD_3CN.

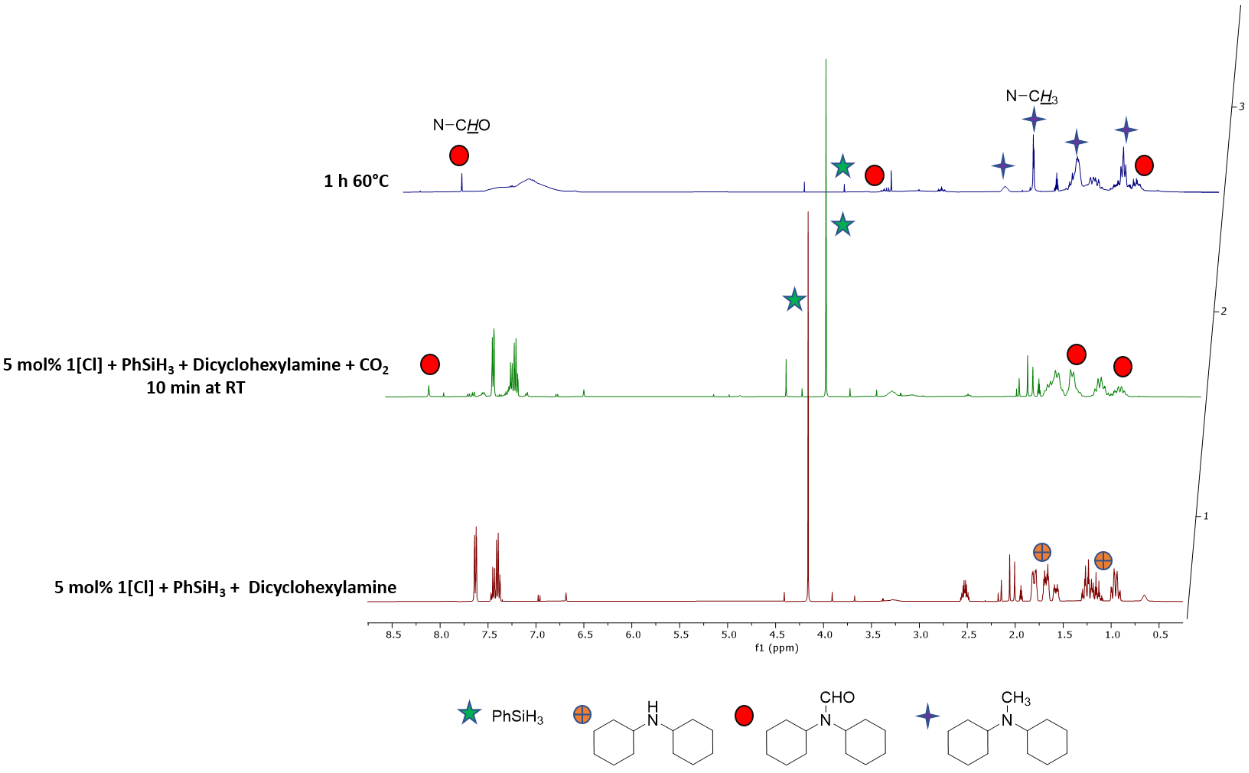

Figure S10. Stacked ^{1}H NMR spectra of reductive functionalization of CO_2 with dicyclohexylamine and $PhSiH_3$ at 60 °C in CD_3CN.

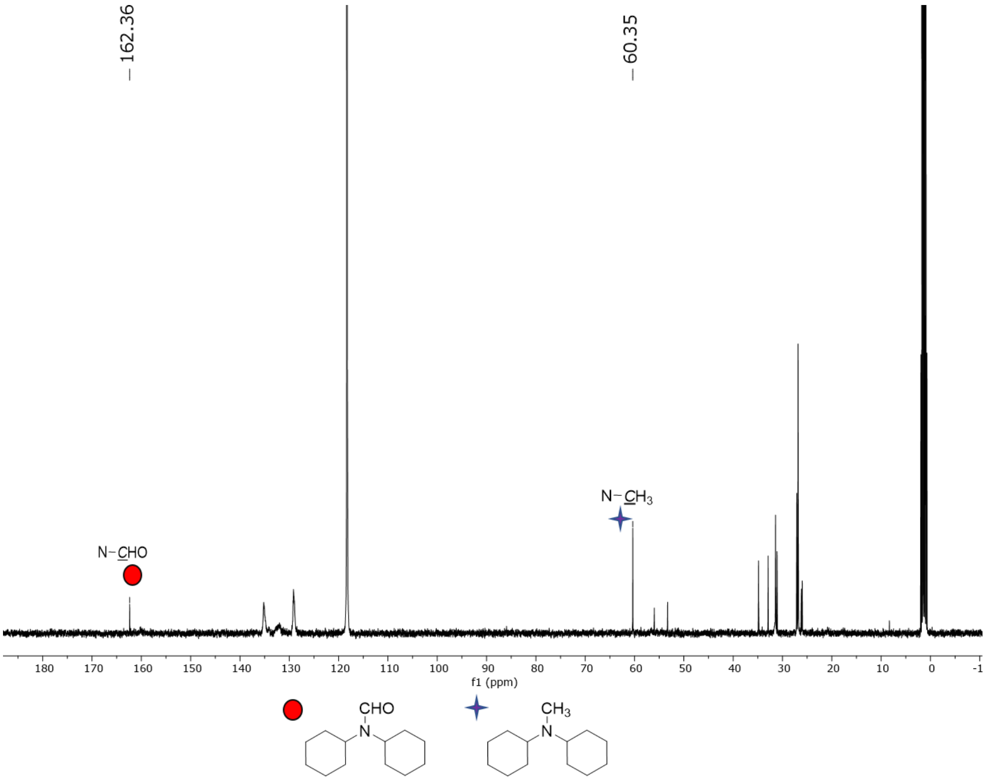

Figure S11. $^{13}C\{^1H\}$ spectra of reductive functionalization of CO_2 with dicyclohexylamine and $PhSiH_3$ at 60 °C in CD_3CN.

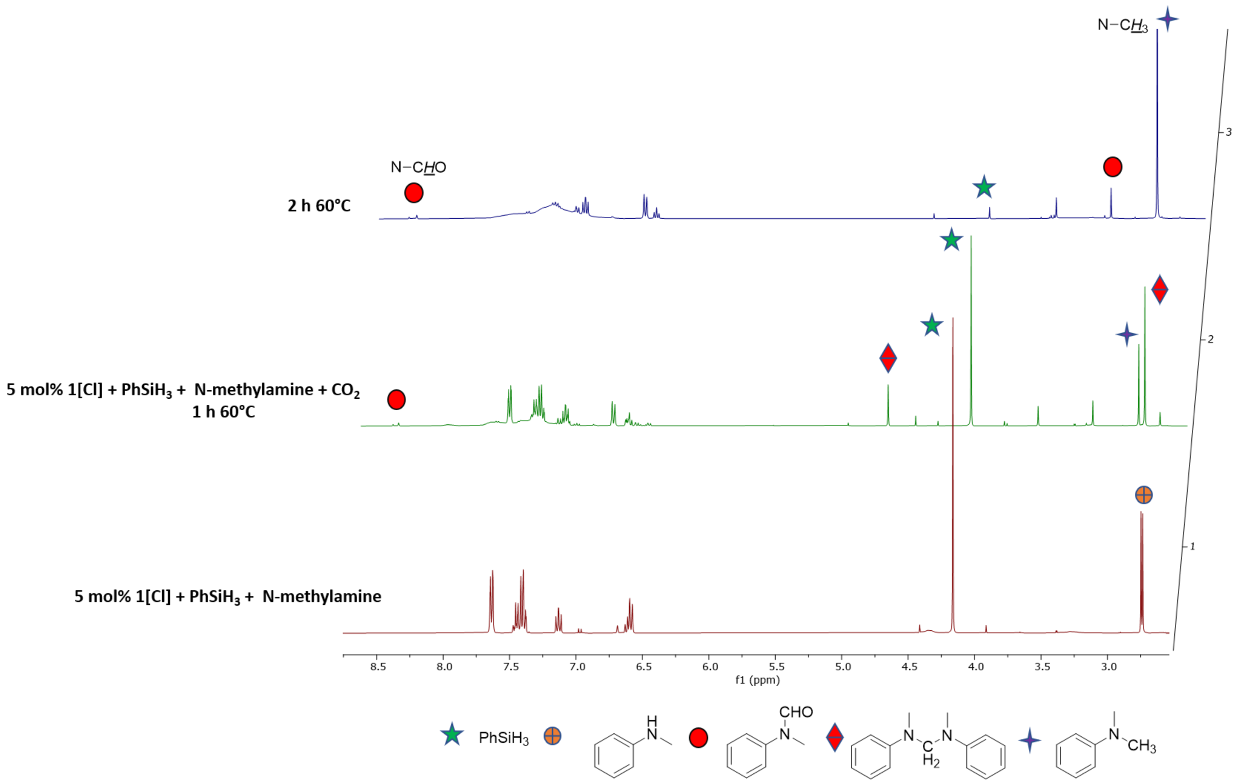

Figure S12. Stacked ^{1}H NMR spectra of reductive functionalization of CO_2 with N-methylamine and $PhSiH_3$ at 60 °C in CD_3CN.

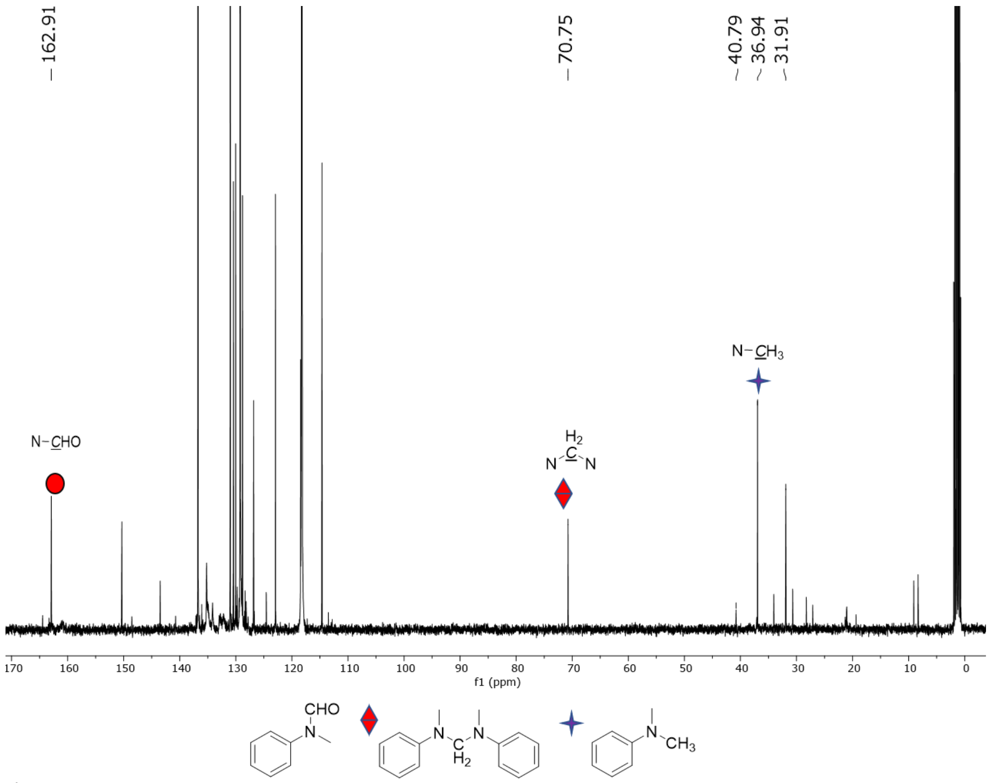

Figure S13. ^{13}C{^{1}H} NMR spectra of reductive functionalization of CO_2 with N-methylamine and $PhSiH_3$ at 60 °C in CD_3CN.

1.1.3. General procedure for catalytic cyanosilylation of carbonyls

0.1 mol% of **1** in 0.5 ml of CD_3CN, aldehyde (10 mg, 1 eq) and 1.01 equivalent of TMSCN were added to an NMR tube. The tube was sealed by wrapping parafilm around the cap. The reactions were monitored regularly using ^{1}H NMR spectroscopy. Notably for ketones 1.0 mol% of **1** and elevated temperatures (50 °C) were required.

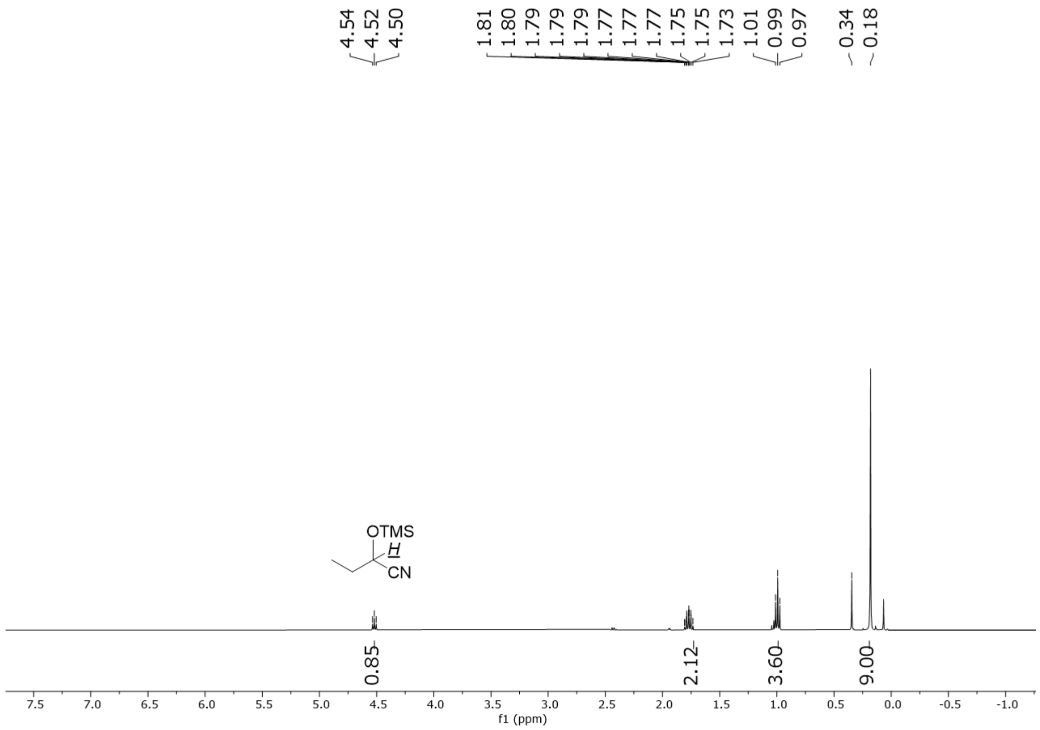

Figure S14. ^{1}H NMR spectra of $CH_3CH_2CH(OTMS)CN$ in CD_3CN.

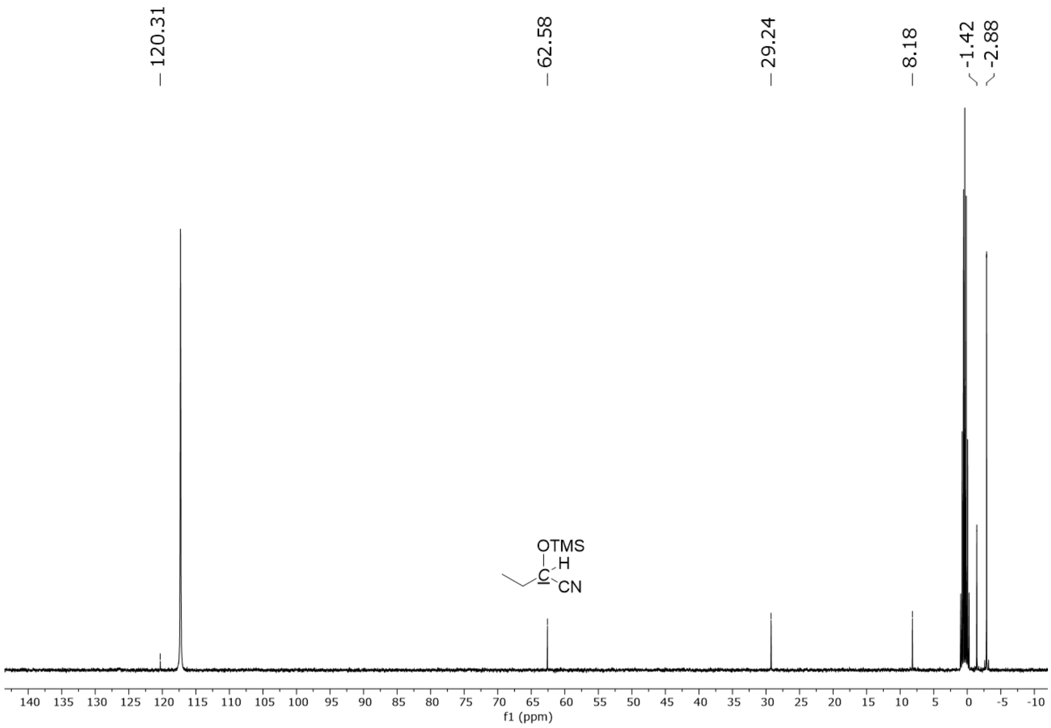

Figure S15. $^{13}C\{^{1}H\}$ NMR spectra of $CH_3CH_2CH(OTMS)CN$ in CD_3CN.

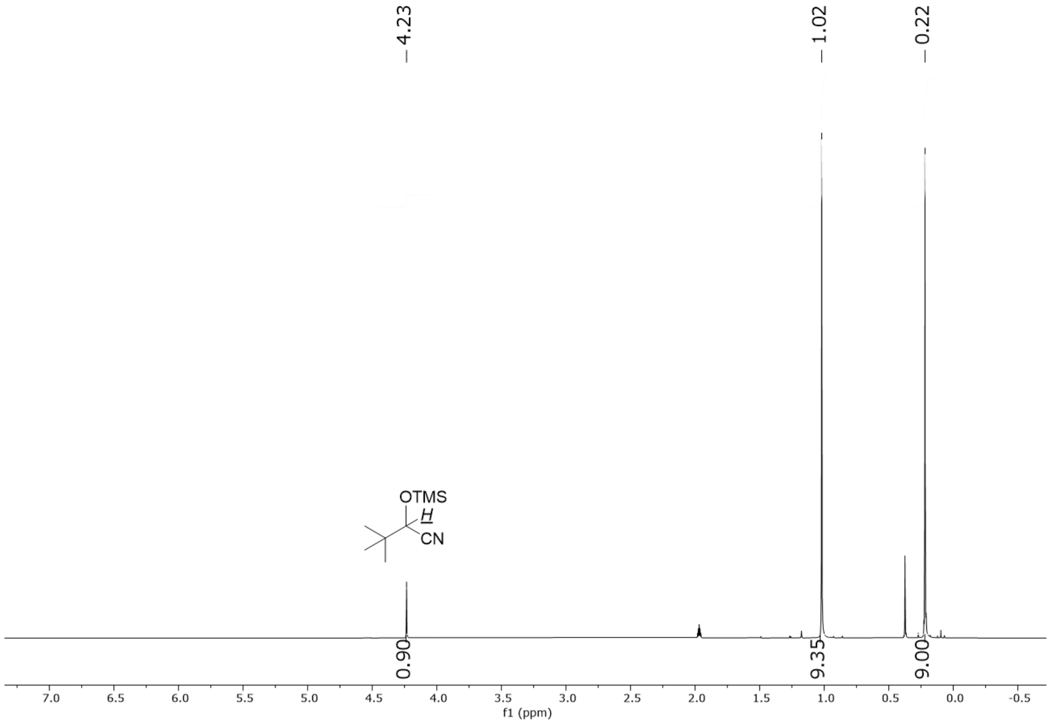

Figure S16. ^{1}H NMR spectra of $(CH_3)_3CH(OTMS)CN$ in CD_3CN.

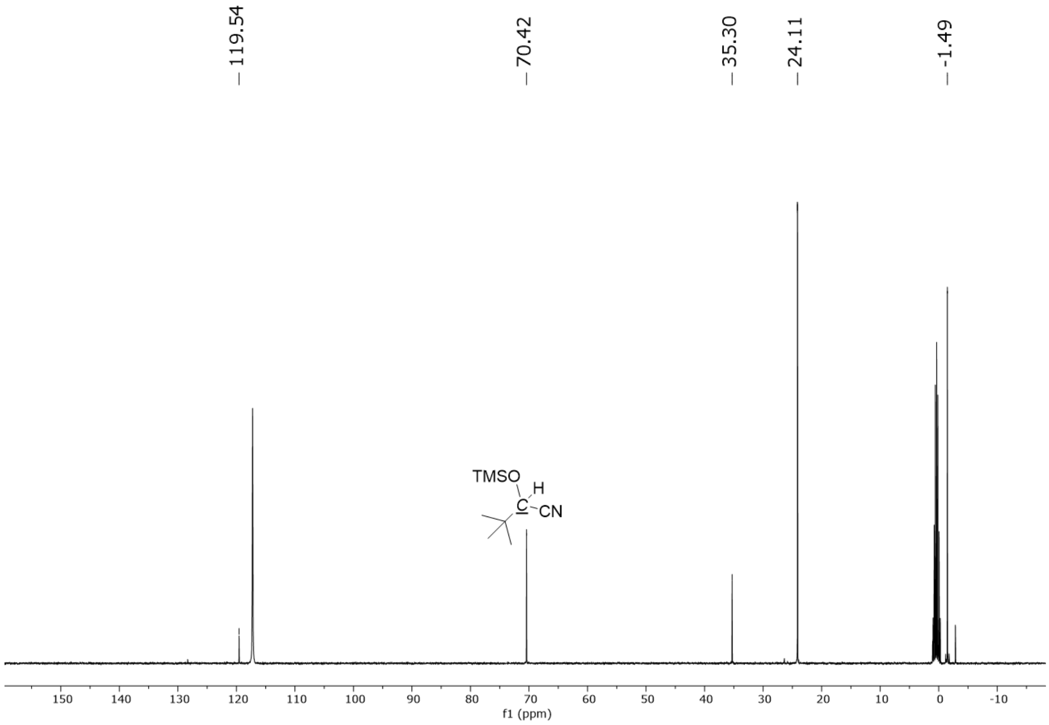

Figure S17. $^{13}C\{^{1}H\}$ NMR spectra of $(CH_3)_3CH(OTMS)CN$ in CD_3CN.

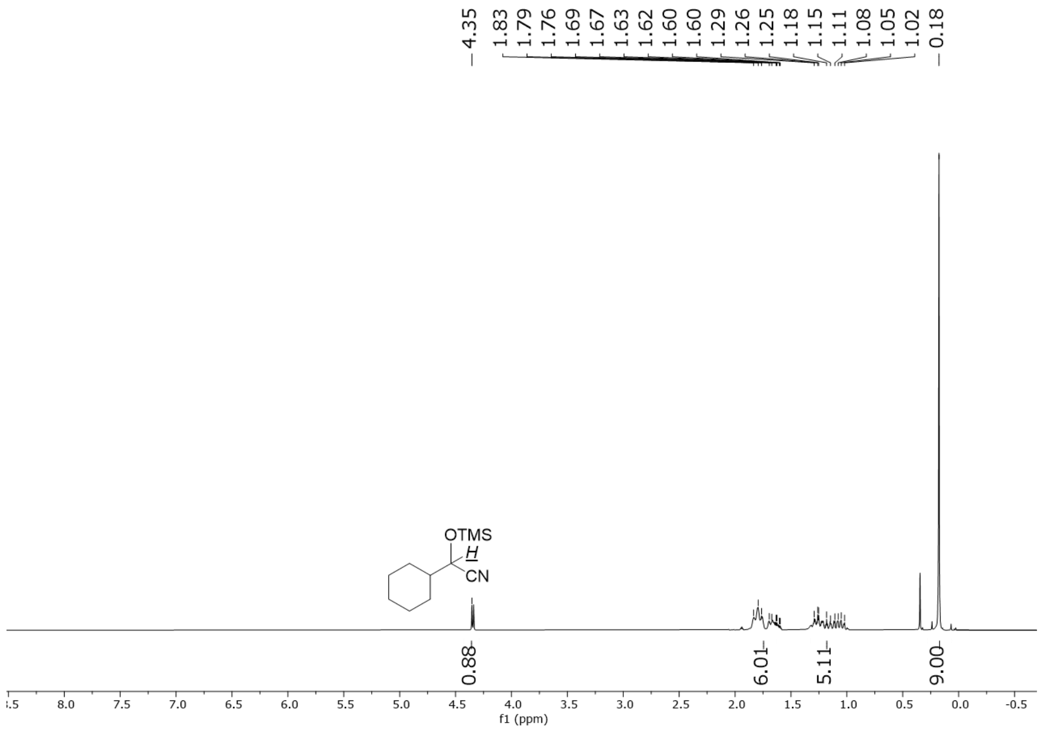

Figure S18. ^{1}H NMR spectra of $C_6H_{11}CH(OTMS)CN$ in CD_3CN.

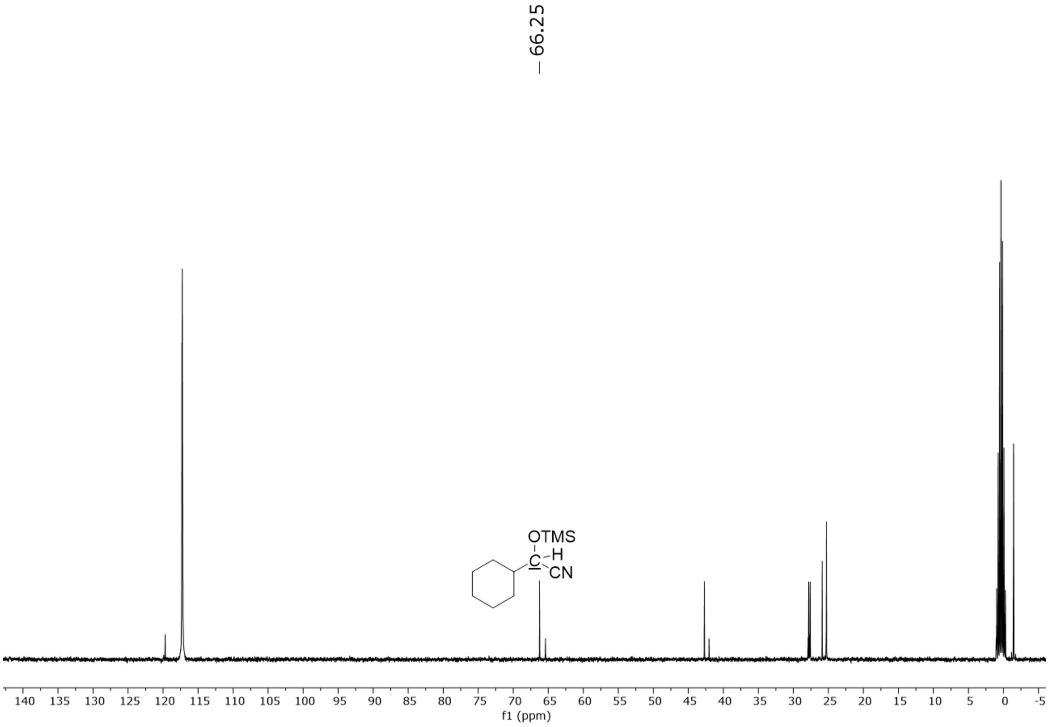

Figure S19. $^{13}C\{^{1}H\}$ NMR spectra of $C_6H_{11}CH(OTMS)CN$ in CD_3CN.

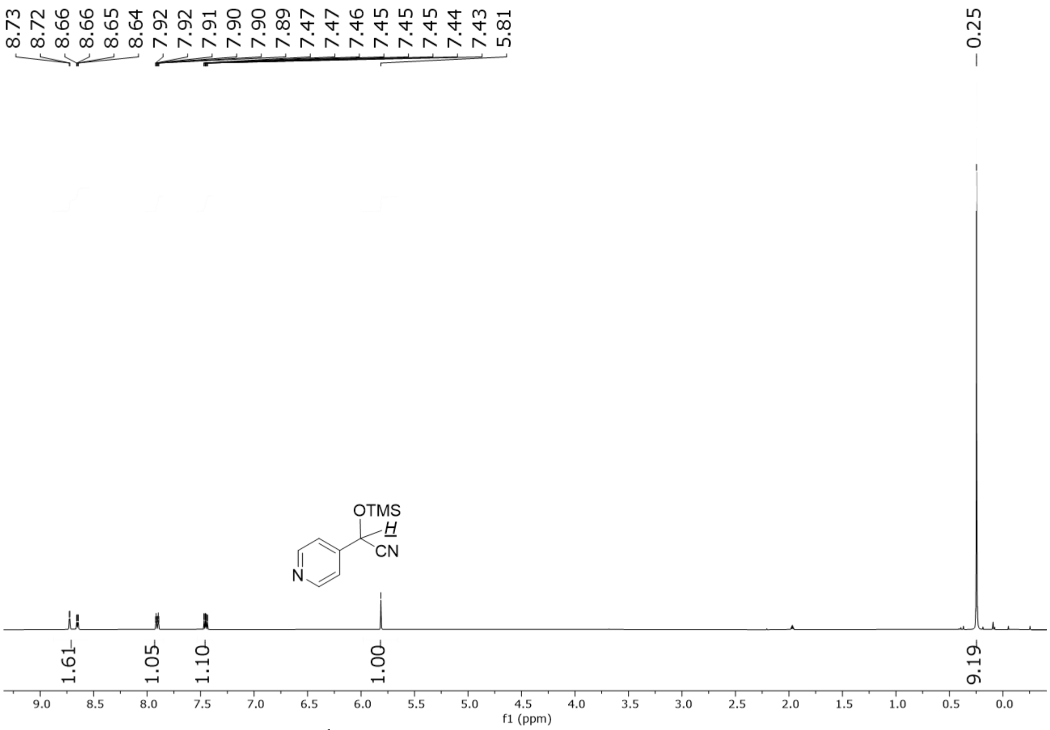

Figure S20. ^{1}H NMR spectra of $(NC_5H_4)CH(OTMS)CN$ in CD_3CN.

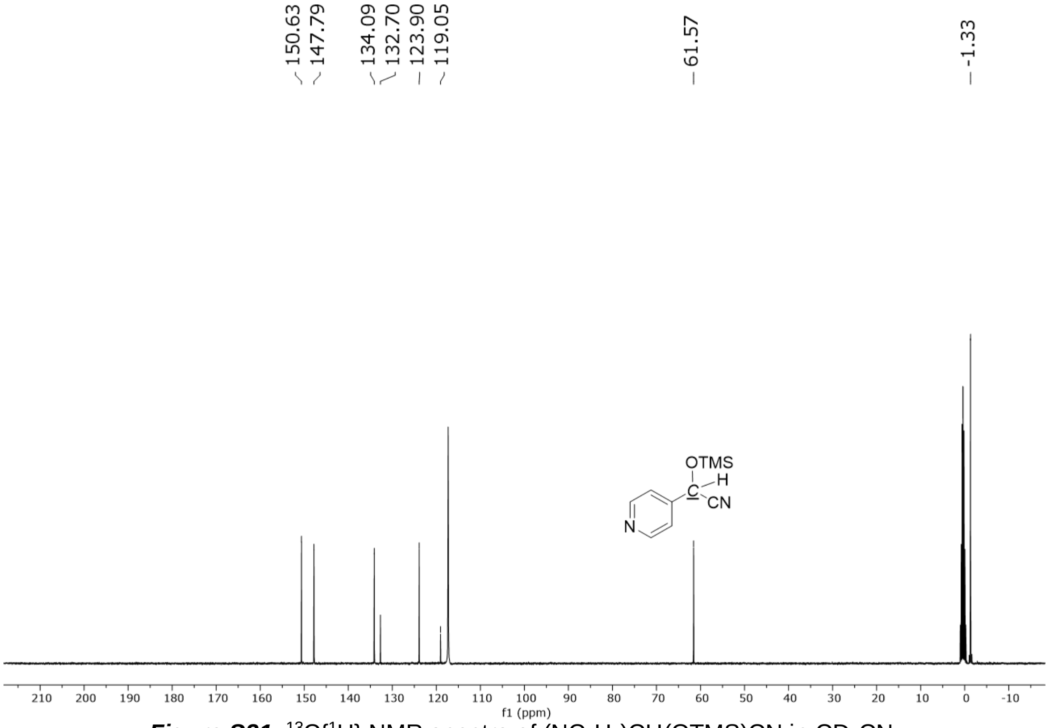

Figure S21. $^{13}C\{^1H\}$ NMR spectra of $(NC_5H_4)CH(OTMS)CN$ in CD_3CN.

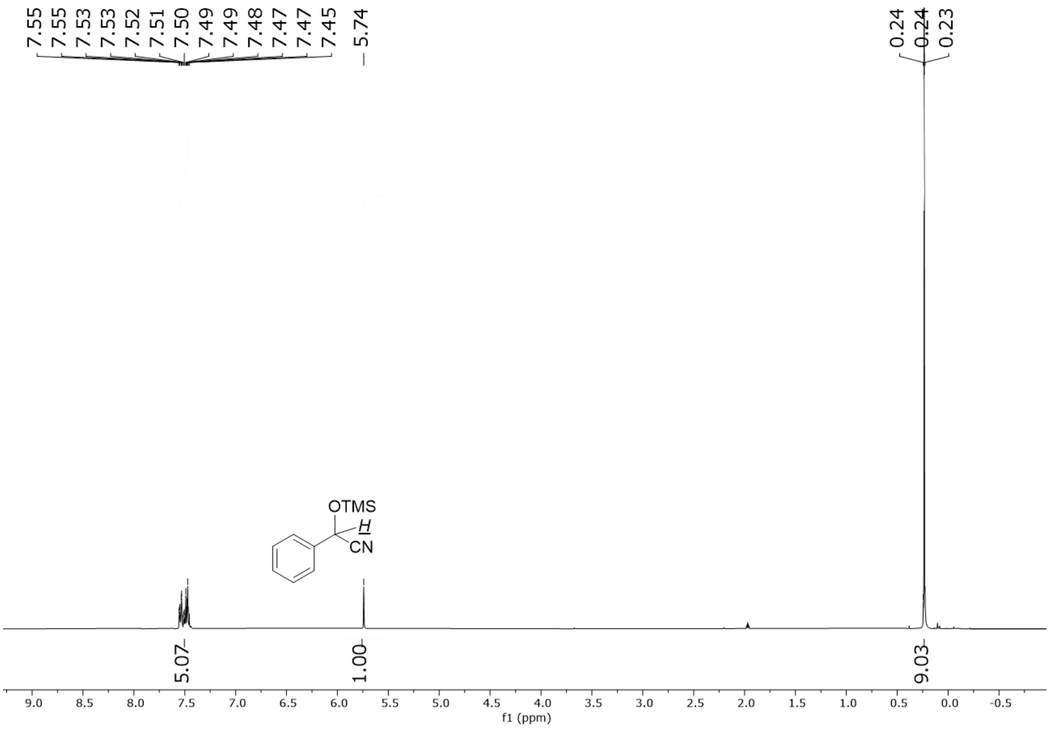

Figure S22. ^{1}H NMR spectra of PhCH(OTMS)CN in CD_3CN.

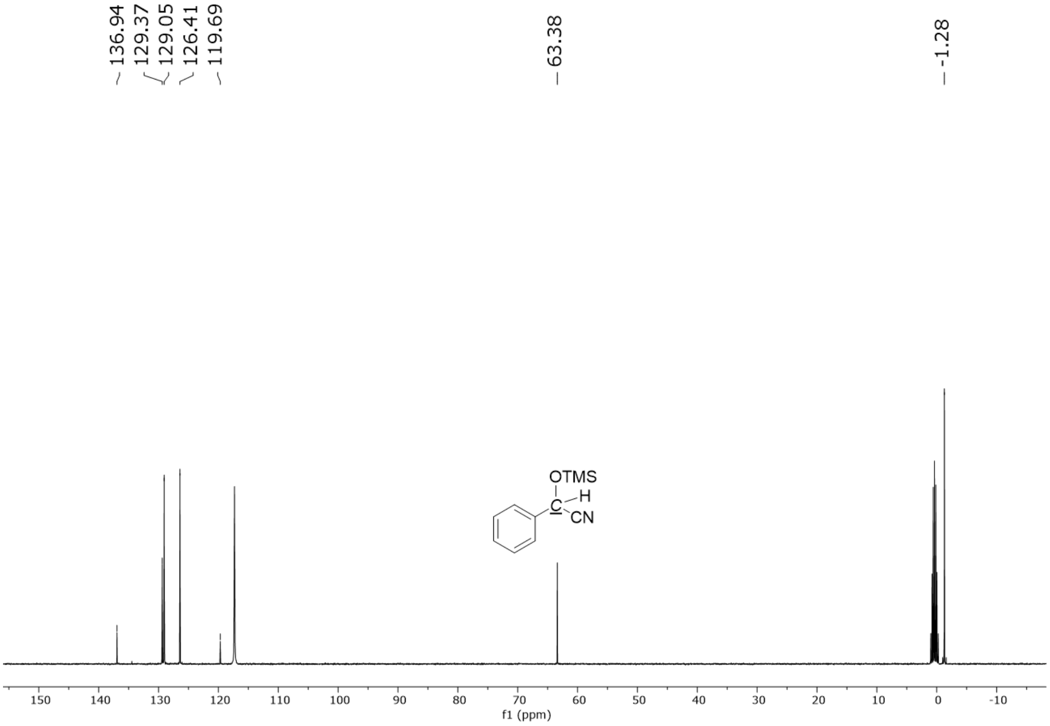

Figure S23. ^{13}C{^{1}H} NMR spectra of PhCH(OTMS)CN in CD_3CN.

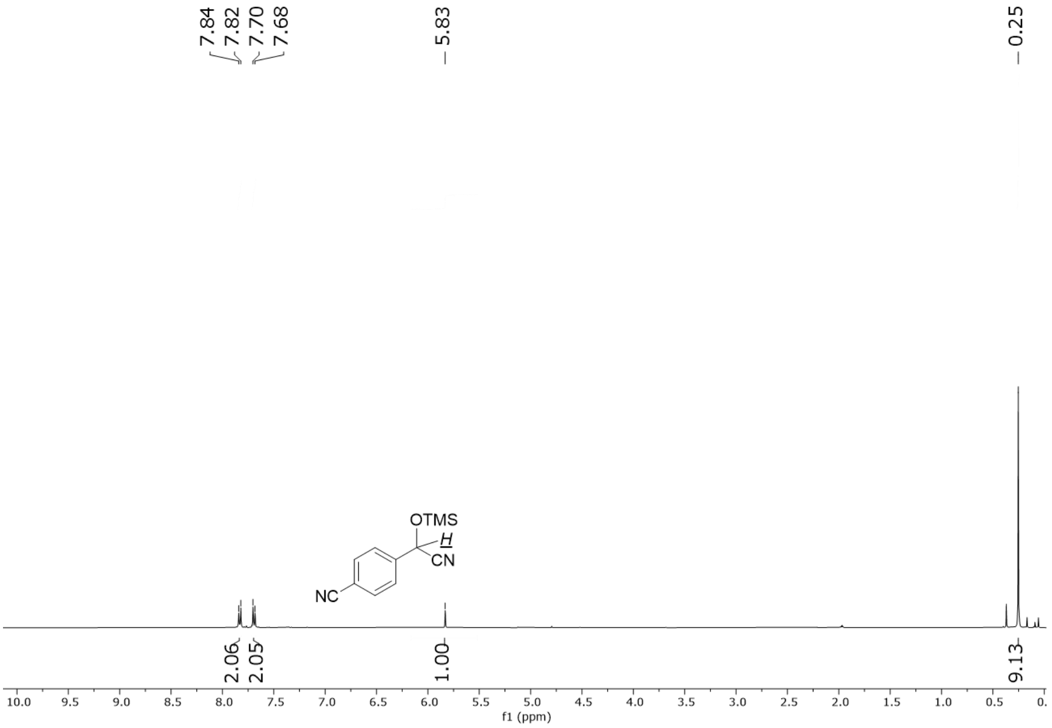

Figure S24. ^{1}H NMR spectra of (CN)PhCH(OTMS)CN in CD_3CN.

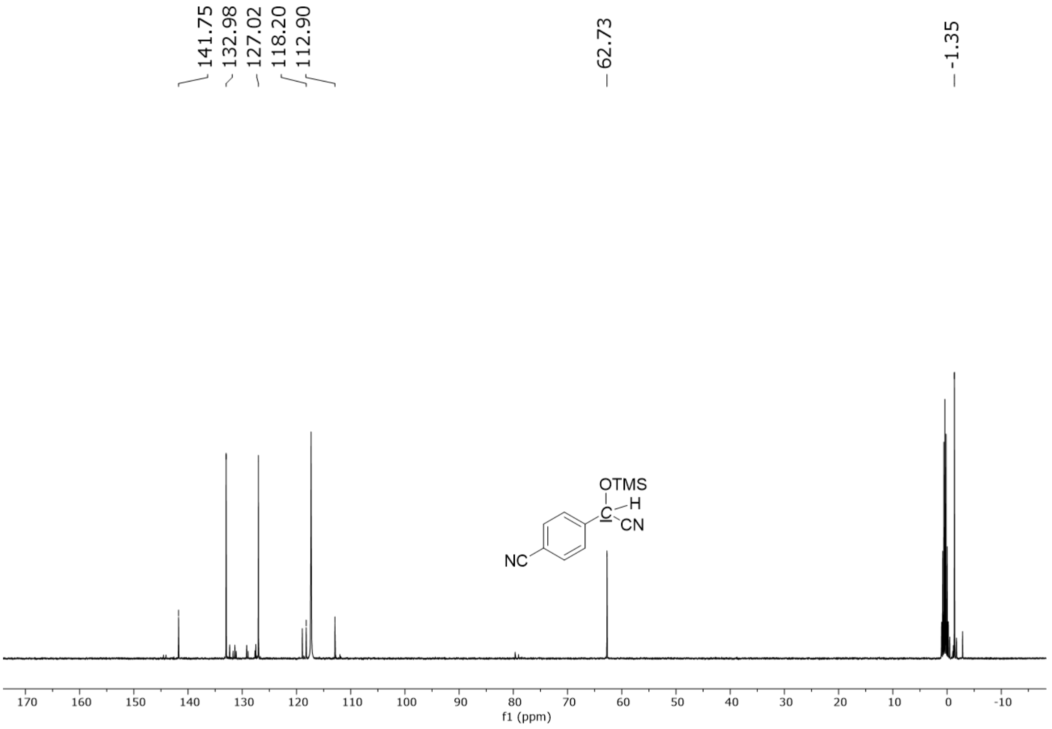

Figure S25. $^{13}C\{^{1}H\}$ NMR spectra of (CN)PhCH(OTMS)CN in CD_3CN.

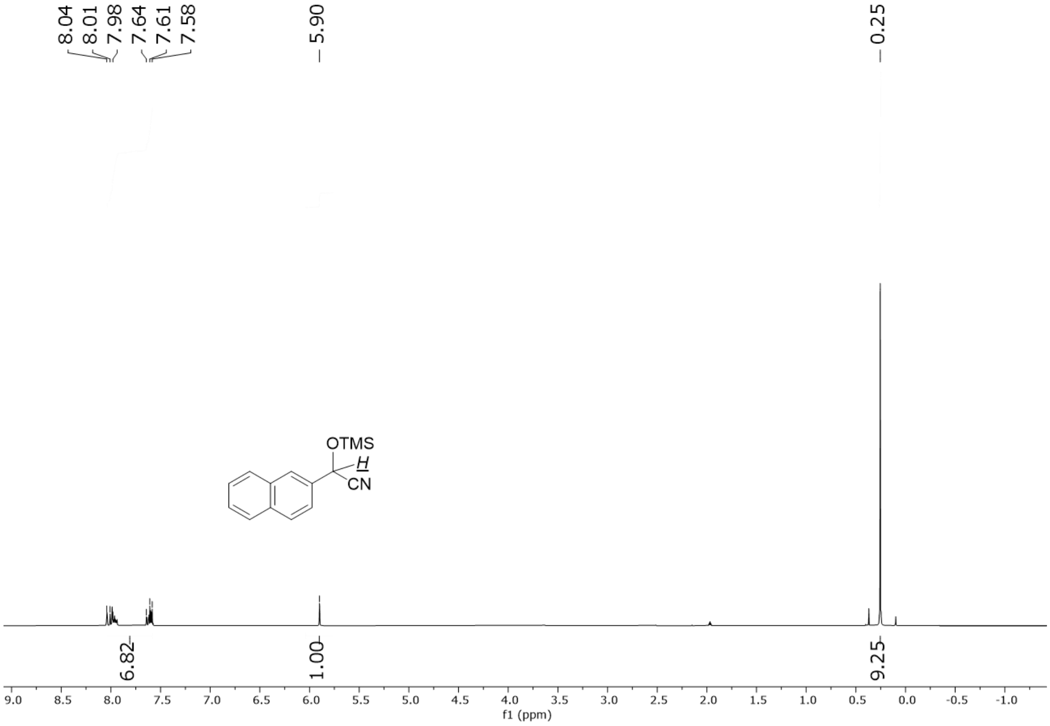

Figure S26. ^{1}H NMR spectra of $(C_{10}H_7)CH(OTMS)CN$ in CD_3CN.

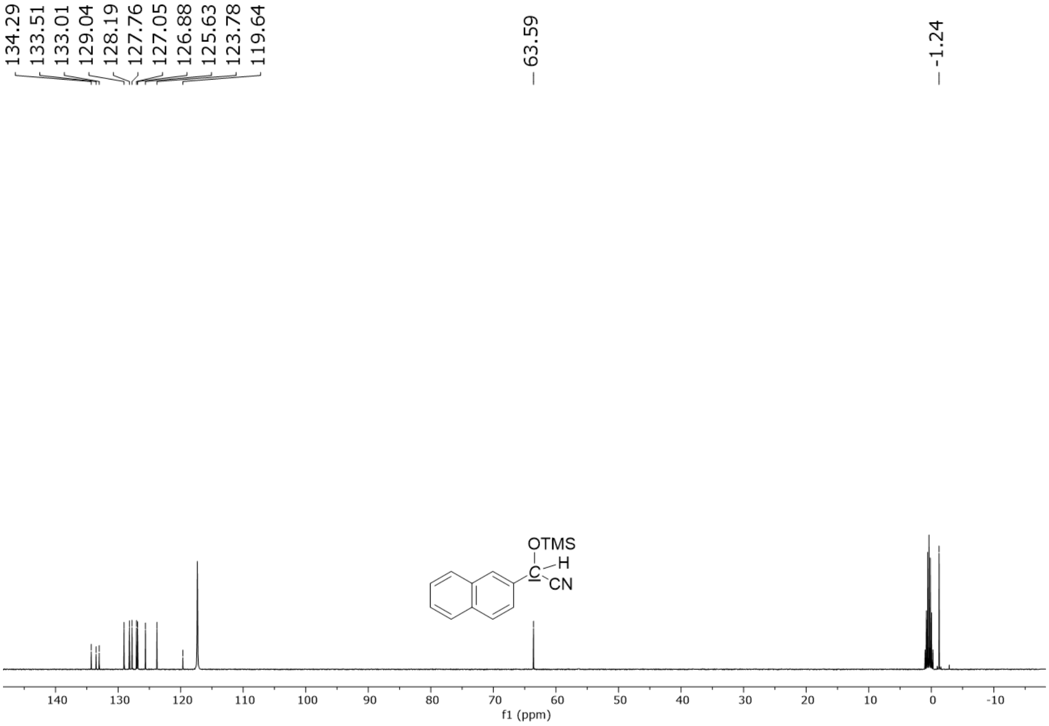

Figure S27. $^{13}C\{^{1}H\}$ NMR spectra of $(C_{10}H_7)CH(OTMS)CN$ in CD_3CN

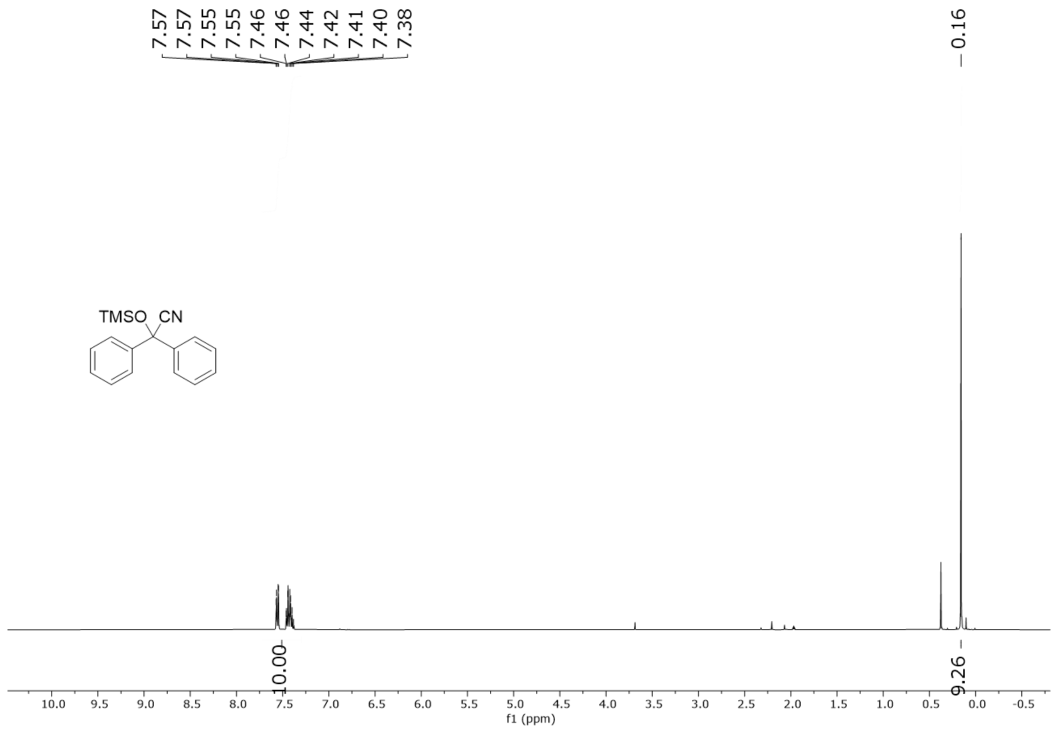

Figure S28. ^{1}H NMR spectra of $Ph_2C(OTMS)CN$ in CD_3CN.

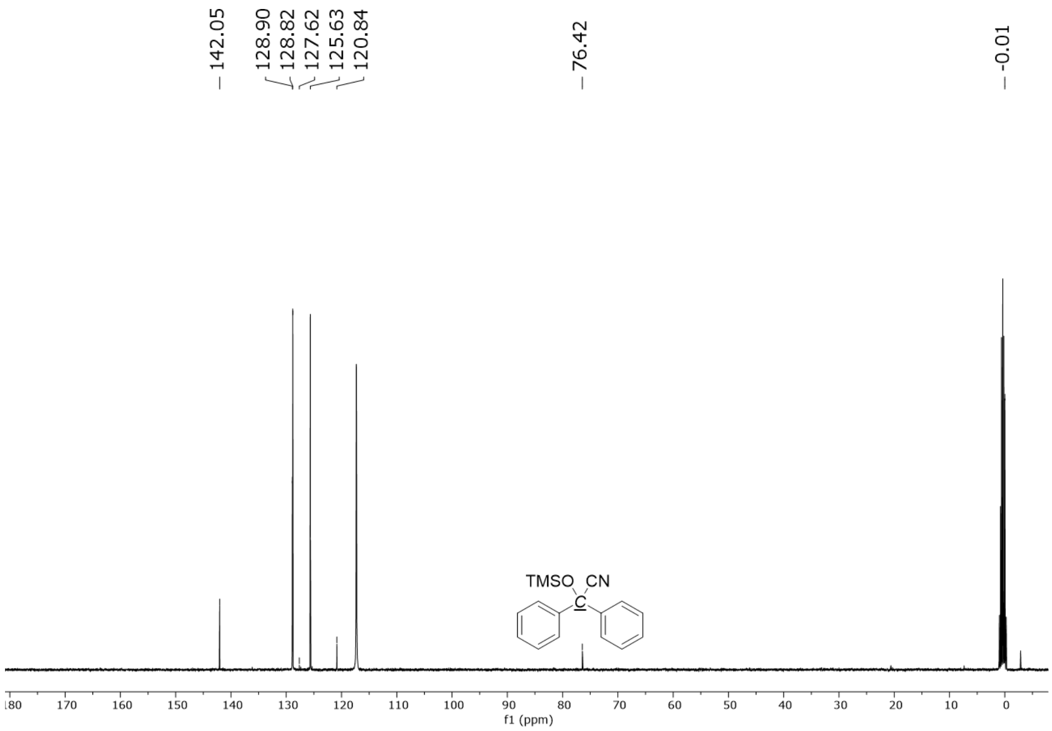

Figure S29. $^{13}C\{^{1}H\}$ NMR spectra of $Ph_2C(OTMS)CN$ in CD_3CN.

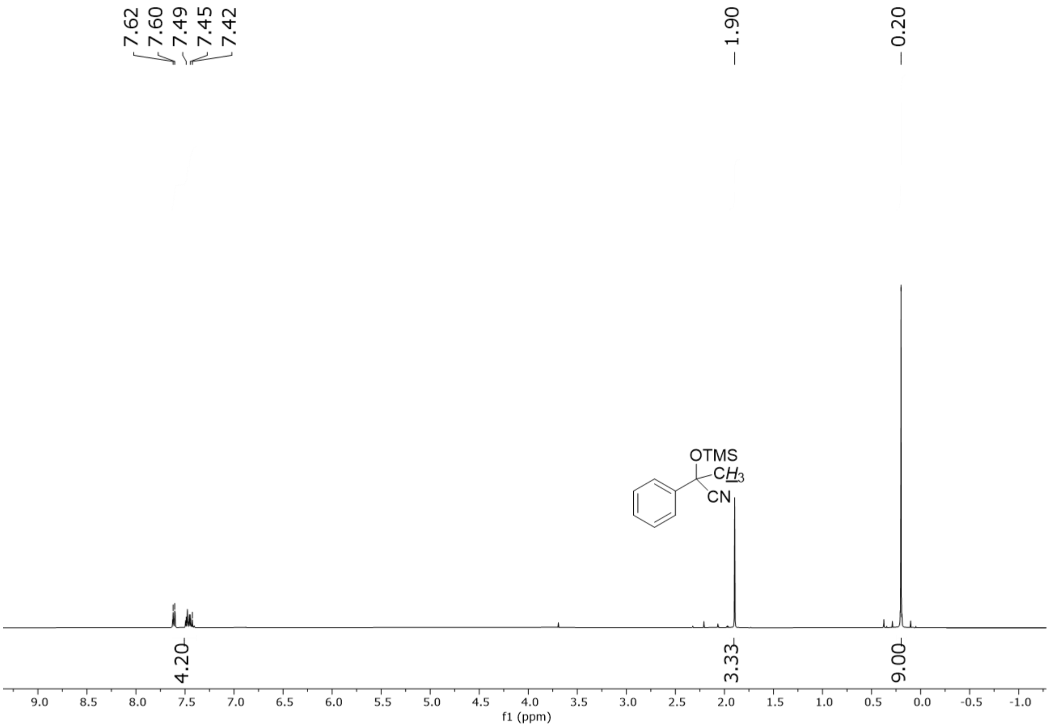

Figure S30. ^{1}H NMR spectra of $Ph(CH_3)C(OTMS)CN$ in CD_3CN.

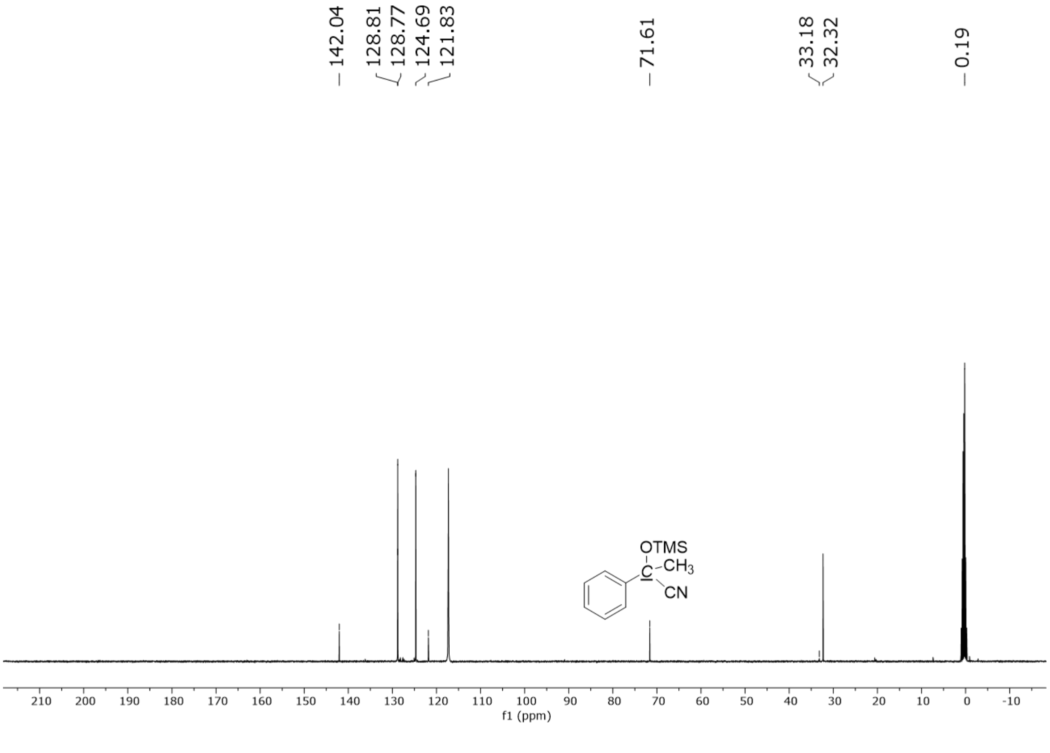

Figure S31. $^{13}C\{^{1}H\}$ NMR spectra of $Ph(CH_3)C(OTMS)CN$ in CD_3CN.

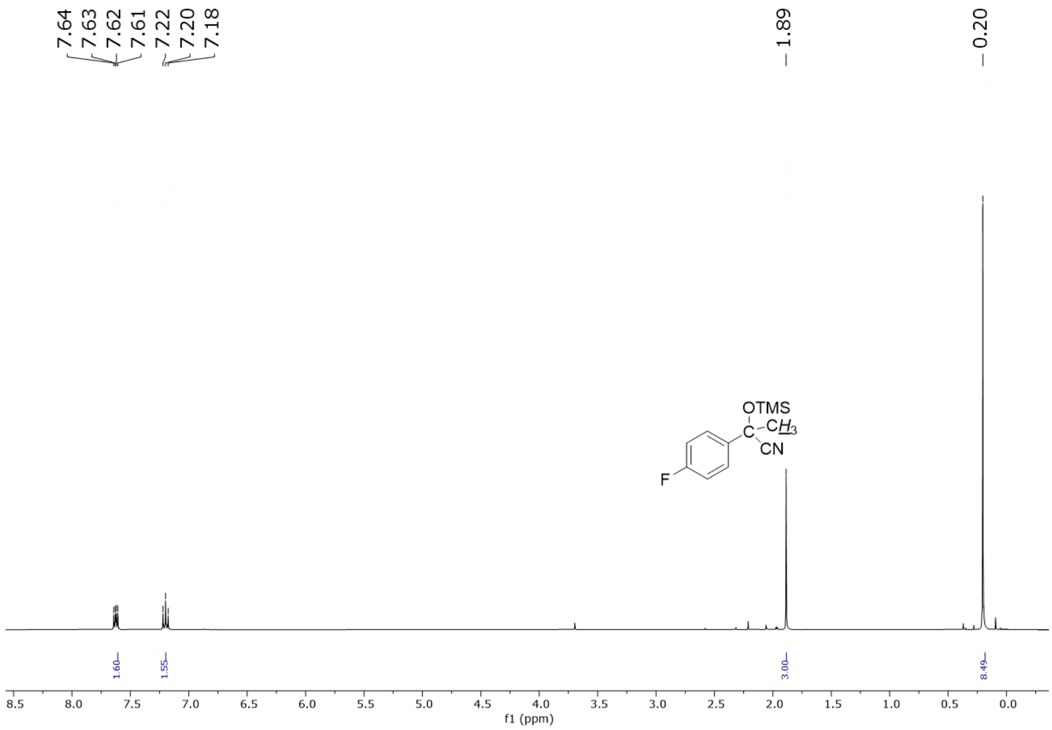

Figure S32. ^{1}H NMR spectra of (F)PhCH(OTMS)CN in CD_3CN.

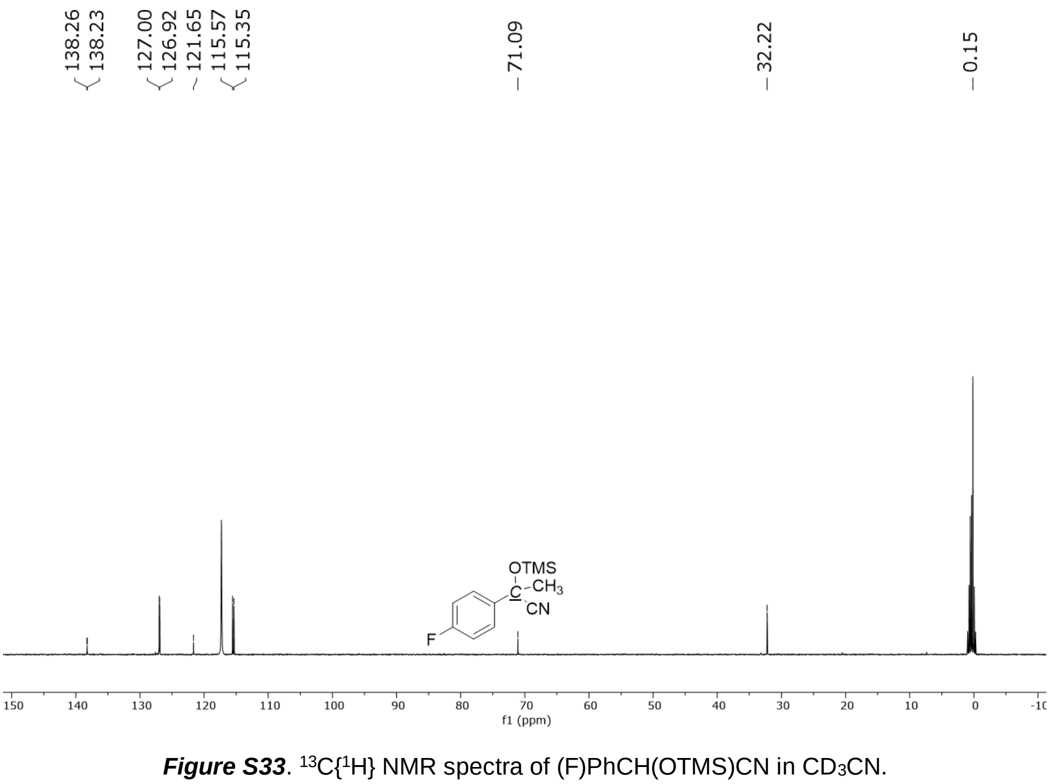

Figure S33. ^{13}C{^{1}H} NMR spectra of (F)PhCH(OTMS)CN in CD_3CN.

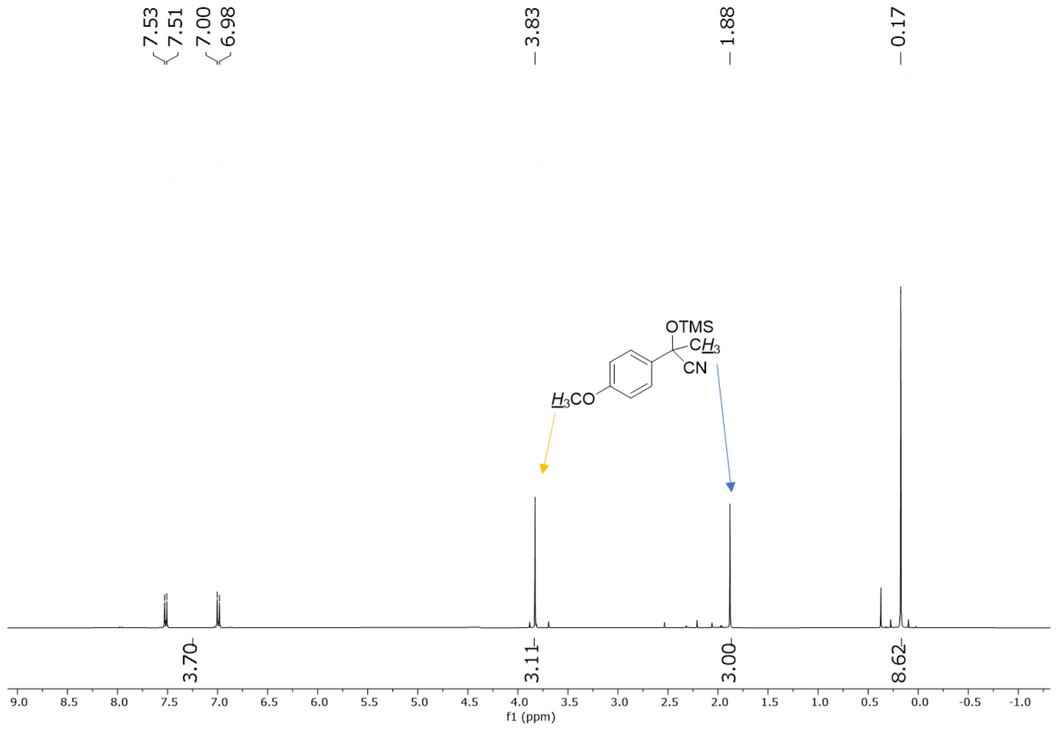

Figure S34. ¹H NMR spectra of MeOPh(CH_3)C(OTMS)CN in CD_3CN.

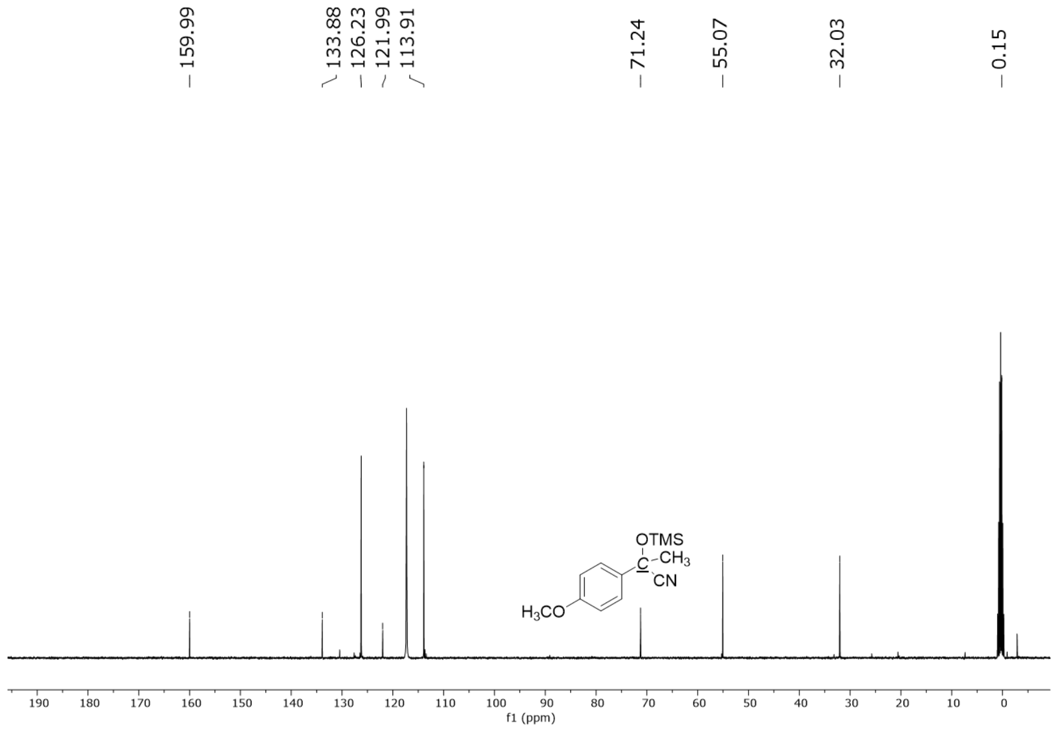

Figure S35. $^{13}C\{^1H\}$ NMR spectra of MeOPh(CH_3)C(OTMS)CN in CD_3CN.

1.1.4. General procedure for catalytic hydroboration of carbonyls

0.01 mol% **1** in 0.5 ml of CD_3CN, aldehyde (10 mg, 1 eq) and 1.1 equivalent of HBpin were added to an NMR tube. The tube was sealed by wrapping parafilm around the cap. The reactions were monitored regularly using ^1H NMR spectroscopy.

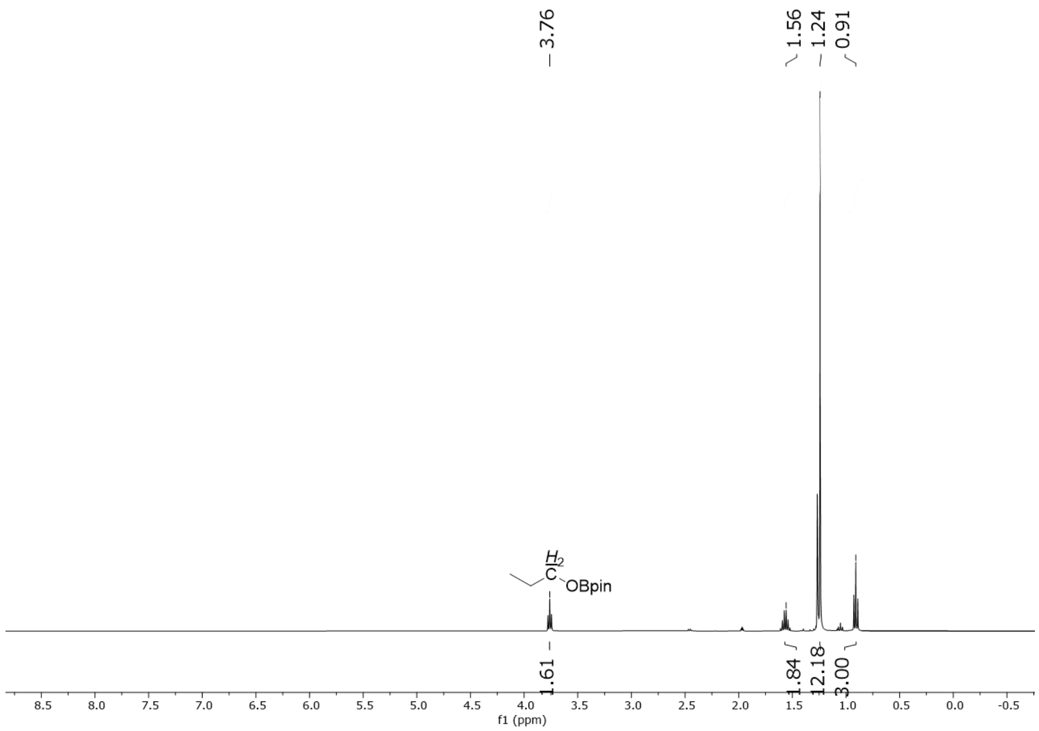

Figure S36. ^{1}H NMR spectra of Et(CH$_2$)OBpin in CD$_3$CN.

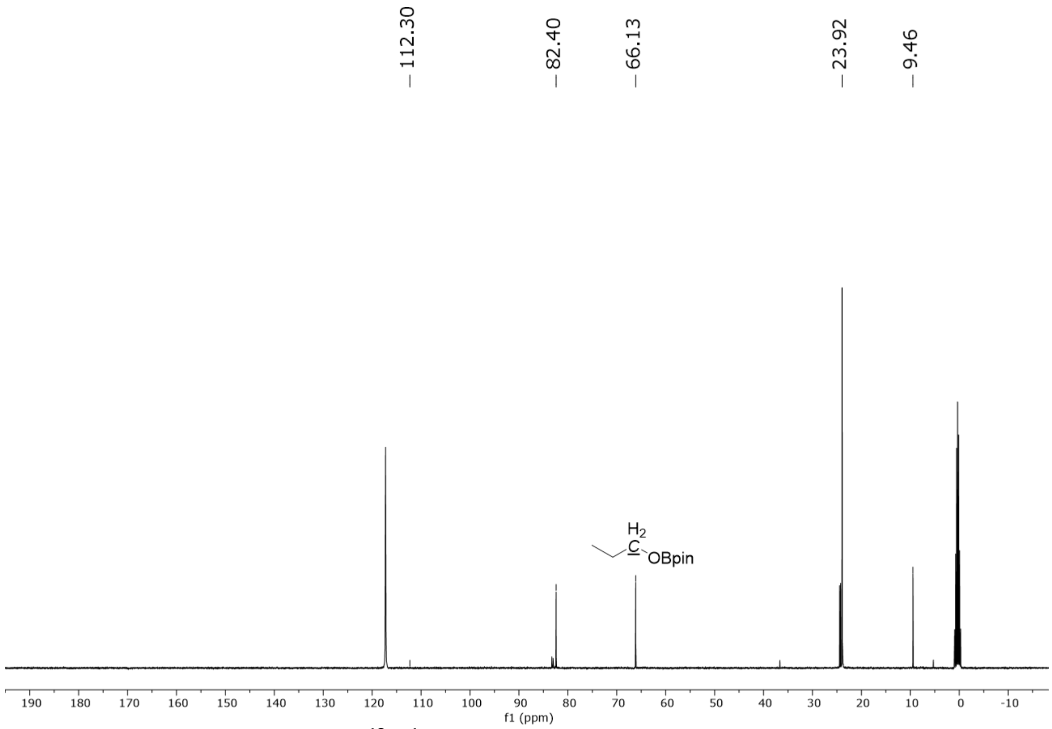

Figure S37. $^{13}C\{^{1}H\}$ NMR spectra of Et(CH$_2$)OBpin in CD$_3$CN.

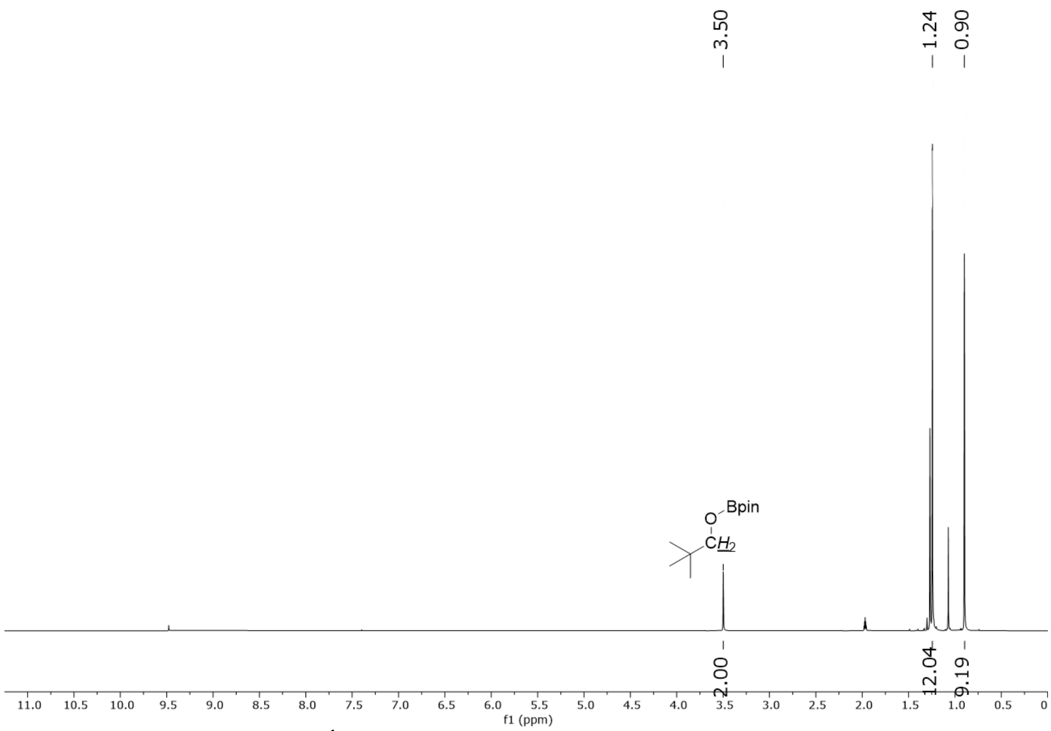

Figure S38. ^{1}H NMR spectra of $(CH_3)_3CH_2OBpin$ in CD_3CN.

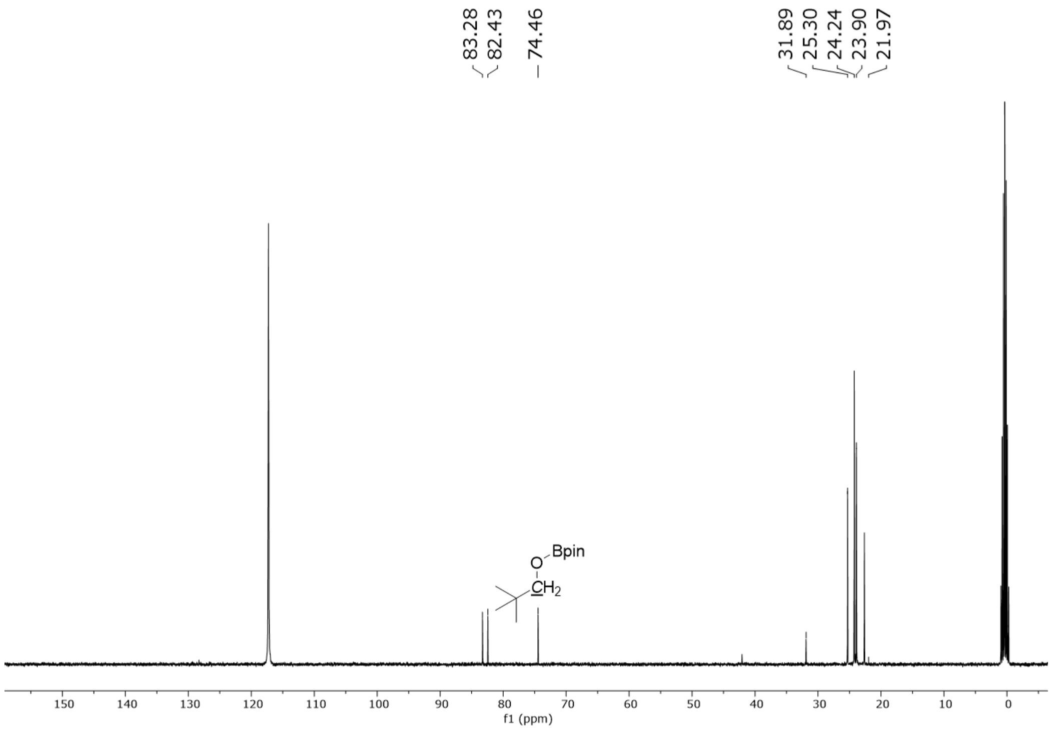

Figure S39. $^{13}C\{^{1}H\}$ NMR spectra of $(CH_3)_3CH_2OBpin$ in CD_3CN.

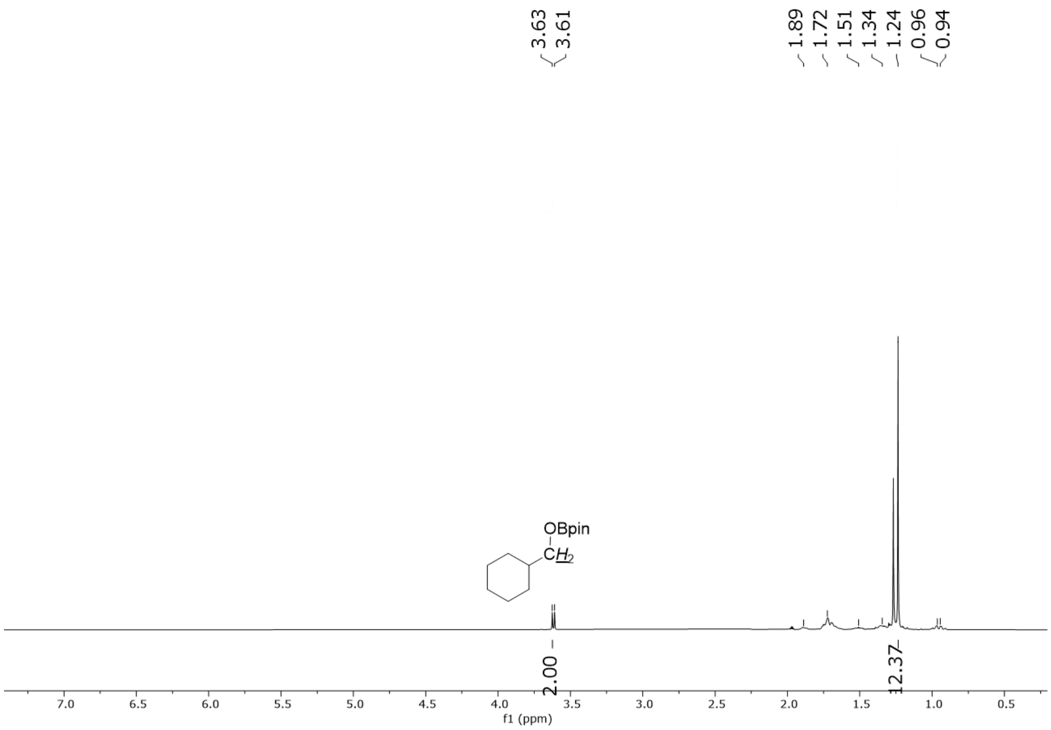

Figure S40. ^{1}H NMR spectra of $(C_6H_{11})CH_2(OBpin)$ in CD_3CN.

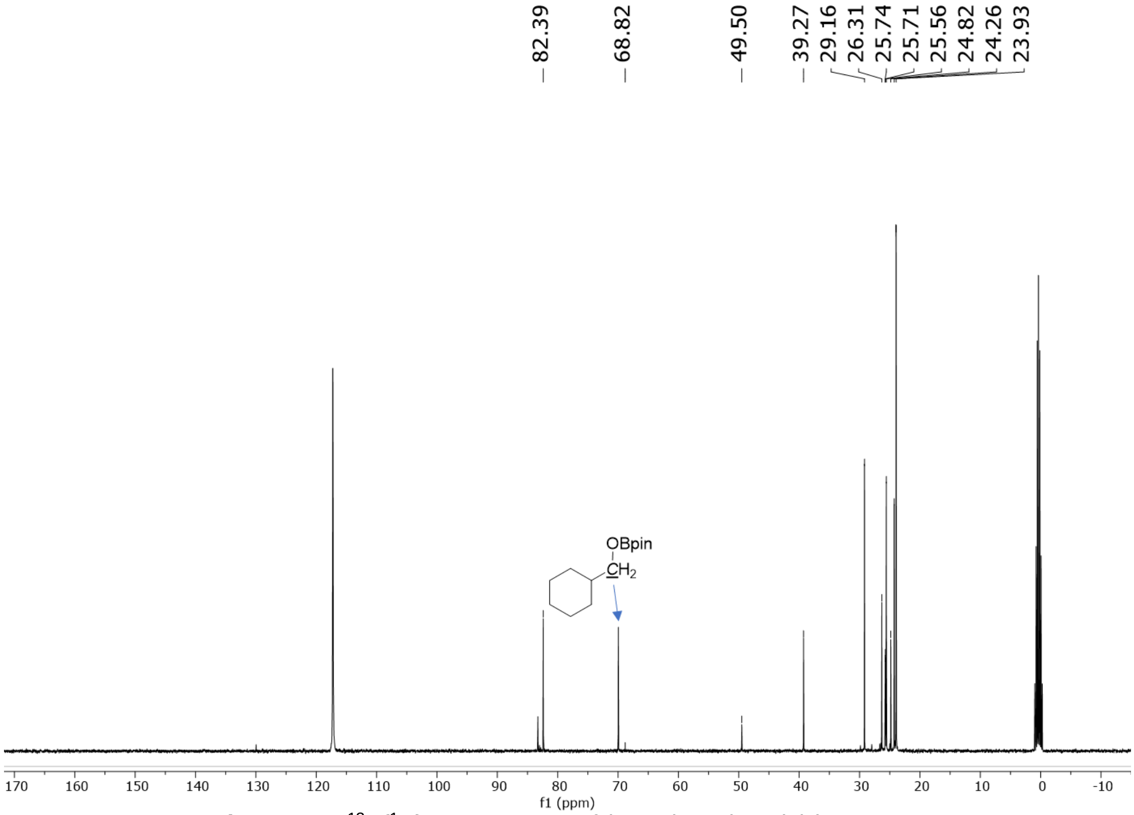

Figure S41. $^{13}C\{^{1}H\}$ NMR spectra of $(C_6H_{11})CH_2(OBpin)$ in CD_3CN.

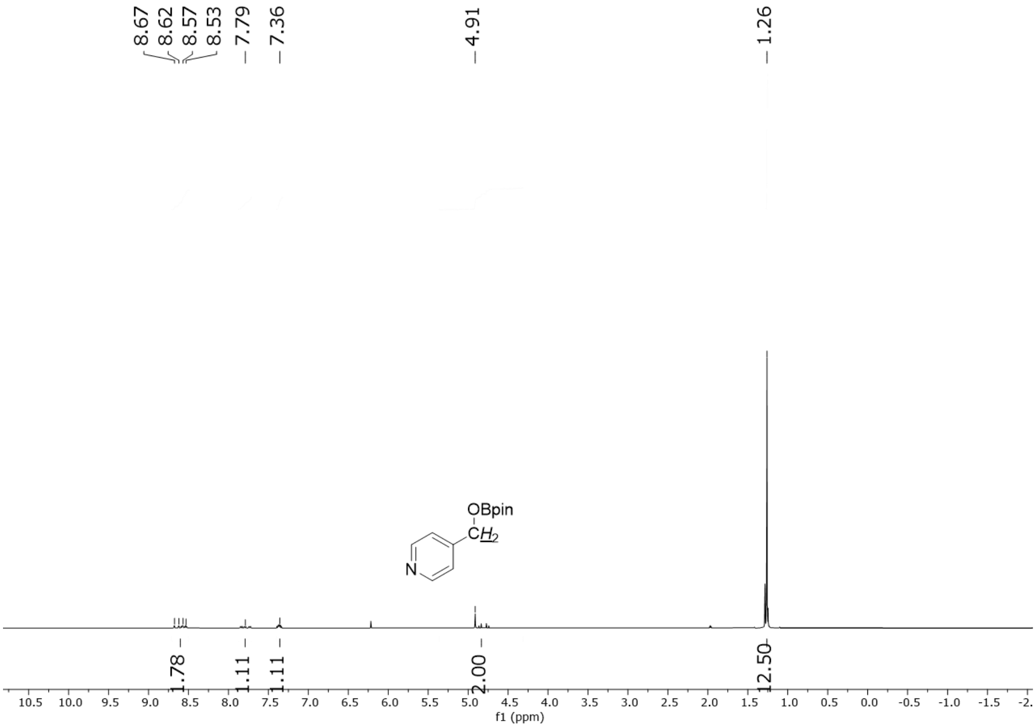

Figure S42. ^{1}H NMR spectra of $(NC_5H_4)CH_2OBpin$ in CD_3CN.

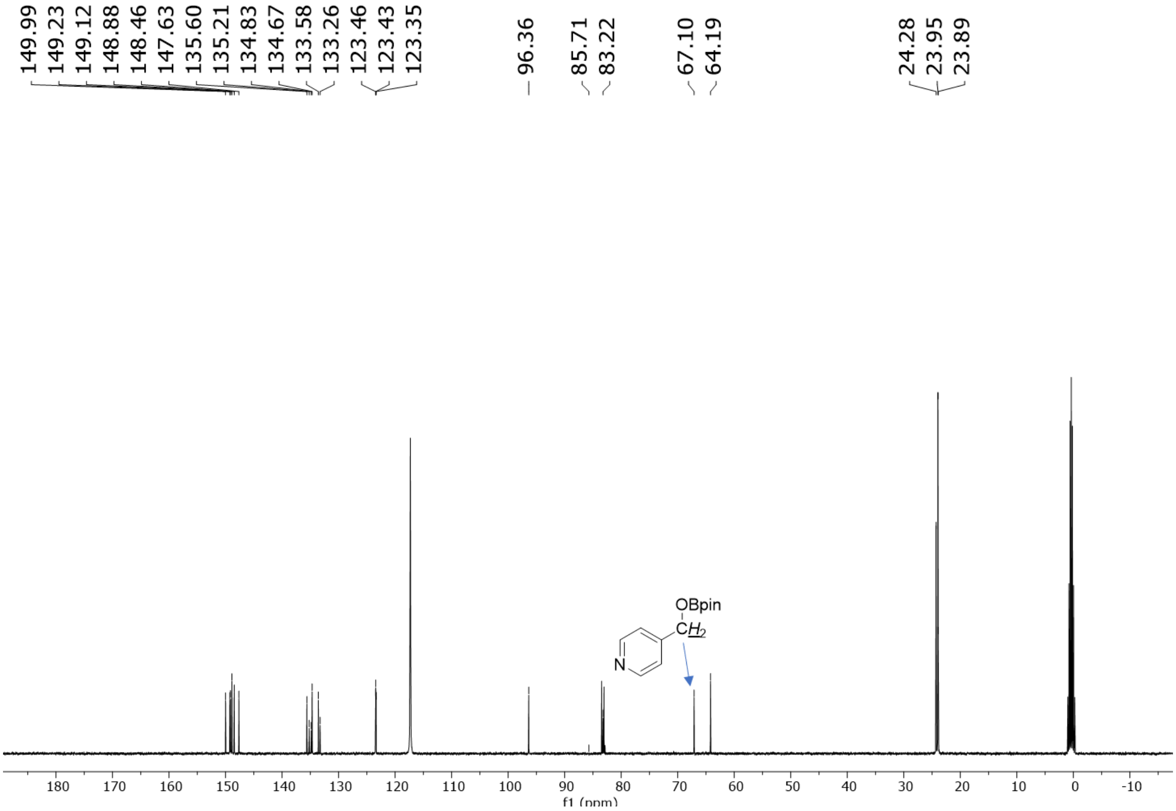

Figure S43. $^{13}C\{^{1}H\}$ NMR spectra of $(NC_5H_4)CH_2OBpin$ in CD_3CN.

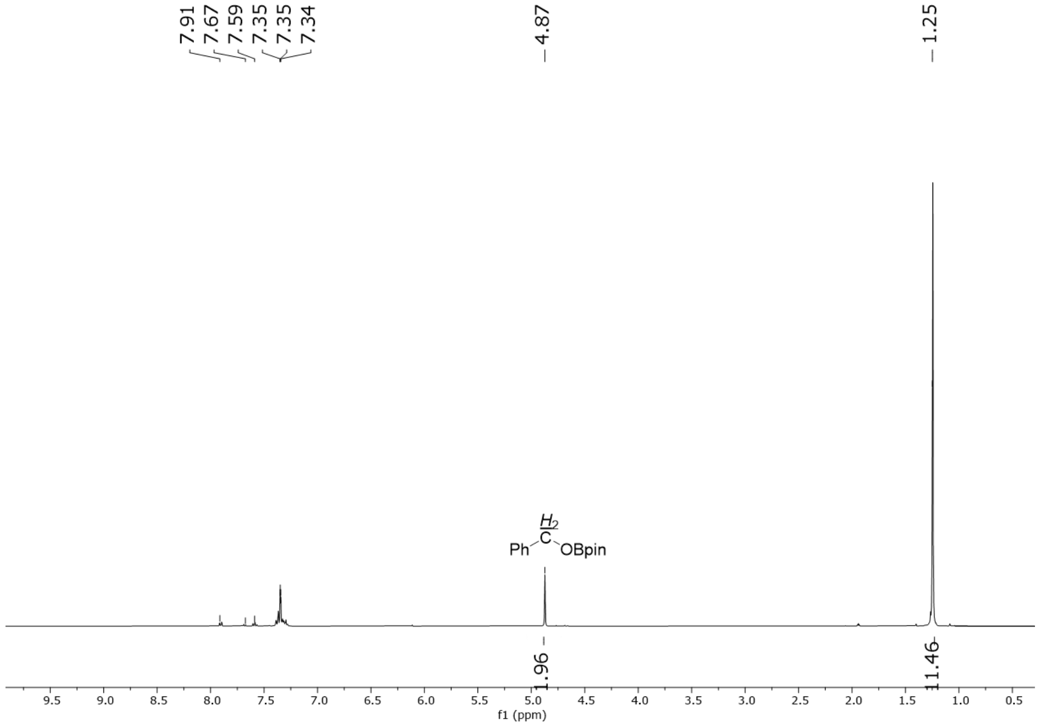

Figure S44. ^{1}H NMR spectra of Ph(CH_2)OBpin in CD_3CN.

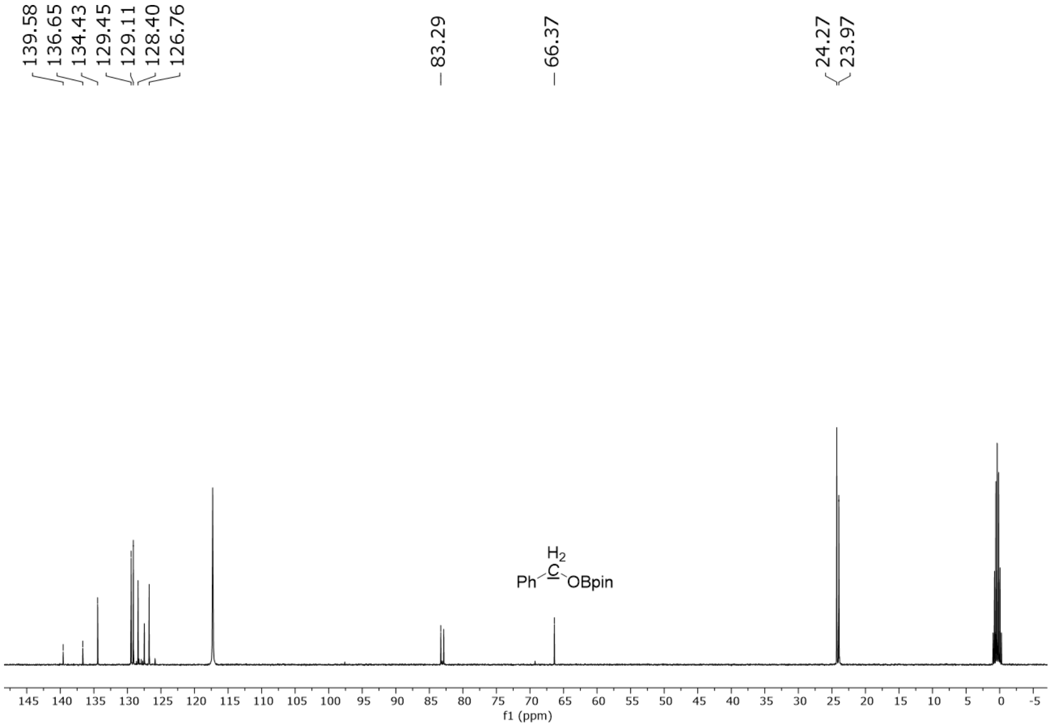

Figure S45. $^{13}C\{^{1}H\}$ NMR spectra of Ph(CH_2)OBpin in CD_3CN.

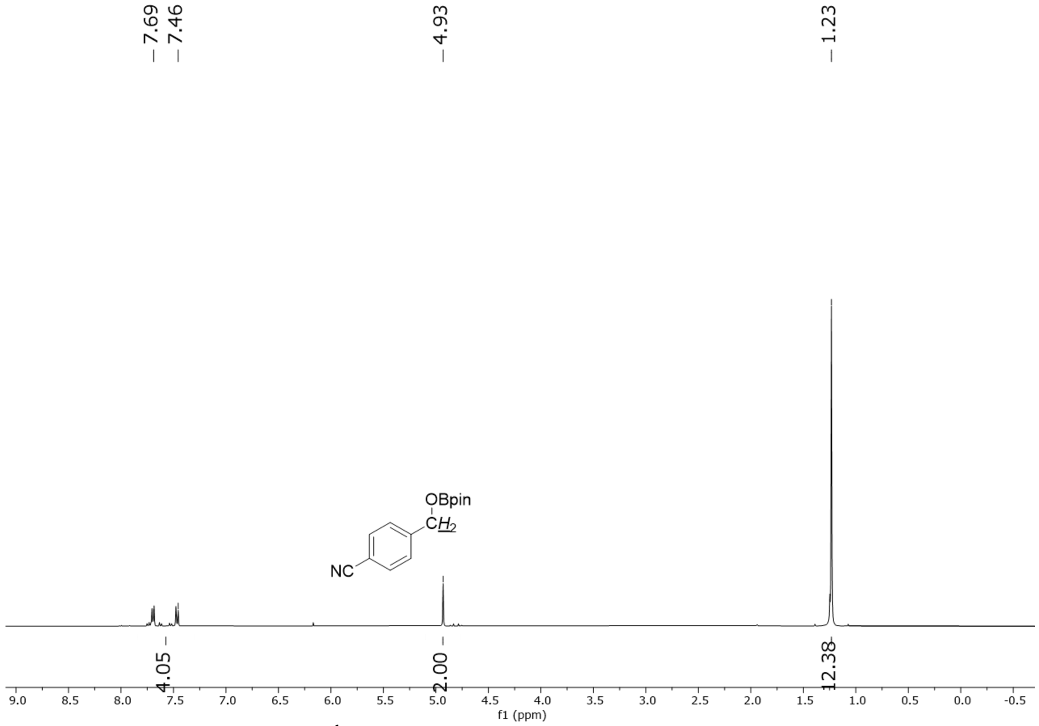

Figure S46. ^{1}H NMR spectra of CNPh(CH_2)OBpin in CD_3CN.

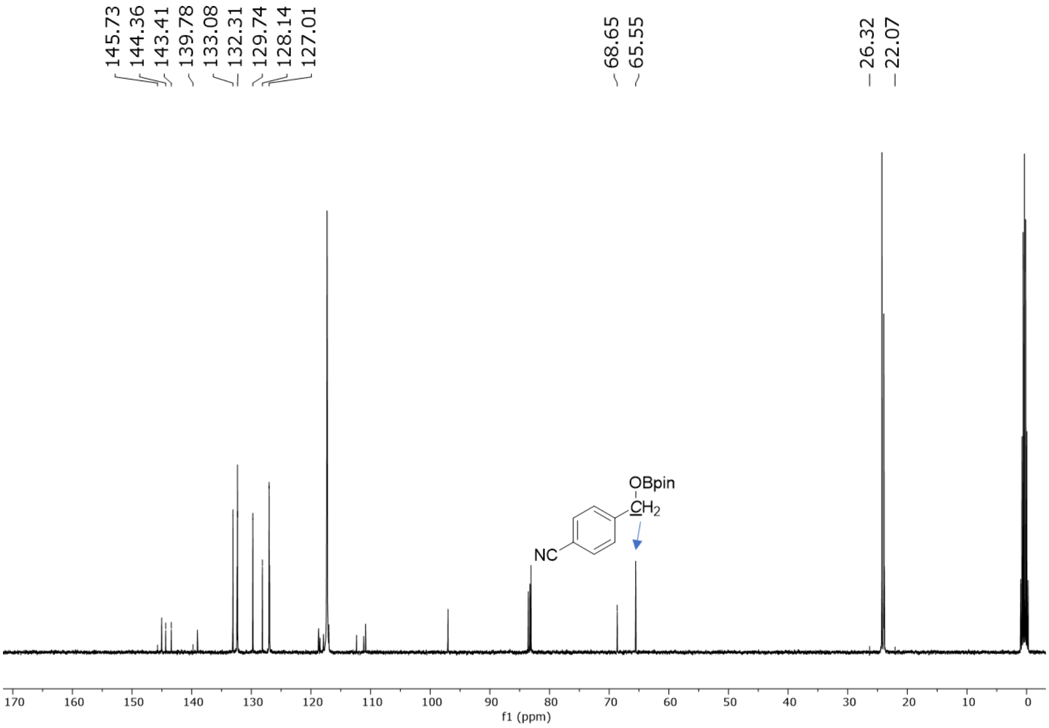

Figure S47. $^{13}C\{^{1}H\}$ NMR spectra of CNPh(CH_2)OBpin in CD_3CN.

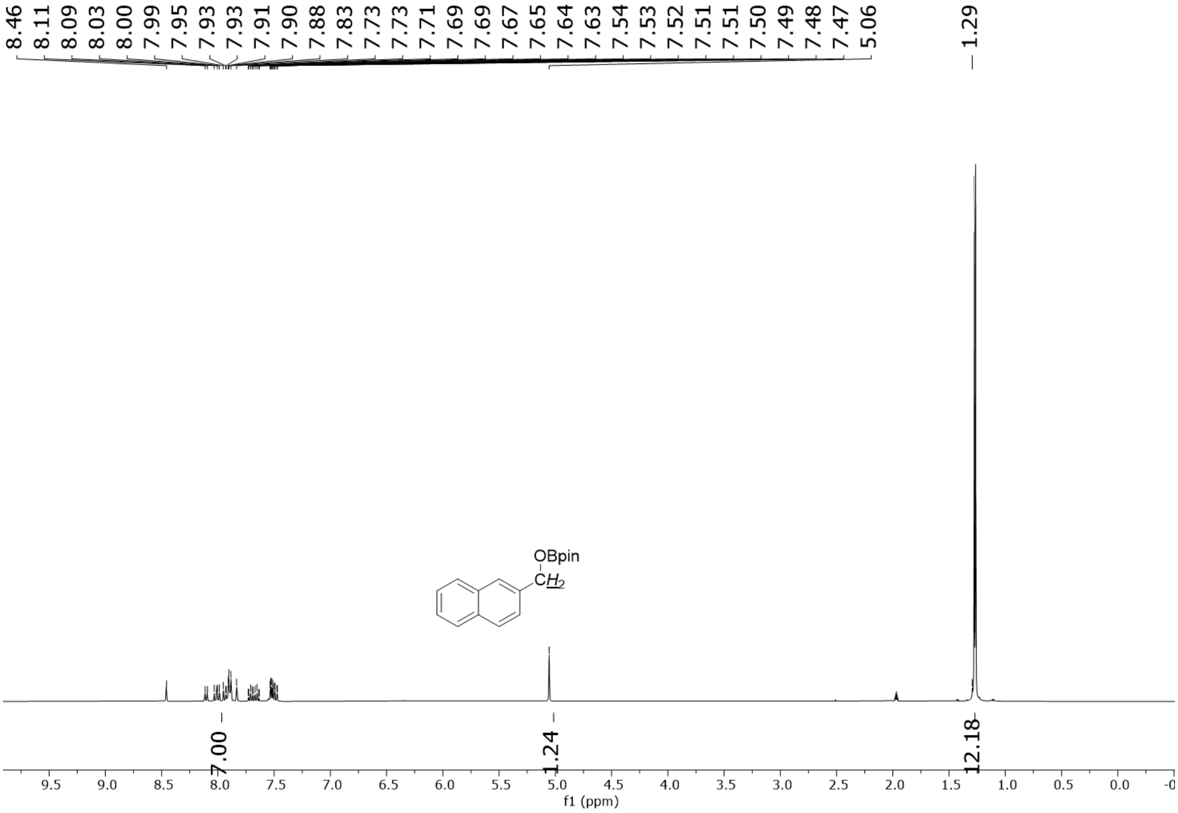

Figure S48. ^{1}H NMR spectra of $(C_{10}H_7)(CH_2)OBpin$ in CD_3CN.

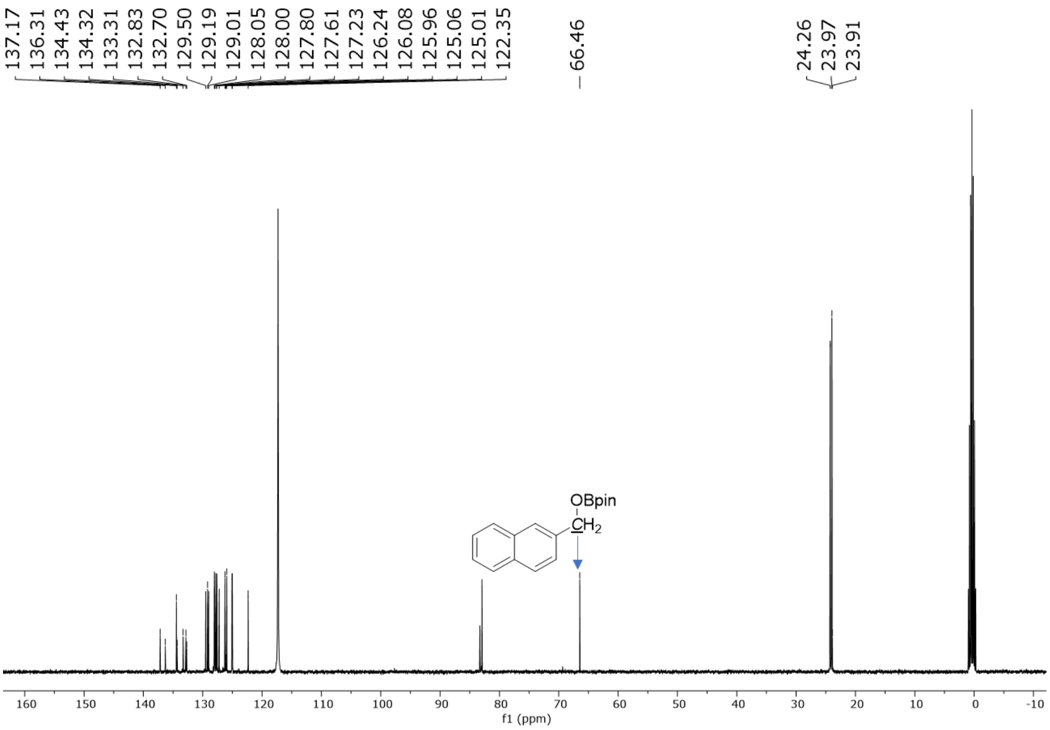

Figure S49. $^{13}C\{^{1}H\}$ NMR spectra of $(C_{10}H_7)(CH_2)OBpin$ in CD_3CN.

2. DFT Calculations

Computational Details

All calculations reported in this article were performed employing a DFT method implemented in the Gaussian 09 suite of programs.[S2] Geometry optimizations were carried out using gradient-corrected BP86 functional[S3,S4] in conjunction with the Ahlrichs' split valence plus polarization basis set (def2-SVP)[S5,S6] for all the atoms. BP86 is composed of Becke's 1988 exchange and Perdew's 1986 correlation functionals. No symmetry constraints were imposed during structural optimizations. The frequency analyses were conducted at the same level of theory to ascertain the nature of stationary points and also to obtain the thermochemical energy values. The stationary points were characterized as either real minimum (no imaginary frequency) or saddle point (one imaginary frequency). The transition states, which are the first-order saddle points on the potential energy surface, were searched using the linear synchronous transit (LST) method,[S7] and subsequent optimizations were performed by utilizing the default Berny algorithm, implemented in the Gaussian 09 code. Intrinsic reaction coordinate (IRC) calculations were enforced to ensure that the transition state connects the corresponding real minima. Furthermore, to improve the accuracy of the energies obtained from the R-BP86/def2-SVP calculations, single-point calculations were performed on the optimized geometries employing Truhlar's global hybrid meta generalized gradient approximation (GGA) functional, M06-2X,[S8] in conjunction with the triple-ζ valence plus polarization basis set (def2-TZVP)[S5,S6] for all the atoms. Tight wave function convergence criteria and "ultrafine" (99,950) grid were used in numerical integration during single-point calculations. Additionally, to incorporate the London dispersion effects, we used Grimme's D3 empirical corrections.[S9] Wiberg bond indices (WBI) were calculated at the same level of theory.[S10] Solvation energies in acetonitrile (ε =35.688) solvent were evaluated by a self-consistent reaction field (SCRF) approach using the SMD continuum solvation model.[S11] All the energy values (ΔG_L^S) reported in the manuscript are obtained at the R-M06-2X-D3/def2-TZVP(SMD)//R-BP86/def2-SVP level. The Gibbs free energy in the solution phase (G_L^S) was calculated as: $G_L^S = H_L^S - TS_L^S$. Here, the solvation enthalpy (H_L^S) was calculated from the total solvent electronic energy (E_L^S) and the enthalpy correction at the lower basis set, whereas the solvation entropy (S_L^S) was estimated as half of the gas-phase value.[S12] Moreover, to better understand the nature of the chemical bonding in selected intermediates and transition states, NOCV (natural orbital for chemical valence)[S13] calculations, developed by Mitoraj and Michalak, were accomplished on the R-BP86/def2-SVP optimized geometries using BP86-D3 functional in ADF 2020.101 program package.[S14] For NOCV calculations, all the atoms were treated with TZ2P basis set.[S15] Uncontracted Slater-type orbitals (STOs) were used as basis functions for the SCF calculations.[S16] Scalar relativistic effects were treated using the zeroth-order regular approximation (ZORA)[S17] without any frozen core approximation. This level of theory is denoted as R-BP86-D3/TZ2P(ZORA)//R-BP86/def2-SVP. Orbital diagrams were rendered in Chemcraft[S18] and optimized geometries were prepared using the CYLview[S19] visualization software. During the mechanistic investigation, we have truncated the bulkier m-terphenyl group with 2,6-dimethylphenyl group (Ar) to reduce the computational cost.[S1]

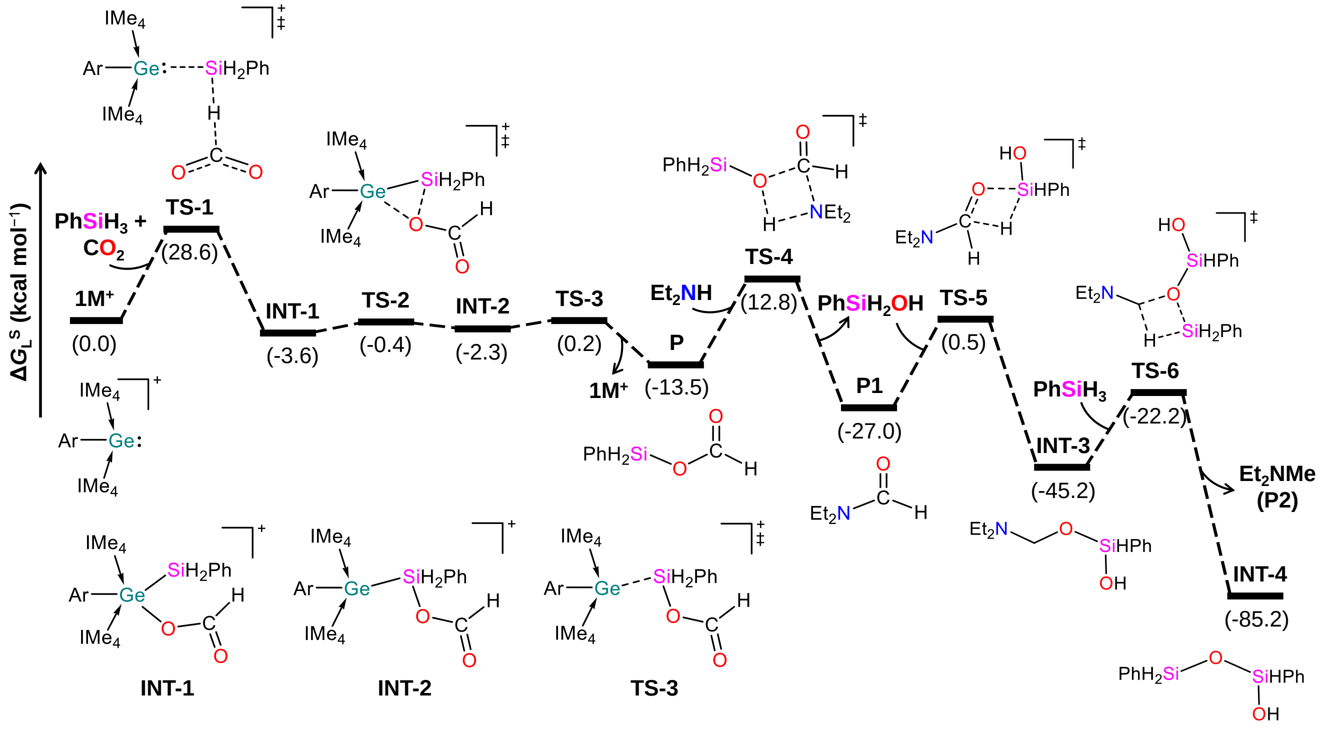

Figure S50. Energy profile for the **1M⁺** catalyzed *N*-functionalization of amine using CO_2 and $PhSiH_3$. Ar = 2,6-dimethylphenyl.

DFT calculations were performed to explore the mechanistic pathways for **1M⁺** catalyzed formation of formoxysilane (Figure S50). The activation of the Si−H bond in $PhSiH_3$ by the germanium lone pair in **1M⁺** and participation of free CO_2 as a hydride acceptor from the hypercoordinate silane occurs in a concerted process via the transition state **TS-1**. This step needs to surmount an energy barrier of 28.6 kcal mol^{-1}. To gain further insight into this reaction step, we have performed IRC calculations on the optimized geometry of **TS-1** in both forward and backward (reverse) directions. IRC forward calculations ensure the formation of the intermediate **INT-1**. On the other hand, IRC reverse calculations lead to separated species (**1M⁺** + $PhSiH_3$ + CO_2) with no interaction and the formation of any adduct of **1M⁺** with $PhSiH_3$ or CO_2 prior to **TS-1** was not observed. Direct formation of the transition state from the separated species following concerted S_N2 or three-component mechanism rather than the classical activation of hydrosilanes is also clearly evident from the substantially longer Ge−Si distance (2.787 Å) in **TS-1**.[S20] The resulting intermediate **INT-1** finally delivers formoxysilane (**P**), accompanying an energy barrier of 3.8 kcal mol^{-1}. **P** reacts with the amine via **TS-4** to furnish the formamide species (**P1**) with the liberation of $PhSiH_2OH$. The reduction of formamide by $PhSiH_2OH$ followed by similar reduction of the resulting intermediate **INT-3** by $PhSiH_3$ affords the *N*-methylated product (**P2**) and silaxone species (**INT-4**).

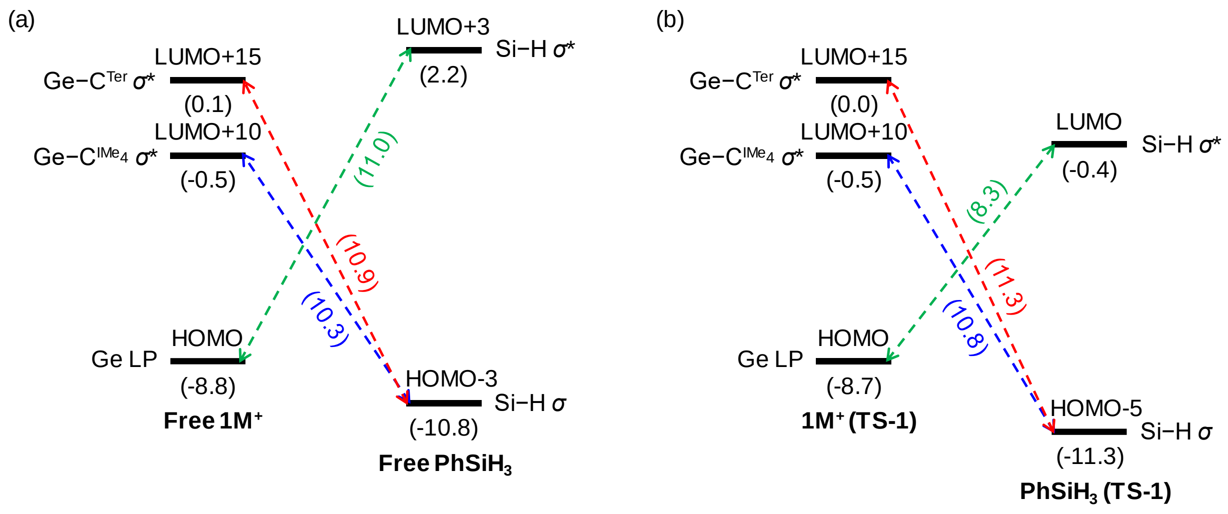

Figure S51. (a) orbital interaction between free **1M**+ and PhSiH3. (b) orbital interaction between **1M**+ and PhSiH3 fragments in **TS-1** at the R-M06-2X-D3/def2-TZVP//R-BP86/def2-SVP level. The orbital energies are in eV.

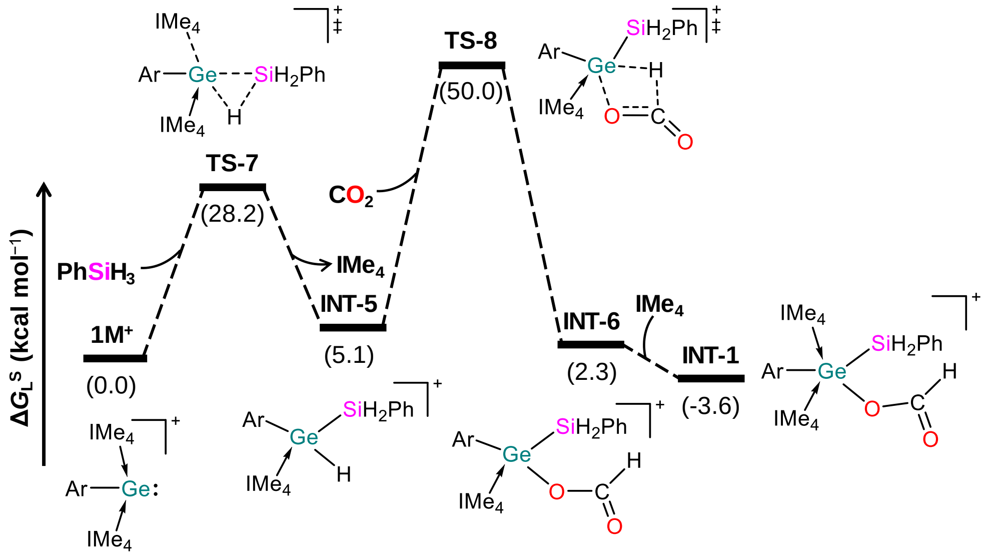

Figure S52. Energy profile for the unfavorable pathway of **1M**+ catalyzed formation of formoxysilane. Ar = 2,6-dimethylphenyl.

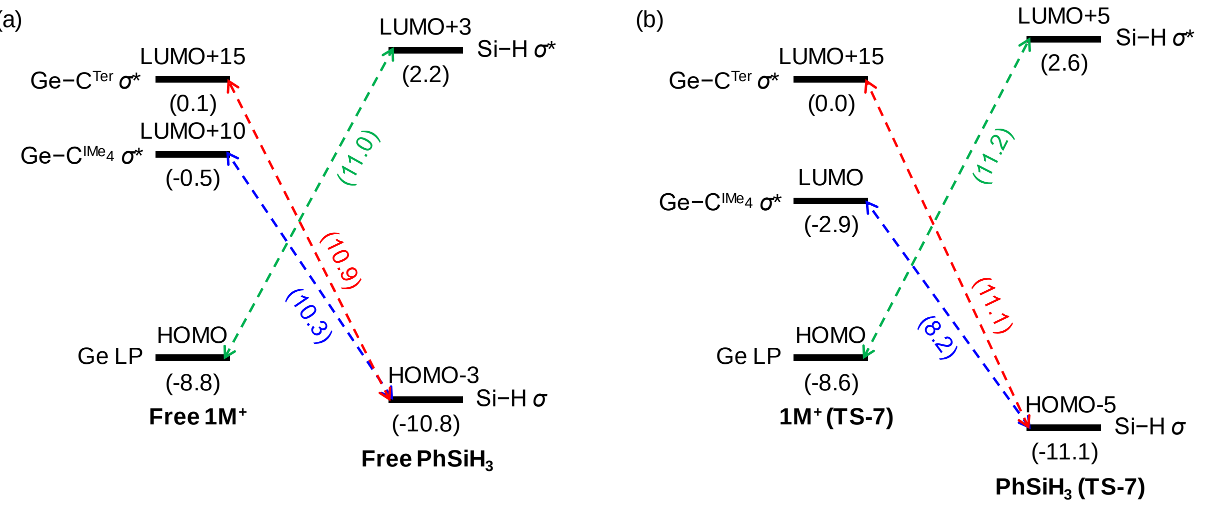

Figure S53. (a) orbital interaction between free **1M**+ and PhSiH3. (b) orbital interaction between **1M**+ and PhSiH3 fragments in **TS-7**. The orbital energies are in eV.

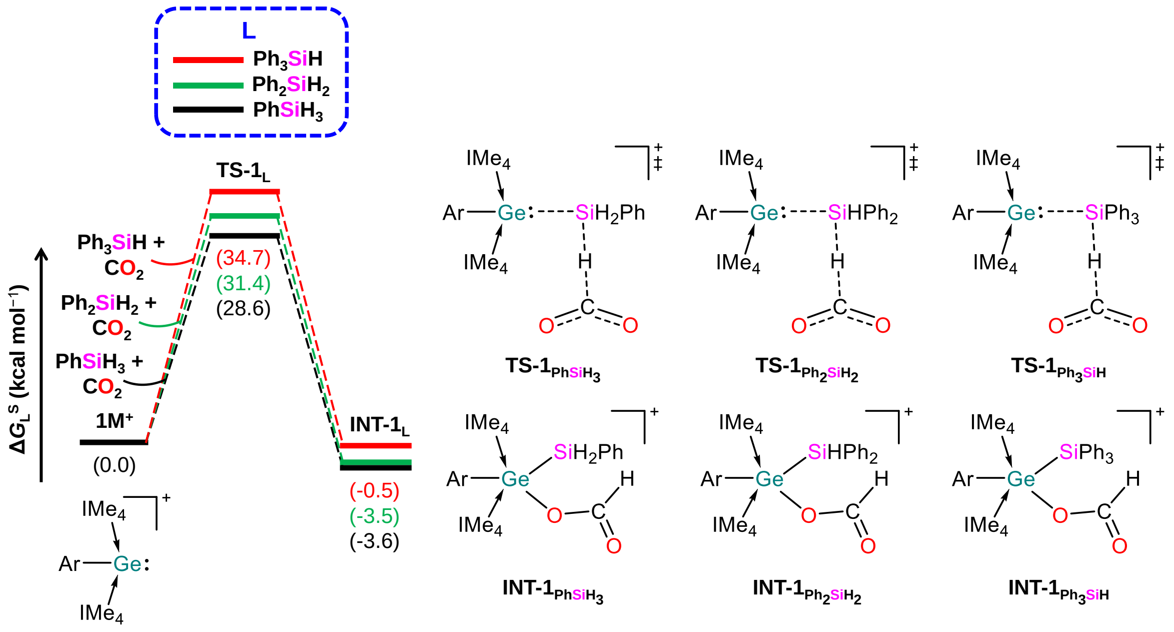

Figure S54. The calculated activation barriers for the **1M**⁺ catalyzed formation of formoxysilane in the reaction of CO2 and various silanes. Ar = 2,6-dimethylphenyl.

We have also calculated the rate-limiting energy barriers for the formation of formoxysilane in the presence of more sterically protected silanes (Figure S54). The computed activation barriers follow the trend: **TS-1**$_{\mathbf{PhSiH_3}}$ (28.6 kcal mol^{-1}) < **TS-1**$_{\mathbf{Ph_2SiH_2}}$ (31.4 kcal mol^{-1}) < **TS-1**$_{\mathbf{Ph_3SiH}}$ (34.7 kcal mol^{-1}). Hence our calculations nicely accord with the experimental findings.

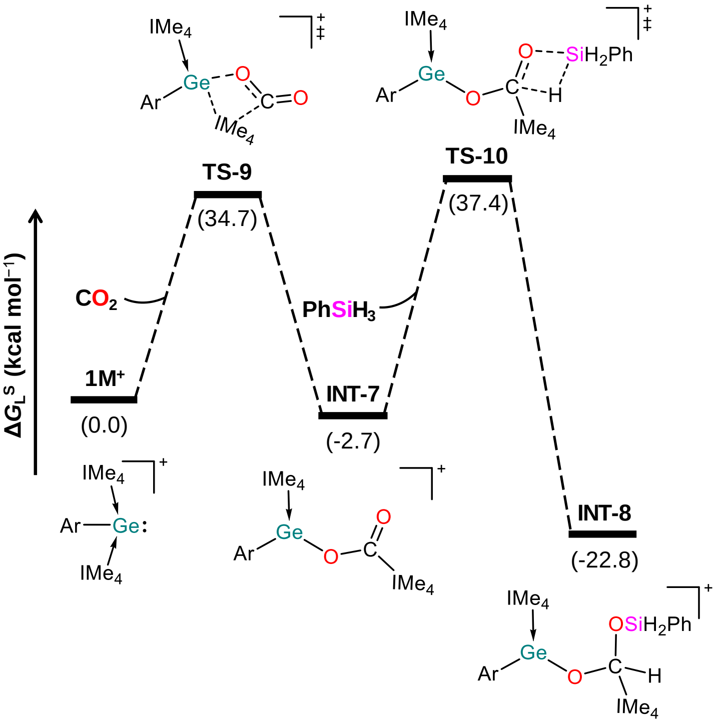

Figure S55. Energy profile for the unfavorable pathway of **1M**⁺ catalyzed formation of formoxysilane. Ar = 2,6-dimethylphenyl.

We have also checked the alternative possibility for the formation of formoxysilane via the insertion of CO_2 into the Ge−C^{IMe_4} bond in **1M⁺** followed by the reduction of **INT-7** by $PhSiH_3$. However, the reduction of **INT-7** by $PhSiH_3$ demands drastically high energy barrier of 40.1 kcal mol^{-1} and hence remains unrealistic under the reaction conditions (Figure S55).

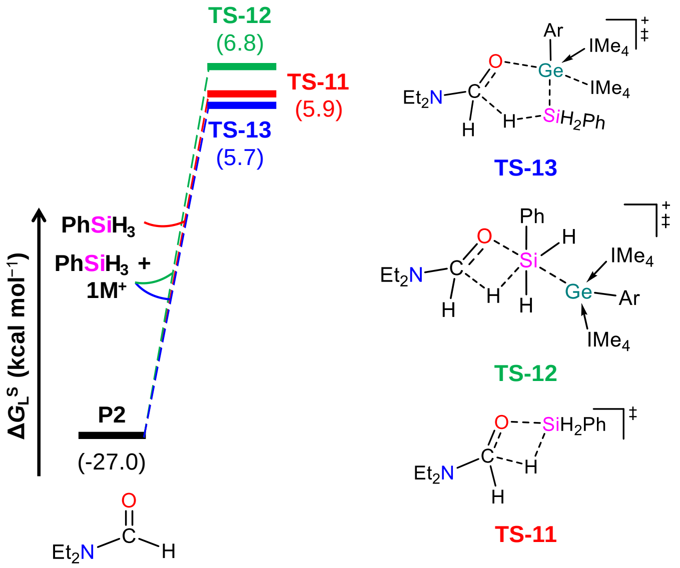

Figure S56. Energy profile for the reduction of formamide by $PhSiH_3$ both in the absence and presence of **1M⁺**.

The activation barriers for the reduction of formamide by $PhSiH_3$ both in the absence and presence of **1M⁺** are signigicantly higher (32.7−33.8 kcal mol^{-1}) compared to that for the reduction by $PhSiH_2OH$ (27.5 kcal mol^{-1}).

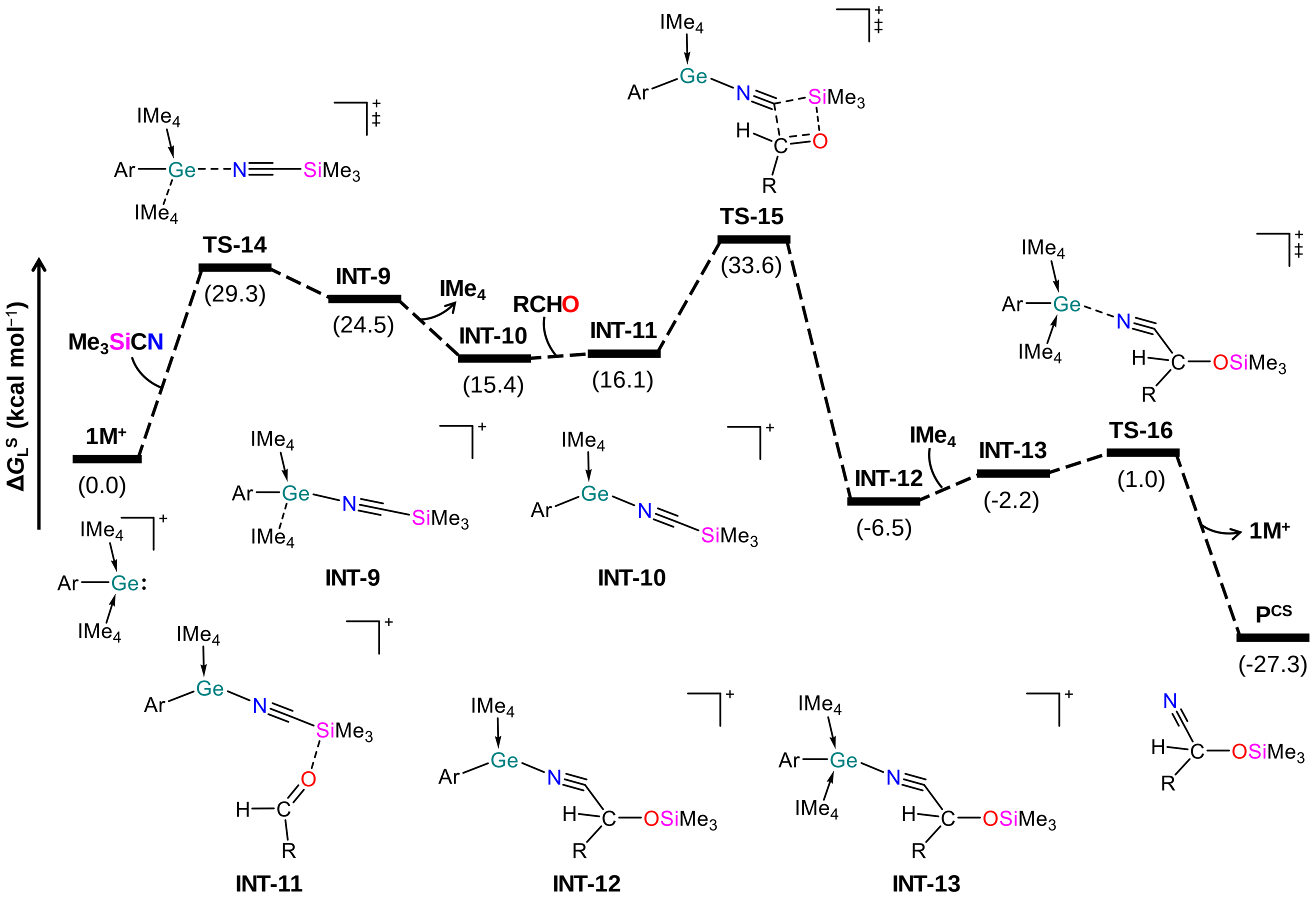

Figure S57. Energy profile for the favorable pathway of **1M⁺** catalyzed cyanosilylation of aldehyde (*Pathway-1a*). Ar = 2,6-dimethylphenyl; RCHO = Pivaldehyde.

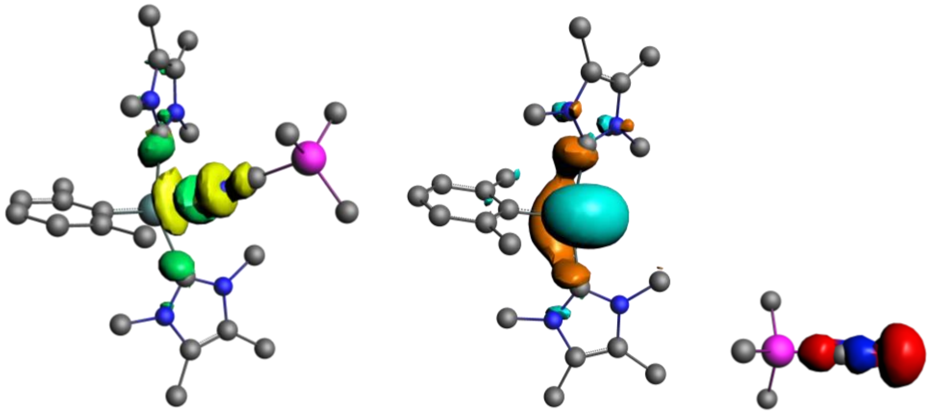

$\Delta E_{orb(1)}$ = -8.2, $|v|_{(1)}$ = 0.30 (**TS-14**)　　LUMO (**1M⁺**)　　HOMO-2 (**TMSCN**)

Figure S58. Plot of deformation density Δ*ρ* of the pairwise orbital interaction between **1M⁺** and Me_3SiCN fragments in **TS-14**. The associated energy (in kcal mol^{-1}) is given in parenthesis. The color code of the charge flow is yellow→green. The most important interacting occupied and vacant molecular orbitals of the fragments are also provided. Hydrogen atoms are omitted for clarity.

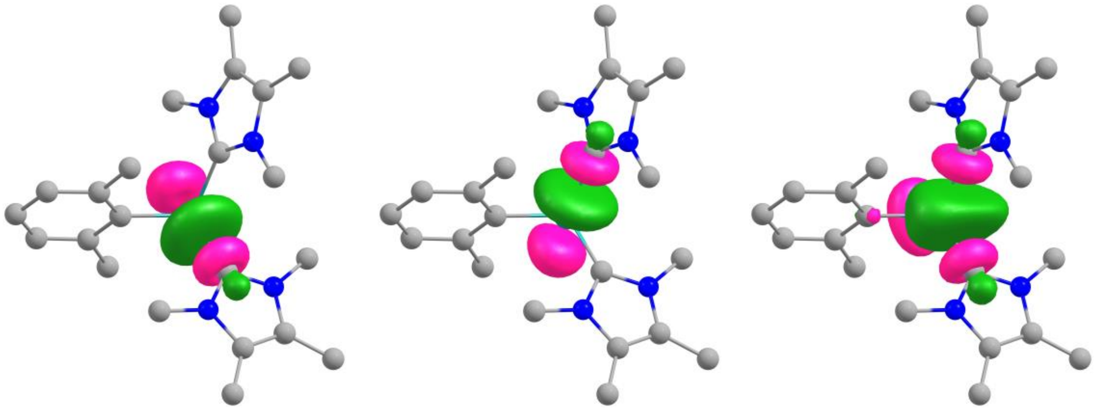

Ge−$C1^{IMe4}$ *σ** NBO　　Ge−$C2^{IMe4}$ *σ** NBO　　Ge−$C1^{IMe4}$ *σ** NBO + Ge−$C2^{IMe4}$ *σ** NBO

Figure S59. The Ge−IMe_4 *σ** NBOs in **1M⁺** (isosurface = 0.070 a.u.). Hydrogen atoms are omitted for clarity.

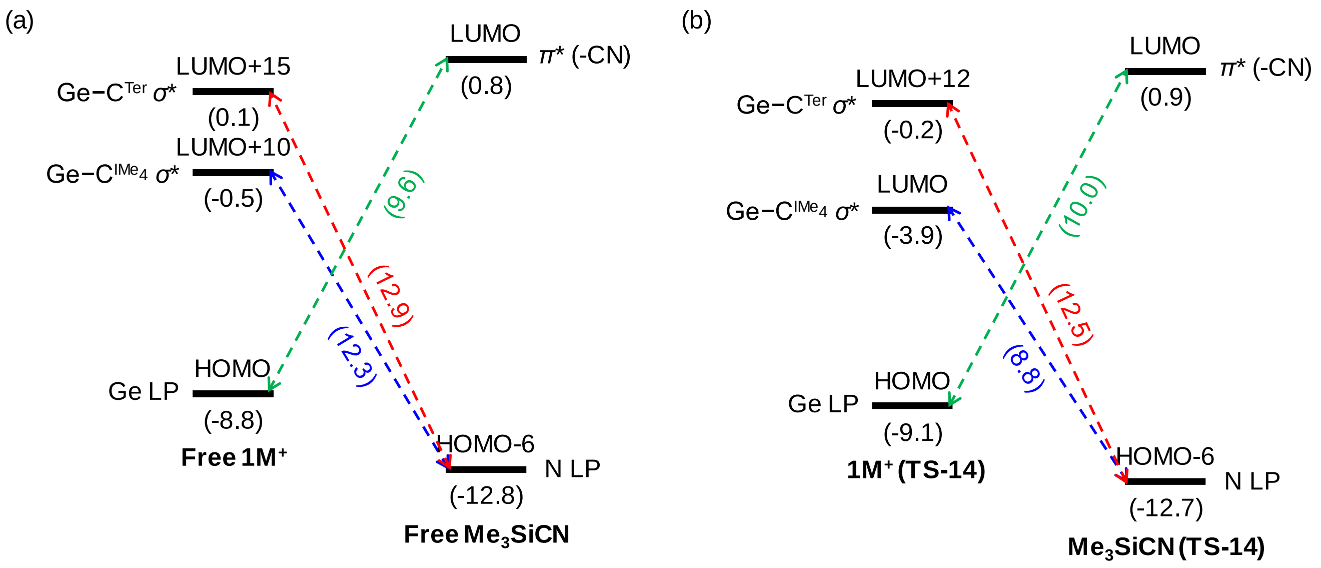

Figure S60. (a) orbital interaction between free **1M**$^{+}$ and Me3SiCN. (b) orbital interaction between **1M**$^{+}$ and Me3SiCN fragments in **TS-14**. The orbital energies are in eV.

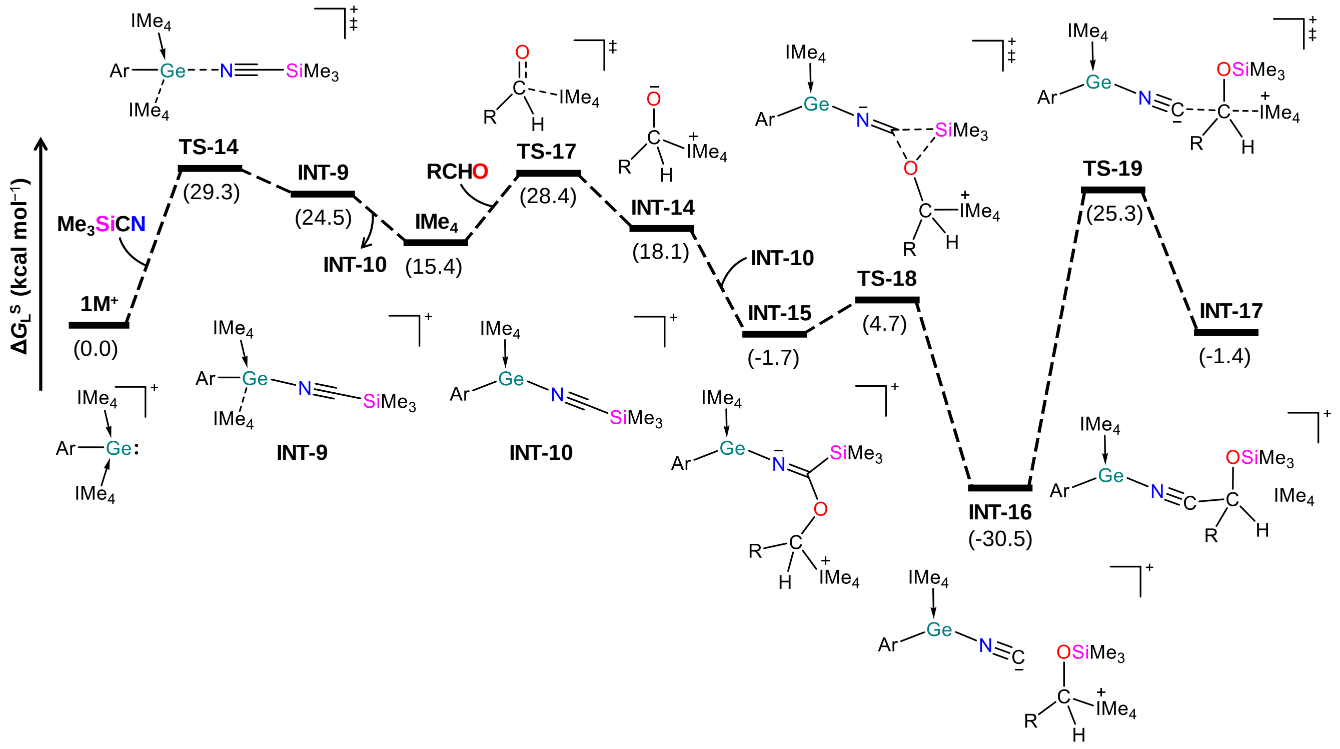

Figure S61. Energy profile for the unfavorable pathway of **1M**$^{+}$ catalyzed cyanosilylation of aldehyde (*Pathway-1b*). Ar = 2,6-dimethylphenyl; RCHO = Pivaldehyde.

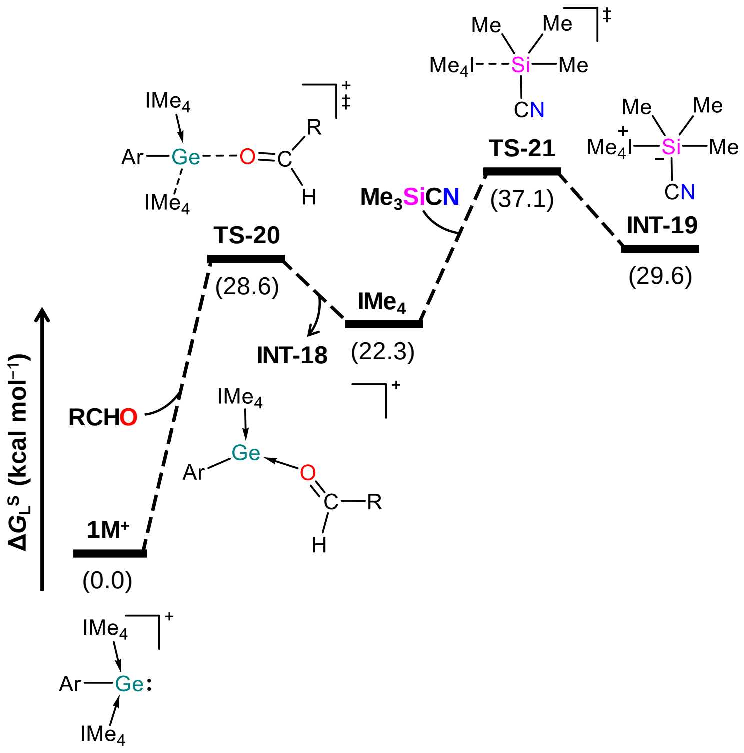

Figure S62. Energy profile for the unfavorable pathway of **1M**$^+$ catalyzed cyanosilylation of aldehyde (*Pathway-1c*). Ar = 2,6-dimethylphenyl; RCHO = Pivaldehyde.

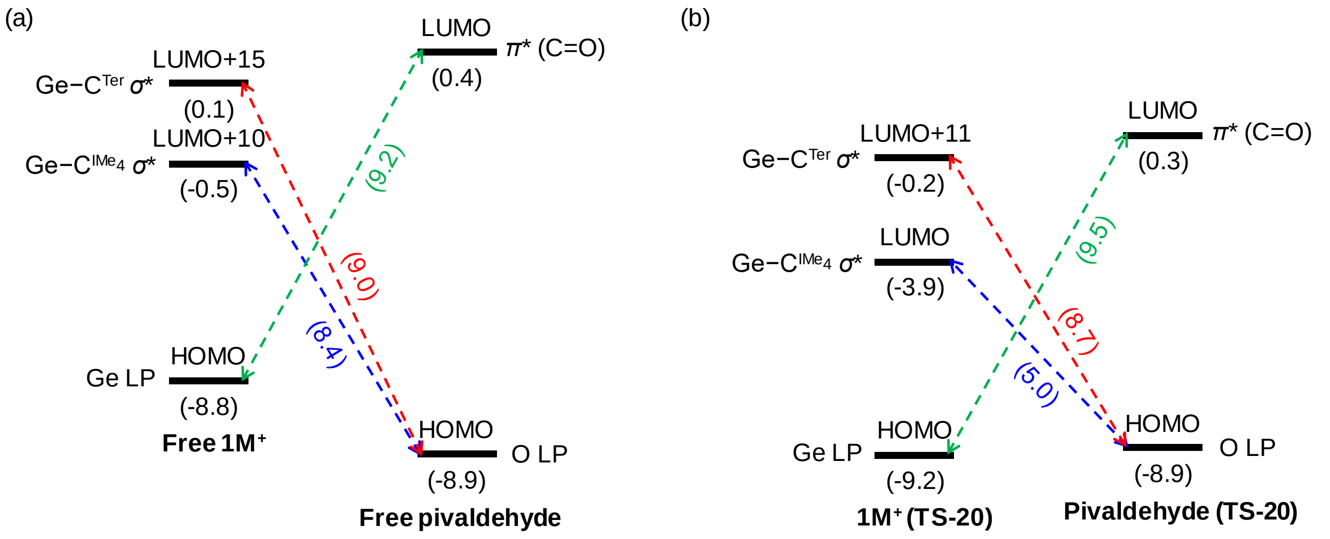

Figure S63. (a) orbital interactions between free **1M**$^+$ and pivaldehyde. (b) orbital interaction between **1M**$^+$ and pivaldehyde fragments in **TS-20**. The orbital energies are in eV.

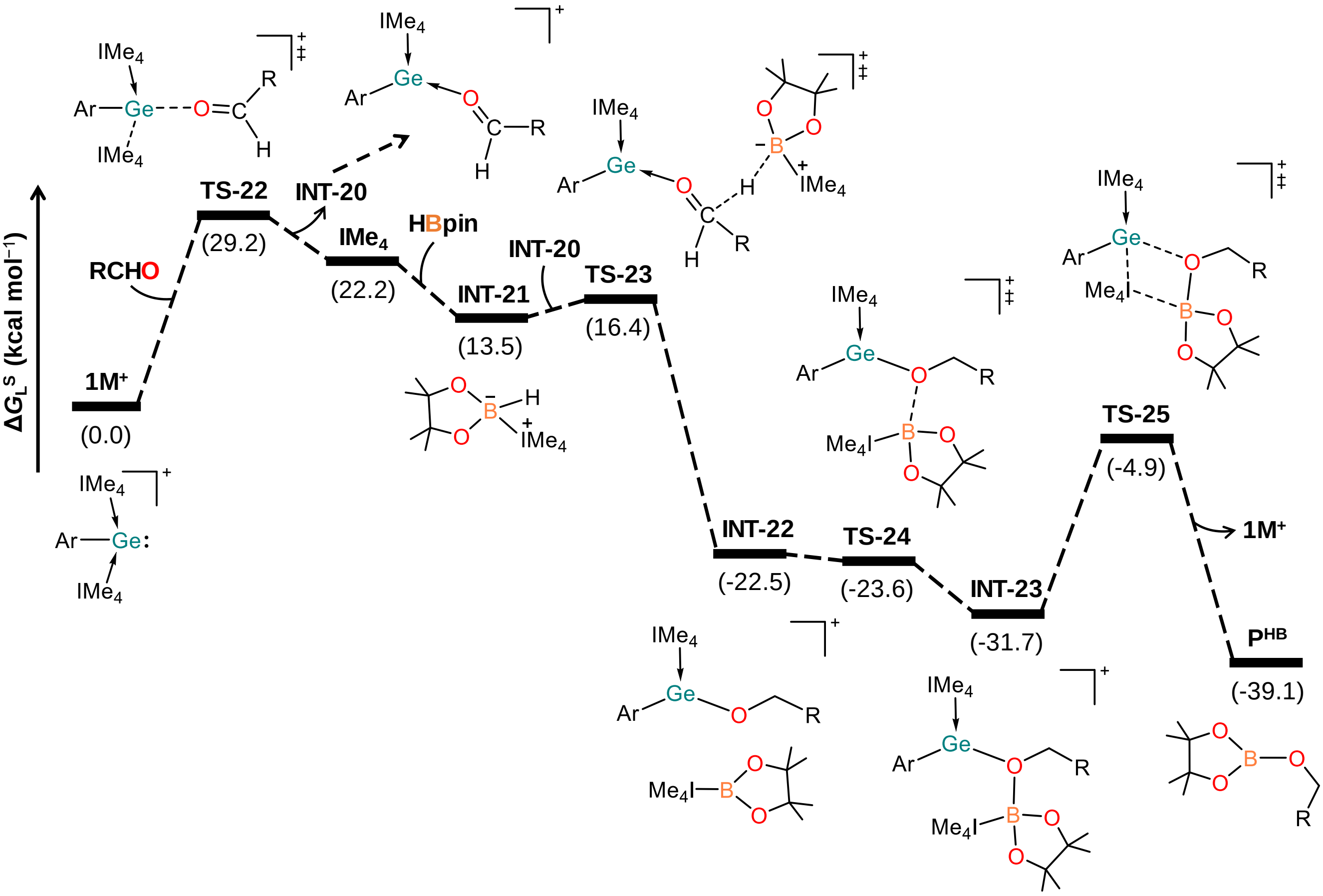

Figure S64. Energy profile for the favorable pathway of **1M**$^{+}$ catalyzed hydroboration of aldehyde (*Pathway-2a*). Ar = 2,6-dimethylphenyl; RCHO = Propanal.

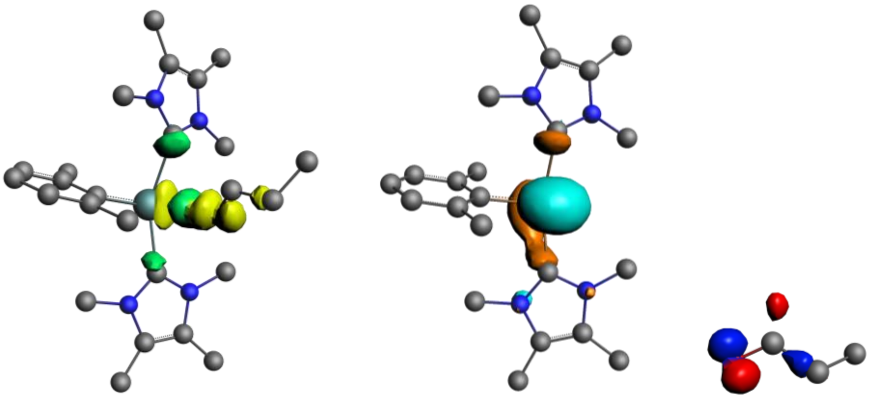

$\Delta E_{orb(1)}$ = -8.7, $|v|_{(1)}$ = 0.32 (**TS-22**) LUMO (**1M**$^{+}$) HOMO (**RCHO**)

Figure S65. Plot of deformation density $\Delta\rho$ of the pairwise orbital interaction between **1M**$^{+}$ and propanal (RCHO) fragments in **TS-22**. The associated energy (in kcal mol^{-1}) is given in parenthesis. The color code of the charge flow is yellow→green. The most important interacting occupied and vacant molecular orbitals of the fragments are also provided. Hydrogen atoms are omitted for clarity.

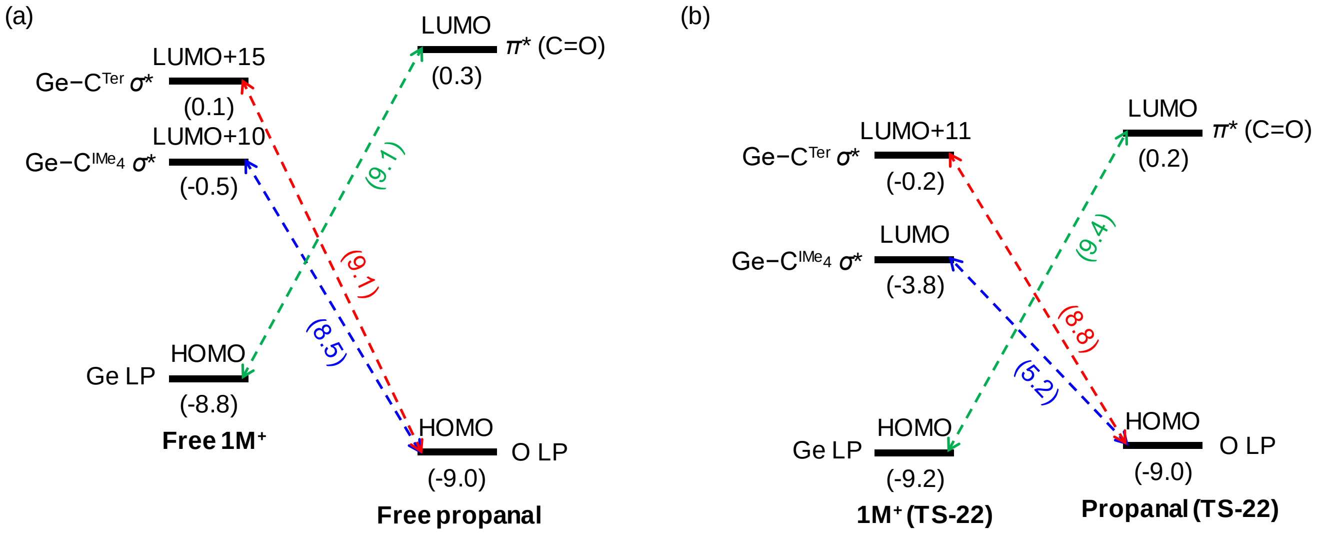

Figure S66. (a) orbital interactions between free **1M**$^{+}$ and propanal. (b) orbital interaction between **1M**$^{+}$ and propanal fragments in **TS-22**. The orbital energies are in eV.

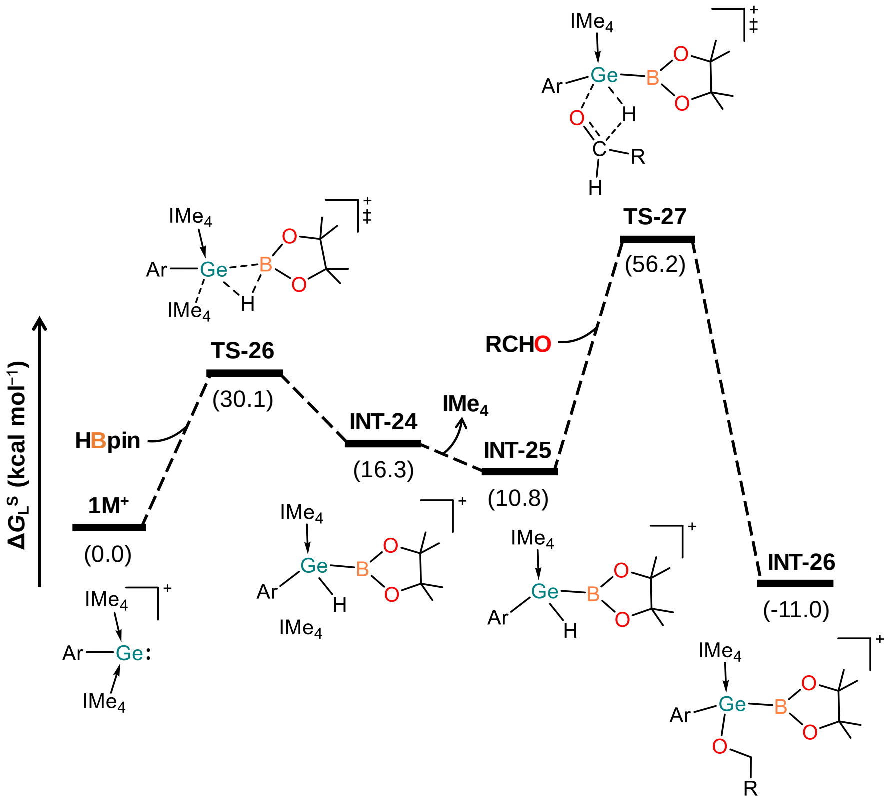

Figure S67. Energy profile for the unfavorable pathway of **1M**$^{+}$ catalyzed hydroboration of aldehyde (*Pathway-2b*). Ar = 2,6-dimethylphenyl; RCHO = Propanal.

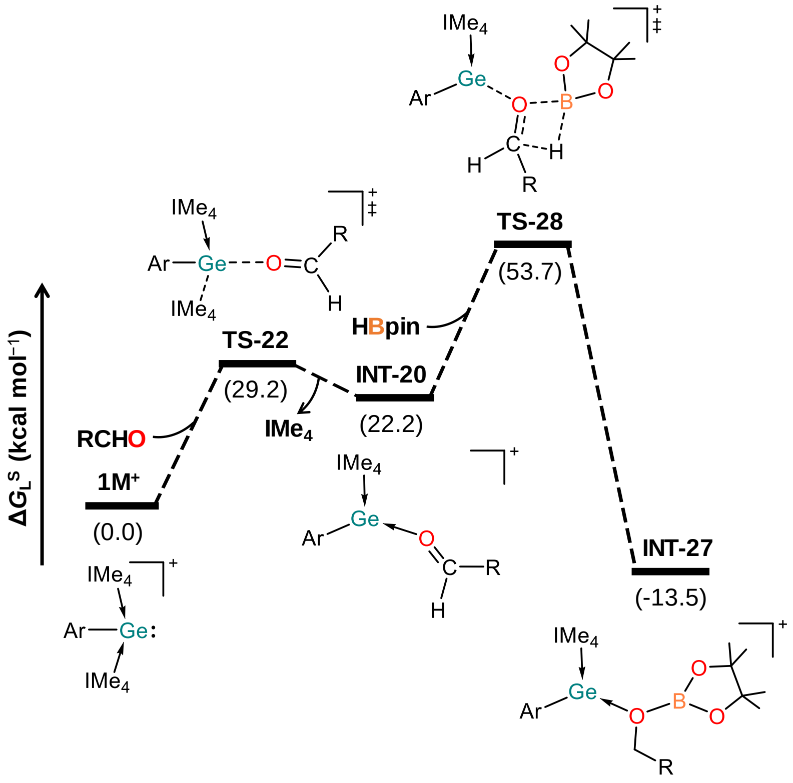

Figure S68. Energy profile for the unfavorable pathway of **1M**[+] catalyzed hydroboration of aldehyde (*Pathway-2c*). Ar = 2,6-dimethylphenyl; RCHO = Propanal.

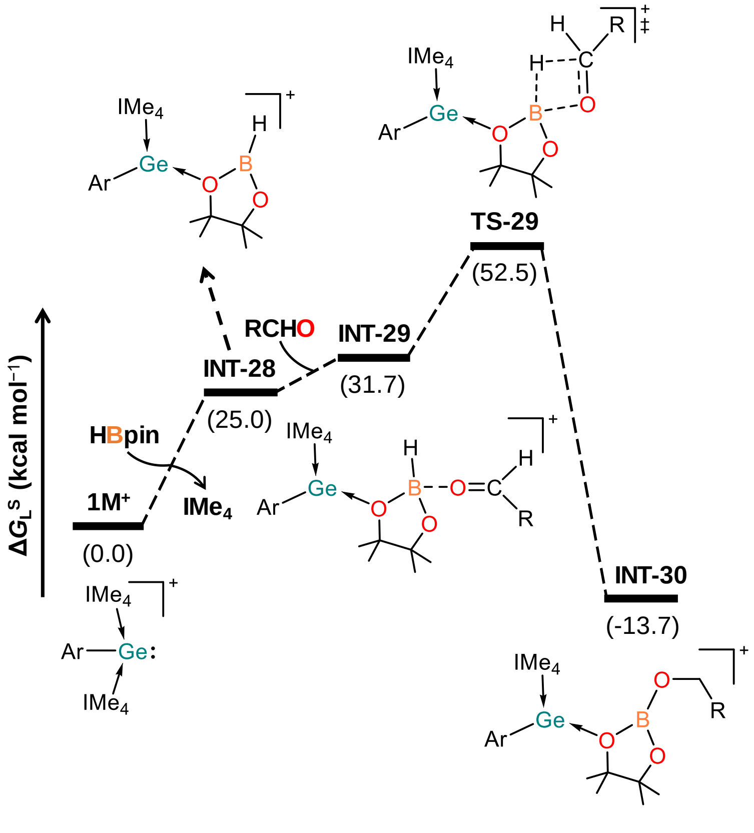

Figure S69. Energy profile for the unfavorable pathway of **1M**[+] catalyzed hydroboration of aldehyde (*Pathway-2d*). Ar = 2,6-dimethylphenyl; RCHO = Propanal.

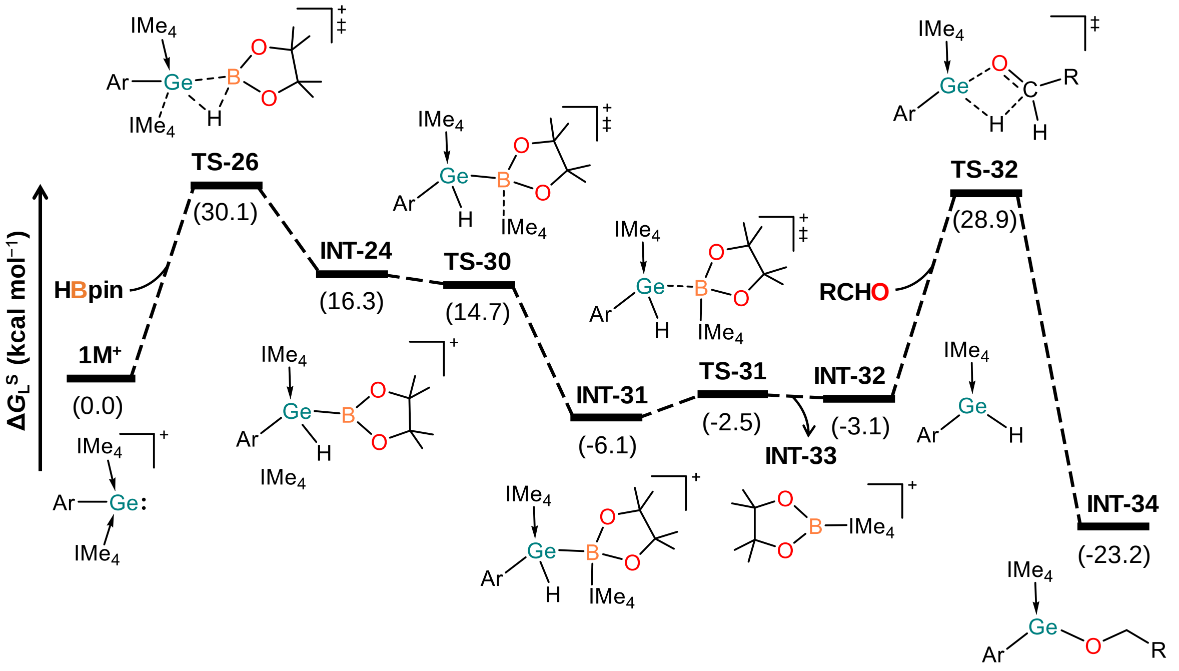

Figure S70. Energy profile for the unfavorable pathway of **1M**⁺ catalyzed hydroboration of aldehyde (*Pathway-2e*). Ar = 2,6-dimethylphenyl; RCHO = Propanal.

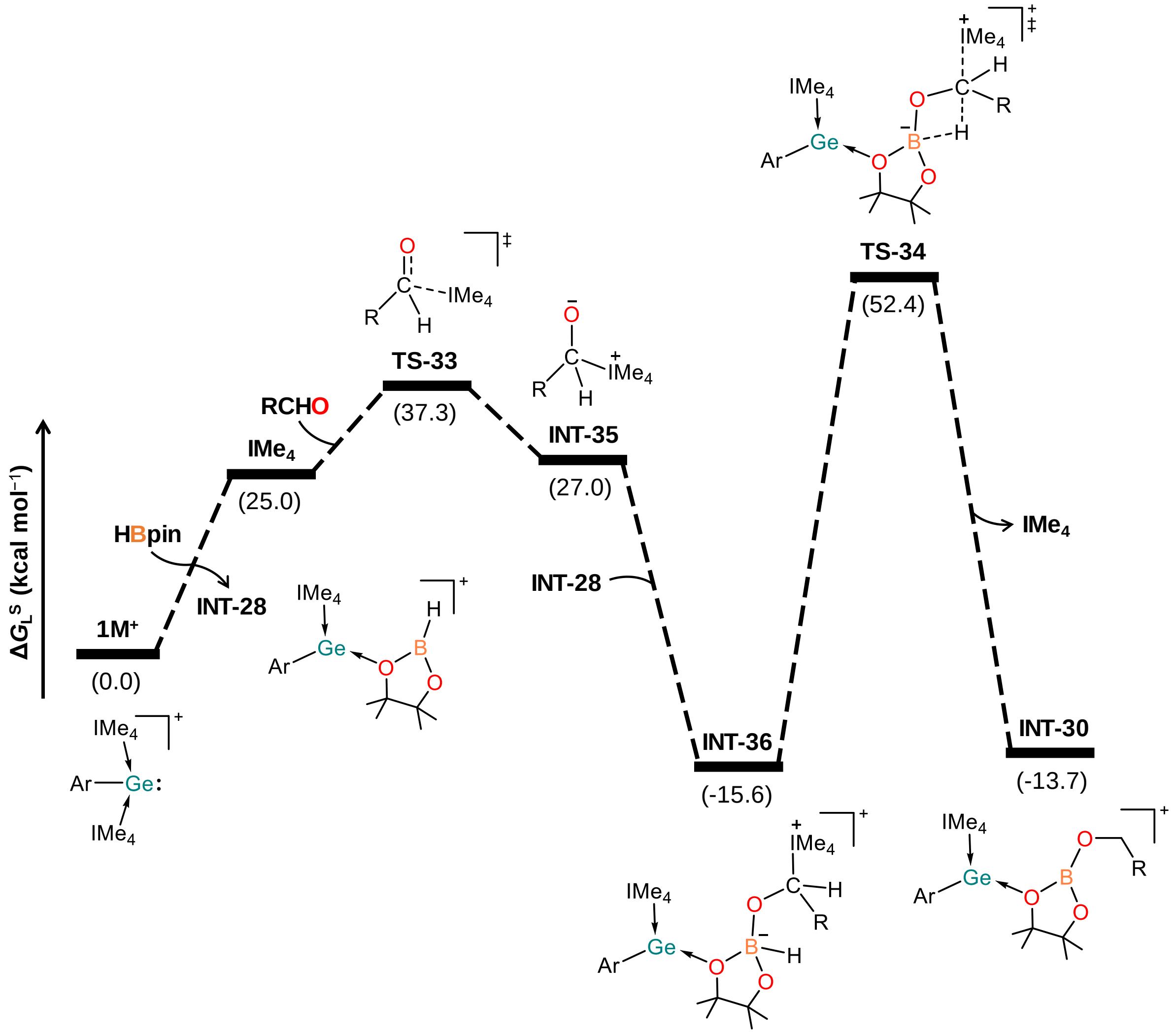

Figure S71. Energy profile for the unfavorable pathway of **1M**⁺ catalyzed hydroboration of aldehyde (*Pathway-2f*). Ar = 2,6-dimethylphenyl; RCHO = Propanal.

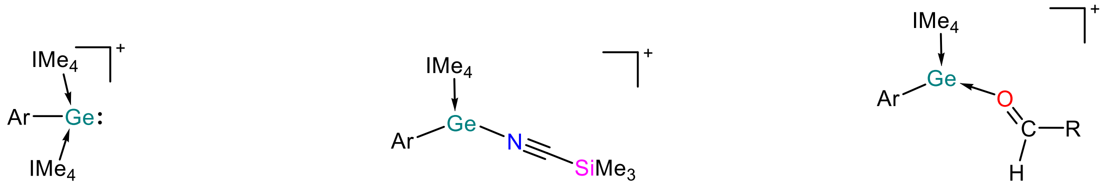

$1M^+$ q_{Ge} = +0.704 *e* | **INT-10** q_{Ge} = +0.922 *e* | **INT-20** q_{Ge} = +0.957 *e*

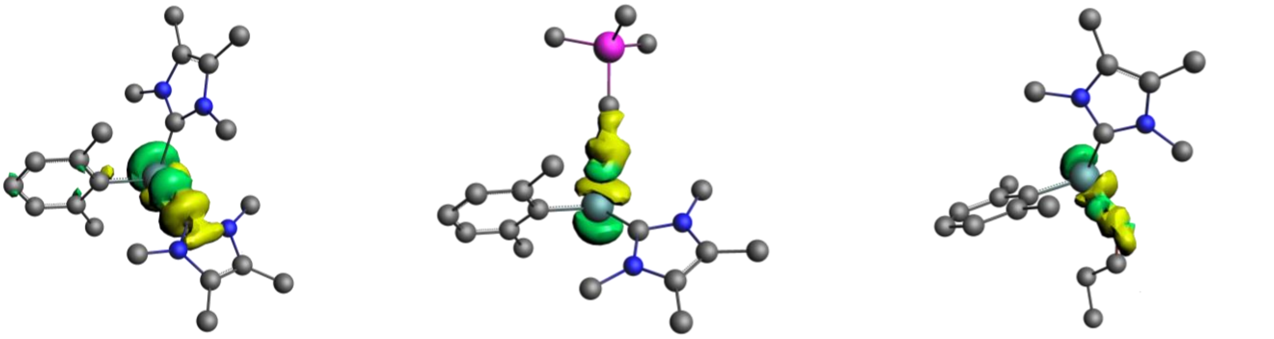

$\Delta E_{orb(1)}$ = -81.9, $|\nu|_{(1)}$ = 0.91 (**$1M^+$**) $\Delta E_{orb(1)}$ = -36.5, $|\nu|_{(1)}$ = 0.60 (**INT-10**) $\Delta E_{orb(1)}$ = -29.7, $|\nu|_{(1)}$ = 0.59 (**INT-20**)

Figure S72. Plot of deformation densities $\Delta\rho$ of the pairwise orbital interactions between $[ArGe(IMe_4)]^+$ and IMe_4/Me_3SiCN/RCHO fragments in **$1M^+$**/**INT-10**/**INT-20**. The associated energies (in kcal mol^{-1}) are given in parentheses. The color code of the charge flow is yellow→green. Ar = 2,6-dimethylphenyl; RCHO = Propanal.

Our calculations indicate that the intermediates **INT-10**/**INT-20** formed by the ligand exchange of IMe_4 in **$1M^+$** by Me_3SiCN/RCHO are significantly less stable than **$1M^+$** by 15.4/22.2 kcal mol^{-1}. The relative destabilizations of **INT-10** and **INT-20** than **$1M^+$** can be nicely correlated with the NPA charges on the germanium centers (q_{Ge}: +0.704/+0.922/0.957 *e* in **$1M^+$**/**INT-10**/**INT-20**) and also the strengths of σ-donation abilities of Me_3SiCN and RCHO compared to IMe_4, as suggested by NOCV calculations (Figure S72).

Lewis basicity of germyliumylidene towards organic substrates:

The detailed theoretical explorations nicely suggest that the germyliumylidene catalyst (**$1M^+$**) acts as a Lewis base towards silane in the hydrosilylation of CO_2, whereas **$1M^+$** exhibits Lewis acidity towards Me_3SiCN and RCHO in cyanosilylation and hydroboration reactions, respectively. Hence, the diverse catalytic activity of **$1M^+$** is attributed to its amphoteric nature. In this regard, special attention is directed towards Lewis basicity of **$1M^+$**. Various intermediates which can be formed due to the nucleophilic attack of germanium lone pair in **$1M^+$** to various organic substrates, i.e., Me_3SiCN, RCHO and HBpin are depicted in Figure S73. However, geometry optimizations of all the intermediates remained unsuccessful at the R-BP86/def2-SVP level despite several attempts. Importantly, we were able to optimize only the intermediate **INT-37** at the R-M06-2X-D3/def2-TZVP level. Our calculations suggest that the formation of **INT-37** because of the nucleophilic attack of germanium lone pair in **$1M^+$** to the silicon center in Me_3SiCN is extremely endergonic by 29.5 kcal mol^{-1} and its generation also demands an activation barrier of 30.8 kcal mol^{-1} (Figure S74). The subsequent −CN transfer to the carbonyl carbon in the incoming substrate demands an energy barrier of 32.9 kcal mol^{-1}. Importantly, the favorable pathway, depicted in Figure S57, shows the rate-limiting energy barrier of 31.8 kcal mol^{-1} at the same level of theory. Hence, we can safely discard the Lewis basicity of germyliumylidene for cyanosilylation and hydroboration reactions.

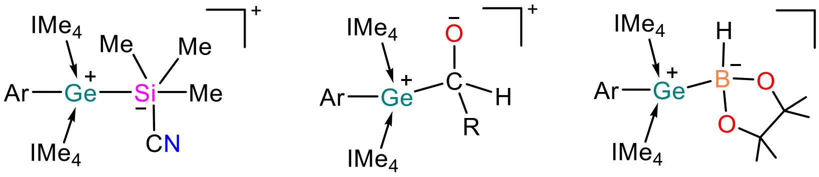

Figure S73. The intermediates formed due to the nucleophilic attack of germanium lone pair in **1M**$^+$ to various organic substrates. Ar = 2,6-dimethylphenyl; RCHO = Propanal.

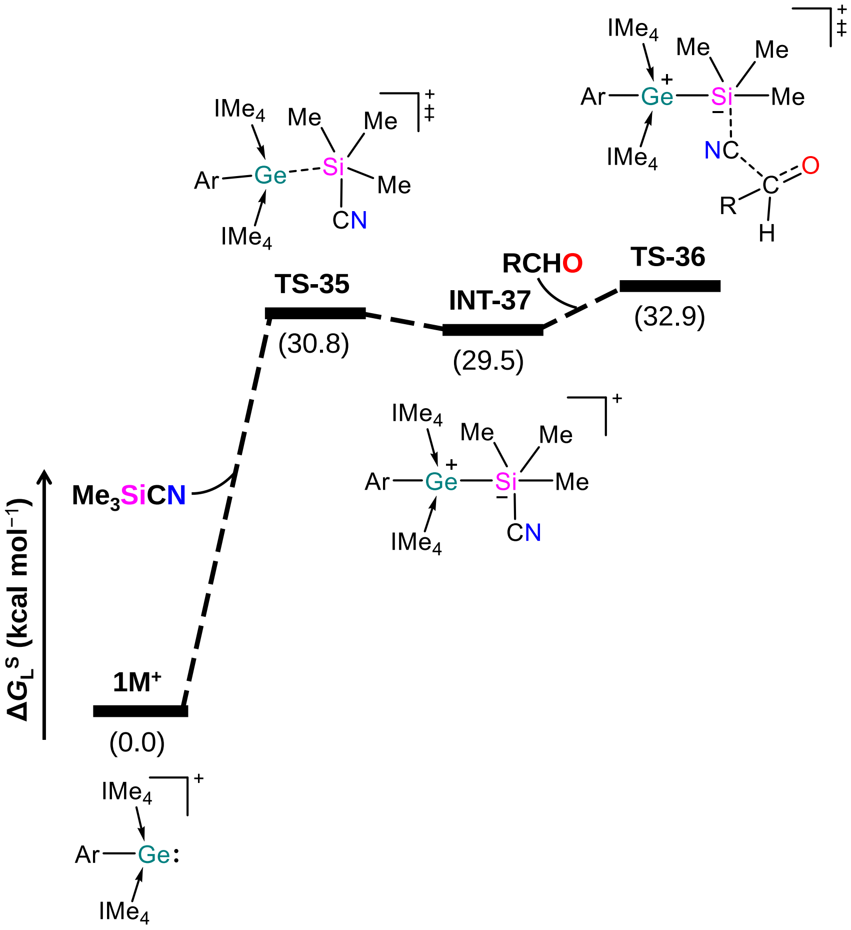

Figure S74. Energy profile for the nucleophilic attack of germanium lone pair in **1M**$^+$ to Me_3SiCN at the R-M06-2X-D3/def2-TZVP(SMD)//R-M06-2X-D3/def2-TZVP level. Ar = 2,6-dimethylphenyl.

Table S1. Absolute energies (in hartree) of all the intermediates and transition states. E_e = Electronic energy, H_{298} = gas-phase enthalpy at 298 K, G_{298} = gas-phase Gibbs free energy and S_{298} = gas-phase entropy.

Species	E_e	G_{298}	H_{298}	S_{298}	No. of imaginary frequency
**1M**$^+$	-3153.276937	-3152.842993	-3152.744214	207.899	0
**CO_2**	-188.4544419	-188.463803	-188.439462	51.23	0
**$PhSiH_3$**	-522.701801	-522.622688	-522.581769	86.121	0
**TS-1**	-3864.390898	-3863.843697	-3863.72149	257.205	1
**INT-1**	-3864.418583	-3863.867709	-3863.744182	259.985	0

**TS-2**	-3864.421153	-3863.866885	-3863.747617	251.02	1
**INT-2**	-3864.422076	-3863.869418	-3863.747641	256.3	0
**TS-3**	-3864.419576	-3863.869525	-3863.746309	259.331	1
**P**	-711.1673254	-711.077607	-711.028085	104.229	0
**Ph_2SiH_2**	-753.5934726	-753.441476	-753.387697	113.188	0
**TS-$1_{Ph_2SiH_2}$**	-4095.272788	-4094.650399	-4094.517416	279.886	1
**INT-$_{1Ph_2SiH_2}$**	-4095.313419	-4094.685038	-4094.552972	277.955	0
**Ph_3SiH**	-984.485261	-984.261198	-984.193883	141.675	0
**TS-1_{Ph_3SiH}**	-4326.15672	-4325.458024	-4325.314758	301.528	1
**INT-1_{Ph_3SiH}**	-4326.192909	-4325.489999	-4325.347056	300.849	0
**Et_2NH**	-213.6305782	-213.516472	-213.478303	80.334	0
**TS-4**	-924.7672472	-924.543784	-924.476239	142.16	1
**P1**	-326.900168	-326.779155	-326.736336	90.121	0
**$PhSiH_2OH$**	-597.9140406	-597.829909	-597.786504	91.354	0
**TS-11**	-849.5529219	-849.331133	-849.268743	131.312	1
**TS-13**	-4002.839197	-4002.156242	-4002.020613	285.455	1
**TS-12**	-4002.845591	-4002.174733	-4002.026476	312.034	1
**TS-5**	-924.7741729	-924.547035	-924.482195	136.466	1
**INT-3**	-924.8439551	-924.613224	-924.547136	139.094	0
**TS-6**	-1447.499015	-1447.171063	-1447.083286	184.742	1
**INT-4**	-1194.693464	-1194.522141	-1194.454654	142.038	0
**P2**	-252.904432	-252.765733	-252.724234	87.343	0
**TS-9**	-3341.687277	-3341.2446	-3341.139609	220.974	1
**INT-7**	-3341.746408	-3341.3039	-3341.196433	226.183	0
**TS-10**	-3864.386012	-3863.842968	-3863.71679	265.564	1
**INT-8**	-3864.466083	-3863.917609	-3863.790435	267.66	0
**IMe_4**	-383.1548132	-383.013452	-382.967314	97.106	0
**HBpin**	-411.5507017	-411.400246	-411.356085	92.945	0
**TS-26**	-3564.781726	-3564.175618	-3564.056268	251.193	1
**INT-24**	-3564.816706	-3564.218227	-3564.088719	272.573	0
**INT-25**	-3181.643099	-3181.201548	-3181.104784	203.657	0
**TS-27**	-3374.580454	-3374.066779	-3373.955269	234.693	1
**INT-26**	-3374.681033	-3374.15946	-3374.048763	232.98	0
**INT-20**	-2963.071248	-2962.729012	-2962.639484	188.428	0
**TS-28**	-3374.581625	-3374.064387	-3373.953806	232.738	1
**INT-27**	-3374.684062	-3374.163939	-3374.051431	236.792	0
**INT-28**	-3181.611411	-3181.170843	-3181.07269	206.58	0
**INT-29**	-3374.61578	-3374.104196	-3373.987191	246.257	0
**TS-29**	-3374.576572	-3374.059928	-3373.949891	231.592	1
**INT-30**	-3374.68261	-3374.162256	-3374.050086	236.082	0
**TS-30**	-3564.807805	-3564.202446	-3564.080605	256.436	1
**INT-31**	-3564.84653	-3564.238007	-3564.118015	252.544	0
**TS-31**	-3564.845608	-3564.240678	-3564.117828	258.56	1
**INT-33**	-793.9928177	-793.684953	-793.614946	147.342	0
**INT-32**	-2770.83807	-2770.563409	-2770.489576	155.394	0
**TS-32**	-2963.800756	-2963.449151	-2963.363963	179.293	1
**INT-34**	-2963.878357	-2963.523853	-2963.435683	185.57	0
**P^{HB}**	-604.6203173	-604.390941	-604.332056	123.935	0
**TS-33**	-576.1554932	-575.938233	-575.878969	124.732	1
**INT-35**	-576.1630443	-575.943766	-575.885664	122.288	0
**INT-21**	-794.7398677	-794.425396	-794.355565	146.972	0
**TS-23**	-3757.828902	-3757.149388	-3757.010793	291.698	1
**INT-22**	-3757.885813	-3757.203658	-3757.063242	295.53	0
**TS-24**	-3757.878079	-3757.190132	-3757.056385	281.496	1
**INT-23**	-3757.89005	-3757.200577	-3757.067481	280.125	0
**TS-25**	-3757.851235	-3757.163662	-3757.030475	280.317	1
**Me_3SiCN**	-501.8903403	-501.8083	-501.764112	93	0
**TS-14**	-3655.134871	-3654.603764	-3654.475563	269.822	1
**INT-9**	-3655.141171	-3654.60912	-3654.48096	269.736	0

**INT-10**	-3271.9703	-3271.600255	-3271.499806	211.411	0
**INT-11**	-3543.547048	-3543.055402	-3542.929999	263.933	0
**TS-15**	-3543.506802	-3543.011089	-3542.890932	252.892	1
**INT-12**	-3543.569483	-3543.071159	-3542.951445	251.959	0
**INT-13**	-3926.7389	-3926.077832	-3925.931128	308.765	0
**TS-16**	-3926.734371	-3926.07162	-3925.9273	303.746	1
**P^{CS}**	-773.4916345	-773.281192	-773.21764	133.756	0
**TS-17**	-654.722088	-654.453195	-654.38888	135.361	1
**INT-14**	-654.7312207	-654.459383	-654.397322	130.617	0
**INT-15**	-3926.750794	-3926.081317	-3925.94151	294.248	0
**TS-18**	-3926.736319	-3926.068832	-3925.929015	294.27	1
**INT-16**	-3926.776406	-3926.113427	-3925.966954	308.278	0
**TS-19**	-3926.696004	-3926.040974	-3925.89005	317.646	1
**INT-17**	-3926.741502	-3926.08297	-3925.933817	313.919	0
**INT-18**	-3041.640506	-3041.24688	-3041.151365	201.028	0
**TS-21**	-885.026629	-884.781466	-884.711661	146.918	1
**INT-19**	-885.0309253	-884.785582	-884.714723	149.136	0

Table S2. Cartesian coordinates (Å) of the reactants, intermediates and transition states at the BP86/def2-SVP level.

**1M$^+$**			
Ge	0.00223	-0.31488	-0.99420
N	-1.65846	1.89680	0.62279
N	2.66215	0.08262	0.67937
N	2.23330	1.67816	-0.73147
N	-2.80126	0.56569	-0.65575
C	-0.20277	-2.11051	-0.04462
C	1.67685	0.56713	-0.14173
C	-1.53889	0.78634	-0.16989
C	-0.85896	-2.36650	1.19969
C	0.26775	-3.21866	-0.82084
C	-1.04995	-3.70217	1.62370
H	-1.55253	-3.89248	2.58649
C	-0.60067	2.48698	1.43290
H	0.30579	1.86286	1.33907
H	-0.90736	2.52280	2.49745
H	-0.37231	3.51953	1.09835
C	0.06008	-4.53611	-0.35854
H	0.42920	-5.38141	-0.96210
C	-0.60357	-4.78168	0.85099
H	-0.76220	-5.81502	1.19772
C	-2.98181	2.36302	0.64448
C	3.53605	1.89923	-0.27604
C	2.55770	-1.08663	1.55087
H	2.61252	-0.78041	2.61510
H	3.38440	-1.79288	1.33986
H	1.59804	-1.59667	1.35456
C	3.81180	0.87939	0.61762
C	-3.70980	1.51079	-0.16434
C	1.55860	2.50686	-1.72527
H	0.78621	1.88638	-2.22816
H	2.28676	2.85729	-2.48051
H	1.07495	3.38916	-1.25815
C	-3.40368	3.56155	1.43054
H	-3.23106	3.43083	2.52040
H	-4.48397	3.75266	1.28931
H	-2.86135	4.47871	1.11579
C	-3.15262	-0.54058	-1.54351
H	-2.29723	-0.73623	-2.22426
H	-4.03735	-0.26938	-2.14732

H	-3.36622	-1.46375	-0.96802
C	4.38091	3.04151	-0.73881
H	4.62162	2.97166	-1.82153
H	5.34131	3.05495	-0.19023
H	3.88590	4.02109	-0.56942
C	5.04312	0.58489	1.41057
H	4.84050	0.55907	2.50226
H	5.81111	1.36071	1.23246
H	5.48977	-0.39396	1.13369
C	-5.16279	1.50962	-0.51325
H	-5.33331	1.67267	-1.59913
H	-5.68591	2.32131	0.02621
H	-5.65601	0.55420	-0.23565
C	1.00890	-3.03345	-2.13323
H	0.39260	-2.50559	-2.89181
H	1.93552	-2.43190	-2.01124
H	1.30745	-4.01118	-2.56047
C	-1.34348	-1.27071	2.12909
H	-0.62446	-0.42877	2.18792
H	-2.31603	-0.84202	1.80411
H	-1.49426	-1.66298	3.15485
**CO_2**			
C	0.00000	0.00000	0.00000
O	0.00000	0.00000	1.17531
O	0.00000	0.00000	-1.17531
**$PhSiH_3$**			
Si	1.14924	0.88025	-1.48181
H	1.77350	2.17061	-1.02546
C	-0.73629	1.00465	-1.38897
C	-1.39295	2.24868	-1.55137
C	-1.53286	-0.14805	-1.18367
C	-2.79538	2.33809	-1.51438
H	-0.80168	3.16731	-1.70509
C	-2.93541	-0.06234	-1.14612
H	-1.05279	-1.13173	-1.04553
C	-3.56926	1.18175	-1.31224
H	-3.28663	3.31638	-1.64077
H	-3.53682	-0.97122	-0.98300
H	-4.66852	1.25074	-1.28056
H	1.61538	0.61206	-2.88933
H	1.63627	-0.24634	-0.61185
**TS-1**			
Si	-2.24316	-0.05681	-1.25779
H	-2.17539	-1.52588	-0.47965
O	-2.68095	-3.62910	-0.24998
C	-1.72342	-2.90693	-0.32190
O	-0.50368	-2.80722	-0.32177
Ge	0.20614	-0.24529	0.05841
C	2.02599	-0.89315	-0.69448
C	0.06780	-0.76423	2.00857
N	3.29200	-0.62550	-0.25150
N	2.19417	-1.62616	-1.83363
C	0.78788	-1.82796	2.63829
C	-0.96717	-0.09735	2.74007
C	4.25188	-1.20175	-1.08960
C	3.62830	0.13892	0.94553
C	3.55064	-1.84371	-2.09551
C	1.13246	-2.14236	-2.69961
C	0.53034	-2.11802	3.99812
C	-1.18855	-0.42320	4.09574
H	3.95011	1.16664	0.67970

H	4.45024	-0.36221	1.49155
H	2.74391	0.18579	1.60444
H	0.17436	-1.67364	-2.41881
H	1.03138	-3.23761	-2.57511
H	1.37136	-1.90739	-3.75456
H	1.09371	-2.93297	4.48113
C	-0.43341	-1.41577	4.73165
H	-1.98262	0.10315	4.65018
H	-0.61563	-1.66250	5.78931
H	-1.91122	1.42779	-1.16368
C	1.78885	-2.71752	1.92675
H	1.41688	-3.02379	0.93113
H	2.78082	-2.23665	1.79696
H	1.96382	-3.63943	2.51551
C	-1.89931	0.91970	2.11005
H	-1.40325	1.61564	1.40318
H	-2.71059	0.41385	1.53936
H	-2.40274	1.53373	2.88321
C	5.72165	-1.08978	-0.84557
H	6.02500	-1.57796	0.10545
H	6.28347	-1.58302	-1.66068
H	6.05863	-0.03254	-0.80157
C	4.03258	-2.64087	-3.26318
H	3.62931	-3.67527	-3.24938
H	3.73978	-2.18335	-4.23233
H	5.13609	-2.71331	-3.25062
C	-4.14128	0.02786	-1.13986
C	-4.78027	1.28763	-1.24365
C	-4.94802	-1.12856	-0.99436
C	-6.18197	1.39138	-1.22562
H	-4.17180	2.20344	-1.34318
C	-6.34956	-1.02497	-0.97553
H	-4.48044	-2.12100	-0.88864
C	-6.96816	0.23306	-1.09282
H	-6.66301	2.37901	-1.31165
H	-6.96337	-1.93310	-0.86474
H	-8.06692	0.31168	-1.07444
C	0.86962	1.77189	0.06675
N	0.91244	2.47689	-1.10736
N	1.11061	2.70685	1.03845
C	1.18132	3.83087	-0.88457
C	0.74646	1.90616	-2.44138
C	1.30324	3.97934	0.48462
C	1.20557	2.46101	2.47644
H	0.09492	2.55704	-3.05347
H	0.26397	0.91488	-2.35125
H	1.72802	1.79548	-2.94652
H	1.02679	1.39088	2.68023
H	0.44273	3.05338	3.01898
H	2.20871	2.75288	2.84705
C	1.57152	5.19188	1.31477
H	1.69975	6.07995	0.66806
H	0.73581	5.40812	2.01389
H	2.49429	5.08733	1.92420
C	1.27989	4.83393	-1.98724
H	2.05464	4.55917	-2.73388
H	0.31769	4.94865	-2.53110
H	1.54802	5.82685	-1.58030
H	-1.76639	-0.50818	-2.62058

INT-1

Si	-1.54774	0.40910	-1.88133
H	-2.48711	-1.81983	1.76525

O	-2.66972	-3.53069	0.63688
C	-2.18955	-2.43118	0.86511
O	-1.28769	-1.85492	0.07971
Ge	-0.17507	-0.17363	0.06159
C	1.37729	-1.50501	-0.19816
C	-0.60647	0.36297	1.94429
N	2.59673	-1.47680	0.42112
N	1.38807	-2.63368	-0.97102
C	-0.33087	-0.47617	3.06535
C	-1.33453	1.57957	2.13665
C	3.37067	-2.58013	0.05366
C	3.05371	-0.47909	1.38438
C	2.60115	-3.31606	-0.83027
C	0.29833	-3.14652	-1.81507
C	-0.72433	-0.04706	4.35559
C	-1.71750	1.95860	3.43971
H	3.75932	0.23219	0.90994
H	3.56573	-0.98664	2.22370
H	2.18738	0.07440	1.78247
H	-0.25931	-2.30745	-2.26220
H	-0.41815	-3.73497	-1.21033
H	0.74040	-3.76092	-2.61983
H	-0.50424	-0.69476	5.22011
C	-1.40384	1.15966	4.54852
H	-2.27979	2.89597	3.58052
H	-1.70737	1.47059	5.56044
H	-1.16491	1.78942	-2.35077
C	0.33886	-1.83490	2.98339
H	0.39865	-2.22936	1.95399
H	1.36190	-1.81362	3.41791
H	-0.23331	-2.57635	3.57785
C	-1.74874	2.47437	0.98828
H	-0.94462	2.61830	0.23699
H	-2.62606	2.05010	0.45358
H	-2.05198	3.47636	1.35132
C	4.74506	-2.82514	0.58609
H	4.74346	-2.99765	1.68399
H	5.18228	-3.72505	0.11488
H	5.43079	-1.97602	0.38156
C	2.90873	-4.60084	-1.52887
H	2.17399	-5.39381	-1.27676
H	2.91577	-4.48811	-2.63403
H	3.90858	-4.96615	-1.22865
C	-3.41401	0.33219	-1.64556
C	-4.17606	1.52574	-1.72950
C	-4.09691	-0.89401	-1.44619
C	-5.57635	1.49574	-1.61095
H	-3.67447	2.49205	-1.90627
C	-5.49649	-0.92035	-1.32709
H	-3.54079	-1.84225	-1.38738
C	-6.23770	0.27239	-1.40573
H	-6.15303	2.43140	-1.68476
H	-6.00983	-1.88223	-1.17138
H	-7.33497	0.24757	-1.31225
C	1.41040	1.52673	-0.41981
N	1.94362	1.68456	-1.67476
N	1.87787	2.61950	0.26684
C	2.71023	2.85284	-1.78286
C	1.78184	0.76892	-2.79963
C	2.66876	3.45116	-0.53997
C	1.66429	2.91057	1.68257
H	1.22147	1.25208	-3.62443

H	1.22744	-0.12533	-2.47209
H	2.77293	0.44970	-3.17977
H	1.06722	2.10478	2.14073
H	1.11603	3.86566	1.80625
H	2.63861	2.99555	2.20474
C	3.28769	4.71770	-0.04474
H	3.85373	5.21214	-0.85649
H	2.52444	5.43977	0.31573
H	3.99566	4.54054	0.79297
C	3.38605	3.27155	-3.04800
H	4.13541	2.52636	-3.39131
H	2.66189	3.41924	-3.87732
H	3.91877	4.22972	-2.90009
H	-1.12441	-0.56032	-2.95623
	**TS-2**		
Si	-1.69491	-0.00906	-1.82210
H	-2.50659	-3.45899	-1.46941
O	-0.73965	-4.08681	-0.62952
C	-1.54024	-3.20501	-0.94410
O	-1.37268	-1.91978	-0.72764
Ge	0.01473	-0.06068	-0.06517
C	1.75361	-1.12842	-0.25738
C	-0.71356	-0.22901	1.78711
N	2.83453	-1.04126	0.57847
N	2.10916	-2.02922	-1.21548
C	-0.47705	-1.36701	2.62153
C	-1.63509	0.78083	2.20653
C	3.85615	-1.89879	0.16337
C	2.93551	-0.19896	1.76644
C	3.39015	-2.53194	-0.97579
C	1.29173	-2.49577	-2.33657
C	-1.09244	-1.40742	3.89387
C	-2.21980	0.69399	3.48886
H	3.50168	0.72872	1.54511
H	3.45748	-0.75237	2.56943
H	1.92241	0.05527	2.12510
H	0.42908	-1.82232	-2.46957
H	0.90010	-3.50663	-2.10719
H	1.90473	-2.50648	-3.25749
H	-0.90842	-2.28031	4.54092
C	-1.93983	-0.38412	4.33682
H	-2.92330	1.47923	3.81049
H	-2.40405	-0.44181	5.33389
H	-1.49805	1.42837	-2.32864
C	0.35853	-2.56604	2.21959
H	0.16650	-2.89823	1.17750
H	1.44879	-2.38526	2.33153
H	0.12212	-3.42544	2.87733
C	-2.09126	1.91515	1.31029
H	-1.33454	2.24084	0.56821
H	-2.98357	1.60268	0.72603
H	-2.38781	2.80416	1.90278
C	5.15393	-2.03675	0.89051
H	5.01684	-2.44445	1.91513
H	5.82126	-2.73266	0.34873
H	5.68794	-1.06752	0.98268
C	4.02475	-3.57159	-1.83973
H	3.40946	-4.49460	-1.88344
H	4.17070	-3.21813	-2.88267
H	5.01799	-3.84916	-1.44045
C	-3.54563	0.03227	-1.40830
C	-4.31846	1.03822	-2.04331

C	-4.20681	-0.87124	-0.54244
C	-5.70671	1.12166	-1.84004
H	-3.82823	1.76612	-2.71149
C	-5.59282	-0.77938	-0.32724
H	-3.62225	-1.64633	-0.02718
C	-6.34668	0.21258	-0.97916
H	-6.29049	1.90224	-2.35363
H	-6.08814	-1.49006	0.35353
H	-7.43387	0.27944	-0.81411
C	1.08311	1.79081	-0.16329
N	1.54896	2.26363	-1.36095
N	1.34535	2.79527	0.72952
C	2.09319	3.54501	-1.23019
C	1.53337	1.54783	-2.63518
C	1.96207	3.88588	0.10308
C	1.05603	2.78787	2.16272
H	1.07647	2.17925	-3.42030
H	0.92588	0.63172	-2.53708
H	2.56458	1.27514	-2.93806
H	0.54842	1.84680	2.43612
H	0.38868	3.63308	2.42097
H	1.99542	2.89016	2.74227
C	2.34702	5.12989	0.83484
H	2.83052	5.84685	0.14531
H	1.46535	5.63940	1.27872
H	3.06251	4.92660	1.65969
C	2.66484	4.30617	-2.38126
H	3.49335	3.75610	-2.87553
H	1.89874	4.52176	-3.15643
H	3.06895	5.27661	-2.03756
H	-1.26239	-0.78756	-3.03947

INT-2

Si	-1.75931	-0.15696	-1.76863
H	-2.87351	-3.12281	-1.81740
O	-1.33130	-4.12311	-0.89598
C	-1.93457	-3.09815	-1.19434
O	-1.54711	-1.88374	-0.83275
Ge	0.08070	0.00046	-0.07858
C	1.75992	-1.17470	-0.19906
C	-0.65859	-0.14109	1.77784
N	2.84602	-1.13064	0.63344
N	2.04824	-2.13934	-1.11841
C	-0.51263	-1.30842	2.59221
C	-1.51761	0.91794	2.20868
C	3.80432	-2.07317	0.25451
C	2.99011	-0.26490	1.79972
C	3.29142	-2.72090	-0.85694
C	1.17627	-2.60318	-2.20103
C	-1.12075	-1.32235	3.86796
C	-2.09646	0.85964	3.49558
H	3.59874	0.62993	1.55703
H	3.48642	-0.82274	2.61573
H	1.98865	0.04221	2.15096
H	0.47614	-1.79860	-2.48394
H	0.58240	-3.47624	-1.85739
H	1.79693	-2.87305	-3.07486
H	-1.00347	-2.21766	4.49984
C	-1.88348	-0.24260	4.33198
H	-2.74660	1.68536	3.82809
H	-2.34176	-0.27751	5.33282
H	-1.50597	1.30189	-2.25102
C	0.19997	-2.57091	2.14715

H	-0.09143	-2.87239	1.11950
H	1.30626	-2.48329	2.18269
H	-0.06649	-3.41129	2.81774
C	-1.92968	2.06773	1.31065
H	-1.17466	2.34657	0.54820
H	-2.84666	1.79193	0.74560
H	-2.17160	2.97663	1.89780
C	5.09412	-2.26710	0.98333
H	4.93645	-2.61972	2.02527
H	5.71055	-3.02878	0.47054
H	5.69194	-1.33256	1.03130
C	3.85556	-3.83719	-1.67358
H	3.17944	-4.71755	-1.68427
H	4.03229	-3.53762	-2.72864
H	4.82543	-4.16517	-1.25544
C	-3.59003	0.07918	-1.30478
C	-4.30335	1.11582	-1.95817
C	-4.28762	-0.71617	-0.36336
C	-5.66883	1.33061	-1.70557
H	-3.77897	1.76443	-2.68057
C	-5.64906	-0.49043	-0.09314
H	-3.75542	-1.51499	0.17383
C	-6.34436	0.52778	-0.76867
H	-6.20754	2.13095	-2.23786
H	-6.17115	-1.11692	0.64777
H	-7.41314	0.69859	-0.56287
C	1.17333	1.78399	-0.23389
N	1.62010	2.20631	-1.45633
N	1.53244	2.77652	0.63680
C	2.25555	3.44777	-1.36252
C	1.46060	1.45458	-2.70418
C	2.19630	3.81308	-0.02874
C	1.27442	2.81315	2.07557
H	1.71778	2.10738	-3.55620
H	0.40090	1.14320	-2.81042
H	2.13006	0.57177	-2.71773
H	0.71763	1.90918	2.37776
H	0.66329	3.70137	2.32877
H	2.23007	2.87130	2.63390
C	2.68661	5.03874	0.67054
H	3.19287	5.71310	-0.04496
H	1.85555	5.61130	1.13476
H	3.41450	4.79945	1.47462
C	2.83974	4.17160	-2.53239
H	3.62790	3.57881	-3.04260
H	2.06842	4.43326	-3.28775
H	3.30402	5.11832	-2.19842
H	-1.40605	-0.83253	-3.07764

TS-3

Si	-1.88696	-0.50028	-2.04973
H	-2.53251	-3.40719	-2.22188
O	-1.16226	-4.24611	-0.93685
C	-1.74756	-3.29403	-1.42424
O	-1.49850	-2.02726	-1.06337
Ge	0.06772	-0.01363	-0.16550
C	1.82623	-1.11863	-0.08357
C	-0.74621	-0.13176	1.67773
N	2.84174	-1.04032	0.83034
N	2.22675	-2.06989	-0.97687
C	-0.64236	-1.29569	2.50460
C	-1.63609	0.92010	2.06251
C	3.86478	-1.94324	0.52754

C	2.85713	-0.17733	2.00684
C	3.46847	-2.60483	-0.62178
C	1.46511	-2.55404	-2.12840
C	-1.31793	-1.31239	3.74597
C	-2.28314	0.86210	3.31677
H	3.47795	0.72425	1.82843
H	3.27295	-0.73142	2.86942
H	1.82044	0.11736	2.24896
H	0.68416	-1.81668	-2.37789
H	0.97085	-3.51512	-1.88155
H	2.14196	-2.68302	-2.99355
H	-1.22836	-2.20572	4.38543
C	-2.11026	-0.23636	4.16707
H	-2.95357	1.68601	3.61195
H	-2.62079	-0.27124	5.14233
H	-1.43422	0.83945	-2.65091
C	0.10432	-2.55452	2.10564
H	-0.12037	-2.86409	1.06413
H	1.20587	-2.45852	2.20658
H	-0.19583	-3.39611	2.76058
C	-2.00812	2.06862	1.14481
H	-2.90775	1.79761	0.55111
H	-2.26151	2.98316	1.71860
H	-1.22389	2.33539	0.40871
C	5.09845	-2.08941	1.35764
H	4.87107	-2.45286	2.38283
H	5.78541	-2.82267	0.89537
H	5.65122	-1.13140	1.45646
C	4.13687	-3.69182	-1.39834
H	3.50175	-4.60027	-1.46191
H	4.37741	-3.37887	-2.43687
H	5.08665	-3.98241	-0.91189
C	-3.59104	-0.05081	-1.36633
C	-4.25256	1.09146	-1.88359
C	-4.26246	-0.82957	-0.39325
C	-5.54744	1.43328	-1.45826
H	-3.74780	1.72085	-2.63651
C	-5.55361	-0.48302	0.04251
H	-3.76679	-1.71347	0.03687
C	-6.19954	0.64597	-0.49140
H	-6.05043	2.31768	-1.88111
H	-6.05951	-1.10001	0.80220
H	-7.21343	0.91389	-0.15377
C	1.14777	1.79876	-0.25908
N	1.64128	2.22200	-1.46525
N	1.43647	2.81697	0.61058
C	2.24281	3.47965	-1.35898
C	1.58579	1.45571	-2.70988
C	2.10668	3.86220	-0.03687
C	1.10930	2.86741	2.03432
H	1.53527	2.14920	-3.56866
H	0.66781	0.83666	-2.71016
H	2.48186	0.81115	-2.81952
H	0.55977	1.95434	2.32154
H	0.46633	3.74399	2.24569
H	2.03532	2.95560	2.63700
C	2.53048	5.11021	0.66632
H	3.05715	5.78583	-0.03323
H	1.66307	5.66927	1.07759
H	3.22065	4.90131	1.51112
C	2.86881	4.19194	-2.51389
H	3.67437	3.59272	-2.98810

H	2.12637	4.44130	-3.30215
H	3.31903	5.14403	-2.17590
H	-1.91321	-1.28407	-3.34967
**P**			
C	0.17253	-1.08891	-0.26765
O	-0.43874	-2.00460	0.22060
O	1.02414	-0.29967	0.43752
H	0.09691	-0.79048	-1.35336
Si	1.98195	1.00123	-0.15900
H	1.52612	1.28433	-1.56505
C	3.80136	0.55228	-0.13686
C	4.43075	0.01563	-1.28712
C	4.57728	0.72929	1.03527
C	5.79024	-0.33864	-1.26536
H	3.85399	-0.12606	-2.21689
C	5.93651	0.37506	1.05907
H	4.11522	1.15067	1.94390
C	6.54353	-0.15980	-0.09139
H	6.26508	-0.75401	-2.16856
H	6.52604	0.51821	1.97868
H	7.60992	-0.43634	-0.07367
H	1.69871	2.14724	0.76049
**Ph_2SiH_2**			
Si	-2.45030	0.19469	-1.86835
C	-4.28672	0.06669	-1.42006
C	-5.21445	1.04689	-1.85174
C	-4.77642	-1.02601	-0.66443
C	-6.58112	0.93647	-1.54438
H	-4.86437	1.91583	-2.43454
C	-6.14355	-1.14011	-0.35467
H	-4.07791	-1.80204	-0.30827
C	-7.04834	-0.15880	-0.79527
H	-7.28618	1.70993	-1.88961
H	-6.50340	-1.99807	0.23603
H	-8.11971	-0.24547	-0.55242
H	-2.21902	-0.15357	-3.31696
C	-1.77109	1.94146	-1.59037
C	-0.94522	2.56319	-2.55792
C	-2.05320	2.65417	-0.39860
C	-0.41507	3.84794	-2.34254
H	-0.71275	2.03650	-3.49895
C	-1.52540	3.93789	-0.17963
H	-2.70084	2.20208	0.37174
C	-0.70400	4.53701	-1.15197
H	0.22443	4.31417	-3.10947
H	-1.75713	4.47532	0.75418
H	-0.29086	5.54433	-0.98183
H	-1.71009	-0.81610	-1.02953
**TS-1$_{Ph_2SiH_2}$**			
Si	-2.25845	-0.03169	-1.28283
H	-1.82673	-1.63742	-0.94150
O	-2.08949	-3.67785	-1.58769
C	-1.25760	-2.81169	-1.48694
O	-0.07796	-2.51867	-1.66674
Ge	0.41993	-0.47393	-0.01216
C	2.25381	-0.63453	-0.97863
C	0.27410	-1.96303	1.36670
N	3.51850	-0.63662	-0.45682
N	2.42755	-0.66230	-2.33215
C	1.01016	-3.19126	1.36526
C	-0.78691	-1.79631	2.31516

C	4.48215	-0.68978	-1.46963
C	3.85393	-0.60825	0.96347
C	3.78512	-0.71021	-2.66481
C	1.37054	-0.64107	-3.34414
C	0.72147	-4.16493	2.35018
C	-1.04055	-2.79877	3.27586
H	4.16927	0.40891	1.27418
H	4.68099	-1.31506	1.16670
H	2.97184	-0.91720	1.55033
H	0.40635	-0.41715	-2.85736
H	1.28332	-1.63128	-3.83052
H	1.60280	0.13113	-4.10277
H	1.29718	-5.10480	2.34724
C	-0.28318	-3.97533	3.30579
H	-1.85706	-2.65086	4.00141
H	-0.48943	-4.75274	4.05800
C	2.07483	-3.55533	0.34863
H	1.76919	-3.27157	-0.67523
H	3.05590	-3.08483	0.56848
H	2.24673	-4.64970	0.35512
C	-1.69754	-0.58464	2.32389
H	-1.16587	0.36874	2.12801
H	-2.49371	-0.67640	1.55147
H	-2.21736	-0.48215	3.29738
C	5.95108	-0.72698	-1.19845
H	6.25044	-1.64452	-0.64758
H	6.51586	-0.71732	-2.14944
H	6.28916	0.14657	-0.60189
C	4.27105	-0.78064	-4.07552
H	3.87176	-1.67222	-4.60330
H	3.97642	0.11341	-4.66552
H	5.37488	-0.84538	-4.09992
C	-4.05970	-0.34995	-0.74196
C	-4.96510	0.72723	-0.55873
C	-4.54966	-1.67168	-0.57385
C	-6.31076	0.49263	-0.22772
H	-4.62156	1.76592	-0.68466
C	-5.89543	-1.90349	-0.24249
H	-3.88134	-2.53538	-0.71893
C	-6.77849	-0.82321	-0.06708
H	-6.99799	1.34375	-0.09617
H	-6.25635	-2.93748	-0.12320
H	-7.83291	-1.00701	0.19413
C	1.16440	1.18595	1.10757
N	1.37784	2.38908	0.48974
N	1.37107	1.44269	2.43876
C	1.71085	3.38895	1.40753
C	1.30399	2.62330	-0.94840
C	1.70278	2.78765	2.65214
C	1.33885	0.47142	3.53080
H	0.75308	3.56154	-1.14915
H	0.74148	1.79595	-1.41678
H	2.32028	2.68642	-1.38878
H	1.13842	-0.53481	3.12393
H	0.53993	0.72675	4.25485
H	2.31064	0.47104	4.06387
C	1.97062	3.35065	4.00978
H	2.20128	4.43015	3.93915
H	1.09517	3.24064	4.68450
H	2.83375	2.85819	4.50654
C	1.98852	4.80056	1.00567
H	2.83228	4.87139	0.28694

H	1.10314	5.27186	0.52826
H	2.25368	5.40825	1.89105
H	-1.99629	-0.24163	-2.75499
C	-2.03303	1.86826	-1.11961
C	-1.91464	2.61090	-2.32139
C	-2.09950	2.59304	0.09591
C	-1.86423	4.01774	-2.31303
H	-1.88324	2.08139	-3.28911
C	-2.04402	3.99841	0.11216
H	-2.22837	2.05668	1.05016
C	-1.92549	4.71641	-1.09394
H	-1.79303	4.57035	-3.26384
H	-2.11931	4.53924	1.06960
H	-1.90399	5.81801	-1.08435

TS-1$_{Ph_3SiH}$

Si	-2.29305	-0.05574	-0.26647
H	-1.76359	-1.26303	0.96571
O	-2.07600	-2.94154	2.17903
C	-1.31938	-2.37351	1.41985
O	-0.18677	-2.53379	0.90902
Ge	0.71898	-0.27361	0.44919
C	2.23398	-1.43745	-0.38739
C	1.04543	-0.21163	2.46382
N	3.58738	-1.34642	-0.21225
N	2.06287	-2.42283	-1.31463
C	1.70993	-1.22589	3.22877
C	0.42802	0.87895	3.15700
C	4.26337	-2.28134	-1.00449
C	4.27157	-0.41972	0.68446
C	3.28975	-2.97019	-1.70489
C	0.78604	-2.87783	-1.85932
C	1.79874	-1.07594	4.63323
C	0.54070	0.98159	4.56047
H	4.69146	0.43678	0.11847
H	5.09712	-0.94346	1.20290
H	3.55659	-0.05209	1.44099
H	-0.00509	-2.17042	-1.56062
H	0.52734	-3.87478	-1.45360
H	0.84852	-2.92886	-2.96346
H	2.31619	-1.85712	5.21364
C	1.23245	0.01586	5.29993
H	0.06217	1.83107	5.07473
H	1.31267	0.10221	6.39492
C	2.32023	-2.49048	2.65413
H	1.69650	-2.91056	1.84465
H	3.34873	-2.32984	2.26670
H	2.40557	-3.26013	3.44648
C	-0.39399	1.93952	2.45502
H	0.00617	2.21776	1.45931
H	-1.43600	1.58998	2.29419
H	-0.45817	2.86468	3.06226
C	5.75023	-2.42892	-1.00398
H	6.13694	-2.75187	-0.01350
H	6.05875	-3.19382	-1.74104
H	6.26515	-1.48203	-1.27139
C	3.40171	-4.09011	-2.68707
H	2.84918	-4.99163	-2.34783
H	3.00339	-3.81343	-3.68666
H	4.46066	-4.37994	-2.82096
C	-3.44522	0.74692	1.02092
C	-4.04584	2.01251	0.79632
C	-3.82607	0.02067	2.18063

C	-4.98670	2.53696	1.70044
H	-3.79134	2.59625	-0.10217
C	-4.76556	0.54837	3.08202
H	-3.40001	-0.97604	2.38291
C	-5.34488	1.80859	2.84757
H	-5.44510	3.51935	1.50330
H	-5.04963	-0.03368	3.97309
H	-6.08078	2.22013	3.55664
C	1.82959	1.47106	-0.26767
N	1.87610	1.73144	-1.61117
N	2.47809	2.53604	0.30471
C	2.53516	2.93307	-1.88672
C	1.34049	0.86730	-2.65678
C	2.91644	3.45045	-0.66372
C	2.77317	2.72087	1.72401
H	0.79904	1.47490	-3.40583
H	0.61678	0.16580	-2.20486
H	2.15476	0.30190	-3.15506
H	2.41678	1.84632	2.29458
H	2.26735	3.62729	2.11150
H	3.86569	2.83816	1.87015
C	3.64281	4.71017	-0.32048
H	3.87743	5.28052	-1.23882
H	3.03932	5.37144	0.33706
H	4.60303	4.51354	0.20272
C	2.72255	3.45934	-3.27211
H	3.28777	2.75127	-3.91464
H	1.74943	3.65765	-3.76976
H	3.28648	4.41073	-3.24986
C	-1.89473	1.26933	-1.60035
C	-2.12385	0.99327	-2.97278
C	-1.44923	2.57350	-1.26321
C	-1.93587	1.97796	-3.96068
H	-2.47838	-0.00450	-3.27688
C	-1.26211	3.56224	-2.24533
H	-1.26848	2.83525	-0.20844
C	-1.50832	3.26835	-3.60029
H	-2.14182	1.73894	-5.01658
H	-0.94223	4.57458	-1.94969
H	-1.38427	4.04766	-4.36942
C	-3.24937	-1.43083	-1.19324
C	-2.86741	-2.78562	-1.32314
C	-4.49163	-1.03776	-1.75787
C	-3.68168	-3.71139	-2.00081
H	-1.92787	-3.14189	-0.87507
C	-5.30994	-1.96104	-2.43004
H	-4.83885	0.00462	-1.66123
C	-4.90442	-3.30148	-2.55708
H	-3.36389	-4.76326	-2.08051
H	-6.27446	-1.63227	-2.84876
H	-5.54732	-4.02741	-3.07956
**INT-1$_{Ph_2SiH_2}$**			
Si	-1.80030	0.27260	-1.34700
H	-0.33742	-3.52430	-3.01976
O	1.57099	-2.86174	-2.64680
C	0.35663	-2.85491	-2.43286
O	-0.29119	-2.12710	-1.55631
Ge	0.17880	-0.62838	-0.12245
C	2.09858	-0.48607	-0.85783
C	-0.16729	-1.97454	1.33417
N	3.25470	-0.65647	-0.15038
N	2.49141	-0.16771	-2.12185

C	0.52433	-3.22076	1.41839
C	-1.23833	-1.70691	2.24752
C	4.37264	-0.48132	-0.97256
C	3.36371	-0.97313	1.26933
C	3.88353	-0.17532	-2.22883
C	1.62708	0.02640	-3.28181
C	0.16959	-4.13509	2.43958
C	-1.56605	-2.65877	3.23548
H	3.75934	-0.10237	1.83048
H	4.04676	-1.83223	1.41031
H	2.37182	-1.24853	1.66530
H	0.57009	-0.01274	-2.96886
H	1.80011	-0.79448	-4.00320
H	1.83958	1.00572	-3.75438
H	0.71779	-5.08940	2.50566
C	-0.86320	-3.86590	3.34154
H	-2.39262	-2.44121	3.93135
H	-1.12663	-4.59698	4.12200
C	1.61124	-3.68740	0.47294
H	1.90348	-2.96288	-0.30576
H	2.51635	-3.99898	1.03661
H	1.26757	-4.58952	-0.07583
C	-2.07240	-0.44492	2.20666
H	-1.48538	0.44869	1.91457
H	-2.90254	-0.54543	1.47554
H	-2.53339	-0.23955	3.19353
C	5.77391	-0.64318	-0.48189
H	5.97697	-1.68202	-0.14375
H	6.49087	-0.41665	-1.29306
H	6.00404	0.03604	0.36617
C	4.59365	0.07060	-3.51883
H	4.34421	-0.70645	-4.27223
H	4.33742	1.05754	-3.95858
H	5.68882	0.04756	-3.36613
C	-3.36751	-0.74292	-1.00999
C	-4.59402	-0.05567	-0.81668
C	-3.38175	-2.16048	-1.01167
C	-5.79464	-0.76360	-0.63201
H	-4.61920	1.04593	-0.81746
C	-4.58368	-2.86352	-0.82463
H	-2.43726	-2.70527	-1.16264
C	-5.79171	-2.16904	-0.63377
H	-6.73757	-0.21187	-0.48982
H	-4.57641	-3.96523	-0.82967
H	-6.73243	-2.72371	-0.48867
C	0.80821	1.18147	1.16970
N	1.06163	2.41000	0.61516
N	1.01476	1.38207	2.51250
C	1.40275	3.36475	1.58028
C	1.05052	2.73460	-0.80668
C	1.37195	2.70875	2.79393
C	0.92121	0.38159	3.57349
H	0.41302	3.61952	-0.99337
H	0.62875	1.89090	-1.37444
H	2.08076	2.93999	-1.16323
H	0.69752	-0.60581	3.13719
H	0.11139	0.64311	4.28331
H	1.87634	0.33319	4.13363
C	1.63601	3.20491	4.17831
H	1.88735	4.28204	4.15850
H	0.75266	3.08169	4.84040
H	2.48523	2.67497	4.66039

C	1.70660	4.78854	1.24522
H	2.56608	4.88116	0.54763
H	0.83810	5.29442	0.77208
H	1.96165	5.35347	2.16148
H	-1.39164	0.16203	-2.79115
C	-2.16617	2.12878	-1.12408
C	-2.17313	2.95133	-2.28024
C	-2.46320	2.74205	0.11904
C	-2.45537	4.32691	-2.19962
H	-1.96334	2.50715	-3.26809
C	-2.74225	4.11780	0.20622
H	-2.49588	2.13768	1.03937
C	-2.73564	4.91506	-0.95330
H	-2.46604	4.94008	-3.11497
H	-2.98039	4.56848	1.18314
H	-2.96407	5.99076	-0.88729
**INT-1$_{Ph_3SiH}$**			
Si	-1.93668	-0.02872	-0.49477
H	-0.99675	-4.10139	1.27227
O	0.97821	-3.92883	0.73594
C	-0.13058	-3.44494	0.96772
O	-0.48486	-2.18358	0.89981
Ge	0.36019	-0.31004	0.57617
C	2.07733	-1.18314	-0.19128
C	0.46726	0.04956	2.56808
N	3.36071	-0.94478	0.21915
N	2.18581	-2.09491	-1.19579
C	1.16548	-0.80190	3.47929
C	-0.29131	1.14742	3.09085
C	4.26833	-1.72671	-0.50224
C	3.78446	-0.01421	1.26021
C	3.51654	-2.46223	-1.39980
C	1.08998	-2.70307	-1.94153
C	1.13975	-0.49932	4.86262
C	-0.29463	1.40078	4.47888
H	4.28193	0.86909	0.81085
H	4.49531	-0.51766	1.94245
H	2.90910	0.31130	1.84689
H	0.13393	-2.25144	-1.63239
H	1.05056	-3.78436	-1.71339
H	1.23413	-2.54332	-3.02827
H	1.69209	-1.15672	5.55432
C	0.42403	0.58969	5.36618
H	-0.88157	2.25086	4.86319
H	0.41586	0.80093	6.44708
C	1.93204	-2.05364	3.10694
H	1.95330	-2.29407	2.03134
H	2.97453	-2.00515	3.48807
H	1.46951	-2.93506	3.59880
C	-1.14718	2.04857	2.23023
H	-0.73599	2.22283	1.21700
H	-2.15748	1.60779	2.09326
H	-1.29807	3.03681	2.70897
C	5.73996	-1.70784	-0.24717
H	5.98925	-2.07286	0.77242
H	6.25999	-2.36715	-0.96712
H	6.17268	-0.69054	-0.35307
C	3.92225	-3.49767	-2.39603
H	3.48925	-4.48933	-2.14568
H	3.59518	-3.24165	-3.42580
H	5.02270	-3.60761	-2.41205
C	-3.28061	-0.67352	0.69478

C	-4.58373	-0.79793	0.14408
C	-3.10261	-1.01670	2.05565
C	-5.66628	-1.23645	0.92626
H	-4.76302	-0.55493	-0.91690
C	-4.18575	-1.45202	2.84009
H	-2.10293	-0.97050	2.51092
C	-5.46971	-1.56220	2.27949
H	-6.66653	-1.32743	0.47351
H	-4.02079	-1.71514	3.89710
H	-6.31587	-1.90803	2.89427
C	1.42036	1.63907	-0.23152
N	1.70867	1.77091	-1.56829
N	1.93973	2.78087	0.32281
C	2.38257	2.96729	-1.84935
C	1.40909	0.79411	-2.61010
C	2.52526	3.61600	-0.64037
C	1.94864	3.13608	1.73963
H	1.06828	1.31249	-3.52650
H	0.59909	0.12071	-2.28126
H	2.30702	0.19280	-2.86045
H	1.58763	2.28484	2.33982
H	1.29258	4.00935	1.92730
H	2.97846	3.39459	2.05619
C	3.14062	4.93646	-0.30831
H	3.51805	5.42654	-1.22551
H	2.40881	5.62780	0.16087
H	3.99757	4.83859	0.39206
C	2.81028	3.36642	-3.22472
H	3.48770	2.61787	-3.68789
H	1.94662	3.50230	-3.91077
H	3.35523	4.32861	-3.19126
C	-2.36037	1.79249	-0.92247
C	-1.62124	2.58174	-1.83945
C	-3.50229	2.39458	-0.33240
C	-2.00227	3.89676	-2.16094
H	-0.72615	2.17025	-2.32564
C	-3.88806	3.70904	-0.64816
H	-4.11209	1.82350	0.38524
C	-3.13991	4.46648	-1.56501
H	-1.40991	4.47846	-2.88556
H	-4.78418	4.14159	-0.17533
H	-3.44379	5.49486	-1.81682
C	-2.07798	-1.08775	-2.07882
C	-2.37053	-2.47181	-1.95568
C	-1.98455	-0.54754	-3.38575
C	-2.53891	-3.28337	-3.09050
H	-2.47902	-2.91554	-0.95347
C	-2.14976	-1.35857	-4.52367
H	-1.81625	0.53224	-3.52559
C	-2.42201	-2.73012	-4.37890
H	-2.77549	-4.35254	-2.96836
H	-2.08593	-0.91184	-5.52898
H	-2.56203	-3.36407	-5.26893
	**Ph_3SiH**		
Si	-2.34624	0.11622	-1.50364
C	-4.24333	0.14286	-1.43542
C	-4.98055	1.32940	-1.66492
C	-4.96470	-1.04815	-1.17112
C	-6.38706	1.32695	-1.63607
H	-4.44678	2.27383	-1.86363
C	-6.36952	-1.05460	-1.13980
H	-4.42074	-1.98977	-0.98284

C	-7.08381	0.13485	-1.37330
H	-6.94153	2.26219	-1.81650
H	-6.91072	-1.99136	-0.92945
H	-8.18551	0.13195	-1.34726
C	-1.64634	1.87158	-1.32193
C	-1.14493	2.59234	-2.43243
C	-1.63446	2.50758	-0.05541
C	-0.65338	3.90224	-2.28538
H	-1.13221	2.12014	-3.42904
C	-1.14289	3.81524	0.09608
H	-2.01334	1.97197	0.83204
C	-0.65146	4.51572	-1.02073
H	-0.26658	4.44527	-3.16298
H	-1.14014	4.29007	1.09066
H	-0.26382	5.54061	-0.90341
C	-1.74692	-0.67896	-3.11999
C	-0.41735	-1.15732	-3.23004
C	-2.59070	-0.79447	-4.25080
C	0.05283	-1.72725	-4.42533
H	0.26518	-1.08720	-2.36563
C	-2.12348	-1.36290	-5.44982
H	-3.63342	-0.43998	-4.19221
C	-0.80090	-1.83038	-5.53889
H	1.08959	-2.09575	-4.48847
H	-2.79741	-1.44425	-6.31808
H	-0.43441	-2.27886	-6.47643
H	-1.84218	-0.71642	-0.35111

Et_2NH

N	2.80115	0.39946	-0.49834
H	2.27349	0.38356	-1.38317
C	3.82247	-0.64264	-0.53054
H	4.33962	-0.64248	0.45669
C	3.31910	1.74525	-0.27329
H	4.10588	2.04627	-1.02098
C	3.20941	-2.02048	-0.78567
H	2.43900	-2.25324	-0.02206
H	2.72018	-2.06522	-1.78327
H	3.98336	-2.81486	-0.76163
H	4.63103	-0.44505	-1.28939
C	2.19662	2.78419	-0.26812
H	1.68781	2.83234	-1.25572
H	1.43090	2.52899	0.49303
H	2.58935	3.79833	-0.04932
H	3.83526	1.75015	0.71445

TS-4

O	0.51285	-0.12730	-0.19740
Si	-0.69948	-1.26308	-0.20253
C	-2.40525	-0.43108	-0.10936
C	-3.60342	-1.18429	-0.10682
C	-2.50559	0.97720	-0.03816
C	-4.85762	-0.55370	-0.03504
H	-3.56185	-2.28663	-0.16154
C	-3.75744	1.61499	0.03349
H	-1.57833	1.57429	-0.04126
C	-4.93674	0.84965	0.03534
H	-5.77950	-1.15831	-0.03346
H	-3.81451	2.71484	0.08799
H	-5.91943	1.34570	0.09162
H	-0.66529	-2.11331	-1.45685
C	1.97210	0.01706	1.20000
O	2.23913	-0.97452	1.83582

H	1.50690	0.95140	1.58693
N	2.80557	0.35573	-0.11729
H	1.87042	0.18380	-0.69747
C	3.86072	-0.66733	-0.38197
H	4.39352	-0.85556	0.57415
C	3.27305	1.77399	-0.15045
H	4.04193	1.84349	-0.94811
C	3.29841	-1.97040	-0.94578
H	2.62605	-2.45828	-0.21560
H	2.73964	-1.80143	-1.88907
H	4.13510	-2.66505	-1.16395
H	4.57965	-0.20962	-1.09292
C	2.14980	2.77422	-0.42289
H	1.64166	2.55474	-1.38349
H	1.37716	2.77337	0.37036
H	2.57606	3.79624	-0.48115
H	3.78919	1.99364	0.81101
H	-0.56309	-2.21532	0.96539
	**P1**		
C	2.70132	0.05488	1.75494
O	2.64405	-1.09355	2.17967
H	2.32500	0.94849	2.33486
N	3.22965	0.42129	0.53947
C	3.80501	-0.62935	-0.31079
H	4.34169	-1.33392	0.35982
C	3.29393	1.82238	0.13241
H	4.35447	2.09044	-0.08477
C	2.77204	-1.41295	-1.13193
H	2.05225	-1.91486	-0.45530
H	2.20662	-0.75788	-1.82558
H	3.27701	-2.19645	-1.73489
H	4.55977	-0.15144	-0.97172
C	2.41036	2.18908	-1.06746
H	2.71361	1.64451	-1.98534
H	1.34515	1.95393	-0.86496
H	2.49066	3.27475	-1.28446
H	2.99530	2.42816	1.01555
	**$PhSiH_2OH$**		
O	0.40066	0.04679	0.10728
Si	-0.69755	-1.18918	-0.23032
C	-2.40275	-0.40453	-0.11499
C	-3.57161	-1.20084	-0.06158
C	-2.54412	1.00306	-0.09655
C	-4.84491	-0.60948	-0.00073
H	-3.49238	-2.30204	-0.06250
C	-3.81699	1.59680	-0.03098
H	-1.64195	1.63538	-0.12590
C	-4.96879	0.79181	0.01427
H	-5.74510	-1.24386	0.03990
H	-3.91038	2.69481	-0.01395
H	-5.96665	1.25669	0.06495
H	-0.43840	-1.76070	-1.60505
H	1.32337	-0.13241	-0.14500
H	-0.59337	-2.33318	0.74789
	**TS-11**		
C	1.42248	0.18946	-0.57847
O	0.94739	-0.90365	-1.11798
H	0.12661	0.02655	0.72829
Si	-0.62601	-1.21975	-0.13281
C	-2.31042	-0.29918	0.02256
H	-1.04560	-2.18264	-1.25119

C	-2.52714	0.73900	0.95828
C	-3.39507	-0.68656	-0.79918
C	-3.78432	1.35315	1.08936
H	-1.68528	1.06632	1.59447
C	-4.65518	-0.07253	-0.68077
H	-3.24266	-1.48810	-1.54297
C	-4.85227	0.94798	0.26663
H	-3.93474	2.15433	1.83200
H	-5.48734	-0.38921	-1.33116
H	-5.83778	1.43290	0.36079
H	0.95755	1.17990	-0.76219
N	2.69656	0.25242	-0.15472
C	3.29196	1.54357	0.21595
C	3.45930	-0.98411	0.09604
H	2.46182	2.25540	0.40817
H	3.81887	1.41368	1.18568
H	4.51861	-0.78131	-0.16809
C	4.24432	2.10361	-0.84645
H	5.08771	1.41200	-1.04991
H	3.71255	2.28556	-1.80270
H	4.67591	3.06620	-0.50311
C	3.33519	-1.48453	1.53994
H	3.70253	-0.73886	2.27555
H	2.28004	-1.72254	1.78302
H	3.93629	-2.40777	1.66927
H	3.07936	-1.74424	-0.61340
H	-0.27398	-2.17025	0.99549

TS-13

C	-2.17193	-1.66614	0.25974
O	-0.90775	-1.92027	0.00136
H	-2.30244	-0.35688	-0.35975
Si	-1.62376	0.95542	-1.25970
C	-3.26325	1.94533	-1.11999
C	-4.51559	1.31750	-0.92135
C	-3.24551	3.35362	-1.26299
C	-5.70727	2.06122	-0.88277
H	-4.56141	0.22207	-0.80249
C	-4.43288	4.10609	-1.21669
H	-2.28268	3.87004	-1.41989
C	-5.66715	3.46008	-1.02793
H	-6.67255	1.54991	-0.73708
H	-4.39562	5.20167	-1.32992
H	-6.59906	4.04655	-0.99194
H	-2.43195	-1.26710	1.27188
H	-0.69704	2.15043	-1.50136
Ge	0.31047	-0.16686	0.14965
C	0.08961	-0.16371	2.16250
C	1.86772	-1.42596	-0.40990
C	0.36267	-1.29658	2.99542
C	-0.52475	0.99103	2.74352
N	3.12911	-1.48951	0.11372
N	1.89265	-2.25594	-1.49403
C	0.10378	-1.20816	4.38306
C	0.88123	-2.62446	2.47612
C	-0.77004	1.03097	4.13433
C	-0.98756	2.17882	1.92268
C	3.94174	-2.35629	-0.62604
C	3.60404	-0.77326	1.29341
C	3.15316	-2.84333	-1.65171
C	0.78628	-2.49797	-2.42031
H	0.32634	-2.07984	5.02027
C	-0.44360	-0.05400	4.95683

H	0.39607	-2.89287	1.51827
H	1.98042	-2.62264	2.31599
H	0.67615	-3.42904	3.20993
H	-1.23855	1.92889	4.56972
H	-0.29866	2.44474	1.09577
H	-1.97778	1.98012	1.45612
H	-1.11767	3.07818	2.55749
C	5.36558	-2.63751	-0.27148
H	4.27483	0.05931	0.99996
H	4.16080	-1.46644	1.95335
H	2.73891	-0.37145	1.84817
C	3.46581	-3.80890	-2.74787
H	-0.13975	-2.10435	-1.96550
H	0.66710	-3.58742	-2.57560
H	0.98876	-2.01747	-3.39895
H	-0.63713	-0.00975	6.04024
H	5.97722	-1.71145	-0.23194
H	5.82308	-3.30622	-1.02455
H	5.45841	-3.14055	0.71510
H	2.85824	-4.73588	-2.66983
H	4.53007	-4.10734	-2.70539
H	3.27967	-3.37453	-3.75274
C	1.83185	1.53695	-0.12149
N	2.15351	1.93771	-1.39201
N	2.42066	2.48267	0.67871
C	2.92558	3.10634	-1.39687
C	1.78052	1.23745	-2.61619
C	3.09482	3.45731	-0.07235
C	2.41193	2.49996	2.13876
C	3.40236	3.76542	-2.65024
H	2.64979	0.69112	-3.03811
H	1.40634	1.95704	-3.36834
H	0.96771	0.52297	-2.39175
C	3.81126	4.61186	0.54915
H	1.82255	1.64592	2.51386
H	1.95024	3.43528	2.51269
H	3.44738	2.43919	2.53149
H	4.01226	3.08119	-3.27762
H	4.03178	4.64242	-2.40800
H	2.55868	4.12739	-3.27655
H	3.12414	5.26288	1.13098
H	4.27882	5.24052	-0.23198
H	4.61794	4.28415	1.23920
H	-1.55184	0.16530	-2.55259
N	-3.15373	-2.54036	-0.19406
C	-4.50561	-2.44813	0.36025
C	-2.93007	-3.39732	-1.35487
H	-4.58171	-1.49258	0.92170
H	-5.24180	-2.36945	-0.47201
C	-4.87415	-3.61959	1.28175
H	-3.51016	-4.33285	-1.20016
C	-3.30696	-2.76884	-2.70676
H	-1.86020	-3.68927	-1.34484
H	-4.82287	-4.59238	0.75040
H	-4.19015	-3.66998	2.15346
H	-5.91046	-3.50257	1.66079
H	-4.37878	-2.48338	-2.74363
H	-2.71019	-1.85651	-2.91246
H	-3.13074	-3.49179	-3.53053
**TS-12**			
C	-5.61578	0.88125	0.45144
O	-4.29700	1.00522	0.44494

H	-5.37033	-0.91645	0.19520
Si	-3.73664	-0.67271	-0.09724
C	-3.87966	-1.51555	-1.81100
C	-2.97305	-1.19463	-2.84928
C	-4.87496	-2.48692	-2.07629
C	-3.04728	-1.82701	-4.10394
H	-2.20045	-0.42622	-2.67014
C	-4.94252	-3.13807	-3.31940
H	-5.61082	-2.73236	-1.29069
C	-4.02926	-2.80592	-4.33781
H	-2.34097	-1.55415	-4.90519
H	-5.71612	-3.90150	-3.50167
H	-4.09006	-3.30485	-5.31834
H	-6.18764	0.85609	-0.49781
N	-6.33551	1.22748	1.52393
C	-7.80263	1.34254	1.42494
C	-5.70592	1.34962	2.85311
H	-8.12422	0.78504	0.52087
H	-8.24361	0.80827	2.29290
H	-6.22483	2.17112	3.38929
C	-8.29478	2.79193	1.37006
H	-7.99564	3.36645	2.27061
H	-7.89829	3.31717	0.47734
H	-9.40214	2.81135	1.31595
C	-5.75280	0.04777	3.66137
H	-6.79203	-0.29513	3.84323
H	-5.20637	-0.76383	3.13995
H	-5.27687	0.20653	4.65034
H	-4.66025	1.67163	2.68728
Ge	2.93743	0.56524	1.06087
C	4.73423	0.53573	0.09226
C	2.23163	-1.36993	0.92412
C	5.27949	-0.50987	-0.71629
C	5.56393	1.65645	0.42200
N	1.21425	-1.99516	0.25544
N	2.62329	-2.26669	1.88554
C	6.62248	-0.42445	-1.15175
C	4.48180	-1.71511	-1.17431
C	6.89750	1.70463	-0.03715
C	5.04890	2.82571	1.24120
C	0.96554	-3.26775	0.78862
C	0.50165	-1.46826	-0.90402
C	1.87059	-3.44433	1.81911
C	3.71273	-2.02979	2.82809
H	7.03397	-1.23331	-1.77806
C	7.43182	0.66717	-0.81273
H	4.32507	-2.45451	-0.36022
H	3.47604	-1.43019	-1.54403
H	5.00680	-2.24561	-1.99373
H	7.52359	2.57421	0.22217
H	4.21007	3.35314	0.73614
H	4.66220	2.50193	2.23134
H	5.84756	3.57411	1.41383
C	-0.10134	-4.17166	0.26197
H	-0.50448	-1.08609	-0.62748
H	0.38173	-2.26468	-1.66312
H	1.09209	-0.64133	-1.33543
C	2.08674	-4.60637	2.73353
H	3.76447	-0.94035	3.03995
H	4.68615	-2.35790	2.40998
H	3.51911	-2.57219	3.77169
H	8.47576	0.71362	-1.16099

H	-1.10144	-3.68941	0.28721
H	-0.15851	-5.09350	0.87082
H	0.09062	-4.48028	-0.78829
H	3.12393	-4.99856	2.66966
H	1.40289	-5.43403	2.46717
H	1.89220	-4.34657	3.79644
H	-2.33481	-0.03913	-0.33377
H	-3.41532	-1.55552	1.09500
C	1.65401	1.52002	-0.25958
N	0.42750	1.95813	0.17129
N	1.83920	2.13960	-1.46828
C	-0.16296	2.81895	-0.75661
C	-0.20292	1.57746	1.43269
C	0.73968	2.94206	-1.79827
C	2.99098	1.97113	-2.35170
C	-1.50887	3.43326	-0.54861
H	-1.01993	0.84917	1.24991
H	-0.62196	2.47515	1.92685
H	0.57286	1.13464	2.09004
C	0.66882	3.73726	-3.06099
H	3.73667	1.32918	-1.84931
H	3.45604	2.95336	-2.56704
H	2.67707	1.50799	-3.30911
H	-2.28456	2.66583	-0.33962
H	-1.81789	3.99281	-1.45169
H	-1.50729	4.15116	0.30032
H	1.47376	4.50119	-3.11661
H	-0.29842	4.27022	-3.12460
H	0.75790	3.09736	-3.96459

TS-5

C	1.31244	0.25656	-0.42488
O	0.80440	-0.85158	-0.87487
H	0.23352	-0.00144	1.14346
Si	-0.58038	-1.23322	0.38748
C	-2.14153	-0.12471	0.24640
C	-2.18203	1.18763	0.77442
C	-3.31296	-0.61299	-0.38492
C	-3.33469	1.98689	0.67116
H	-1.28665	1.58078	1.28796
C	-4.46886	0.17945	-0.49508
H	-3.30925	-1.64259	-0.77771
C	-4.48157	1.48393	0.03120
H	-3.34101	3.00554	1.09333
H	-5.36873	-0.22458	-0.98784
H	-5.38737	2.10661	-0.05251
H	0.76866	1.22226	-0.50031
N	2.62430	0.36950	-0.17080
C	3.20342	1.67292	0.18320
C	3.46237	-0.83968	-0.06390
H	2.36919	2.34131	0.48222
H	3.83071	1.53490	1.09017
H	4.47862	-0.57329	-0.42257
C	4.01941	2.30509	-0.94947
H	4.86447	1.65729	-1.26133
H	3.38598	2.49464	-1.84002
H	4.44577	3.27364	-0.61685
C	3.50656	-1.40985	1.35863
H	3.92364	-0.68440	2.08766
H	2.49126	-1.69817	1.69799
H	4.14737	-2.31483	1.37933
H	3.04228	-1.58485	-0.76671
O	-1.03992	-2.59452	-0.56928

H	-1.16600	-2.38317	-1.51287
H	-0.30031	-2.09077	1.60333
**INT-3**			
C	1.65341	0.48377	1.74984
O	0.49630	0.53691	0.86616
H	1.56446	-0.41890	2.40101
Si	-0.46101	-0.76245	0.40270
C	-2.13103	-0.05721	-0.06197
C	-2.62076	1.11541	0.56211
C	-2.94132	-0.69289	-1.03343
C	-3.88659	1.63046	0.23481
H	-1.99283	1.63648	1.30306
C	-4.20711	-0.17855	-1.36307
H	-2.56678	-1.59434	-1.54510
C	-4.68246	0.98213	-0.72686
H	-4.25284	2.54553	0.72791
H	-4.82477	-0.68308	-2.12371
H	-5.67499	1.38591	-0.98483
H	1.58333	1.39182	2.38594
N	2.92641	0.46991	1.11186
C	3.36174	1.72810	0.49298
C	3.27839	-0.77129	0.41984
H	3.10921	2.54039	1.20935
H	4.47280	1.71968	0.43221
H	2.76018	-0.86779	-0.56970
C	2.77297	2.06315	-0.88955
H	3.03975	1.29777	-1.64859
H	1.66777	2.12417	-0.84664
H	3.16956	3.03602	-1.24991
C	4.78601	-0.96497	0.22689
H	5.21737	-0.22748	-0.48089
H	5.32242	-0.86772	1.19353
H	4.99292	-1.97304	-0.18796
H	2.88956	-1.60339	1.05019
O	0.07477	-1.58491	-0.96895
H	0.75050	-2.26552	-0.80129
H	-0.46930	-1.73329	1.56035
**TS-6**			
C	-2.48317	-0.27458	1.18275
O	-0.27419	-0.43918	-0.07319
H	-1.34929	1.56732	0.18774
Si	-0.20729	1.15537	-0.89816
C	0.84824	2.70184	-0.40010
C	0.37803	3.66558	0.52171
C	2.12265	2.91676	-0.97721
C	1.13554	4.80493	0.84618
H	-0.60936	3.50985	0.99208
C	2.89047	4.05175	-0.65914
H	2.51231	2.17232	-1.69355
C	2.39706	5.00057	0.25456
H	0.74462	5.54368	1.56600
H	3.87932	4.19788	-1.12516
H	2.99708	5.88976	0.50934
Si	0.26329	-1.92678	-0.59746
H	-2.30350	0.62476	1.78380
H	-1.94421	-1.20900	1.38001
N	-3.45422	-0.28158	0.29747
C	-4.14101	0.98408	-0.03548
C	-3.71541	-1.48091	-0.52672
H	-4.68976	0.82302	-0.98425
H	-3.56975	-1.18728	-1.58880

H	-2.92966	-2.22466	-0.28116
C	2.03390	-2.30725	-0.07076
C	2.52671	-3.63600	-0.10844
C	2.90340	-1.28404	0.37714
C	3.84217	-3.93329	0.28768
H	1.87315	-4.45584	-0.45460
C	4.22002	-1.57797	0.77363
H	2.54450	-0.24250	0.41128
C	4.69100	-2.90228	0.72977
H	4.20838	-4.97223	0.24946
H	4.88271	-0.76760	1.11805
H	5.72349	-3.13236	1.03922
H	-1.20105	1.36419	-2.04731
C	-5.11519	-2.06030	-0.30957
H	-5.26225	-2.37623	0.74357
H	-5.91635	-1.33884	-0.57261
H	-5.24732	-2.95223	-0.95493
C	-5.08249	1.48792	1.06085
H	-5.90118	0.76873	1.26875
H	-4.53955	1.68021	2.00878
H	-5.53947	2.44694	0.74291
H	-3.31969	1.71797	-0.22728
O	-0.74571	-3.08108	0.17143
H	-0.34386	-3.42892	0.98926
H	0.92655	0.56966	-1.81224
H	0.00975	-2.15594	-2.05671
**INT-4**			
O	-0.44636	-0.10633	0.50924
Si	0.55256	1.17205	0.95642
C	-0.29057	2.81494	0.58814
C	-0.10249	3.46725	-0.65496
C	-1.14059	3.42730	1.54186
C	-0.74556	4.68438	-0.93924
H	0.56081	3.01958	-1.41454
C	-1.78578	4.64399	1.26172
H	-1.29999	2.94902	2.52338
C	-1.58906	5.27346	0.01943
H	-0.58602	5.17758	-1.91168
H	-2.44228	5.10581	2.01674
H	-2.09216	6.22884	-0.20077
Si	-0.94827	-1.62892	0.99781
C	-2.49935	-2.02097	0.00901
C	-2.94895	-3.36022	-0.10195
C	-3.25218	-1.00495	-0.62731
C	-4.11625	-3.67378	-0.81923
H	-2.37361	-4.17193	0.37527
C	-4.41963	-1.31594	-1.34587
H	-2.91140	0.04122	-0.56321
C	-4.85396	-2.65029	-1.44109
H	-4.45169	-4.72079	-0.89537
H	-4.99334	-0.51312	-1.83671
H	-5.76956	-2.89420	-2.00385
H	1.82450	1.08562	0.15991
O	0.21850	-2.81250	0.75187
H	0.35810	-3.05134	-0.18323
H	0.85021	1.08238	2.42981
H	-1.13996	-1.63317	2.48221
**P2**			
C	0.00041	1.47808	0.18967
H	0.89035	1.97297	-0.24787
H	0.00139	1.68940	1.29631

H	-0.89014	1.97322	-0.24631
N	-0.00009	0.05519	-0.11997
C	1.21470	-0.61962	0.34379
H	1.37210	-0.47744	1.45141
H	1.05802	-1.71080	0.19854
C	2.48330	-0.20459	-0.40879
H	2.34339	-0.32870	-1.50266
H	2.76680	0.85106	-0.21771
H	3.34063	-0.83332	-0.09104
C	-1.21499	-0.61917	0.34427
H	-1.05824	-1.71047	0.19997
H	-1.37255	-0.47613	1.45175
C	-2.48356	-0.20507	-0.40886
H	-2.76688	0.85090	-0.21929
H	-2.34381	-0.33070	-1.50258
H	-3.34099	-0.83318	-0.09013
**TS-9**			
Ge	-0.09629	-0.06003	-0.87693
N	2.52185	-1.73745	-0.70789
N	2.63779	-0.26429	0.90438
N	-2.76437	-1.51656	0.09526
N	-3.08894	0.45320	-0.75989
C	1.75299	-1.13474	0.28062
C	3.81017	-1.24022	-0.72364
C	3.88508	-0.29837	0.30552
C	1.98429	-2.73132	-1.62525
H	2.76716	-3.05220	-2.33359
H	1.13374	-2.27943	-2.18460
H	1.61536	-3.59335	-1.03602
C	4.86663	-1.69262	-1.67778
H	5.08791	-2.77486	-1.56178
H	5.80800	-1.14103	-1.49804
H	4.57470	-1.51944	-2.73514
C	5.03419	0.53550	0.76721
H	4.77453	1.61452	0.78916
H	5.90162	0.40978	0.09298
H	5.36430	0.25284	1.79016
C	2.33665	0.56796	2.06917
H	3.16610	0.48748	2.79777
H	1.40666	0.19501	2.53031
H	2.20999	1.62571	1.76352
C	-2.10460	-0.41079	-0.35626
C	-4.15083	-1.33742	0.03201
C	-4.35844	-0.08522	-0.51951
C	-2.13103	-2.73454	0.59523
H	-1.91448	-2.65848	1.67943
H	-1.18151	-2.90848	0.05971
H	-2.79562	-3.59700	0.40823
C	-5.13074	-2.36790	0.49079
H	-5.07789	-3.29673	-0.11700
H	-6.16239	-1.97854	0.40422
H	-4.96899	-2.65079	1.55211
C	-5.63019	0.62808	-0.84657
H	-5.69700	1.61634	-0.34511
H	-6.49764	0.02828	-0.51307
H	-5.74901	0.79453	-1.93910
C	-2.87017	1.75999	-1.38234
H	-2.72986	2.55260	-0.62106
H	-3.73924	2.00894	-2.01785
H	-1.97072	1.72817	-2.02524
C	0.09071	1.78177	-0.04190
C	-0.50364	2.24120	1.17610

C	-0.30478	3.58352	1.57451
H	-0.75917	3.93545	2.51540
C	0.46732	4.46640	0.80708
H	0.61140	5.50701	1.13845
C	1.06769	4.01413	-0.37690
H	1.68397	4.70349	-0.97710
C	0.89309	2.68369	-0.81413
O	0.30495	-1.61198	2.34031
C	0.83286	-2.26475	1.45412
O	0.97577	-3.40681	1.05865
C	-1.33800	1.36244	2.08820
H	-0.99194	0.31165	2.11011
H	-1.31344	1.74733	3.12777
H	-2.40799	1.35697	1.78456
C	1.55671	2.27333	-2.11949
H	0.82295	2.16971	-2.94854
H	2.29903	3.03118	-2.44023
H	2.08487	1.29954	-2.04393

INT-7

Ge	-0.95602	0.18798	-1.20188
N	4.13481	-1.63485	-0.19470
N	3.54350	0.40366	0.35585
N	-2.45900	-2.20871	0.12499
N	-3.70605	-0.43422	0.13066
C	3.07012	-0.78037	-0.13525
C	5.28122	-0.99622	0.26646
C	4.90831	0.29736	0.60857
C	4.12799	-3.03309	-0.64646
H	4.34213	-3.70210	0.21053
H	4.91087	-3.16772	-1.41729
H	3.13631	-3.25038	-1.07798
C	6.60955	-1.67380	0.33460
H	6.58067	-2.57437	0.98345
H	7.37088	-0.98735	0.74864
H	6.95989	-1.99706	-0.66861
C	5.72051	1.43201	1.13876
H	5.70116	2.30716	0.45548
H	6.77593	1.12608	1.26030
H	5.35358	1.77529	2.12906
C	2.80105	1.65462	0.56321
H	2.92406	1.98124	1.61355
H	1.73300	1.48253	0.35413
H	3.20328	2.43697	-0.10966
C	-2.42701	-0.86264	-0.11056
C	-3.73096	-2.62312	0.53820
C	-4.52864	-1.49422	0.53158
C	-1.32897	-3.12145	-0.02547
H	-0.89884	-3.37990	0.96425
H	-0.54158	-2.66175	-0.65307
H	-1.66890	-4.05240	-0.51821
C	-4.05090	-4.03927	0.89040
H	-3.92850	-4.72360	0.02344
H	-5.10029	-4.12235	1.23037
H	-3.40397	-4.42034	1.70885
C	-5.97577	-1.31884	0.85861
H	-6.13041	-0.62978	1.71641
H	-6.42911	-2.29095	1.12911
H	-6.54928	-0.91206	-0.00132
C	-4.19982	0.92969	-0.04945
H	-4.77017	1.24878	0.84430
H	-4.86202	0.98979	-0.93732
H	-3.33895	1.61100	-0.18119

C	-1.03108	1.98938	-0.20821
C	-0.95188	2.17373	1.20836
C	-0.88048	3.48317	1.73615
H	-0.82095	3.61979	2.82907
C	-0.89455	4.60662	0.89667
H	-0.84131	5.61984	1.32610
C	-0.98133	4.43269	-0.49120
H	-0.99685	5.31348	-1.15425
C	-1.04830	3.14000	-1.05793
O	0.75427	-0.29716	-0.11501
C	1.64986	-1.12273	-0.56442
O	1.46320	-2.12051	-1.26921
C	-0.97256	1.01571	2.18780
H	-0.26781	0.21287	1.89629
H	-0.71655	1.35476	3.21182
H	-1.98076	0.55092	2.24307
C	-1.15404	3.02688	-2.57030
H	-2.12277	2.58637	-2.89123
H	-1.07557	4.02298	-3.04952
H	-0.36115	2.38044	-3.00308

TS-10

Ge	1.58614	-0.13870	-0.96308
N	-1.48408	-1.99120	-1.61840
N	-1.63312	-2.85202	0.39985
N	2.02829	2.93650	-0.51102
N	3.85543	1.79306	-0.25137
C	-1.43172	-1.69446	-0.28882
C	-1.73987	-3.35322	-1.77764
C	-1.82005	-3.89905	-0.50287
C	-1.48957	-0.98555	-2.67846
H	-1.85322	-1.44484	-3.61360
H	-0.46711	-0.57893	-2.82864
H	-2.17617	-0.16884	-2.36062
C	-1.90266	-4.00183	-3.11294
H	-2.80604	-3.63501	-3.64572
H	-2.01592	-5.09522	-2.99262
H	-1.02508	-3.82549	-3.76925
C	-2.04831	-5.30654	-0.06103
H	-1.21874	-5.67830	0.57639
H	-2.12457	-5.97690	-0.93698
H	-2.99033	-5.40777	0.51895
C	-1.53677	-3.02947	1.85352
H	-2.35675	-3.68873	2.19316
H	-1.63495	-2.04533	2.34311
H	-0.55792	-3.48027	2.11357
C	2.50063	1.65984	-0.39297
C	3.07104	3.86835	-0.43515
C	4.23575	3.14018	-0.27430
C	0.62924	3.31711	-0.71047
H	-0.01686	2.44364	-0.52080
H	0.47407	3.68893	-1.74390
H	0.35295	4.11758	0.00255
C	2.84542	5.34288	-0.51091
H	2.34179	5.63841	-1.45545
H	3.81018	5.88193	-0.46481
H	2.21903	5.71138	0.32968
C	5.65435	3.58532	-0.12981
H	6.08106	3.30638	0.85763
H	5.72170	4.68583	-0.21835
H	6.30943	3.14715	-0.91230
C	4.80469	0.68898	-0.12409
H	4.25196	-0.23501	0.12980

H	5.52259	0.89927	0.69173
H	5.36748	0.54286	-1.06888
C	2.12710	-1.36562	0.58689
C	1.99193	-1.07330	1.98195
C	2.30263	-2.07864	2.92833
H	2.20877	-1.84800	4.00248
C	2.73647	-3.34989	2.52828
H	2.97789	-4.11532	3.28314
C	2.88018	-3.63603	1.16278
H	3.23952	-4.62880	0.84489
C	2.58605	-2.66154	0.18404
O	-0.23253	0.37620	-0.06889
C	1.52490	0.26526	2.51570
H	0.41735	0.35166	2.50563
H	1.85723	0.40886	3.56305
H	1.90813	1.11000	1.90988
C	2.81350	-3.02849	-1.27376
H	1.93983	-2.78999	-1.91607
H	3.67729	-2.48116	-1.71064
H	3.03099	-4.10976	-1.38157
C	-1.21011	-0.34170	0.31845
O	-1.84848	-0.04872	1.41870
H	-3.00278	0.05669	-0.51772
Si	-3.70701	0.13780	1.02131
H	-3.90926	0.16941	2.53752
C	-4.46160	1.83245	0.57765
C	-4.89802	2.67425	1.62924
C	-4.65525	2.26778	-0.75467
C	-5.52052	3.90550	1.35686
H	-4.75420	2.35315	2.67449
C	-5.28677	3.49122	-1.03149
H	-4.30966	1.63053	-1.58663
C	-5.71793	4.31415	0.02619
H	-5.85655	4.54715	2.18730
H	-5.44524	3.80770	-2.07525
H	-6.20885	5.27693	-0.18871
H	-4.46328	-1.14425	0.68886

INT-8

Ge	0.44201	1.03749	-0.87689
N	2.83753	-1.78181	-1.63267
N	2.76872	-1.73423	0.55145
N	-2.65191	1.53854	-0.72095
N	-1.50816	3.37994	-0.64127
C	2.00319	-1.77681	-0.56222
C	4.16657	-1.73150	-1.19102
C	4.11857	-1.69851	0.19446
C	2.40304	-1.77990	-3.03294
H	3.29079	-1.68442	-3.68149
H	1.73160	-0.91460	-3.21095
H	1.88089	-2.72376	-3.28378
C	5.34044	-1.72164	-2.11537
H	5.37559	-2.62744	-2.75701
H	6.27877	-1.69827	-1.53046
H	5.34546	-0.83134	-2.77948
C	5.21096	-1.62729	1.20942
H	5.11378	-0.72266	1.84580
H	6.19775	-1.58496	0.71237
H	5.21264	-2.51304	1.87972
C	2.26696	-1.69611	1.92795
H	2.82964	-2.42615	2.54013
H	1.19884	-1.96761	1.91964
H	2.39088	-0.67575	2.34267

C	-1.37924	2.01739	-0.59808
C	-3.57768	2.58353	-0.83614
C	-2.84935	3.75731	-0.78945
C	-3.02037	0.12455	-0.74709
H	-2.12461	-0.47211	-0.49392
H	-3.39835	-0.15664	-1.75122
H	-3.80976	-0.08245	0.00153
C	-5.04709	2.34851	-0.96573
H	-5.29306	1.74020	-1.86199
H	-5.58484	3.31083	-1.05830
H	-5.46255	1.81812	-0.08216
C	-3.29024	5.18314	-0.85988
H	-3.06025	5.73939	0.07450
H	-4.38432	5.23841	-1.01412
H	-2.80770	5.72775	-1.69910
C	-0.39713	4.32689	-0.59985
H	0.47876	3.83382	-0.13732
H	-0.67019	5.20300	0.01833
H	-0.13510	4.67448	-1.62076
C	1.46309	1.79140	0.74707
C	0.99175	1.74336	2.09598
C	1.83656	2.17368	3.14574
H	1.46536	2.13554	4.18369
C	3.12861	2.65498	2.88807
H	3.77140	2.99033	3.71775
C	3.59190	2.71654	1.56619
H	4.60209	3.10672	1.35715
C	2.77712	2.29274	0.49110
O	-0.07387	-0.67764	-0.01683
C	-0.40086	1.26370	2.45187
H	-0.64332	0.30982	1.94372
H	-0.50917	1.12510	3.54643
H	-1.16929	2.00355	2.13913
C	3.33894	2.41314	-0.91651
H	3.30641	1.44882	-1.46625
H	2.76492	3.13510	-1.53652
H	4.39069	2.76265	-0.89640
C	0.46965	-1.80249	-0.59967
O	0.08446	-2.97029	0.10490
H	0.17683	-1.90451	-1.68063
Si	-1.22428	-3.99719	-0.29172
H	-0.81763	-5.30426	0.31434
C	-2.88096	-3.40263	0.37171
C	-3.02749	-2.96072	1.71019
C	-4.03308	-3.45097	-0.45112
C	-4.28446	-2.58107	2.20952
H	-2.14942	-2.91289	2.37536
C	-5.29394	-3.07772	0.04876
H	-3.95090	-3.79239	-1.49722
C	-5.42015	-2.64198	1.37974
H	-4.38264	-2.24532	3.25423
H	-6.18163	-3.13266	-0.60147
H	-6.40792	-2.35641	1.77549
H	-1.30468	-4.09596	-1.79379
	**IMe_4**		
N	1.47698	-0.39296	-1.07188
C	0.43097	-0.01592	-0.26131
C	2.30758	-1.37199	-0.49939
N	0.63971	-0.80183	0.84863
C	1.76596	-1.63648	0.74312
C	-0.23616	-0.75440	2.00757
H	-0.71159	-1.74013	2.20016

H	0.31420	-0.44641	2.92247
H	-1.02395	-0.00928	1.79207
C	1.68131	0.18196	-2.39106
H	1.63509	-0.59350	-3.18563
H	0.87240	0.91721	-2.55644
H	2.66223	0.69906	-2.46319
C	3.50814	-1.94196	-1.18521
H	4.26481	-1.16289	-1.42620
H	4.00478	-2.69483	-0.54156
H	3.24669	-2.44621	-2.14175
C	2.19898	-2.58105	1.81869
H	2.43587	-2.05556	2.77017
H	1.41874	-3.33867	2.05283
H	3.11018	-3.13151	1.51133
**HBpin**			
H	-1.58046	-1.98850	1.04753
B	-1.88647	-1.50997	-0.02003
O	-3.02953	-1.86108	-0.69484
O	-1.12133	-0.56858	-0.66293
C	-2.96254	-1.24329	-2.02116
C	-1.89606	-0.08050	-1.80604
C	-2.48588	-2.33798	-2.99020
C	-4.36618	-0.77017	-2.40726
C	-0.93944	0.14512	-2.97956
C	-2.52584	1.25302	-1.37058
H	-3.17614	-3.20269	-2.92208
H	-2.47572	-1.98289	-4.04053
H	-1.46929	-2.69543	-2.73001
H	-4.35072	-0.23633	-3.37996
H	-5.03991	-1.64494	-2.50863
H	-4.79872	-0.09808	-1.64230
H	-1.49451	0.45433	-3.88929
H	-0.22244	0.95209	-2.72634
H	-0.35468	-0.76417	-3.21447
H	-3.07470	1.74042	-2.20157
H	-3.22487	1.11332	-0.52161
H	-1.72069	1.93787	-1.03659
**TS-26**			
B	-2.06618	0.00051	-0.01718
O	-2.95381	-0.91909	0.51958
O	-2.65081	0.82118	-0.98520
C	-4.22654	-0.78119	-0.19639
C	-4.11423	0.67317	-0.82301
C	-4.27118	-1.89415	-1.25571
C	-5.36508	-0.95986	0.81044
C	-4.76472	0.84106	-2.19571
C	-4.58047	1.77851	0.13566
H	-4.14697	-2.87514	-0.75511
H	-5.23904	-1.90767	-1.79610
H	-3.45613	-1.78365	-1.99925
H	-6.35019	-0.78867	0.32948
H	-5.35595	-1.99637	1.20307
H	-5.26535	-0.27286	1.67147
H	-5.85708	0.65742	-2.13591
H	-4.61827	1.87794	-2.56051
H	-4.33469	0.15044	-2.94530
H	-5.67757	1.74732	0.28845
H	-4.08809	1.69297	1.12568
H	-4.33136	2.77008	-0.29426
Ge	-0.12008	0.01818	0.51842
C	0.39015	-1.00559	2.14256

C	0.88539	1.75167	0.25940
C	0.88704	-0.38099	3.32686
C	0.15377	-2.41535	2.12758
N	2.24258	1.90930	0.32676
N	0.38772	2.99502	-0.00958
C	1.22003	-1.19044	4.43670
C	1.02361	1.12004	3.48352
C	0.49847	-3.18123	3.26302
C	-0.52223	-3.11329	0.96678
C	3.22026	0.87006	0.63867
C	2.60144	3.24200	0.10138
C	1.42172	3.93252	-0.10881
C	-1.03023	3.34865	-0.09190
H	1.60431	-0.70942	5.35093
C	1.04548	-2.57977	4.40412
H	1.93087	1.52729	2.98808
H	0.14627	1.64613	3.05423
H	1.09890	1.39353	4.55434
H	0.31525	-4.26807	3.24984
H	-1.59656	-2.83394	0.92467
H	-0.08876	-2.82997	-0.01490
H	-0.45601	-4.21457	1.06759
H	3.73961	0.52808	-0.27849
H	2.70884	0.01388	1.11016
H	3.96781	1.27155	1.34965
C	4.01859	3.71522	0.11525
C	1.19271	5.38363	-0.38275
H	-1.44856	3.47472	0.92759
H	-1.60112	2.55416	-0.60896
H	-1.13066	4.29469	-0.65297
H	1.31042	-3.19177	5.28073
H	4.48949	3.59835	1.11504
H	4.06550	4.78916	-0.14486
H	4.64868	3.16736	-0.61687
H	0.69559	5.55277	-1.36175
H	2.15860	5.92204	-0.40704
H	0.56631	5.86132	0.39978
H	-1.39346	0.97569	1.39141
C	1.34142	-1.01677	-1.14372
N	2.28775	-2.00929	-1.16284
N	1.09090	-0.78333	-2.47100
C	2.88661	-2.62630	0.01681
C	2.63336	-2.38567	-2.46947
C	1.86921	-1.59744	-3.30785
C	0.15478	0.21874	-2.97882
H	2.74176	-3.72470	-0.00542
H	2.39480	-2.23317	0.92389
H	3.97577	-2.41856	0.06030
C	3.63329	-3.45423	-2.77271
C	1.80243	-1.55137	-4.80057
H	0.66761	1.18366	-3.17279
H	-0.66387	0.37468	-2.25246
H	-0.29243	-0.13580	-3.92621
H	4.63073	-3.23097	-2.33733
H	3.76574	-3.56080	-3.86603
H	3.31694	-4.44458	-2.38012
H	0.80308	-1.84884	-5.18572
H	2.54123	-2.24928	-5.23813
H	2.02530	-0.53909	-5.19983
	**INT-24**		
B	2.86073	-0.77920	-0.37543
O	3.54718	-0.94601	-1.54237

O	2.81057	-1.89410	0.42489
C	3.87128	-2.38663	-1.66720
C	3.73100	-2.89852	-0.16759
C	2.82883	-2.98576	-2.62124
C	5.27402	-2.50712	-2.26263
C	3.09408	-4.27917	-0.01277
C	5.03709	-2.81385	0.63375
H	2.86599	-2.44527	-3.58793
H	3.03424	-4.05606	-2.82208
H	1.80008	-2.89718	-2.21782
H	5.58740	-3.56986	-2.31405
H	5.27783	-2.10271	-3.29433
H	6.02499	-1.94592	-1.67598
H	3.71853	-5.05346	-0.50340
H	3.01702	-4.54051	1.06171
H	2.07843	-4.32019	-0.44878
H	5.76976	-3.57136	0.29144
H	5.50795	-1.81379	0.55131
H	4.82531	-3.00909	1.70428
Ge	2.11887	1.05029	0.30470
C	1.54898	2.49559	-0.91417
C	0.66925	0.66944	1.65943
C	1.60900	3.84429	-0.44912
C	1.12001	2.21101	-2.24429
N	-0.66579	0.93298	1.62057
N	0.90556	0.09964	2.87805
C	1.25609	4.88517	-1.33523
C	2.03376	4.20966	0.95903
C	0.77535	3.28508	-3.09292
C	1.01571	0.79959	-2.78286
C	-1.41760	1.53497	0.51364
C	-1.27848	0.54147	2.81332
C	-0.27823	0.01327	3.61553
C	2.22058	-0.34418	3.34608
H	1.30263	5.92779	-0.98150
C	0.84801	4.61196	-2.64765
H	1.47030	3.64375	1.73124
H	3.11186	4.00368	1.13213
H	1.86908	5.28731	1.15338
H	0.44378	3.06939	-4.12143
H	2.01498	0.33098	-2.89625
H	0.42191	0.13771	-2.11495
H	0.52159	0.79004	-3.77397
H	-2.29647	0.88906	0.26676
H	-0.75449	1.64884	-0.36016
H	-1.77393	2.54125	0.81363
C	-2.74202	0.71645	3.04901
C	-0.35386	-0.55599	4.99520
H	2.85343	0.52434	3.61832
H	2.71413	-0.93805	2.55338
H	2.08995	-0.98335	4.23684
H	0.57794	5.43743	-3.32497
H	-3.00993	1.79035	3.15499
H	-3.04874	0.20323	3.97975
H	-3.33420	0.30233	2.19735
H	-0.04235	-1.62200	5.02851
H	-1.39545	-0.50655	5.36397
H	0.27842	0.00501	5.71558
H	3.21782	1.61895	1.25000
C	-4.31795	-0.41230	-0.01440
N	-5.48766	0.15046	-0.46257
N	-4.47813	-1.73319	-0.35503

C	-5.79763	1.56608	-0.32642
C	-6.35276	-0.77849	-1.06383
C	-5.70148	-1.99442	-0.99431
C	-3.48073	-2.75441	-0.07650
H	-5.94158	2.04450	-1.31773
H	-4.95105	2.05602	0.18723
H	-6.71862	1.72056	0.27362
C	-7.68730	-0.41343	-1.63019
C	-6.11284	-3.35366	-1.46165
H	-3.88753	-3.54582	0.58761
H	-2.62619	-2.26774	0.42778
H	-3.12264	-3.23432	-1.01164
H	-8.36654	0.01369	-0.86076
H	-8.18805	-1.30635	-2.05154
H	-7.60542	0.33583	-2.44732
H	-5.40813	-3.77042	-2.21386
H	-7.11297	-3.31513	-1.93477
H	-6.17141	-4.08497	-0.62630

INT-25

B	1.71021	0.25017	0.18028
O	2.54592	1.31301	0.01395
O	2.31576	-0.97906	0.10709
C	3.86784	0.77782	-0.39784
C	3.78416	-0.73850	0.07578
C	3.94644	0.94261	-1.92143
C	4.94992	1.61032	0.28906
C	4.41270	-1.75018	-0.88138
C	4.28733	-0.96285	1.50781
H	3.80959	2.01123	-2.18078
H	4.93361	0.62294	-2.31025
H	3.16120	0.35891	-2.44263
H	5.95716	1.20447	0.06343
H	4.91364	2.65253	-0.08557
H	4.81935	1.63961	1.38683
H	5.50012	-1.56020	-0.98855
H	4.28951	-2.77625	-0.48057
H	3.95101	-1.71660	-1.88584
H	5.38855	-0.85651	1.56838
H	3.83036	-0.25036	2.22346
H	4.03013	-1.99133	1.83186
Ge	-0.30196	0.40050	0.72218
C	-1.41173	1.98195	0.31484
C	-1.23362	-1.27848	0.07088
C	-2.50675	2.29420	1.17772
C	-1.10539	2.82435	-0.79573
N	-2.20594	-1.44180	-0.87048
N	-0.98452	-2.52366	0.56948
C	-3.26250	3.45847	0.92004
C	-2.89699	1.42952	2.35959
C	-1.89219	3.97571	-1.01375
C	0.03117	2.53014	-1.75170
C	-2.79174	-0.39176	-1.70581
C	-2.57357	-2.78706	-0.96925
C	-1.79892	-3.47594	-0.04942
C	-0.00786	-2.81416	1.62406
H	-4.10703	3.70568	1.58319
C	-2.95751	4.29629	-0.16115
H	-3.02413	0.36177	2.08086
H	-2.13362	1.46304	3.16627
H	-3.85411	1.77182	2.79861
H	-1.65955	4.62915	-1.86990
H	1.01873	2.66077	-1.26292

H	-0.00044	1.48715	-2.13662
H	-0.00455	3.20375	-2.62996
H	-2.48183	-0.52450	-2.76157
H	-2.46014	0.59623	-1.33998
H	-3.89604	-0.43843	-1.64643
C	-3.61532	-3.27042	-1.92422
C	-1.76770	-4.93207	0.28452
H	-0.38574	-2.47387	2.60881
H	0.94905	-2.31049	1.38837
H	0.16587	-3.90352	1.66436
H	-3.55869	5.20056	-0.34506
H	-4.61052	-2.82338	-1.71512
H	-3.72399	-4.36840	-1.85061
H	-3.35603	-3.03083	-2.97708
H	-0.77478	-5.38636	0.08042
H	-2.50941	-5.47716	-0.32858
H	-2.01642	-5.12146	1.34995
H	-0.35595	0.13984	2.25484

TS-27

B	-1.70770	-0.20550	-0.20154
O	-2.65114	-0.25389	0.77909
O	-2.15623	0.20295	-1.42997
C	-3.96684	-0.05658	0.12053
C	-3.56488	0.65013	-1.24654
C	-4.56703	-1.45676	-0.06406
C	-4.84132	0.78533	1.04907
C	-4.36798	0.20291	-2.46674
C	-3.52215	2.18108	-1.15836
H	-4.62877	-1.95697	0.92276
H	-5.58981	-1.40657	-0.48760
H	-3.94392	-2.08846	-0.72836
H	-5.81597	1.01135	0.57025
H	-5.04577	0.22242	1.98150
H	-4.35627	1.73979	1.32698
H	-5.43999	0.45891	-2.34170
H	-4.00011	0.72501	-3.37260
H	-4.28634	-0.88574	-2.64300
H	-4.54137	2.60822	-1.07661
H	-2.92958	2.52734	-0.28774
H	-3.05735	2.58773	-2.07882
Ge	0.27893	-0.75993	0.16762
C	1.77450	-1.28398	-1.02931
C	0.92942	1.31643	0.52886
C	3.14324	-1.06624	-0.69567
C	1.42519	-1.98488	-2.22589
N	1.07680	2.13342	-0.56180
N	1.18377	2.12445	1.60304
C	4.13887	-1.52057	-1.59086
C	3.60509	-0.36743	0.56955
C	2.45372	-2.40646	-3.09282
C	-0.01167	-2.29760	-2.58323
C	0.87774	1.72215	-1.95571
C	1.44521	3.43067	-0.18488
C	1.51129	3.42451	1.19540
C	1.14186	1.75808	3.01861
H	5.19935	-1.36096	-1.33553
C	3.80211	-2.17339	-2.78313
H	3.70680	0.72934	0.41773
H	2.91577	-0.51422	1.42322
H	4.60415	-0.73909	0.87430
H	2.18684	-2.93634	-4.02160
H	-0.48632	-2.93434	-1.80410

H	-0.63925	-1.38635	-2.67714
H	-0.07356	-2.84411	-3.54427
H	-0.07403	1.16531	-2.05343
H	1.71599	1.08992	-2.30613
H	0.81283	2.62383	-2.58964
C	1.69372	4.54030	-1.15535
C	1.84298	4.51675	2.15960
H	0.80581	0.71469	3.11822
H	0.43788	2.42302	3.55701
H	2.14828	1.86842	3.47060
H	4.59364	-2.51692	-3.46756
H	2.50410	4.29527	-1.87393
H	2.00020	5.45463	-0.61328
H	0.78679	4.79753	-1.74349
H	0.99645	4.74117	2.84327
H	2.08456	5.44933	1.61608
H	2.72044	4.26347	2.79166
O	0.51741	-1.05901	2.20317
C	0.63729	-2.32753	2.30898
C	0.11496	-3.06389	3.49390
H	1.31391	-2.88786	1.62696
C	-0.26979	-4.52289	3.19432
H	-0.71365	-2.48459	3.95085
H	0.95555	-3.04759	4.23400
H	-0.57237	-5.03610	4.12759
H	-1.11808	-4.57341	2.48319
H	0.57864	-5.08904	2.75817
H	-0.20025	-2.38912	0.45154

INT-26

B	1.75738	0.06005	0.05613
O	2.67142	0.63632	0.88556
O	2.26410	-0.86869	-0.81654
C	4.01691	0.18253	0.44850
C	3.67984	-1.10746	-0.42259
C	4.62043	1.33705	-0.36131
C	4.85249	-0.08639	1.70006
C	4.51125	-1.26003	-1.69550
C	3.68216	-2.41355	0.38286
H	4.64003	2.24978	0.26672
H	5.66083	1.11214	-0.66921
H	4.02934	1.55983	-1.27165
H	5.84811	-0.49070	1.42553
H	5.01331	0.86084	2.25210
H	4.35963	-0.80027	2.38608
H	5.58556	-1.37685	-1.44612
H	4.19460	-2.16780	-2.24726
H	4.40329	-0.39181	-2.37203
H	4.71084	-2.70844	0.67022
H	3.07437	-2.32981	1.30600
H	3.25601	-3.22675	-0.23814
Ge	-0.28840	0.47337	0.18288
C	-1.18233	1.67627	-1.10952
C	-1.19263	-1.33193	0.05797
C	-2.59774	1.84655	-1.03650
C	-0.41919	2.39167	-2.08071
N	-1.23417	-2.04946	-1.10055
N	-1.80132	-2.10087	1.00134
C	-3.22617	2.71829	-1.95169
C	-3.45400	1.12676	-0.01426
C	-1.09221	3.24853	-2.97735
C	1.09021	2.28725	-2.17883
C	-0.64029	-1.60199	-2.36578

C	-1.87450	-3.27450	-0.89707
C	-2.23703	-3.30498	0.44118
C	-1.99850	-1.74906	2.41128
H	-4.31766	2.85919	-1.89417
C	-2.48374	3.40955	-2.91836
H	-3.65589	0.07616	-0.32040
H	-2.97008	1.10357	0.98312
H	-4.43894	1.62147	0.09466
H	-0.50786	3.80573	-3.72719
H	1.58639	2.59533	-1.23376
H	1.43056	1.25528	-2.40867
H	1.47912	2.94598	-2.97956
H	0.46292	-1.55785	-2.26601
H	-1.03090	-0.60003	-2.63085
H	-0.90949	-2.31202	-3.16643
C	-2.08213	-4.28820	-1.97523
C	-2.94971	-4.34927	1.23612
H	-1.54730	-0.75534	2.59700
H	-1.51799	-2.51154	3.05538
H	-3.08198	-1.71683	2.64057
H	-2.99053	4.08730	-3.62321
H	-2.69756	-3.89047	-2.80988
H	-2.61255	-5.16934	-1.56848
H	-1.12205	-4.64880	-2.40118
H	-2.32804	-4.72708	2.07536
H	-3.20499	-5.21393	0.59576
H	-3.89639	-3.96433	1.67079
O	-0.71617	0.94681	1.89211
C	-0.12198	2.15146	2.42806
C	-0.53581	2.32631	3.88835
H	-0.45696	3.03385	1.83090
C	-2.03954	2.52954	4.10673
H	0.02611	3.20205	4.28269
H	-0.17361	1.44708	4.46663
H	-2.27215	2.67517	5.18119
H	-2.41005	3.42320	3.56082
H	-2.62549	1.65585	3.75418
H	0.98821	2.09255	2.35257
**INT-20**			
Ge	0.51329	-0.35302	0.10561
N	0.92948	2.58201	-0.84578
N	2.34150	2.06245	0.71905
C	2.14494	-1.56505	0.25377
C	1.41294	1.52286	-0.12953
C	3.28272	-1.55151	-0.61203
C	2.05301	-2.55045	1.28831
C	4.29001	-2.52694	-0.43334
H	5.16591	-2.52209	-1.10275
C	-0.13119	2.52478	-1.85104
H	-0.36547	1.47217	-2.07752
H	0.20539	3.01609	-2.78475
H	-1.04059	3.04021	-1.48129
C	3.08791	-3.49909	1.43571
H	3.01610	-4.25541	2.23413
C	4.19705	-3.49271	0.57831
H	4.99346	-4.24385	0.69941
C	1.55509	3.77516	-0.46876
C	2.45096	3.44527	0.53293
C	1.23996	5.09052	-1.10195
H	1.47058	5.09438	-2.18886
H	1.83844	5.89477	-0.63490
H	0.16925	5.36179	-0.98692

C	3.10765	1.33401	1.73076
H	2.99223	0.24735	1.56326
H	2.75229	1.59458	2.74837
H	4.18121	1.59182	1.65034
C	3.39274	4.29738	1.31891
H	3.20058	4.22940	2.41056
H	3.28350	5.35883	1.02802
H	4.45340	4.01420	1.14833
C	0.87938	-2.61075	2.25201
H	1.01002	-3.43277	2.98288
H	0.76066	-1.67277	2.83594
H	-0.08828	-2.78292	1.73325
C	3.50233	-0.50880	-1.69140
H	2.56991	-0.20838	-2.20423
H	3.94749	0.41745	-1.26521
H	4.21295	-0.87608	-2.45859
O	0.27695	-0.66233	-1.98813
C	-0.15374	-1.70480	-2.53141
C	-0.36930	-2.99715	-1.82687
H	-0.40254	-1.62833	-3.61986
C	-1.02356	-4.09860	-2.66619
H	-0.95837	-2.75243	-0.90472
H	0.62328	-3.30990	-1.41756
H	-1.14736	-5.02026	-2.06572
H	-2.02808	-3.79694	-3.02613
H	-0.40359	-4.35796	-3.54842

TS-28

Ge	0.30867	-0.31196	0.61918
N	0.99456	2.48581	-0.82532
N	2.24419	1.98815	0.87545
C	1.87685	-1.58720	0.27920
C	1.28816	1.47299	0.04207
C	2.81577	-1.51994	-0.79612
C	2.02660	-2.60463	1.27500
C	3.88529	-2.44262	-0.84240
H	4.60609	-2.39042	-1.67526
C	0.06145	2.40784	-1.94707
H	-0.49339	1.45547	-1.88810
H	0.61217	2.45145	-2.90907
H	-0.64990	3.25587	-1.90874
C	3.10795	-3.50934	1.19068
H	3.21615	-4.29315	1.95826
C	4.03695	-3.42652	0.14458
H	4.87520	-4.13917	0.09112
C	1.76676	3.62545	-0.55340
C	2.55733	3.31100	0.53545
C	1.67250	4.87689	-1.36371
H	1.91916	4.70075	-2.43245
H	2.38026	5.63635	-0.98177
H	0.65549	5.32270	-1.32707
C	2.82020	1.27697	2.01562
H	2.81015	0.18892	1.81127
H	2.25202	1.48912	2.94515
H	3.87149	1.58809	2.15887
C	3.56990	4.12622	1.27207
H	3.31443	4.23724	2.34724
H	3.62993	5.14251	0.83941
H	4.58722	3.68282	1.21324
C	1.06508	-2.75257	2.44421
H	1.23411	-3.71157	2.97301
H	1.18951	-1.94263	3.19567
H	0.00055	-2.72515	2.13000

C	2.72188	-0.49945	-1.91453
H	1.67280	-0.26581	-2.17997
H	3.21169	0.45837	-1.63386
H	3.23659	-0.86407	-2.82655
O	-0.72480	-0.53542	-1.22136
C	-1.25535	-1.56152	-1.90216
B	-2.75600	-0.71281	-0.84122
C	-0.92451	-3.01933	-1.59697
H	-1.35484	-1.35659	-2.99737
H	-2.60118	-1.62952	-1.76032
O	-2.93484	-1.18020	0.41835
O	-3.40489	0.40617	-1.24592
C	0.28282	-3.52499	-2.40246
H	-1.81727	-3.63039	-1.85474
H	-0.76354	-3.13113	-0.50498
C	-4.02547	-0.32617	0.97255
C	-3.98920	0.95627	0.01468
H	0.43988	-4.60201	-2.19493
H	0.11748	-3.41626	-3.49459
H	1.21403	-2.99515	-2.12656
C	-5.31402	-1.14688	0.84664
C	-3.70096	-0.03894	2.43640
C	-5.35901	1.53642	-0.32847
C	-3.03692	2.05322	0.50190
H	-5.18299	-2.11078	1.37710
H	-6.17377	-0.61850	1.30417
H	-5.56432	-1.36769	-0.21056
H	-4.44777	0.65793	2.86883
H	-3.74088	-0.98051	3.01927
H	-2.69102	0.39499	2.55716
H	-5.86911	1.89402	0.58909
H	-5.24003	2.40258	-1.00940
H	-6.01103	0.79630	-0.82823
H	-3.44215	2.56339	1.39822
H	-2.03644	1.64628	0.75599
H	-2.91931	2.81094	-0.29813
**INT-27**			
Ge	-0.64570	-0.26043	-0.89535
N	-1.00486	2.47139	0.76709
N	-2.60201	2.01399	-0.62492
C	-1.99262	-1.60065	-0.15105
C	-1.48525	1.48374	-0.03967
C	-2.59202	-1.60047	1.14814
C	-2.34934	-2.62906	-1.08438
C	-3.52392	-2.60769	1.48210
H	-3.97719	-2.61037	2.48709
C	0.16037	2.36133	1.63869
H	0.55376	1.33390	1.57835
H	-0.12503	2.58237	2.68628
H	0.95335	3.06477	1.31836
C	-3.29234	-3.61159	-0.71261
H	-3.56308	-4.39916	-1.43468
C	-3.87798	-3.60303	0.56040
H	-4.60654	-4.38060	0.83942
C	-1.81054	3.61746	0.69977
C	-2.82576	3.32821	-0.19317
C	-1.53015	4.84875	1.49745
H	-1.57546	4.65702	2.59102
H	-2.27616	5.63289	1.26934
H	-0.52634	5.26846	1.27434
C	-3.42414	1.32190	-1.61642
H	-3.33919	0.22852	-1.46142

H	-3.10848	1.58174	-2.64809
H	-4.48622	1.60085	-1.48494
C	-3.97513	4.15745	-0.66562
H	-3.97128	4.28484	-1.76883
H	-3.92849	5.16675	-0.21540
H	-4.95447	3.71549	-0.38182
C	-1.76747	-2.70153	-2.48798
H	-1.99657	-3.67838	-2.95795
H	-2.18449	-1.91805	-3.15774
H	-0.66434	-2.57538	-2.50419
C	-2.28850	-0.55629	2.20590
H	-1.22572	-0.24549	2.21023
H	-2.89558	0.36240	2.05307
H	-2.53270	-0.93669	3.21813
O	1.04061	-0.79906	0.60417
C	1.03082	-2.07692	1.34176
B	2.28810	-0.26324	0.24071
C	1.30972	-3.27932	0.44758
H	0.02578	-2.14456	1.80016
H	1.78831	-1.98822	2.14778
O	3.47612	-0.88623	0.49290
O	2.36434	0.95510	-0.37744
C	1.27541	-4.59038	1.24630
H	2.30223	-3.15191	-0.03339
H	0.54854	-3.31016	-0.36187
C	4.53241	0.09219	0.15502
C	3.76764	1.08546	-0.83093
H	1.47662	-5.45657	0.58521
H	2.04240	-4.60058	2.04901
H	0.28536	-4.75598	1.72016
C	4.95890	0.74823	1.47519
C	5.70223	-0.66282	-0.47393
C	4.18006	2.55157	-0.71166
C	3.79816	0.63247	-2.29675
H	5.28612	-0.03934	2.18274
H	5.80520	1.44769	1.32615
H	4.12472	1.30681	1.94647
H	6.48708	0.04456	-0.81104
H	6.15770	-1.34086	0.27512
H	5.38468	-1.27444	-1.33899
H	5.24913	2.67787	-0.97811
H	3.58383	3.16834	-1.41371
H	4.02763	2.94603	0.31072
H	4.80491	0.76352	-2.74054
H	3.50604	-0.43148	-2.40501
H	3.08175	1.24351	-2.88087
**INT-28**			
B	1.28114	-1.75658	-1.14631
O	2.41789	-2.44344	-1.39798
O	1.43439	-0.77205	-0.16261
C	3.43534	-2.05946	-0.40109
C	2.92287	-0.64450	0.09914
C	3.40847	-3.14483	0.68433
C	4.79722	-2.02796	-1.09496
C	3.16525	-0.36932	1.58002
C	3.40817	0.52569	-0.76095
H	3.56879	-4.13222	0.20827
H	4.21018	-2.99099	1.43317
H	2.43595	-3.17599	1.21609
H	5.58467	-1.67369	-0.39914
H	5.06841	-3.05137	-1.42126
H	4.79452	-1.37720	-1.98892

H	4.25516	-0.40795	1.78417
H	2.81722	0.64665	1.85432
H	2.66282	-1.10028	2.23990
H	4.48349	0.72088	-0.57930
H	3.27322	0.32988	-1.84313
H	2.84558	1.44335	-0.49846
Ge	-0.14697	0.34196	1.10749
C	-0.00480	2.12130	0.13980
C	-1.81775	-0.56906	0.20431
C	0.12099	3.23828	1.03048
C	0.10736	2.33803	-1.27051
N	-2.97832	-0.05345	-0.31040
N	-2.09849	-1.89192	0.43748
C	0.36540	4.52496	0.50464
C	-0.05309	3.10676	2.53385
C	0.34769	3.64291	-1.75367
C	-0.01664	1.22754	-2.29194
C	-3.21463	1.34058	-0.68710
C	-3.96267	-1.03838	-0.42318
C	-3.39814	-2.21408	0.04590
C	-1.17039	-2.85452	1.02454
H	0.45980	5.37929	1.19468
C	0.48346	4.72844	-0.87684
H	-1.12766	3.06949	2.81893
H	0.41603	2.18725	2.94568
H	0.38831	3.97361	3.06441
H	0.42924	3.80713	-2.84092
H	0.97847	0.81103	-2.55877
H	-0.63125	0.38119	-1.92921
H	-0.46147	1.60273	-3.23575
H	-4.06881	1.74742	-0.11065
H	-3.44584	1.41080	-1.76834
H	-2.30959	1.93418	-0.46912
C	-5.32961	-0.76089	-0.95682
C	-3.96680	-3.59077	0.15741
H	-0.36149	-2.29977	1.53885
H	-0.73199	-3.51146	0.24638
H	-1.69912	-3.47985	1.76885
H	0.67626	5.73800	-1.27283
H	-5.87644	-0.02355	-0.33124
H	-5.93110	-1.68872	-0.97681
H	-5.29777	-0.35999	-1.99193
H	-3.35269	-4.33733	-0.38903
H	-4.98607	-3.62127	-0.27066
H	-4.04187	-3.92697	1.21383
H	0.22569	-1.96701	-1.68903
	**INT-29**		
C	-3.88195	0.00570	2.60898
O	-3.02800	0.24111	1.76240
H	-1.59989	-2.06725	1.05148
B	-1.93982	-1.52655	0.03041
O	-3.03975	-1.85561	-0.69062
O	-1.09961	-0.60801	-0.63567
C	-2.95025	-1.23559	-2.01869
C	-1.89422	-0.06605	-1.80963
C	-2.47267	-2.33731	-2.97748
C	-4.34612	-0.75425	-2.41928
C	-0.95462	0.15334	-2.98981
C	-2.51895	1.25608	-1.36035
H	-3.16968	-3.19581	-2.91008
H	-2.45709	-1.98847	-4.02889
H	-1.46006	-2.70464	-2.71461

H	-4.31828	-0.22654	-3.39426
H	-5.01967	-1.62767	-2.52593
H	-4.78214	-0.07666	-1.66220
H	-1.54729	0.41588	-3.88999
H	-0.27308	1.00095	-2.78469
H	-0.35256	-0.74337	-3.22898
H	-3.10252	1.70652	-2.18749
H	-3.18032	1.12219	-0.48411
H	-1.71754	1.97036	-1.08761
C	-4.75638	-1.22148	2.65269
C	-6.21368	-0.91696	3.03970
H	-4.30034	-1.88184	3.43053
H	-4.67235	-1.75998	1.68631
H	-6.79246	-1.85556	3.14474
H	-6.27639	-0.37679	4.00752
H	-6.71736	-0.29562	2.27114
H	-4.04713	0.74390	3.45020
Ge	0.46668	0.28629	0.87778
C	0.88425	2.03583	-0.08033
C	2.23541	-0.79581	0.41746
C	1.70614	2.25702	-1.23211
C	0.26043	3.15949	0.56009
N	3.39888	-0.47669	1.06220
N	2.44086	-2.04894	-0.08017
C	1.87986	3.57351	-1.71447
C	2.43567	1.15380	-1.97547
C	0.46714	4.45761	0.04596
C	-0.61517	3.01905	1.79617
C	3.62481	0.76234	1.80486
C	4.33343	-1.51767	0.97000
C	3.72492	-2.51776	0.23466
C	1.47718	-2.80998	-0.86836
H	2.50548	3.74102	-2.60666
C	1.26976	4.66638	-1.08342
H	3.41244	0.92059	-1.49851
H	1.86244	0.20911	-2.01395
H	2.65755	1.46197	-3.01712
H	-0.01648	5.31570	0.54053
H	-1.37756	2.21578	1.70741
H	-0.01464	2.79171	2.70401
H	-1.15246	3.96685	1.99913
H	4.65499	1.12594	1.62978
H	3.47930	0.60504	2.89363
H	2.92135	1.53766	1.44363
C	5.69363	-1.44643	1.58346
C	4.22615	-3.85463	-0.20390
H	0.56220	-2.20527	-0.99105
H	1.22035	-3.75951	-0.35733
H	1.89393	-3.04395	-1.86855
H	1.41620	5.68438	-1.47792
H	6.30455	-0.62435	1.15245
H	6.24262	-2.39053	1.40783
H	5.64654	-1.28804	2.68171
H	3.59825	-4.68334	0.18713
H	5.25671	-4.01834	0.16300
H	4.24984	-3.94832	-1.31078

INT-30

C	-2.18434	-3.44294	0.88869
O	-1.52585	-2.22415	0.45661
H	-3.12794	-3.57390	0.31761
B	-2.23206	-1.18304	-0.03692
O	-3.57666	-1.12475	-0.26414

O	-1.55086	0.01048	-0.41335
C	-3.85651	0.08852	-1.03751
C	-2.63553	1.03340	-0.66307
C	-3.87973	-0.31998	-2.51840
C	-5.22218	0.62757	-0.60986
C	-2.19878	1.97226	-1.78105
C	-2.84682	1.79787	0.64720
H	-4.61843	-1.13473	-2.65241
H	-4.17788	0.52338	-3.17195
H	-2.89359	-0.69494	-2.86070
H	-5.43952	1.59505	-1.10637
H	-6.01155	-0.09039	-0.90858
H	-5.28779	0.76606	0.48541
H	-3.04087	2.64258	-2.04959
H	-1.35729	2.60997	-1.44717
H	-1.89151	1.42618	-2.69224
H	-3.62797	2.57353	0.52368
H	-3.15143	1.12837	1.47639
H	-1.91066	2.31857	0.93048
C	-2.45458	-3.43668	2.39199
C	-1.19931	-3.30108	3.26133
H	-2.98538	-4.38610	2.62735
H	-3.17806	-2.62322	2.62273
H	-1.45850	-3.31423	4.33921
H	-0.48890	-4.13561	3.07868
H	-0.66634	-2.35020	3.05252
H	-1.49531	-4.27174	0.61950
Ge	0.36654	0.30263	0.91327
C	0.92290	2.18551	0.34262
C	1.79598	-0.77024	-0.21747
C	1.34404	2.61646	-0.95488
C	0.89267	3.13521	1.41699
N	3.06381	-0.84956	0.28943
N	1.73951	-1.73018	-1.18316
C	1.71851	3.96335	-1.15200
C	1.42122	1.68842	-2.15068
C	1.28032	4.47196	1.17725
C	0.48651	2.76499	2.83566
C	3.56124	-0.02882	1.39245
C	3.80388	-1.85427	-0.34770
C	2.96099	-2.41187	-1.29118
C	0.59835	-1.99399	-2.05342
H	2.03393	4.29357	-2.15548
C	1.69088	4.88611	-0.09633
H	2.37414	1.11581	-2.15617
H	0.59443	0.95342	-2.16806
H	1.38567	2.26082	-3.09942
H	1.25207	5.19766	2.00648
H	-0.41263	2.11546	2.87624
H	1.29320	2.21813	3.37131
H	0.26859	3.67362	3.43119
H	4.64102	0.16730	1.25716
H	3.40613	-0.53404	2.36833
H	3.03713	0.94718	1.38674
C	5.22276	-2.16801	-0.00083
C	3.19020	-3.50391	-2.28415
H	-0.19667	-1.26381	-1.83007
H	0.20754	-3.01613	-1.88335
H	0.89880	-1.89184	-3.11520
H	1.98304	5.93383	-0.27011
H	5.90029	-1.30831	-0.19296
H	5.58699	-3.01461	-0.61252

H	5.33762	-2.45490	1.06585
H	2.47628	-4.34408	-2.14994
H	4.21113	-3.91519	-2.17454
H	3.08880	-3.14489	-3.33083

TS-29

C	-2.65677	-1.23413	2.16898
O	-2.89568	-0.21090	1.42884
H	-1.84482	-2.11453	0.76463
B	-2.30981	-1.10260	0.14169
O	-3.35889	-1.37326	-0.73984
O	-1.26079	-0.33598	-0.54744
C	-3.08332	-0.76397	-2.02659
C	-1.98846	0.33797	-1.69956
C	-2.57425	-1.87126	-2.96658
C	-4.39016	-0.17967	-2.57577
C	-0.99880	0.59923	-2.82760
C	-2.58522	1.65199	-1.18556
H	-3.32202	-2.68859	-2.98343
H	-2.43745	-1.50910	-4.00514
H	-1.61567	-2.30236	-2.61521
H	-4.22003	0.36244	-3.52862
H	-5.10146	-1.00508	-2.77819
H	-4.86785	0.50729	-1.85300
H	-1.55271	0.92375	-3.73237
H	-0.30503	1.41700	-2.55491
H	-0.41251	-0.29998	-3.09475
H	-3.07211	2.20036	-2.01639
H	-3.33604	1.48843	-0.38977
H	-1.77765	2.29712	-0.78792
C	-3.73994	-2.13528	2.66248
C	-4.29729	-1.52677	3.97907
H	-3.31838	-3.14247	2.86306
H	-4.54029	-2.20922	1.89971
H	-5.08501	-2.19603	4.37742
H	-3.51246	-1.42635	4.75633
H	-4.74598	-0.53037	3.79819
H	-1.66000	-1.29544	2.68267
Ge	0.34357	0.23933	0.87617
C	0.79942	2.10662	0.19867
C	1.99813	-0.86309	0.16809
C	1.65219	2.48797	-0.88641
C	0.19411	3.13067	1.00244
N	3.22237	-0.69204	0.75491
N	2.08725	-2.05288	-0.49275
C	1.87179	3.86106	-1.14257
C	2.37203	1.50702	-1.79272
C	0.44469	4.48809	0.71116
C	-0.72011	2.81786	2.17684
C	3.58002	0.42223	1.63125
C	4.08087	-1.76004	0.45886
C	3.35921	-2.62459	-0.34275
C	1.01667	-2.66009	-1.27637
H	2.52114	4.15164	-1.98480
C	1.27841	4.85471	-0.35389
H	3.35928	1.21472	-1.37265
H	1.80320	0.57384	-1.95654
H	2.57486	1.96432	-2.78214
H	-0.02886	5.26763	1.32984
H	-1.52014	2.09284	1.91779
H	-0.16067	2.38766	3.03573
H	-1.21379	3.74031	2.54176
H	4.61127	0.75909	1.41373

H	3.51780	0.12276	2.69794
H	2.89400	1.27107	1.44518
C	5.48463	-1.83841	0.96374
C	3.74281	-3.91852	-0.98260
H	0.11383	-2.02977	-1.18966
H	0.79167	-3.67737	-0.89875
H	1.30909	-2.73402	-2.34342
H	1.46111	5.91840	-0.57396
H	6.11094	-1.00024	0.58955
H	5.96026	-2.77912	0.62847
H	5.52955	-1.81961	2.07331
H	3.10496	-4.75990	-0.63706
H	4.79027	-4.17415	-0.73577
H	3.66406	-3.87458	-2.08989
	**TS-30**		
B	0.62854	-1.44373	-0.75024
O	1.13107	-2.61887	-0.26129
O	1.27233	-0.97562	-1.88327
C	2.38213	-2.91744	-0.98519
C	2.27425	-1.98677	-2.28108
C	3.54354	-2.54902	-0.05338
C	2.39842	-4.42003	-1.27833
C	3.56814	-1.26026	-2.65381
C	1.69530	-2.70479	-3.50882
H	3.46547	-3.15095	0.87423
H	4.52374	-2.77220	-0.51967
H	3.50834	-1.47771	0.22533
H	3.29442	-4.69656	-1.87071
H	2.43749	-4.98303	-0.32450
H	1.49691	-4.74882	-1.82829
H	4.36505	-1.98595	-2.91540
H	3.39620	-0.61796	-3.54121
H	3.93414	-0.62075	-1.82916
H	2.39886	-3.46281	-3.90694
H	0.73627	-3.20871	-3.27622
H	1.50767	-1.96395	-4.31223
Ge	-1.37129	-0.87262	-0.54267
C	-2.65850	-1.34989	0.88712
C	-1.50980	1.12546	-0.83612
C	-4.05720	-1.20985	0.63056
C	-2.20935	-1.88917	2.12701
N	-1.75016	2.11392	0.06799
N	-1.42624	1.74012	-2.05022
C	-4.97502	-1.60842	1.62667
C	-4.60492	-0.66307	-0.67205
C	-3.16306	-2.27517	3.09364
C	-0.73982	-2.06138	2.43159
C	-1.84814	1.93410	1.51738
C	-1.82681	3.35664	-0.56918
C	-1.62573	3.11848	-1.91982
C	-1.16629	1.04482	-3.31499
H	-6.05460	-1.50065	1.43315
C	-4.53578	-2.13827	2.84778
H	-4.16688	0.32273	-0.93711
H	-4.39385	-1.34520	-1.52334
H	-5.70316	-0.53271	-0.61549
H	-2.81561	-2.69171	4.05290
H	-0.28419	-2.86561	1.81555
H	-0.16405	-1.13894	2.20596
H	-0.58015	-2.31874	3.49723
H	-0.90016	2.23622	2.00516
H	-2.05368	0.87085	1.73744

H	-2.68078	2.54481	1.91400
C	-2.08982	4.62932	0.16805
C	-1.61828	4.06480	-3.07657
H	-2.09080	0.56721	-3.69695
H	-0.38553	0.27563	-3.15967
H	-0.80435	1.77515	-4.06031
H	-5.26782	-2.44468	3.61158
H	-3.08826	4.62763	0.65554
H	-2.06442	5.48895	-0.52737
H	-1.33346	4.81600	0.95919
H	-0.64031	4.07732	-3.60349
H	-1.81559	5.09520	-2.72615
H	-2.40037	3.81421	-3.82396
H	-2.04665	-1.33551	-1.87372
C	1.93749	0.52604	1.00385
N	2.34178	1.74131	0.50378
N	2.56735	0.48873	2.22561
C	1.96839	2.22324	-0.81794
C	3.18240	2.44871	1.38061
C	3.32726	1.64060	2.49057
C	2.50419	-0.64816	3.13524
H	1.23524	3.05680	-0.75835
H	1.53372	1.37489	-1.37635
H	2.86027	2.58813	-1.36697
C	3.76596	3.78855	1.06429
C	4.11149	1.83751	3.74819
H	3.52401	-0.97104	3.42743
H	2.00379	-1.48113	2.61278
H	1.93806	-0.40075	4.05768
H	4.44777	3.75381	0.18642
H	4.35705	4.16552	1.92106
H	2.98495	4.54756	0.84188
H	3.47213	1.77616	4.65502
H	4.59673	2.83251	3.75194
H	4.91615	1.07904	3.86363
**TS-31**			
B	2.24703	0.02579	-0.37425
O	2.92737	-1.13976	-0.06116
O	2.22248	0.29368	-1.74084
C	3.23190	-1.84902	-1.31248
C	3.08961	-0.69372	-2.40617
C	4.64302	-2.43147	-1.18405
C	2.21265	-2.98617	-1.46540
C	4.41077	0.02920	-2.71126
C	2.41412	-1.13392	-3.70638
H	4.65475	-3.20829	-0.39336
H	4.95586	-2.91421	-2.13216
H	5.39176	-1.66164	-0.91872
H	2.44443	-3.61541	-2.34783
H	2.25459	-3.63272	-0.56617
H	1.17744	-2.60759	-1.56707
H	5.11422	-0.62080	-3.26870
H	4.20193	0.92014	-3.33702
H	4.91437	0.37280	-1.78550
H	3.01079	-1.92098	-4.21092
H	1.39586	-1.52752	-3.52733
H	2.33919	-0.27347	-4.40186
Ge	-0.60315	-0.70071	-0.20327
C	-1.58353	-2.11475	0.88627
C	-2.15061	0.56374	-0.69063
C	-2.63769	-2.90974	0.34050
C	-1.15356	-2.35755	2.22443

N	-3.13401	1.08495	0.10486
N	-2.28756	1.20998	-1.89153
C	-3.24242	-3.90415	1.14334
C	-3.13693	-2.74253	-1.07936
C	-1.78284	-3.35828	2.99674
C	-0.01446	-1.57426	2.84898
C	-3.36323	0.72349	1.50112
C	-3.89421	2.03769	-0.58776
C	-3.36032	2.11383	-1.86085
C	-1.46777	0.93091	-3.06878
H	-4.05584	-4.51467	0.71751
C	-2.82489	-4.12806	2.46201
H	-3.47499	-1.70413	-1.28164
H	-2.33661	-2.95616	-1.81918
H	-3.98600	-3.42347	-1.28711
H	-1.44365	-3.53695	4.03059
H	0.92061	-1.69928	2.26421
H	-0.22450	-0.48280	2.88129
H	0.17989	-1.91346	3.88671
H	-3.03392	1.53901	2.17787
H	-2.80239	-0.20218	1.72764
H	-4.43986	0.53019	1.67180
C	-5.04920	2.75550	0.03155
C	-3.77868	2.93514	-3.03763
H	-1.82562	0.02005	-3.59163
H	-0.41636	0.76781	-2.76636
H	-1.51689	1.78906	-3.76357
H	-3.30882	-4.90731	3.07199
H	-5.86375	2.05813	0.32365
H	-5.47923	3.48302	-0.68230
H	-4.75322	3.31715	0.94311
H	-2.97440	3.61940	-3.38435
H	-4.65079	3.56244	-2.77330
H	-4.07610	2.30431	-3.90233
H	-0.62171	-1.39207	-1.63172
C	2.13688	1.18199	0.71118
N	1.64742	2.44199	0.51274
N	2.55351	1.14529	2.01259
C	1.04978	2.92031	-0.73037
C	1.70996	3.18550	1.69216
C	2.29007	2.36355	2.64413
C	3.24898	0.03811	2.67308
H	-0.05604	2.83881	-0.67787
H	1.42367	2.30096	-1.56463
H	1.33366	3.97648	-0.89634
C	1.23531	4.59864	1.78714
C	2.63885	2.62058	4.07325
H	4.26315	0.36474	2.97947
H	3.33354	-0.80376	1.96498
H	2.68668	-0.27975	3.57201
H	1.81617	5.27743	1.12602
H	1.34821	4.97222	2.82201
H	0.16471	4.69907	1.50998
H	2.13172	1.90864	4.75839
H	2.33397	3.64219	4.36735
H	3.73129	2.53395	4.25559

INT-31

B	-1.56495	0.27316	-0.41172
O	-2.28754	1.51845	-0.36832
O	-1.80832	-0.34518	-1.70522
C	-3.28956	1.50214	-1.42316
C	-2.64046	0.54065	-2.51188

C	-4.60205	0.93929	-0.84274
C	-3.51300	2.94400	-1.89076
C	-3.64758	-0.32855	-3.27615
C	-1.75399	1.29388	-3.51902
H	-4.92519	1.56814	0.01142
H	-5.42089	0.94993	-1.58979
H	-4.48244	-0.10311	-0.48266
H	-4.20899	2.98170	-2.75388
H	-3.96206	3.53916	-1.06992
H	-2.56362	3.43234	-2.17953
H	-4.36913	0.29856	-3.83932
H	-3.11369	-0.96438	-4.01177
H	-4.21490	-0.99607	-2.60039
H	-2.36297	1.91160	-4.20936
H	-1.02066	1.95347	-3.01729
H	-1.19474	0.56173	-4.13685
Ge	0.65771	0.84962	-0.45195
C	1.79571	2.15810	0.57422
C	1.88783	-0.78426	-0.60584
C	3.04498	2.60321	0.04205
C	1.32994	2.69485	1.80877
N	2.70245	-1.35198	0.33216
N	1.99097	-1.58639	-1.70736
C	3.80458	3.54702	0.76989
C	3.59069	2.12459	-1.28747
C	2.11808	3.63538	2.50619
C	-0.01331	2.29916	2.38179
C	2.90711	-0.85557	1.69211
C	3.32850	-2.49754	-0.17520
C	2.87908	-2.64433	-1.47657
C	1.29651	-1.34303	-2.97422
H	4.76816	3.88921	0.35802
C	3.35233	4.05761	1.99393
H	3.69604	1.01967	-1.32811
H	2.92025	2.40648	-2.12701
H	4.58742	2.56364	-1.48898
H	1.75003	4.04574	3.46076
H	-0.83461	2.57273	1.68719
H	-0.08440	1.20273	2.55119
H	-0.20186	2.80341	3.35055
H	2.44082	-1.53828	2.43119
H	2.46498	0.15379	1.77817
H	3.99026	-0.77907	1.90805
C	4.29447	-3.31155	0.62291
C	3.22556	-3.66839	-2.50883
H	1.84814	-0.60004	-3.58562
H	0.27612	-0.96393	-2.77260
H	1.22499	-2.29090	-3.53765
H	3.95951	4.79284	2.54528
H	5.19884	-2.72819	0.89967
H	4.63370	-4.18822	0.03995
H	3.84471	-3.69055	1.56499
H	2.33826	-4.24683	-2.84430
H	3.95386	-4.39260	-2.09802
H	3.68625	-3.21035	-3.40959
H	0.85590	1.33192	-1.92833
C	-1.87185	-0.76775	0.83073
N	-1.57288	-2.10123	0.86589
N	-2.43157	-0.49567	2.04797
C	-1.00883	-2.86997	-0.24185
C	-1.92053	-2.66446	2.09896
C	-2.46834	-1.64260	2.85100

C	-3.02829	0.77625	2.46365
H	0.05600	-3.11567	-0.05036
H	-1.11494	-2.27309	-1.16538
H	-1.57458	-3.81433	-0.36115
C	-1.72099	-4.11066	2.41675
C	-3.03923	-1.64319	4.23134
H	-4.11041	0.63165	2.65556
H	-2.89535	1.50341	1.64354
H	-2.54589	1.14046	3.39098
H	-2.33269	-4.76943	1.76305
H	-2.01846	-4.31858	3.46168
H	-0.66137	-4.42318	2.30254
H	-2.51547	-0.92683	4.89924
H	-2.95069	-2.64822	4.68478
H	-4.11630	-1.37081	4.23555
**INT-33**			
B	4.04376	0.28718	-0.34580
O	5.06787	0.18193	0.55207
O	3.63225	1.56141	-0.61611
C	5.27052	1.53747	1.13067
C	4.61824	2.47399	0.02292
C	4.51814	1.54888	2.46746
C	6.76809	1.74433	1.34738
C	3.86378	3.68512	0.56740
C	5.59516	2.89095	-1.08387
H	4.90358	0.73397	3.11192
H	4.66980	2.50614	3.00411
H	3.42845	1.39567	2.33218
H	6.96753	2.77426	1.70677
H	7.13811	1.04179	2.12044
H	7.35128	1.57638	0.42284
H	4.55454	4.35384	1.11999
H	3.43105	4.26788	-0.26999
H	3.04000	3.39412	1.24550
H	6.33200	3.62912	-0.71033
H	6.15040	2.02434	-1.49602
H	5.03071	3.36424	-1.91174
C	3.38101	-0.97737	-1.02468
N	3.70812	-2.28796	-0.81663
N	2.36501	-0.98166	-1.93886
C	4.75489	-2.78842	0.08204
C	2.90690	-3.11654	-1.59292
C	2.04978	-2.28405	-2.30688
C	1.67391	0.19772	-2.47280
H	5.52073	-3.33313	-0.50444
H	4.30827	-3.47979	0.82299
H	5.22297	-1.93561	0.60225
C	3.03133	-4.60386	-1.58256
C	0.97562	-2.60956	-3.29070
H	2.12931	1.10758	-2.04645
H	0.60068	0.15566	-2.20144
H	1.76689	0.21425	-3.57624
H	4.04312	-4.93485	-1.89868
H	2.29982	-5.05718	-2.27655
H	2.84194	-5.02642	-0.57314
H	-0.01632	-2.23999	-2.95502
H	0.89559	-3.70377	-3.42681
H	1.17918	-2.16206	-4.28653
**INT-32**			
Ge	-0.37683	-0.12618	-1.24854
C	-1.33601	-1.47574	-0.01438

C	1.45210	-0.01294	-0.30887
C	-1.57240	-1.26672	1.37923
C	-1.86705	-2.65216	-0.62781
N	2.30637	-1.04022	0.00952
N	2.23749	1.11435	-0.25134
C	-2.30655	-2.22162	2.12069
C	-1.06527	-0.03962	2.10749
C	-2.59567	-3.58645	0.14312
C	-1.67655	-2.94227	-2.10676
C	1.92941	-2.44749	0.02113
C	3.60616	-0.57082	0.24694
C	3.56343	0.79864	0.07973
C	1.75708	2.45056	-0.57110
H	-2.48146	-2.04868	3.19642
C	-2.81542	-3.37674	1.51145
H	0.03678	0.06476	2.01585
H	-1.49096	0.88992	1.67332
H	-1.32428	-0.07505	3.18549
H	-2.99839	-4.49042	-0.34486
H	-2.09832	-2.13495	-2.74203
H	-0.60335	-3.01249	-2.38542
H	-2.16601	-3.89625	-2.39161
H	0.82632	-2.51794	0.07511
H	2.36235	-2.95410	0.90580
H	2.28349	-2.96048	-0.89835
C	4.73733	-1.47375	0.61910
C	4.63811	1.82854	0.21390
H	0.67401	2.49689	-0.33987
H	1.89088	2.67648	-1.65081
H	2.30054	3.20480	0.02905
H	-3.38631	-4.11170	2.10234
H	4.56072	-1.99021	1.58807
H	5.67527	-0.89485	0.72066
H	4.91201	-2.26122	-0.14544
H	4.76999	2.41916	-0.71833
H	5.60737	1.34407	0.44005
H	4.43110	2.54952	1.03514
H	-0.89926	1.21726	-0.53522

TS-32

Ge	-0.42876	0.38714	-1.02845
C	-1.50678	-1.03466	-0.03859
C	1.50466	0.05106	-0.14231
C	-1.47976	-1.28004	1.37162
C	-2.44540	-1.74264	-0.85413
N	2.17893	-1.14381	-0.16057
N	2.47895	0.99919	-0.02131
C	-2.37590	-2.23042	1.91761
C	-0.52497	-0.57568	2.31240
C	-3.31265	-2.68931	-0.27049
C	-2.54745	-1.49591	-2.35037
C	1.56363	-2.46060	-0.28916
C	3.56137	-0.94932	-0.04556
C	3.74914	0.41717	0.04462
C	2.23915	2.44584	-0.01293
H	-2.35890	-2.41328	3.00535
C	-3.27971	-2.93351	1.11086
H	0.49055	-1.02936	2.26211
H	-0.40183	0.50717	2.06495
H	-0.86692	-0.67688	3.36288
H	-4.02865	-3.23314	-0.90954
H	-2.76648	-0.42977	-2.57854
H	-1.60735	-1.74680	-2.88773

H	-3.35840	-2.10418	-2.79989
H	0.46389	-2.34800	-0.24421
H	1.88706	-3.11964	0.54121
H	1.84673	-2.93419	-1.25206
C	4.54035	-2.07803	-0.01747
C	5.00080	1.21845	0.19768
H	1.16867	2.62554	0.27762
H	2.48432	2.87627	-1.00703
H	2.88587	2.91668	0.75279
H	-3.96693	-3.66922	1.55974
H	4.37723	-2.75327	0.85063
H	5.57374	-1.68869	0.05688
H	4.48476	-2.70297	-0.93484
H	5.12609	1.95279	-0.62621
H	5.88710	0.55547	0.19538
H	5.01247	1.79025	1.15074
O	-0.39883	2.33031	1.10829
C	-1.62865	2.31261	0.64545
C	-2.29619	3.66524	0.32047
H	-2.36657	1.64286	1.19152
C	-3.72008	3.56278	-0.23422
H	-1.63263	4.22486	-0.37566
H	-2.28462	4.23916	1.27627
H	-4.16529	4.56268	-0.41861
H	-3.74184	3.00864	-1.19779
H	-4.39126	3.02449	0.46948
H	-1.69445	1.68562	-0.46117

INT-34

Ge	-0.50594	0.22400	-1.00814
C	-1.45717	-1.24885	0.11964
C	1.42332	-0.02672	-0.22511
C	-1.45939	-1.29909	1.54683
C	-2.25557	-2.18958	-0.60112
N	2.15359	-1.17854	-0.36934
N	2.34441	0.92632	0.11593
C	-2.24216	-2.26906	2.21464
C	-0.63007	-0.34351	2.38035
C	-3.02062	-3.15082	0.09787
C	-2.31557	-2.19915	-2.12043
C	1.58986	-2.45536	-0.79059
C	3.51589	-0.95407	-0.12937
C	3.63574	0.38545	0.18421
C	2.03651	2.33149	0.37396
H	-2.24090	-2.29360	3.31809
C	-3.01728	-3.19247	1.49926
H	0.44544	-0.62990	2.36098
H	-0.69728	0.68622	1.97352
H	-0.95302	-0.34822	3.44169
H	-3.63394	-3.87030	-0.47131
H	-2.61483	-1.21141	-2.52998
H	-1.33049	-2.43051	-2.58072
H	-3.03912	-2.95712	-2.48434
H	0.51273	-2.46878	-0.53200
H	2.09214	-3.28659	-0.25987
H	1.70706	-2.60025	-1.88547
C	4.54634	-2.03332	-0.21149
C	4.83226	1.20098	0.55231
H	0.93681	2.46396	0.28188
H	2.56429	2.97898	-0.35666
H	2.35890	2.61104	1.39774
H	-3.62436	-3.94135	2.03404
H	4.36920	-2.84070	0.53243

H	5.55298	-1.61819	-0.01288
H	4.57407	-2.50976	-1.21513
H	4.99107	2.04767	-0.14975
H	5.74638	0.57713	0.53494
H	4.74309	1.63457	1.57200
O	-0.89309	1.76072	0.07768
C	-2.13277	2.39018	-0.18429
C	-2.10435	3.85490	0.26940
H	-2.96947	1.86281	0.34448
C	-3.44133	4.57768	0.06374
H	-1.29421	4.38032	-0.28477
H	-1.81377	3.88307	1.34358
H	-3.39119	5.63376	0.40120
H	-3.74062	4.58490	-1.00662
H	-4.26086	4.08620	0.63106
H	-2.38942	2.35270	-1.27727

P^{HB}

C	-2.18358	-3.36073	0.89309
O	-1.52233	-2.13483	0.55478
H	-3.14004	-3.44798	0.33276
B	-2.21749	-1.09519	0.01382
O	-3.58402	-1.10282	-0.23056
O	-1.59371	0.08516	-0.34764
C	-3.87412	0.08689	-1.02247
C	-2.64885	1.03837	-0.65994
C	-3.88276	-0.34519	-2.49876
C	-5.25009	0.62128	-0.61787
C	-2.17241	1.93869	-1.80270
C	-2.89352	1.87373	0.60889
H	-4.61385	-1.16867	-2.62796
H	-4.17400	0.48619	-3.17220
H	-2.88797	-0.71970	-2.81466
H	-5.47534	1.57362	-1.14104
H	-6.03372	-0.11301	-0.89496
H	-5.31823	0.79217	0.47317
H	-2.97648	2.63274	-2.12412
H	-1.31089	2.54842	-1.46259
H	-1.84187	1.34840	-2.67821
H	-3.62803	2.68653	0.43700
H	-3.25887	1.24125	1.44329
H	-1.93485	2.33159	0.92541
C	-2.44017	-3.46597	2.39891
C	-1.16974	-3.40185	3.25478
H	-2.97983	-4.42309	2.58012
H	-3.14497	-2.65666	2.69434
H	-1.40695	-3.46299	4.33696
H	-0.47836	-4.23796	3.01408
H	-0.62029	-2.45506	3.07763
H	-1.51479	-4.18720	0.56272

TS-33

C	2.18600	0.23095	-0.83443
O	2.04855	1.26557	-1.51974
C	3.13516	0.20395	0.37949
C	3.17539	-1.09936	1.17658
H	2.90451	1.07356	1.03132
H	4.14252	0.41360	-0.05166
H	3.96442	-1.07882	1.95696
H	2.20503	-1.29015	1.68116
H	3.39274	-1.96991	0.51857
H	2.11077	-0.78858	-1.33469
C	0.28547	-0.08030	0.10230

N	-0.51836	1.01443	0.22871
N	-0.59418	-1.12034	-0.02295
C	0.01746	2.36967	0.34725
C	-1.88088	0.67852	0.19730
C	-1.93150	-0.69269	0.03389
C	-0.18723	-2.50786	-0.18893
H	0.19939	2.63518	1.40947
H	-0.69886	3.09488	-0.08374
H	0.96510	2.40231	-0.23577
C	-2.98055	1.68346	0.32196
C	-3.09372	-1.62671	-0.07347
H	0.91443	-2.53951	-0.26396
H	-0.62251	-2.94125	-1.11314
H	-0.50533	-3.12650	0.67650
H	-2.90097	2.27448	1.25987
H	-3.96601	1.17844	0.32956
H	-2.98677	2.40653	-0.52284
H	-3.11977	-2.15441	-1.05216
H	-4.04698	-1.07210	0.02589
H	-3.08039	-2.40718	0.71856
**INT-35**			
C	-0.42001	0.04428	-2.94286
O	0.80047	-0.31173	-2.58091
C	-1.59680	-0.79875	-2.32978
C	-3.00022	-0.50134	-2.86841
H	-1.56918	-0.68607	-1.22261
H	-1.31060	-1.85409	-2.53082
H	-3.76779	-1.16741	-2.42075
H	-3.31699	0.54362	-2.65228
H	-3.05133	-0.64241	-3.97080
H	-0.61653	0.01661	-4.07758
C	-0.62341	1.56914	-2.60011
N	-0.16168	2.14606	-1.45556
N	-0.96869	2.60236	-3.42650
C	0.42911	1.36404	-0.36849
C	-0.21002	3.54028	-1.55167
C	-0.72266	3.83599	-2.80027
C	-1.57106	2.45753	-4.74492
H	-0.33209	1.10860	0.39671
H	1.24409	1.94511	0.10331
H	0.81813	0.44092	-0.89328
C	0.24485	4.43914	-0.44838
C	-1.00103	5.14788	-3.45936
H	-1.39779	1.42123	-5.09521
H	-1.10355	3.16518	-5.45730
H	-2.66448	2.64500	-4.71179
H	-0.26114	4.19962	0.51107
H	0.02233	5.49529	-0.69323
H	1.33965	4.36225	-0.27096
H	-0.36938	5.30951	-4.36038
H	-0.79202	5.97971	-2.75974
H	-2.06168	5.24058	-3.77916
**INT-21**			
B	-1.83726	-1.52069	0.00475
O	-3.12109	-1.73344	-0.70119
O	-1.05077	-0.60767	-0.85582
C	-2.99802	-1.21073	-2.02971
C	-1.93626	-0.04735	-1.83181
C	-2.47380	-2.31735	-2.97239
C	-4.38274	-0.74651	-2.50153
C	-1.12911	0.30880	-3.08773

C	-2.57886	1.22942	-1.24723
H	-3.14237	-3.20040	-2.89965
H	-2.45053	-1.99543	-4.03470
H	-1.45029	-2.63380	-2.68459
H	-4.32594	-0.23311	-3.48485
H	-5.05670	-1.62157	-2.61423
H	-4.84558	-0.06001	-1.76715
H	-1.79114	0.65707	-3.90867
H	-0.41681	1.12849	-2.85747
H	-0.54054	-0.55595	-3.45141
H	-3.20295	1.77019	-1.98929
H	-3.20515	0.98524	-0.36600
H	-1.77012	1.91058	-0.91173
H	-2.03568	-1.11269	1.17858
C	-0.99210	-2.93976	0.24148
N	0.35507	-3.09255	0.38286
N	-1.52630	-4.14959	0.57238
C	0.67433	-4.38936	0.80915
C	1.33741	-2.03268	0.15684
C	-0.52573	-5.06326	0.92977
C	-2.95975	-4.43866	0.59338
C	2.07967	-4.84072	1.04118
H	2.13085	-2.39136	-0.52916
H	0.80590	-1.17550	-0.30244
H	1.80306	-1.72754	1.11687
C	-0.82313	-6.47129	1.33164
H	-3.16647	-5.37082	0.03059
H	-3.31474	-4.56114	1.63773
H	-3.47702	-3.58613	0.10852
H	2.59458	-4.22211	1.80749
H	2.09534	-5.88961	1.39431
H	2.69295	-4.79207	0.11509
H	-1.49581	-6.51900	2.21490
H	-1.31372	-7.04444	0.51492
H	0.11104	-7.00327	1.59503
	**TS-23**		
Ge	-2.58940	0.14184	-1.26761
N	-2.83434	2.49643	0.84190
N	-4.74424	1.87235	0.02434
C	-3.65997	-1.51620	-0.72893
C	-3.42135	1.52445	0.08275
C	-3.90090	-1.96433	0.60674
C	-4.13870	-2.29554	-1.82937
C	-4.59894	-3.17646	0.81131
H	-4.77888	-3.52489	1.84176
C	-1.41460	2.58403	1.17850
H	-0.89588	1.68063	0.81609
H	-1.28880	2.64756	2.27739
H	-0.96735	3.48696	0.71557
C	-4.83798	-3.49664	-1.57976
H	-5.20399	-4.09391	-2.43086
C	-5.06523	-3.93828	-0.26917
H	-5.60635	-4.88059	-0.08866
C	-3.77595	3.44221	1.26988
C	-4.99176	3.04823	0.74381
C	-3.41761	4.60468	2.13664
H	-3.01194	4.28179	3.11942
H	-4.31145	5.22490	2.33627
H	-2.65666	5.25978	1.66177
C	-5.77298	1.16013	-0.73247
H	-5.42653	0.12905	-0.93500
H	-5.98821	1.67929	-1.68910

H	-6.70475	1.10144	-0.13873
C	-6.34934	3.66125	0.85764
H	-6.77825	3.90902	-0.13637
H	-6.29818	4.60003	1.44043
H	-7.06912	2.99050	1.37423
C	-3.93234	-1.87565	-3.27599
H	-4.34535	-2.63411	-3.96993
H	-4.43041	-0.91100	-3.51339
H	-2.85912	-1.74294	-3.53177
C	-3.46788	-1.18789	1.83525
H	-2.45262	-0.75933	1.73515
H	-4.15801	-0.33998	2.03848
H	-3.47925	-1.83350	2.73606
O	-0.92000	-0.26918	-0.04137
C	-0.02754	-1.14163	-0.20140
B	3.45771	-0.69085	-0.01300
C	-0.12191	-2.27114	-1.16994
H	0.88108	-1.03550	0.45196
H	2.65042	-0.93544	-0.95691
O	4.27080	-1.83468	0.42595
O	2.68400	-0.26778	1.18316
C	-0.53264	-3.59496	-0.47232
H	0.88631	-2.39355	-1.62001
H	-0.84692	-2.01681	-1.97298
C	3.97468	-2.14990	1.79385
C	3.36357	-0.79185	2.34769
H	-0.52847	-4.41299	-1.21918
H	0.18064	-3.86762	0.33083
H	-1.55031	-3.52309	-0.04074
C	2.95766	-3.30978	1.82165
C	5.26676	-2.59844	2.49258
C	2.34021	-0.95843	3.47734
C	4.45535	0.20351	2.79151
H	3.35570	-4.14581	1.21196
H	2.76872	-3.68728	2.84771
H	1.98925	-3.00016	1.37921
H	5.10295	-2.76988	3.57710
H	5.61978	-3.55278	2.05080
H	6.07589	-1.85317	2.37134
H	2.79841	-1.44433	4.36353
H	1.96236	0.03501	3.79716
H	1.47138	-1.56673	3.15977
H	4.95504	-0.11857	3.72757
H	5.23584	0.32745	2.01398
H	3.99356	1.19458	2.98146
C	4.44697	0.49230	-0.65335
N	5.54620	0.28918	-1.43376
N	4.23224	1.83879	-0.68206
C	6.13671	-1.01710	-1.73365
C	6.02489	1.49816	-1.95603
C	5.18843	2.48745	-1.47560
C	3.14290	2.52242	0.00972
H	7.23079	-0.98098	-1.56524
H	5.94526	-1.29103	-2.79146
H	5.68368	-1.76246	-1.05334
C	7.22618	1.57776	-2.84053
C	5.20704	3.96825	-1.67246
H	2.62575	1.77328	0.64000
H	2.43922	2.96742	-0.72417
H	3.54685	3.33087	0.65160
H	8.14622	1.22544	-2.32630
H	7.40376	2.62281	-3.15740

H	7.10717	0.96514	-3.75943
H	4.26285	4.34196	-2.12358
H	6.03371	4.25602	-2.34914
H	5.35653	4.51521	-0.71640
	**INT-22**		
Ge	-1.64357	-0.19785	-0.06756
N	-2.91351	2.68940	-0.19335
N	-4.05512	1.22490	-1.31242
C	-2.97859	-1.52407	0.77714
C	-3.00515	1.34875	-0.44186
C	-3.81541	-1.20557	1.89009
C	-2.99678	-2.85530	0.26035
C	-4.63969	-2.20775	2.45180
H	-5.27866	-1.95453	3.31447
C	-1.94863	3.31356	0.71021
H	-1.34285	2.50485	1.16956
H	-2.48059	3.87463	1.50473
H	-1.29988	4.01813	0.15017
C	-3.83779	-3.82930	0.84301
H	-3.84260	-4.85414	0.43507
C	-4.65667	-3.51015	1.93482
H	-5.30506	-4.27837	2.38575
C	-3.89453	3.40451	-0.89441
C	-4.61974	2.47229	-1.61107
C	-4.04182	4.88733	-0.78818
H	-4.27263	5.20898	0.25021
H	-4.86676	5.24087	-1.43520
H	-3.11926	5.42170	-1.10066
C	-4.50324	-0.03628	-1.89441
H	-4.21075	-0.86531	-1.22187
H	-4.05647	-0.19232	-2.89878
H	-5.60550	-0.03750	-1.98836
C	-5.78885	2.64011	-2.52653
H	-5.58702	2.23712	-3.54190
H	-6.03683	3.71258	-2.63835
H	-6.69852	2.13037	-2.14137
C	-2.12904	-3.27930	-0.91379
H	-2.26557	-4.35688	-1.13747
H	-2.36853	-2.71409	-1.84100
H	-1.05067	-3.10472	-0.71041
C	-3.86785	0.18351	2.49375
H	-2.85169	0.61524	2.58860
H	-4.46471	0.87396	1.85743
H	-4.34844	0.16675	3.49272
O	-0.89660	0.58819	1.51099
C	0.43599	0.26694	1.84424
B	4.00976	0.29017	-0.32302
C	0.60514	-1.09692	2.53541
H	0.81023	1.06709	2.53152
H	1.10612	0.29391	0.93994
O	5.01831	0.18524	0.59836
O	3.63912	1.57169	-0.62751
C	-0.17595	-1.23850	3.84547
H	1.69486	-1.25199	2.71846
H	0.29437	-1.89669	1.82359
C	5.24425	1.54453	1.14680
C	4.63462	2.46882	0.00542
H	-0.00534	-2.22906	4.31518
H	0.12514	-0.46100	4.58030
H	-1.26468	-1.13279	3.66881
C	4.47066	1.61071	2.47005
C	6.74323	1.72000	1.38540

C	3.90506	3.71333	0.50883
C	5.64291	2.83601	-1.09184
H	4.82388	0.80214	3.14045
H	4.63828	2.57724	2.98513
H	3.38048	1.47932	2.31776
H	6.96335	2.75285	1.72402
H	7.08151	1.02664	2.18109
H	7.33780	1.51468	0.47565
H	4.60405	4.37715	1.05715
H	3.50125	4.28552	-0.35021
H	3.06307	3.45800	1.17880
H	6.39220	3.56414	-0.72305
H	6.18171	1.94527	-1.47315
H	5.10451	3.30117	-1.94150
C	3.35895	-0.97180	-1.00665
N	3.72035	-2.28052	-0.82747
N	2.34937	-0.97921	-1.92739
C	4.77414	-2.77625	0.06262
C	2.94160	-3.10809	-1.62543
C	2.06493	-2.28029	-2.32021
C	1.56506	0.17673	-2.36698
H	5.55345	-3.29514	-0.52992
H	4.34064	-3.48917	0.79113
H	5.22322	-1.92468	0.60110
C	3.10178	-4.59209	-1.65035
C	0.99579	-2.60614	-3.30900
H	2.06895	1.10131	-2.03948
H	0.54799	0.11489	-1.90074
H	1.47734	0.16303	-3.47000
H	4.12162	-4.89278	-1.97135
H	2.38224	-5.04475	-2.35726
H	2.91941	-5.04322	-0.65199
H	0.01073	-2.22142	-2.97266
H	0.90818	-3.70084	-3.43692
H	1.21124	-2.16672	-4.30647
**TS-24**			
Ge	-1.08726	-0.47153	0.37662
C	-2.35449	-1.58987	-0.81176
C	-2.19522	1.31519	0.32603
C	-3.21001	-1.12785	-1.85979
C	-2.40921	-2.97534	-0.45074
N	-3.37816	1.40298	1.00853
N	-1.88696	2.60142	-0.00814
C	-4.08476	-2.03657	-2.50332
C	-3.26670	0.31058	-2.34348
C	-3.29496	-3.85017	-1.11507
C	-1.53120	-3.56846	0.63998
C	-4.07941	0.24501	1.55573
C	-3.82859	2.72989	1.07960
C	-2.87630	3.49396	0.43118
C	-0.71256	2.98839	-0.78159
H	-4.73455	-1.67016	-3.31567
C	-4.13520	-3.38583	-2.13599
H	-3.98696	0.91676	-1.75095
H	-2.28826	0.82183	-2.28209
H	-3.60818	0.35536	-3.39772
H	-3.32002	-4.91424	-0.82702
H	-0.45402	-3.35891	0.46518
H	-1.77000	-3.15683	1.64352
H	-1.65575	-4.66906	0.68822
H	-4.66359	-0.27912	0.77280
H	-4.75570	0.56654	2.36862

H	-3.33491	-0.46638	1.97048
C	-5.10381	3.12978	1.74864
C	-2.80360	4.96557	0.18301
H	-0.04171	2.11002	-0.85206
H	-0.18248	3.82171	-0.27963
H	-1.00522	3.31636	-1.80075
H	-4.82104	-4.07741	-2.65058
H	-5.98501	2.61620	1.30868
H	-5.26716	4.21849	1.63847
H	-5.09646	2.90785	2.83783
H	-1.92096	5.43027	0.67332
H	-3.70469	5.46940	0.58081
H	-2.74168	5.20165	-0.90080
O	0.41537	-0.03907	-0.82142
C	0.41366	-0.38877	-2.20928
C	0.74360	-1.85665	-2.50271
H	1.16906	0.25498	-2.72010
C	0.71878	-2.17212	-4.00397
H	0.00748	-2.49942	-1.97298
H	1.74570	-2.08325	-2.08245
H	0.97071	-3.23449	-4.19818
H	-0.28461	-1.98594	-4.44186
H	1.44898	-1.55123	-4.56654
H	-0.56951	-0.14035	-2.67077
B	2.57542	0.31139	0.04871
O	2.71945	1.68626	0.13211
O	3.33530	-0.25100	-0.96299
C	3.57995	2.13582	-0.97254
C	4.26033	0.77594	-1.46126
C	2.68988	2.79854	-2.03215
C	4.55976	3.16899	-0.40624
C	4.38348	0.62832	-2.97900
C	5.61784	0.49704	-0.79546
H	2.14671	3.64745	-1.57132
H	3.29479	3.20157	-2.86842
H	1.94534	2.09283	-2.44764
H	5.28467	3.49017	-1.18156
H	4.00429	4.06883	-0.07328
H	5.12399	2.77746	0.46061
H	5.04529	1.41395	-3.39698
H	4.83222	-0.35510	-3.22390
H	3.40245	0.68973	-3.48583
H	6.39942	1.19831	-1.14911
H	5.55869	0.57117	0.30906
H	5.94396	-0.53073	-1.05239
C	2.19566	-0.51850	1.35746
N	2.38075	-1.85434	1.56274
N	1.71963	-0.02116	2.53406
C	2.90136	-2.81375	0.58748
C	1.98404	-2.20897	2.85055
C	1.55808	-1.04222	3.46632
C	1.30410	1.35949	2.77225
H	2.08732	-3.47838	0.23545
H	3.69317	-3.42739	1.05848
H	3.32080	-2.25736	-0.26797
C	2.06163	-3.60890	3.36393
C	1.01776	-0.80848	4.83822
H	1.73425	2.00293	1.98569
H	1.66649	1.69059	3.76405
H	0.19591	1.41669	2.74088
H	1.47294	-4.31113	2.73714
H	1.66102	-3.66343	4.39326

H	3.10707	-3.98406	3.39403
H	1.66188	-0.12309	5.42959
H	0.95305	-1.76277	5.39336
H	-0.00122	-0.36893	4.80539
	**INT-23**		
Ge	-1.00565	-0.56065	0.57652
C	-2.25258	-1.80881	-0.47845
C	-2.19701	1.17224	0.53834
C	-3.31176	-1.47438	-1.38075
C	-2.05284	-3.18808	-0.14066
N	-3.27087	1.20055	1.38657
N	-2.09087	2.44364	0.05683
C	-4.12590	-2.50305	-1.91207
C	-3.65573	-0.06240	-1.82046
C	-2.88769	-4.18253	-0.69242
C	-0.95754	-3.64682	0.80868
C	-3.75298	0.03660	2.12595
C	-3.86299	2.47013	1.41427
C	-3.11192	3.26170	0.56506
C	-1.11150	2.88388	-0.93456
H	-4.93382	-2.23643	-2.61341
C	-3.92356	-3.84563	-1.57321
H	-4.30154	0.45788	-1.08010
H	-2.76255	0.57310	-1.96888
H	-4.21863	-0.07838	-2.77522
H	-2.71604	-5.23856	-0.42675
H	0.03872	-3.25319	0.51879
H	-1.13688	-3.31293	1.85308
H	-0.88921	-4.75300	0.82134
H	-4.41323	-0.59532	1.49818
H	-4.30417	0.36594	3.02546
H	-2.88425	-0.57469	2.45061
C	-5.06316	2.79731	2.24258
C	-3.26373	4.70259	0.19843
H	-0.15547	2.34276	-0.79502
H	-0.91860	3.96411	-0.80147
H	-1.49472	2.71949	-1.96364
H	-4.56768	-4.62992	-2.00123
H	-5.92947	2.14677	1.99811
H	-5.37539	3.84352	2.06460
H	-4.86344	2.69589	3.33128
H	-2.39295	5.31226	0.52281
H	-4.16096	5.12792	0.68625
H	-3.38244	4.84454	-0.89654
O	0.41478	-0.16665	-0.88603
C	0.12382	-0.27936	-2.31123
C	0.42713	-1.66095	-2.88700
H	0.71457	0.49749	-2.83820
C	0.04487	-1.75224	-4.37078
H	-0.13237	-2.42387	-2.30450
H	1.50955	-1.86609	-2.75888
H	0.27628	-2.75652	-4.77908
H	-1.04040	-1.57523	-4.52651
H	0.59873	-1.01042	-4.98476
H	-0.95018	-0.03878	-2.44212
B	1.85145	0.32903	-0.46495
O	1.91659	1.78123	-0.35834
O	2.79770	-0.05186	-1.48676
C	2.94123	2.30928	-1.24214
C	3.76857	1.00649	-1.64958
C	2.25887	2.99187	-2.44190
C	3.75503	3.36206	-0.47517

C	4.26116	0.98925	-3.10177
C	4.96023	0.73369	-0.70893
H	1.58836	3.79541	-2.07486
H	2.99760	3.46181	-3.12165
H	1.65178	2.28046	-3.03608
H	4.59561	3.74472	-1.09001
H	3.10782	4.22638	-0.22065
H	4.16973	2.95975	0.46835
H	4.95343	1.83302	-3.30022
H	4.81314	0.04783	-3.29842
H	3.42342	1.04436	-3.82215
H	5.77306	1.47502	-0.84439
H	4.66072	0.74656	0.35842
H	5.37795	-0.26809	-0.93642
C	2.16506	-0.40370	1.00464
N	2.69376	-1.65240	1.17484
N	2.09996	0.13190	2.25898
C	2.99250	-2.60370	0.10339
C	2.92791	-1.91705	2.52736
C	2.54626	-0.78099	3.21807
C	1.52314	1.43175	2.59223
H	2.22707	-3.40512	0.06636
H	3.98250	-3.06436	0.28421
H	3.01971	-2.04947	-0.85164
C	3.50398	-3.20647	3.01460
C	2.57172	-0.47596	4.68041
H	1.50640	2.04476	1.67297
H	2.14377	1.92996	3.36141
H	0.49215	1.29783	2.98214
H	2.89764	-4.07971	2.69408
H	3.54413	-3.21606	4.12001
H	4.53969	-3.36776	2.64508
H	3.25711	0.36547	4.91975
H	2.92149	-1.35715	5.25032
H	1.56592	-0.20611	5.06615
**TS-25**			
Ge	-1.27961	0.69376	-0.95477
C	-1.97751	1.91728	0.52850
C	-2.45316	-1.04080	-0.67251
C	-2.27723	1.56252	1.87988
C	-2.15032	3.27732	0.11153
N	-3.81996	-1.06933	-0.53734
N	-2.12712	-2.28362	-1.14510
C	-2.73973	2.55536	2.77420
C	-2.14116	0.14868	2.40365
C	-2.61613	4.23803	1.03472
C	-1.86363	3.73689	-1.30958
C	-4.68168	0.04058	-0.13443
C	-4.33906	-2.31712	-0.90164
C	-3.25961	-3.09098	-1.28557
C	-0.79345	-2.70084	-1.56788
H	-2.96577	2.27545	3.81654
C	-2.91031	3.88274	2.35968
H	-1.94226	0.14852	3.49477
H	-3.08205	-0.42723	2.25896
H	-1.33069	-0.40753	1.89593
H	-2.75190	5.28110	0.70440
H	-0.82383	3.50683	-1.62554
H	-2.53371	3.25300	-2.05218
H	-2.00494	4.83200	-1.40546
H	-4.05852	0.90622	0.15040
H	-5.30738	-0.25338	0.73117

H	-5.34778	0.32983	-0.97237
C	-5.79690	-2.63717	-0.84133
C	-3.19792	-4.50436	-1.76520
H	-0.03251	-2.06266	-1.09024
H	-0.70418	-2.64462	-2.67250
H	-0.60787	-3.74309	-1.24733
H	-3.27135	4.64363	3.06979
H	-6.19995	-2.55572	0.19076
H	-5.97737	-3.67252	-1.18635
H	-6.39846	-1.96170	-1.48612
H	-2.74890	-4.58121	-2.77802
H	-4.21476	-4.93654	-1.81820
H	-2.59904	-5.14826	-1.08581
O	0.54220	-1.28474	0.99750
C	0.52626	-2.47002	1.81049
C	0.96909	-2.23686	3.25916
H	1.15409	-3.26524	1.34258
C	0.89589	-3.51433	4.10547
H	0.32466	-1.44732	3.70453
H	2.00158	-1.82892	3.25422
H	1.21244	-3.32253	5.15057
H	-0.13575	-3.92575	4.14277
H	1.55575	-4.31233	3.70251
H	-0.52199	-2.84776	1.79774
B	1.77563	-0.94729	0.37312
O	2.22868	-1.65380	-0.77168
O	2.86850	-0.57248	1.17693
C	3.65453	-1.92941	-0.62570
C	4.11291	-0.88819	0.50077
C	3.78416	-3.39851	-0.18629
C	4.34794	-1.74865	-1.98076
C	5.08480	-1.46567	1.53881
C	4.70560	0.41419	-0.06404
H	3.28188	-4.04471	-0.93426
H	4.84339	-3.71683	-0.11507
H	3.30375	-3.57721	0.79598
H	5.43857	-1.93039	-1.89267
H	3.94543	-2.48055	-2.71046
H	4.20251	-0.73404	-2.39658
H	6.03465	-1.78394	1.06243
H	5.32826	-0.68951	2.29212
H	4.65395	-2.33150	2.07519
H	5.69828	0.24531	-0.52740
H	4.04325	0.87854	-0.81878
H	4.83836	1.13730	0.76586
C	1.07437	0.92152	-0.47995
N	1.33703	1.97759	0.37162
N	1.61917	1.33832	-1.68606
C	1.08072	1.95355	1.80810
C	1.99999	3.01214	-0.27134
C	2.17121	2.60840	-1.59404
C	1.47830	0.56549	-2.91043
H	0.61370	0.98689	2.05864
H	0.40518	2.78267	2.09571
H	2.03911	2.03474	2.35781
C	2.42850	4.26057	0.42718
C	2.81740	3.31973	-2.73870
H	1.67541	-0.49758	-2.67584
H	2.19536	0.92247	-3.67134
H	0.43683	0.66752	-3.29482
H	1.56780	4.78407	0.89411
H	2.90616	4.96241	-0.28192

H	3.16228	4.04903	1.23436
H	3.69462	2.76317	-3.13273
H	3.17737	4.31523	-2.41765
H	2.11363	3.47584	-3.58358

Me_3SiCN

Si	-0.31248	-0.00008	-0.00015
C	-0.86104	-1.70862	-0.58962
H	-1.96989	-1.77099	-0.61104
H	-0.48564	-1.91966	-1.61217
H	-0.48534	-2.50509	0.08530
C	-0.85906	0.34352	1.77505
H	-1.96789	0.35618	1.84095
H	-0.48306	-0.43661	2.46863
H	-0.48326	1.32625	2.12724
C	-0.86068	1.36558	-1.18428
H	-1.96952	1.41619	-1.22693
H	-0.48420	2.35612	-0.85547
H	-0.48592	1.17960	-2.21191
C	1.57308	-0.00039	-0.00078
N	2.74796	-0.00020	-0.00069

INT-9

Si	-0.61738	4.05893	1.41317
C	-2.05186	3.84946	2.61496
H	-2.32724	4.84152	3.03248
H	-2.94857	3.43250	2.11285
H	-1.78244	3.18786	3.46338
C	0.96784	4.63912	2.24734
H	0.81111	5.65833	2.66080
H	1.25612	3.97357	3.08644
H	1.81596	4.69183	1.53451
C	-1.07423	5.08024	-0.10227
H	-1.31662	6.11712	0.21492
H	-0.23960	5.13743	-0.83048
H	-1.96462	4.66854	-0.61996
C	-0.24007	2.29800	0.76852
N	-0.02503	1.19706	0.41680
Ge	0.28090	-0.54528	-0.70063
C	0.13552	-1.98167	0.75456
C	2.57899	-0.12978	-0.42869
C	0.41106	-1.85356	2.15001
C	-0.27252	-3.25165	0.24133
N	3.63499	-0.95961	-0.15172
N	3.14988	0.88135	-1.16380
C	0.24734	-2.97659	2.99507
C	0.88207	-0.56003	2.78261
C	-0.42322	-4.34901	1.11689
C	-0.54222	-3.46392	-1.23690
C	3.56080	-2.20598	0.60527
C	4.84001	-0.47668	-0.68300
C	4.53124	0.70466	-1.32764
C	2.40891	2.00638	-1.71650
H	0.46164	-2.87173	4.07174
C	-0.17159	-4.21469	2.48942
H	1.63624	-0.04533	2.15332
H	0.04238	0.15424	2.92456
H	1.32404	-0.74535	3.78210
H	-0.73741	-5.32396	0.70881
H	-1.31718	-2.76162	-1.61423
H	0.36769	-3.30636	-1.85579
H	-0.89836	-4.49416	-1.43640
H	4.17312	-2.13889	1.52741

H	3.93433	-3.05157	-0.00652
H	2.50987	-2.39735	0.88116
C	6.14374	-1.18890	-0.52125
C	5.40357	1.66248	-2.07329
H	1.36623	1.68530	-1.90953
H	2.86271	2.32583	-2.67364
H	2.40142	2.87173	-1.02096
H	-0.29063	-5.07783	3.16339
H	6.41555	-1.32443	0.54742
H	6.95984	-0.61602	-1.00103
H	6.12852	-2.19759	-0.98723
H	5.13542	1.72714	-3.15034
H	6.46022	1.33832	-2.02011
H	5.35136	2.69032	-1.65464
C	-2.26973	-0.40477	-0.73101
N	-3.30318	-0.68344	0.12326
N	-2.90419	-0.11364	-1.91169
C	-3.14042	-1.05309	1.52458
C	-4.55655	-0.56982	-0.49803
C	-4.30073	-0.20348	-1.80509
C	-2.22039	0.24825	-3.14851
H	-3.63191	-2.02476	1.73158
H	-3.58432	-0.28410	2.19013
H	-2.06246	-1.15543	1.73781
C	-5.84688	-0.82943	0.21033
C	-5.22700	0.05658	-2.94897
H	-1.12994	0.17038	-2.97591
H	-2.47260	1.28512	-3.45325
H	-2.50576	-0.43894	-3.97069
H	-5.91748	-1.87595	0.57779
H	-6.70343	-0.66038	-0.46984
H	-5.98396	-0.16364	1.08941
H	-5.11099	1.08343	-3.35781
H	-6.27993	-0.05389	-2.62666
H	-5.06307	-0.65192	-3.78954

INT-10

Si	4.31154	-1.30530	0.27547
C	5.22089	0.33987	0.20956
H	6.30447	0.15933	0.37661
H	5.10769	0.83306	-0.77731
H	4.86806	1.03830	0.99540
C	4.35084	-2.12349	1.96952
H	5.39738	-2.41131	2.20726
H	4.00058	-1.43691	2.76699
H	3.73390	-3.04473	2.00001
C	4.76134	-2.47265	-1.13030
H	5.82751	-2.76815	-1.02684
H	4.15284	-3.39977	-1.11143
H	4.63990	-1.98952	-2.12124
C	2.46955	-0.83666	-0.02376
N	1.35647	-0.51021	-0.22004
Ge	-0.26059	0.35024	-1.21420
C	-0.23551	2.07504	-0.13692
C	-1.69584	-0.80978	-0.23017
C	-0.03518	2.20630	1.27150
C	-0.36270	3.25008	-0.94467
N	-2.89686	-0.45286	0.31776
N	-1.76650	-2.16834	-0.38394
C	0.03288	3.49927	1.83898
C	0.11643	1.02464	2.20724
C	-0.28963	4.52064	-0.33564
C	-0.58840	3.18503	-2.44625

C	-3.33167	0.90850	0.63153
C	-3.70674	-1.57465	0.52885
C	-2.98317	-2.66985	0.08973
C	-0.71372	-2.98952	-0.97476
H	0.18251	3.60079	2.92667
C	-0.08923	4.64767	1.04625
H	-0.43322	0.12890	1.85579
H	1.18210	0.72481	2.30819
H	-0.24091	1.27655	3.22618
H	-0.39383	5.42314	-0.95961
H	0.23154	2.65053	-2.97214
H	-1.53247	2.66174	-2.71198
H	-0.65088	4.20248	-2.87993
H	-3.61307	0.98169	1.70030
H	-4.20783	1.18214	0.01027
H	-2.50255	1.61003	0.42976
C	-5.07500	-1.48303	1.12087
C	-3.33171	-4.12260	0.07498
H	-0.13790	-2.37366	-1.69379
H	-1.16600	-3.83770	-1.52123
H	-0.02733	-3.38416	-0.19846
H	-0.03382	5.64648	1.50732
H	-5.05837	-1.04452	2.14123
H	-5.52950	-2.48834	1.19844
H	-5.75324	-0.85822	0.50172
H	-3.39432	-4.52784	-0.95791
H	-4.31759	-4.28365	0.54977
H	-2.59056	-4.73400	0.63188

INT-11

C	-3.69614	-0.38128	0.03476
O	-3.33877	0.78825	0.05982
C	-5.12477	-0.87413	-0.16786
C	-5.48869	-1.70684	1.08967
H	-6.49047	-2.16586	0.95768
H	-4.76178	-2.52815	1.26292
H	-5.52141	-1.07475	2.00131
H	-2.93513	-1.20841	0.17231
C	-6.09598	0.30062	-0.35899
H	-5.82959	0.90348	-1.25096
H	-7.13170	-0.07210	-0.49539
H	-6.08794	0.97790	0.51898
C	-5.10752	-1.79984	-1.41207
H	-4.37418	-2.62597	-1.29855
H	-6.10920	-2.25615	-1.55373
H	-4.85666	-1.23699	-2.33514
Si	-1.61954	3.70965	0.35803
C	-2.45193	3.41098	2.01519
H	-3.06840	4.29773	2.27587
H	-3.11833	2.52823	1.95477
H	-1.71544	3.25767	2.83004
C	-0.35306	5.10960	0.41976
H	-0.88503	6.06351	0.62467
H	0.39199	4.96329	1.22864
H	0.18356	5.22874	-0.54384
C	-2.80762	3.85214	-1.08788
H	-3.43178	4.76170	-0.95586
H	-2.27707	3.93933	-2.05773
H	-3.47315	2.96658	-1.11182
C	-0.53610	2.15655	0.01155
N	0.17918	1.24825	-0.20414
Ge	1.01984	-0.36115	-1.18286
C	0.13605	-1.80122	-0.04266

C	2.88718	-0.11187	-0.26955
C	-0.10240	-1.75913	1.36529
C	-0.34454	-2.91359	-0.80529
N	3.73102	-1.01854	0.31011
N	3.66304	0.99097	-0.50749
C	-0.81377	-2.81808	1.97604
C	0.36972	-0.62948	2.25642
C	-1.04850	-3.95008	-0.15498
C	-0.10733	-3.03635	-2.30143
C	3.38695	-2.38116	0.71545
C	5.01758	-0.48689	0.45732
C	4.97261	0.79741	-0.05608
C	3.19199	2.20984	-1.15928
H	-0.99044	-2.78811	3.06400
C	-1.28669	-3.90348	1.22661
H	1.30911	-0.16688	1.89348
H	-0.38729	0.18218	2.30668
H	0.53196	-0.98402	3.29432
H	-1.40637	-4.80994	-0.74455
H	-0.52445	-2.17559	-2.86628
H	0.97380	-3.08810	-2.55480
H	-0.57637	-3.95675	-2.70177
H	3.60366	-2.52532	1.79216
H	3.97681	-3.11427	0.12972
H	2.30971	-2.55031	0.53958
C	6.14466	-1.25537	1.06583
C	6.03718	1.84058	-0.15965
H	2.35439	1.95386	-1.83813
H	4.00978	2.65067	-1.75889
H	2.84483	2.95574	-0.41610
H	-1.83265	-4.72211	1.72193
H	5.92523	-1.55860	2.11161
H	7.06421	-0.64106	1.08389
H	6.37321	-2.17925	0.49307
H	6.27904	2.08903	-1.21549
H	6.97027	1.48255	0.31423
H	5.74617	2.78374	0.34929

INT-12

C	-3.08316	-0.86762	-0.23263
O	-3.65227	0.39977	-0.04604
C	-3.61131	-1.57820	-1.53573
C	-5.13267	-1.76838	-1.36434
H	-5.55003	-2.27649	-2.25742
H	-5.36942	-2.39619	-0.47963
H	-5.64982	-0.79530	-1.25247
H	-3.28139	-1.56305	0.62278
C	-3.32092	-0.69396	-2.76446
H	-2.22968	-0.58160	-2.94193
H	-3.75654	-1.15076	-3.67624
H	-3.76314	0.31444	-2.63955
C	-2.91918	-2.95017	-1.66867
H	-3.10864	-3.59633	-0.78519
H	-3.30908	-3.48508	-2.55846
H	-1.82013	-2.85794	-1.80143
Si	-4.23740	1.05990	1.45208
C	-5.20610	2.57013	0.90502
H	-5.61021	3.11198	1.78607
H	-4.56672	3.27699	0.33689
H	-6.06258	2.28805	0.25871
C	-5.31728	-0.22276	2.31476
H	-5.73705	0.21516	3.24590
H	-6.17179	-0.53420	1.67917

H	-4.75776	-1.13375	2.61508
C	-2.75262	1.52024	2.52416
H	-3.09364	1.97580	3.47852
H	-2.13487	0.63585	2.78765
H	-2.09790	2.25962	2.01734
C	-1.60190	-0.70650	-0.28482
N	-0.44571	-0.52024	-0.35293
Ge	1.49666	-0.63703	-1.13765
C	2.24109	-1.84812	0.31492
C	2.00932	1.28106	-0.47790
C	1.96762	-1.78382	1.71550
C	3.05651	-2.90833	-0.19586
N	3.14050	1.74040	0.13798
N	1.39068	2.40128	-0.96503
C	2.50756	-2.77244	2.56931
C	1.11518	-0.70354	2.34820
C	3.57642	-3.87303	0.69267
C	3.40078	-3.03148	-1.67119
C	4.16560	0.91813	0.78109
C	3.22422	3.13510	0.06043
C	2.10439	3.55784	-0.63520
C	0.15353	2.38850	-1.74191
H	2.29930	-2.71990	3.65079
C	3.30181	-3.81088	2.06617
H	1.17632	0.26059	1.80525
H	0.04221	-0.99466	2.36100
H	1.41070	-0.53049	3.40268
H	4.20801	-4.68461	0.29623
H	2.49541	-3.12500	-2.30845
H	3.96870	-2.15407	-2.04996
H	4.02857	-3.92527	-1.85621
H	4.30026	1.23182	1.83481
H	5.13157	1.02609	0.24841
H	3.84831	-0.13984	0.75858
C	4.35612	3.91042	0.65106
C	1.65874	4.93301	-1.01313
H	0.10608	1.44764	-2.32598
H	0.14840	3.24080	-2.44569
H	-0.73829	2.45275	-1.08656
H	3.71374	-4.57210	2.74742
H	4.43749	3.75791	1.74826
H	4.21479	4.99321	0.47544
H	5.33169	3.62543	0.20289
H	1.63939	5.07919	-2.11461
H	2.35187	5.68704	-0.59554
H	0.64394	5.16318	-0.62537
**INT-13**			
Ge	-1.15082	0.71721	-0.72183
C	-1.93359	1.79618	0.84135
C	-2.77170	-1.06916	-0.60779
C	-2.11083	1.38462	2.19695
C	-2.38352	3.09513	0.44987
N	-4.11727	-1.07114	-0.34179
N	-2.61801	-2.12912	-1.46925
C	-2.69189	2.27937	3.12641
C	-1.71209	0.01160	2.69631
C	-2.96199	3.95880	1.40508
C	-2.27083	3.57869	-0.98423
C	-4.80944	-0.12208	0.52489
C	-4.78873	-2.11065	-1.00296
C	-3.82793	-2.79195	-1.72315
C	-1.34553	-2.51957	-2.05900

H	-2.82353	1.95421	4.17191
C	-3.11045	3.55974	2.74064
H	-1.99415	-0.78216	1.97451
H	-0.61290	-0.06756	2.84385
H	-2.18704	-0.21124	3.67249
H	-3.30539	4.95779	1.08895
H	-1.21602	3.57023	-1.33496
H	-2.85325	2.94505	-1.68786
H	-2.64768	4.61545	-1.08953
H	-5.25558	-0.64212	1.39707
H	-5.61907	0.39058	-0.03242
H	-4.08685	0.63113	0.88230
C	-6.26062	-2.33799	-0.87949
C	-3.94488	-3.98645	-2.61422
H	-0.69782	-1.62357	-2.13067
H	-1.50525	-2.92017	-3.07816
H	-0.83510	-3.29619	-1.45107
H	-3.56361	4.24153	3.47765
H	-6.56931	-2.51300	0.17336
H	-6.56608	-3.22497	-1.46647
H	-6.84935	-1.47351	-1.25508
H	-3.68204	-3.75392	-3.66925
H	-4.98524	-4.36362	-2.61393
H	-3.29031	-4.82155	-2.28443
C	0.89378	2.11190	-0.58614
N	1.62989	2.69862	0.40637
N	1.49278	2.55660	-1.73712
C	1.38549	2.51361	1.83235
C	2.66946	3.49298	-0.10009
C	2.58034	3.40470	-1.47615
C	1.04607	2.22237	-3.08625
H	1.35358	3.49314	2.34860
H	2.18100	1.89213	2.29297
H	0.40571	2.02150	1.95759
C	3.61631	4.25388	0.77106
C	3.39537	4.04289	-2.55428
H	0.17042	1.55050	-3.00478
H	1.84994	1.70808	-3.65139
H	0.75301	3.13694	-3.64095
H	3.09483	5.02367	1.38009
H	4.37272	4.77777	0.15611
H	4.16094	3.59065	1.47650
H	3.87060	3.29226	-3.22167
H	4.20557	4.65544	-2.11496
H	2.78537	4.71392	-3.19681
N	0.15365	-0.62651	0.29030
C	0.95240	-1.44986	0.53141
C	2.01757	-2.45620	0.82115
O	3.26667	-1.82489	0.73089
C	1.80333	-3.14158	2.22034
H	1.88409	-3.25606	0.04658
Si	4.32270	-1.84446	-0.64287
C	2.87904	-4.23817	2.36364
C	1.96417	-2.09500	3.34015
C	0.39808	-3.77590	2.25641
C	5.78003	-0.80320	-0.08520
C	4.81463	-3.62562	-1.02114
C	3.41508	-1.07376	-2.11021
H	2.76605	-4.75261	3.33964
H	2.78992	-5.00635	1.56648
H	3.89865	-3.80784	2.32035
H	1.18258	-1.30808	3.27930

H	1.87277	-2.57846	4.33404
H	2.95610	-1.60514	3.28272
H	0.26688	-4.53004	1.45066
H	0.24221	-4.29771	3.22261
H	-0.40773	-3.01817	2.15829
H	6.55072	-0.75139	-0.88292
H	5.46532	0.23347	0.15346
H	6.25756	-1.23438	0.81876
H	5.50570	-3.64981	-1.89105
H	5.34005	-4.09346	-0.16308
H	3.94522	-4.26553	-1.28157
H	4.09652	-0.99072	-2.98390
H	2.54542	-1.68435	-2.43320
H	3.04883	-0.05586	-1.86129

P^{CS}

N	-0.74649	2.82591	0.08205
C	-0.79352	1.67273	-0.12656
C	-0.79381	0.20743	-0.39065
O	0.28235	-0.42495	0.26708
C	-2.15341	-0.45633	0.02325
H	-0.70053	0.09804	-1.50152
Si	1.96370	-0.18484	-0.00455
C	-2.08093	-1.94810	-0.36426
C	-2.36191	-0.31228	1.54428
C	-3.29925	0.23137	-0.74727
C	2.74394	-1.79541	0.58251
C	2.26180	0.10494	-1.85023
C	2.57388	1.27562	1.02109
H	-3.02379	-2.46281	-0.08530
H	-1.93779	-2.07527	-1.45887
H	-1.24136	-2.45213	0.15309
H	-2.43269	0.75441	1.84412
H	-3.30127	-0.81530	1.85472
H	-1.52088	-0.77139	2.10099
H	-3.16056	0.14843	-1.84682
H	-4.26826	-0.24798	-0.49661
H	-3.38125	1.30850	-0.49213
H	3.85135	-1.74645	0.51224
H	2.48090	-1.99653	1.64202
H	2.39939	-2.65836	-0.02427
H	3.35199	0.17652	-2.05285
H	1.86205	-0.72753	-2.46703
H	1.80126	1.05168	-2.20316
H	3.66905	1.41410	0.89368
H	2.07105	2.21976	0.72690
H	2.37551	1.10852	2.10050

TS-17

O	1.61234	-1.15258	1.52073
C	1.66090	-0.06122	0.91957
C	2.71197	0.18238	-0.21239
C	2.49914	1.52062	-0.93308
C	4.06970	0.21693	0.53843
C	2.70984	-0.98363	-1.21328
H	3.33786	1.73341	-1.63030
H	1.55868	1.51397	-1.52152
H	2.45813	2.36610	-0.20998
H	4.23696	-0.73757	1.07716
H	4.90959	0.37918	-0.17116
H	4.09658	1.03767	1.28801
H	1.76957	-0.99033	-1.80393
H	3.56158	-0.90659	-1.92279

H	2.79128	-1.94958	-0.67426
H	1.43592	0.90132	1.48140
C	-0.27858	0.11631	-0.05794
N	-1.04636	-1.01119	-0.12830
N	-1.19846	1.12761	0.03360
C	-0.47817	-2.35645	-0.19688
C	-2.41994	-0.72354	-0.10036
C	-2.51911	0.64971	0.00626
C	-0.85695	2.53714	0.15670
H	-0.30494	-2.66379	-1.24908
H	-1.17084	-3.07804	0.27670
H	0.48011	-2.34250	0.36485
C	-3.48388	-1.77103	-0.17564
C	-3.71416	1.54439	0.08432
H	0.24113	2.62788	0.21433
H	-1.30277	2.97280	1.07479
H	-1.21744	3.11515	-0.72010
H	-3.38753	-2.39873	-1.08773
H	-4.48631	-1.30117	-0.19966
H	-3.46197	-2.45702	0.69932
H	-3.75302	2.11185	1.04004
H	-4.64684	0.95141	0.01648
H	-3.73518	2.29065	-0.73979
	**INT-14**		
O	-1.52817	-1.07935	-1.58318
C	-1.34528	-0.01207	-0.82738
C	-2.42025	0.18525	0.35725
C	-2.18545	1.46656	1.17318
C	-3.78233	0.25184	-0.35862
C	-2.39069	-1.04495	1.28011
H	-2.97730	1.59981	1.94254
H	-1.21307	1.44537	1.71429
H	-2.20874	2.37278	0.52896
H	-3.87103	-0.61835	-1.04011
H	-4.62638	0.24735	0.36452
H	-3.86367	1.17358	-0.97591
H	-1.44761	-1.09574	1.86855
H	-3.23131	-1.02657	2.00732
H	-2.47680	-1.96613	0.66853
H	-1.41311	0.99036	-1.39352
C	0.13212	0.03443	-0.28913
N	0.90274	-1.06416	-0.05001
N	0.98889	1.10330	-0.25144
C	0.39788	-2.43705	-0.12718
C	2.23618	-0.69694	0.15751
C	2.29584	0.67698	0.03056
C	0.61545	2.49480	-0.46759
H	0.12600	-2.81597	0.87811
H	1.17667	-3.08680	-0.57057
H	-0.51262	-2.34008	-0.78964
C	3.30859	-1.69563	0.44815
C	3.45104	1.61807	0.14626
H	-0.31597	2.51881	-1.06681
H	1.41959	3.01462	-1.02296
H	0.43354	3.02218	0.49091
H	3.04200	-2.34411	1.30959
H	4.25863	-1.18278	0.69172
H	3.50407	-2.36438	-0.41809
H	3.68083	2.12633	-0.81593
H	4.36197	1.06770	0.44998
H	3.27187	2.41051	0.90465

INT-15			
C	-2.05680	-0.89989	0.91775
O	-1.61058	-1.85799	-0.03252
H	-1.20011	-0.22435	1.16544
Si	0.15043	-3.54209	-1.39186
C	-0.13760	-3.14624	-3.22546
H	0.07768	-4.03595	-3.85494
H	-1.18785	-2.84415	-3.42008
H	0.52299	-2.32206	-3.56618
C	1.95924	-4.01752	-1.10307
H	2.26164	-4.85668	-1.76437
H	2.62120	-3.15234	-1.31190
H	2.12471	-4.33816	-0.05302
C	-1.00388	-4.94170	-0.84089
H	-0.76820	-5.87755	-1.39169
H	-0.89858	-5.15742	0.24293
H	-2.06873	-4.69928	-1.03688
C	-0.16291	-1.92055	-0.39074
N	0.69237	-1.03806	-0.16155
Ge	1.26873	0.46946	0.89433
C	0.65597	2.14906	-0.12747
C	3.28114	0.32697	0.24992
C	-0.06678	2.19591	-1.36030
C	0.82341	3.36517	0.61365
N	4.09843	1.03026	-0.59118
N	4.10250	-0.59827	0.83620
C	-0.63182	3.41961	-1.79517
C	-0.21966	0.99210	-2.26770
C	0.24865	4.56768	0.14681
C	1.64596	3.40982	1.88717
C	3.68702	2.11805	-1.47433
C	5.41341	0.54955	-0.54514
C	5.41407	-0.49386	0.36190
C	3.67296	-1.59888	1.81000
H	-1.17149	3.44753	-2.75714
C	-0.48872	4.59769	-1.04712
H	0.70944	0.39064	-2.31157
H	-1.00912	0.29539	-1.91339
H	-0.48988	1.30544	-3.29646
H	0.39454	5.49528	0.72525
H	1.31981	2.63965	2.61999
H	2.72031	3.20317	1.68698
H	1.58879	4.40378	2.37387
H	3.87160	1.84313	-2.53222
H	4.25442	3.04099	-1.24090
H	2.60961	2.30730	-1.32377
C	6.51818	1.13136	-1.36567
C	6.52069	-1.38802	0.81864
H	2.70828	-1.26994	2.24524
H	4.42656	-1.68873	2.61550
H	3.53143	-2.58492	1.32418
H	-0.92437	5.54332	-1.40819
H	6.30856	1.06477	-2.45463
H	7.46473	0.59089	-1.17633
H	6.69368	2.20237	-1.12787
H	6.73523	-1.26845	1.90251
H	7.45336	-1.15539	0.27103
H	6.28795	-2.45949	0.64189
C	-2.48272	-1.59907	2.27108
C	-3.64460	-2.58881	2.05269
C	-1.24691	-2.35828	2.80005
C	-2.90112	-0.53665	3.30968

H	-4.57648	-2.07526	1.73348
H	-3.38367	-3.35926	1.30029
H	-3.87662	-3.11506	3.00166
H	-0.38573	-1.67193	2.94456
H	-1.47980	-2.82989	3.77696
H	-0.93799	-3.15793	2.09877
H	-3.17486	-1.03280	4.26351
H	-2.07435	0.16838	3.53587
H	-3.78915	0.04681	2.98553
C	-3.12093	-0.02268	0.28196
N	-4.08617	-0.39545	-0.60492
N	-3.27621	1.31608	0.48450
C	-4.31134	-1.73531	-1.15726
C	-4.86191	0.71265	-0.96228
C	-4.34964	1.79597	-0.27196
C	-2.47051	2.18405	1.34526
H	-4.38920	-1.66346	-2.25908
H	-5.25082	-2.16208	-0.75455
H	-3.45018	-2.37058	-0.89343
C	-5.99973	0.62070	-1.92501
C	-4.75509	3.23263	-0.26510
H	-1.58865	1.63576	1.71559
H	-3.07445	2.52940	2.20757
H	-2.11413	3.05419	0.76113
H	-5.66334	0.31939	-2.94008
H	-6.49987	1.60235	-2.02011
H	-6.76403	-0.11401	-1.59506
H	-5.02500	3.58247	0.75363
H	-5.63572	3.38662	-0.91590
H	-3.93930	3.88739	-0.63878

INT-16

C	-3.39714	-0.97956	0.63544
O	-4.06933	-1.94221	-0.15789
H	-2.29106	-1.05196	0.49804
Si	-3.39265	-2.79745	-1.50634
C	-2.69787	-1.54097	-2.73880
H	-2.31522	-2.06655	-3.64004
H	-3.46740	-0.81705	-3.08025
H	-1.84486	-0.97431	-2.30922
C	-2.03944	-3.96228	-0.91696
H	-1.61431	-4.51454	-1.78271
H	-1.21369	-3.40024	-0.43040
H	-2.43037	-4.71107	-0.19753
C	-4.87480	-3.71801	-2.20985
H	-4.56371	-4.35192	-3.06702
H	-5.32912	-4.38509	-1.44801
H	-5.66169	-3.02538	-2.57433
C	0.29605	-1.37542	0.60140
N	1.34335	-0.82232	0.69950
Ge	2.83144	0.36597	1.31032
C	2.31326	1.92966	0.06609
C	4.30549	-0.61020	0.19884
C	1.90629	1.82725	-1.30107
C	2.26123	3.20661	0.71072
N	5.38636	-0.08216	-0.45261
N	4.59591	-1.93890	0.34618
C	1.44907	2.98264	-1.98000
C	1.94413	0.52891	-2.07974
C	1.79799	4.33618	-0.00093
C	2.70911	3.40774	2.14929
C	5.56487	1.32691	-0.79272
C	6.34077	-1.06953	-0.73050

C	5.83404	-2.25407	-0.22852
C	3.73238	-2.89459	1.03668
H	1.15374	2.89961	-3.03979
C	1.38927	4.22800	-1.33808
H	2.83789	-0.07777	-1.83156
H	1.07118	-0.11424	-1.84128
H	1.93928	0.71861	-3.17196
H	1.77227	5.31677	0.50288
H	2.14457	2.76843	2.86053
H	3.78094	3.15266	2.29225
H	2.57784	4.46306	2.46150
H	5.81786	1.43336	-1.86585
H	6.37954	1.77329	-0.18660
H	4.62036	1.86632	-0.59385
C	7.62204	-0.77713	-1.44110
C	6.40576	-3.63476	-0.24422
H	3.27008	-2.40088	1.91405
H	4.33755	-3.75040	1.38616
H	2.92322	-3.25844	0.37342
H	1.04527	5.11976	-1.88696
H	7.45103	-0.37892	-2.46429
H	8.22593	-1.69855	-1.54144
H	8.23977	-0.03278	-0.89474
H	6.60818	-4.01761	0.77933
H	7.36682	-3.64261	-0.79214
H	5.73092	-4.35988	-0.74661
C	-3.63913	-1.25065	2.17467
C	-5.13497	-1.19494	2.54091
C	-3.08571	-2.66041	2.47644
C	-2.86112	-0.21457	3.01342
H	-5.56073	-0.17689	2.41433
H	-5.72814	-1.91133	1.93844
H	-5.27005	-1.46828	3.60782
H	-2.01039	-2.73359	2.21243
H	-3.19103	-2.88478	3.55800
H	-3.63841	-3.43568	1.91089
H	-2.99188	-0.43366	4.09310
H	-1.77251	-0.24938	2.80149
H	-3.22602	0.82241	2.85081
C	-3.79506	0.41970	0.17357
N	-5.02355	0.83734	-0.24187
N	-2.96752	1.49842	0.08688
C	-6.24183	0.02902	-0.36550
C	-4.97839	2.19438	-0.58053
C	-3.67786	2.61426	-0.36822
C	-1.53836	1.54419	0.40838
H	-6.67420	0.17974	-1.37351
H	-6.98421	0.33767	0.39623
H	-5.97133	-1.03157	-0.23658
C	-6.17516	2.94013	-1.07217
C	-3.04119	3.95218	-0.55280
H	-1.12570	0.52170	0.48172
H	-1.37699	2.06761	1.37089
H	-0.99432	2.08420	-0.39000
H	-6.54797	2.53979	-2.03916
H	-5.92315	4.00514	-1.23054
H	-7.01597	2.89879	-0.34815
H	-2.60042	4.33281	0.39249
H	-3.79137	4.68912	-0.89487
H	-2.22663	3.92488	-1.30720
	**TS-18**		
C	-2.42151	-0.57871	0.88044

O	-2.24521	-1.86107	0.44947
H	-1.52071	0.07636	0.67584
Si	-1.03941	-3.09605	-1.06717
C	-2.17545	-2.39232	-2.41478
H	-2.15988	-3.05056	-3.30876
H	-3.21655	-2.27870	-2.06171
H	-1.81498	-1.39229	-2.73595
C	0.36520	-3.92937	-2.10325
H	-0.05391	-4.75867	-2.71498
H	0.85928	-3.22255	-2.80486
H	1.15601	-4.37463	-1.45973
C	-1.60650	-4.50406	0.05783
H	-1.64779	-5.44523	-0.52999
H	-0.86652	-4.65536	0.87136
H	-2.58629	-4.30864	0.52860
C	0.10271	-1.76925	-0.28752
N	0.83583	-0.90664	0.05007
Ge	1.95452	0.41287	1.10487
C	1.41924	2.06987	0.01978
C	3.74535	-0.16331	0.17182
C	1.22994	2.14233	-1.39534
C	1.17389	3.23312	0.81783
N	4.78386	0.58390	-0.31209
N	4.25181	-1.42824	0.30165
C	0.79986	3.35923	-1.97555
C	1.47885	0.97420	-2.32642
C	0.74327	4.42782	0.19906
C	1.38677	3.24499	2.32262
C	4.75814	2.02126	-0.57577
C	5.92468	-0.20433	-0.50804
C	5.58317	-1.48878	-0.12477
C	3.50746	-2.56821	0.83177
H	0.67013	3.41490	-3.06943
C	0.55448	4.49331	-1.18931
H	2.31872	0.33649	-1.98619
H	0.59104	0.30943	-2.38962
H	1.69748	1.32525	-3.35497
H	0.56803	5.32236	0.81923
H	0.78769	2.46643	2.84041
H	2.44696	3.05583	2.59713
H	1.10965	4.22720	2.75414
H	5.04751	2.22336	-1.62585
H	5.46376	2.54813	0.09773
H	3.73486	2.40131	-0.40719
C	7.20824	0.34876	-1.03545
C	6.38725	-2.74815	-0.12428
H	2.76218	-2.20370	1.56570
H	4.20292	-3.25657	1.34600
H	2.98101	-3.11583	0.02444
H	0.23109	5.43547	-1.66046
H	7.08758	0.78976	-2.04792
H	7.96969	-0.45004	-1.11026
H	7.62146	1.14101	-0.37541
H	6.54059	-3.14748	0.90144
H	7.38842	-2.56216	-0.55629
H	5.90738	-3.54754	-0.72753
C	-2.60095	-0.46450	2.46763
C	-3.85086	-1.22502	2.94931
C	-1.34976	-1.11237	3.09183
C	-2.69488	1.00887	2.90950
H	-4.79142	-0.75257	2.59363
H	-3.82751	-2.27958	2.61003

H	-3.89407	-1.23017	4.05869
H	-0.42362	-0.56687	2.80693
H	-1.41720	-1.10778	4.19988
H	-1.25031	-2.15916	2.74434
H	-2.75899	1.07694	4.01580
H	-1.80042	1.59046	2.59988
H	-3.59898	1.51460	2.50598
C	-3.56508	0.11553	0.11464
N	-4.73561	-0.45686	-0.27831
N	-3.58187	1.39044	-0.37190
C	-5.13245	-1.84569	-0.02884
C	-5.50798	0.45907	-1.00118
C	-4.77956	1.63182	-1.06008
C	-2.53465	2.39902	-0.21612
H	-5.53614	-2.28122	-0.96296
H	-5.90879	-1.89362	0.75965
H	-4.21308	-2.38175	0.28134
C	-6.84981	0.11391	-1.55872
C	-5.09583	2.94453	-1.69908
H	-1.58014	1.91405	0.05560
H	-2.80280	3.12754	0.57425
H	-2.39319	2.93637	-1.17204
H	-6.78664	-0.67469	-2.33894
H	-7.31293	1.00355	-2.02491
H	-7.54216	-0.25207	-0.77165
H	-5.05143	3.78263	-0.97184
H	-6.11736	2.92585	-2.12287
H	-4.39847	3.18576	-2.53008
	**TS-19**		
C	2.34507	-1.67911	-0.55025
O	2.60068	-2.64395	0.25413
Si	2.50864	-2.66804	2.05432
C	4.14331	-3.44783	2.53973
H	4.15601	-3.63859	3.63411
H	4.29529	-4.42044	2.02802
H	5.00179	-2.78663	2.30447
C	2.30385	-0.90139	2.64051
H	2.37797	-0.86215	3.74824
H	3.10184	-0.25633	2.21502
H	1.31617	-0.49092	2.34701
C	1.03714	-3.78103	2.37730
H	1.19607	-4.79545	1.95769
H	0.87295	-3.88487	3.47109
H	0.12090	-3.34796	1.92708
C	-0.28030	-1.21457	-0.10773
N	-1.27405	-0.59347	-0.29800
Ge	-2.62921	0.64108	-1.13065
C	-2.38629	2.19080	0.18818
C	-4.28840	-0.35354	-0.33310
C	-2.21535	2.08238	1.60277
C	-2.30816	3.48080	-0.42563
N	-5.45334	0.15149	0.17687
N	-4.56524	-1.66536	-0.60969
C	-1.96989	3.24872	2.36395
C	-2.28694	0.76372	2.34353
C	-2.06158	4.62103	0.37026
C	-2.49887	3.68256	-1.92021
C	-5.67835	1.53720	0.58281
C	-6.44672	-0.83406	0.24015
C	-5.87899	-1.99408	-0.25358
C	-3.61644	-2.59126	-1.22152
H	-1.84668	3.16017	3.45644

C	-1.88955	4.50896	1.75700
H	-3.06176	0.08993	1.92528
H	-1.32887	0.20728	2.26910
H	-2.50091	0.92215	3.41974
H	-2.00985	5.61206	-0.11035
H	-1.77765	3.08820	-2.52032
H	-3.51171	3.37722	-2.26095
H	-2.36965	4.74854	-2.19463
H	-6.05654	1.57482	1.62333
H	-6.41973	2.01972	-0.08593
H	-4.72115	2.08735	0.53048
C	-7.82033	-0.56373	0.76178
C	-6.45676	-3.36262	-0.41647
H	-2.98759	-2.03661	-1.94593
H	-4.16868	-3.38301	-1.75969
H	-2.95577	-3.05296	-0.46124
H	-1.70182	5.40725	2.36682
H	-7.80596	-0.22587	1.82011
H	-8.43790	-1.48030	0.71465
H	-8.34200	0.21948	0.17140
H	-6.50421	-3.67508	-1.48205
H	-7.48999	-3.38936	-0.02205
H	-5.87143	-4.13040	0.13271
C	2.34899	-1.94948	-2.03741
C	1.41875	-3.15306	-2.34732
C	1.89509	-0.68631	-2.79299
C	3.80185	-2.32865	-2.45621
H	1.73361	-4.05727	-1.78972
H	0.37151	-2.91765	-2.07471
H	1.46620	-3.37920	-3.43190
H	2.57201	0.16598	-2.57852
H	1.91686	-0.86978	-3.88627
H	0.86097	-0.39926	-2.51473
H	3.80769	-2.57654	-3.53753
H	4.49223	-1.48206	-2.27759
H	4.16147	-3.21516	-1.89620
C	4.55483	0.38734	-0.17273
N	5.91643	0.22956	-0.12293
N	4.40824	1.75023	-0.23020
C	6.57774	-1.06516	-0.04089
C	6.60866	1.45181	-0.14889
C	5.63733	2.43113	-0.21763
C	3.12034	2.42991	-0.28646
H	7.18193	-1.14720	0.88656
H	7.24806	-1.22993	-0.90978
H	5.80111	-1.85010	-0.03596
C	8.09946	1.55196	-0.10463
C	5.75112	3.92068	-0.26988
H	2.31664	1.67272	-0.26888
H	3.02401	3.02889	-1.21543
H	2.98747	3.10646	0.58267
H	8.52234	1.10763	0.82238
H	8.41752	2.61169	-0.13616
H	8.57962	1.03713	-0.96478
H	5.30072	4.34283	-1.19415
H	6.81412	4.22872	-0.25109
H	5.24974	4.40977	0.59321
H	2.22539	-0.66114	-0.15286

INT-17

C	1.48754	-1.65089	-0.89548
O	1.15281	-2.83604	-0.20148
Si	1.63563	-3.31108	1.39396

C	3.22307	-4.31991	1.26648
H	3.52111	-4.69922	2.26741
H	3.08811	-5.19767	0.60080
H	4.06776	-3.71889	0.87197
C	1.85922	-1.77748	2.46496
H	2.23423	-2.06234	3.47123
H	2.59187	-1.07271	2.01144
H	0.89941	-1.23754	2.60789
C	0.21302	-4.39934	1.97844
H	0.03517	-5.23752	1.27272
H	0.44082	-4.84054	2.97196
H	-0.72916	-3.82043	2.07565
C	0.28685	-0.78556	-0.83923
N	-0.70243	-0.15986	-0.74354
Ge	-2.40184	0.88918	-1.31638
C	-2.06488	2.53027	-0.15642
C	-3.65005	-0.28391	-0.10968
C	-1.55390	2.54993	1.17763
C	-2.31932	3.76769	-0.82948
N	-4.68645	0.07362	0.70798
N	-3.80574	-1.62901	-0.31191
C	-1.30930	3.79319	1.80529
C	-1.25355	1.29441	1.96951
C	-2.06179	4.98630	-0.16566
C	-2.87515	3.82535	-2.24302
C	-4.98538	1.42639	1.17663
C	-5.47405	-1.03571	1.03652
C	-4.90818	-2.12282	0.39360
C	-2.94554	-2.44038	-1.17092
H	-0.92348	3.80598	2.83826
C	-1.55691	5.00242	1.14215
H	-1.95111	0.46670	1.73138
H	-0.23061	0.91716	1.75421
H	-1.30170	1.49049	3.05960
H	-2.26536	5.93621	-0.68641
H	-2.22038	3.30448	-2.97389
H	-3.87870	3.35428	-2.32412
H	-2.98270	4.87459	-2.58205
H	-5.03224	1.44535	2.28312
H	-5.95943	1.76588	0.77066
H	-4.18637	2.11107	0.84002
C	-6.67495	-0.94037	1.91985
C	-5.31205	-3.56144	0.38406
H	-2.60104	-1.82022	-2.02240
H	-3.52220	-3.29499	-1.56875
H	-2.06123	-2.82119	-0.62127
H	-1.36359	5.96138	1.64873
H	-6.41834	-0.56320	2.93268
H	-7.14177	-1.93533	2.04425
H	-7.44660	-0.26144	1.49851
H	-5.61817	-3.90426	-0.62777
H	-6.17505	-3.71955	1.05766
H	-4.49288	-4.22606	0.73089
C	1.91238	-1.92849	-2.38594
C	0.81255	-2.72042	-3.11938
C	2.17160	-0.57525	-3.07986
C	3.21630	-2.75067	-2.34060
H	0.58722	-3.66872	-2.59183
H	-0.12686	-2.13231	-3.20532
H	1.13884	-2.96850	-4.15031
H	2.93528	0.01499	-2.53115
H	2.54285	-0.74294	-4.11183

H	1.24720	0.03632	-3.16249
H	3.56078	-2.97154	-3.37180
H	4.02149	-2.18933	-1.82373
H	3.06489	-3.71505	-1.81689
C	4.05122	0.43394	0.22525
N	5.39482	0.25378	0.44710
N	3.93379	1.80297	0.23140
C	6.02806	-1.05357	0.53083
C	6.09823	1.46118	0.58765
C	5.15578	2.46066	0.44862
C	2.67343	2.50423	0.03902
H	6.52254	-1.19865	1.51374
H	6.78806	-1.18586	-0.26722
H	5.24406	-1.82216	0.40931
C	7.57080	1.53099	0.83583
C	5.29189	3.94853	0.49971
H	1.87593	1.75407	-0.10545
H	2.71023	3.16294	-0.85353
H	2.41908	3.12681	0.92173
H	7.86068	1.02826	1.78403
H	7.90301	2.58459	0.90734
H	8.15644	1.05458	0.01990
H	4.97633	4.43162	-0.45052
H	6.34570	4.23558	0.68035
H	4.68294	4.39790	1.31408
H	2.32489	-1.04931	-0.41766

INT-18

C	2.40887	-1.23618	-0.05133
O	1.19475	-1.35874	-0.30547
C	3.32417	-2.37917	0.28506
C	3.99130	-2.03412	1.64700
H	4.74929	-2.80883	1.88282
H	4.50915	-1.05310	1.62095
H	3.25025	-2.01802	2.47191
H	2.86444	-0.21224	-0.08460
C	2.57943	-3.72210	0.34668
H	2.10052	-3.96633	-0.62299
H	3.29044	-4.53707	0.58883
H	1.79331	-3.71447	1.12883
C	4.41105	-2.38761	-0.83283
H	4.94756	-1.41875	-0.90104
H	5.15969	-3.17071	-0.59378
H	3.97527	-2.62092	-1.82556
Ge	0.00938	0.19896	-1.26676
C	0.80197	1.65973	-0.08868
C	-1.76590	-0.25489	-0.25017
C	0.87414	1.64660	1.34144
C	1.43003	2.72352	-0.81426
N	-2.74963	0.64205	0.06619
N	-2.36954	-1.47939	-0.19054
C	1.57401	2.67863	2.00541
C	0.19432	0.59979	2.20434
C	2.11158	3.73913	-0.10863
C	1.37151	2.82124	-2.32974
C	-2.60332	2.09742	0.09084
C	-3.95966	-0.00999	0.33276
C	-3.71525	-1.36291	0.17491
C	-1.73361	-2.75658	-0.51078
H	1.63324	2.66644	3.10630
C	2.18744	3.71723	1.29052
H	-0.87341	0.85837	2.37828
H	0.20409	-0.41166	1.75669

H	0.66944	0.54021	3.20411
H	2.59400	4.55509	-0.67087
H	1.65834	1.87347	-2.83269
H	0.35064	3.07405	-2.69041
H	2.04962	3.61415	-2.70197
H	-1.53032	2.35602	0.02266
H	-3.00729	2.50385	1.03815
H	-3.15013	2.55738	-0.75728
C	-5.20743	0.71923	0.70984
C	-4.61539	-2.54346	0.33785
H	-0.64665	-2.60872	-0.62468
H	-2.15303	-3.17150	-1.44972
H	-1.90531	-3.48137	0.30880
H	2.72911	4.51209	1.82724
H	-5.09361	1.27594	1.66454
H	-6.04366	0.00721	0.84049
H	-5.51121	1.45070	-0.06863
H	-4.69667	-3.13664	-0.59750
H	-5.63501	-2.21446	0.61231
H	-4.26164	-3.22861	1.13789
**TS-21**			
Si	-2.00725	0.20146	-0.18091
C	0.21080	0.14843	-0.11487
N	1.15398	1.11460	0.09305
N	0.93750	-0.99480	-0.29651
C	0.87736	2.53276	0.28539
C	2.45546	0.58728	0.07124
C	2.31522	-0.76314	-0.17495
C	0.36052	-2.31909	-0.51476
H	1.40589	3.13734	-0.47945
H	1.20498	2.86546	1.29180
H	-0.20728	2.70221	0.19224
C	3.67529	1.42349	0.28529
C	3.34259	-1.84137	-0.30030
H	0.24374	-2.85912	0.44634
H	1.00323	-2.89637	-1.20669
H	-0.64014	-2.21586	-0.97190
H	3.78560	2.21344	-0.48927
H	4.58507	0.79419	0.24636
H	3.66498	1.93112	1.27401
H	3.18592	-2.65258	0.44245
H	4.35538	-1.42751	-0.13237
H	3.34031	-2.30960	-1.30880
C	-2.45519	1.82236	0.73955
H	-2.47759	2.70054	0.05946
H	-3.47793	1.70234	1.15413
H	-1.78923	2.04614	1.59695
C	-1.72397	0.64269	-2.06703
H	-1.39237	-0.24508	-2.65154
H	-2.67308	0.99253	-2.53043
H	-0.95112	1.42955	-2.21155
C	-3.68407	-0.74963	-0.47510
H	-4.21268	-0.94665	0.48212
H	-4.36135	-0.18040	-1.14927
H	-3.51175	-1.73963	-0.95473
C	-1.65962	-0.95949	1.40105
N	-1.41511	-1.69240	2.29200
**INT-19**			
Si	-1.89562	0.22399	-0.24813
C	0.10208	0.20285	-0.32396
N	0.93844	1.25446	-0.08873

N	0.92090	-0.85952	-0.56595
C	0.52437	2.61630	0.23248
C	2.28063	0.85706	-0.16202
C	2.26773	-0.49060	-0.46301
C	0.46779	-2.23941	-0.73207
H	0.87576	3.32399	-0.54535
H	0.94063	2.92016	1.21386
H	-0.57586	2.66016	0.28702
C	3.41624	1.80087	0.06440
C	3.39344	-1.45462	-0.65249
H	0.40969	-2.73411	0.25942
H	1.15878	-2.77870	-1.40657
H	-0.54308	-2.24117	-1.17395
H	3.41086	2.64484	-0.65873
H	4.38223	1.27319	-0.04893
H	3.39579	2.23952	1.08540
H	3.34706	-2.29233	0.07546
H	4.36386	-0.94223	-0.50995
H	3.39861	-1.89754	-1.67196
C	-2.77733	1.41944	0.97062
H	-3.16394	2.34087	0.48531
H	-3.63563	0.86676	1.40993
H	-2.13261	1.69773	1.82979
C	-2.00020	1.33514	-1.89058
H	-1.51551	0.84512	-2.76610
H	-3.06709	1.49838	-2.16732
H	-1.54718	2.34883	-1.80030
C	-2.91006	-1.23871	-0.97804
H	-2.92951	-2.14827	-0.34685
H	-3.95408	-0.86693	-1.07787
H	-2.58301	-1.50158	-2.00689
C	-1.40566	-1.00286	1.47767
N	-0.99693	-1.75487	2.29271

TS-22

Ge	-0.19783	0.09480	-0.78649
C	-0.06413	1.93605	0.11241
C	-2.37718	-0.30165	-0.30857
C	-0.29064	2.24593	1.48738
C	0.32958	2.98169	-0.77825
N	-3.46202	0.52684	-0.16397
N	-2.93012	-1.50808	-0.66056
C	-0.12948	3.57548	1.94050
C	-0.68031	1.18895	2.49940
C	0.48227	4.29845	-0.29006
C	0.58561	2.72097	-2.25308
C	-3.43154	1.94695	0.17642
C	-4.66779	-0.14892	-0.39452
C	-4.32806	-1.45025	-0.70742
C	-2.17877	-2.71761	-0.97460
H	-0.31135	3.80865	3.00294
C	0.25522	4.59774	1.06099
H	-1.37171	0.43622	2.07037
H	0.21474	0.63335	2.86017
H	-1.15485	1.64058	3.39357
H	0.77990	5.09996	-0.98642
H	1.34572	1.92522	-2.41164
H	-0.33285	2.39410	-2.78693
H	0.95203	3.63446	-2.76288
H	-3.91029	2.12155	1.16128
H	-3.97555	2.53270	-0.59082
H	-2.38459	2.28972	0.21673
C	-6.00342	0.51379	-0.29639

C	-5.18952	-2.62397	-1.04511
H	-1.17139	-2.42718	-1.32456
H	-2.69122	-3.27462	-1.78147
H	-2.07107	-3.36694	-0.08421
H	0.37389	5.62951	1.42802
H	-6.17832	0.95380	0.70841
H	-6.81145	-0.21838	-0.48365
H	-6.11937	1.33175	-1.03956
H	-5.03439	-2.97423	-2.08867
H	-6.25810	-2.35468	-0.94681
H	-5.00073	-3.48720	-0.37223
C	2.13704	-0.39273	-0.26371
N	3.19834	0.38335	0.13066
N	2.71834	-1.33594	-1.07849
C	3.12939	1.50673	1.06052
C	4.41092	-0.05682	-0.41980
C	4.10402	-1.16070	-1.19150
C	1.99264	-2.41954	-1.72932
H	3.49992	2.43453	0.58098
H	3.74581	1.30141	1.95944
H	2.07966	1.66651	1.35581
C	5.71861	0.61602	-0.15400
C	4.98243	-2.04756	-2.01392
H	0.93822	-2.10380	-1.85359
H	2.03390	-3.35310	-1.13048
H	2.42125	-2.62018	-2.72948
H	5.72140	1.67181	-0.50011
H	6.53744	0.09256	-0.68300
H	5.97320	0.62412	0.92752
H	4.90964	-3.11210	-1.70476
H	6.04115	-1.74410	-1.90745
H	4.73414	-1.99807	-3.09631
O	-0.29650	-1.74981	1.06448
C	0.53260	-1.99300	1.93741
C	0.35521	-3.03192	3.01033
H	1.49561	-1.40681	1.95455
C	1.60054	-3.91168	3.22119
H	-0.55703	-3.62568	2.79520
H	0.15130	-2.45670	3.94611
H	1.44897	-4.59485	4.07968
H	1.81350	-4.53549	2.32919
H	2.50233	-3.30121	3.43570

3. References

[S1] D. Sarkar, C. Weetman, S. Dutta, E. Schubert, C. Jandl, D. Koley, S. Inoue, *J. Am. Chem. Soc.* **2020**, *142*, 15403−15411.

[S2] M. J. Frisch, G. W. Trucks, H. B. Schlegel, G. E. Scuseria, M. A. Robb, J. R. Cheeseman, G. Scalmani, V. Barone, B. Mennucci, G. A. Petersson, G. H. Nakatsuji, M. Caricato, X. Li, H. P. Hratchian, A. F. Izmaylov, J. Bloino, G. Zheng, J. L. Sonnenberg, M. Hada, M. Ehara, K. Toyota, R. Fukuda, J. Hasegawa, M. Ishida, T. Nakajima, Y. Honda, O. Kitao, H. Nakai, T. Vreven, J. A. Montgomery, Jr., J. E. Peralta, F. Ogliaro, M. Bearpark, J. J. Heyd, E. Brothers, K. N. Kudin, V. N. Staroverov, R. Kobayashi, J. Normand, K. Raghavachari, A. Rendell, J. C. Burant, S. S. Iyengar, J. Tomasi, M. Cossi, N. Rega, J. M. Millam, M. Klene, J. E. Knox, J. B. Cross, V. Bakken, C. Adamo, J. Jaramillo, R. Gomperts, R. E. Stratmann, O. Yazyev, A. J. Austin, R. Cammi, C. Pomelli, J. W. Ochterski, R. L. Martin, K. Morokuma, V. G. Zakrzewski, G. A. Voth, P. Salvador, J. J. Dannenberg, S. Dapprich, A. D. Daniels, O. Farkas, J. B. Foresman, J. V. Ortiz, J. Cioslowski, D. J. Fox, *Gaussian 09*, Revision D.01; Gaussian, Inc., Wallingford, CT, **2013**.

[S3] A. D. Becke, *Phys. Rev. A: At., Mol., Opt. Phys.* **1988**, *38*, 3098−3100.

[S4] J. P. Perdew, *Phys. Rev. B: Condens. Matter Mater. Phys.* **1986**, *33*, 8822−8824.

[S5] F. Weigend, R. Ahlrichs, *Phys. Chem. Chem. Phys.* **2005**, *7*, 3297−3305.

[S6] F. Weigend, *Phys. Chem. Chem. Phys.* **2006**, *8*, 1057−1065.

[S7] T. A. Halgren, W. N. Lipscomb, *Chem. Phys. Lett.* **1977**, *49*, 225−232.

[S8] Y. Zhao, N. E. Schultz, D. G. Truhlar, *J. Chem. Theory Comput.* **2006**, *2*, 364−382.

[S9] S. Grimme, J. Antony, S. Ehrlich, H. Krieg, *J. Chem. Phys.* **2010**, *132*, 154104−154119.

[S10] (75) K. B. Wiberg, *Tetrahedron* **1968**, *24*, 1083−1096.

[S11] A. V. Marenich, C. J. Cramer, D. G. Truhlar, *J. Phys. Chem. B* **2009**, *113*, 6378−6396.
[S12] J. Cooper, T. Ziegler, *Inorg. Chem.* **2002**, *41*, 6614−6622.
[S13] a) M. Mitoraj, A. Michalak, *Organometallics* **2007**, *26*, 6576−6580; b) M. P. Mitoraj, A. Michalak, T. Ziegler, *J. Chem. Theory Comput.* **2009**, *5*, 962−975.
[S14] G. te Velde, F. M. Bickelhaupt, E. J. Baerends, C. Fonseca Guerra, S. J. A. Van Gisbergen, J. G. Snijders, T. Ziegler, *J. Comput. Chem.* **2001**, *22*, 931−967.
[S15] E. Van Lenthe, E. J. Baerends, *J. Comput. Chem.* **2003**, *24*, 1142−1156.
[S16] J. G. Snijders, P. Vernooijs, E. J. Baerends, *At. Data Nucl. Data Tables* **1981**, *26*, 483−509.
[S17] E. van Lenthe, E. J. Baerends, J. G. Snijders, *J. Chem. Phys.* **1993**, *99*, 4597−4610.
[S18] G. A. Andrienko, http://www.chemcraftprog.com.
[S19] C. Y. Legault, *CYLView, 1.0b,* Université de Sherbrooke, **2009**. See http://www.cylview.org.
[S20] Q. Zhou, Y. Li, *J. Am. Chem. Soc.* **2015**, *137*, 10182−10189.
